# Habitat- and soil-related drivers of the root-associated fungal community of *Quercus suber* in the Northern Moroccan forest

**DOI:** 10.1371/journal.pone.0187758

**Published:** 2017-11-20

**Authors:** Fatima Zahra Maghnia, Younes Abbas, Frédéric Mahé, Benaissa Kerdouh, Estelle Tournier, Mohamed Ouadji, Pierre Tisseyre, Yves Prin, Naïma El Ghachtouli, Salah Eddine Bakkali Yakhlef, Robin Duponnois, Hervé Sanguin

**Affiliations:** 1 Forestry research center, Rabat, Morocco; 2 CIRAD, UMR LSTM, Montpellier, France; 3 LSTM, Univ Montpellier, CIRAD, IRD, INRA, Montpellier SupAgro, Montpellier, France; 4 IRD, UMR LSTM, Montpellier, France; 5 Polyvalent Laboratory, Multidisciplinary Faculty, University of Sultan Moulay Slimane, Béni Mellal, Morocco; 6 Laboratory of Microbial Biotechnology, Faculty of Sciences and Technology, University of Sidi Mohamed Ben Abdellah, Fez, Morocco; 7 Directorat for Higher Education, Training and Research, Ministry of Agriculture and Fisheries, Morocco; Estacion Experimental del Zaidin, SPAIN

## Abstract

Soil fungi associated with plant roots, notably ectomycorrhizal (EcM) fungi, are central in above- and below-ground interactions in Mediterranean forests. They are a key component in soil nutrient cycling and plant productivity. Yet, major disturbances of Mediterranean forests, particularly in the Southern Mediterranean basin, are observed due to the greater human pressures and climate changes. These disturbances highly impact forest cover, soil properties and consequently the root-associated fungal communities. The implementation of efficient conservation strategies of Mediterranean forests is thus closely tied to our understanding of root-associated fungal biodiversity and environmental rules driving its diversity and structure. In our study, the root-associated fungal community of *Q*. *suber* was analyzed using high-throughput sequencing across three major Moroccan cork oak habitats. Significant differences in root-associated fungal community structures of *Q*. *suber* were observed among Moroccan cork oak habitats (Maâmora, Benslimane, Chefchaoun) subjected to different human disturbance levels (high to low disturbances, respectively). The fungal community structure changes correlated with a wide range of soil properties, notably with pH, C:N ratio (*P* = 0.0002), and available phosphorus levels (*P* = 0.0001). More than 90 below-ground fungal indicators (*P* < 0.01)–either of a type of habitat and/or a soil property–were revealed. The results shed light on the ecological significance of ubiquitous ectomycorrhiza (*Tomentella*, *Russula*, *Cenococcum*), and putative sclerotia-associated/ericoid mycorrhizal fungal taxa (*Cladophialophora*, *Oidiodendron*) in the Moroccan cork oak forest, and their intraspecific variability regarding their response to land use and soil characteristics.

## Introduction

Soil fungi are one of the most diverse groups of organisms on Earth [1] colonizing a wide range of ecological niches [2] and playing a central role in major ecological and biogeochemical processes, notably in forests [3,4]. Yet, forest ecosystems are highly threatened by global changes [5,6]. Recent extreme droughts in Southern Mediterranean forests have increased tree mortality [5]. This negative impact has been exacerbated by increasing human pressures such as deforestation and overharvesting [7]. In this context, the Mediterranean cork oak (*Quercus suber*) forest (covering more than 20,000 square kilometers) is particularly at risk due to bark exploitation, intensive agro-silvo-pastoral management, and oak decline [7]. The degradation of cork oak forests strongly affects soil fungi, notably ectomycorrhizal (EcM) fungi [8–10], a key component of nutrient cycling and plant productivity [11]. Conservation strategies of these forests are dependent on our understanding of the soil fungal biodiversity, notably the fungal components directly associated with plant roots. The conservation of soil fungi themselves has seldom been considered compared to other Eukaryotes [12] and initiatives are relatively recent (http://iucn.ekoo.se/en/iucn/welcome). For instance, in December 2016, 34 fungal species mostly distributed in Europe, were present in the IUCN Red List of threatened species (http://www.iucnredlist.org). The diversity and dynamic of soil and root-associated fungal communities in cork oak ecosystems has been mainly investigated in the Northern Mediterranean basin [8–10,13,14]. Hitherto, estimations of soil fungal diversity in Southern cork oak forests have been almost exclusively based on fungal sporocarp surveys [15], but this approach has been shown to provide a partial view of true below-ground soil fungal diversity, even for the EcM community [16]. To address the challenges of cork oak forest conservation, soil fungal diversity in Southern cork oak ecosystems must be more extensively explored and environmental drivers affecting fungal diversity identified. The influence of environmental factors has been shown to vary with the type of habitats, the spatial scale considered and the fungal taxa analyzed [2,17,18]. For instance, variations in phosphate (P) and nitrogen (N) soil content have been suggested as important drivers of intraspecific variability of *Pisolithus* spp. in the Maâmora habitat, the largest cork oak forest in Morocco [19].

The main goals of the present study are (i) to characterize in depth the molecular diversity of the root-associated fungal community of *Q*. *suber* (fungal endophytes, EcM fungi and fungi in adherent soil); (ii) to determine the main environmental factors (habitats, soil characteristics) driving this fungal community; and (iii) to identify fungal indicators associated with a type of habitat and/or a soil property.

## Material and methods

### Study site and sampling

The study was conducted in three habitats of the Moroccan cork oak forest, located in the Moroccan Northern Mountains known as “Chefchaoun” (35°15’5.14”N 005°30’6.68”W, 1534 m elevation), and in the lowland bordering the Atlantic Ocean (North-West of Morocco) known as “Maâmora” (34°17’06.186”N 6°28’30.792”W, 27 m elevation) and “Benslimane” (33°41’9.85”'N, 6°54’7.26”W; 326 m elevation). The three habitats are under a Mediterranean-type climate characterized by hot and dry summers, mild and wet winters, and mean annual rainfall levels of 453 mm (Benslimane), 570 mm (Maâmora) and 880 mm (Chefchaoun). They are characterized by an abundant understory, notably *Cistus salviifolius*, *Lavandula stoechas*, and *Thymeleae lythroides* for Maâmora, and *Arbutus unedo* and *Pistacia lentiscus* for Benslimane, and *Erica arborea* and *Arbutus unedo* for Chefchaoun. Twenty seven cork oak trees were sampled between February and June 2013. The sampling design was based on the selection of three plots per forest spaced 100 meters apart, each composed of three trees 20–30 meters away from any other. Roots with soil were sampled under the crown of each tree and stored at +4°C. The roots were rinsed under tap water to remove the non-adherent soil and observed under a binocular microscope to select root zones rich in ectomycorrhizal (EcM) fungi, dried and stored at -20°C. The product of this sampling process resulted in a fungal community including fungal endophytes, EcM fungi and fungi in adherent soil. This fungal community is hereafter named “root-associated fungal community”.

Soil physico-chemical parameters were measured at the LAMA Laboratory (Dakar, Senegal): pH, total nitrogen (N), total carbon (C), Carbon:Nitrogen ratio (C:N ratio), total and available phosphate (P), K^+^, Mg^2+^, Na^+^, Cation exchange capacity (CEC). The habitats are managed by the High Commission for Water and Forests and Combatting Desertification. The permissions for root and soil sampling were provided by the Forestry Research Centre of Rabat (Morocco).

### DNA extraction, ITS amplification and Illumina Miseq sequencing

For each of the 27 cork oak tree samples, all root pieces and the adherent soil were subjected to liquid nitrogen grinding for homogeneization. The total DNA was extracted from a sub-sample (70–80 mg) using a FastPrep-24 homogenizer (MP biomedicals Europe, Illkirch, France) and the FastDNA® SPIN kit (MP biomedicals Europe) according to the manufacturer’s instructions. The purity of DNA extracts was improved by adding 20–30 mg Polyvinylpolypyrrolidon (PVPP) during the first step of DNA extraction in order to avoid the presence of PCR inhibitors.

The Internal transcribed spacer ITS1 of the nuclear ribosomal RNA was amplified using the primers ITS1FI2 (5’-GAACCWGCGGARGGATCA-3’) and ITS2 (5’-GCTGCGTTCTTCATCGATGC-3’) [20]. The amplification reaction was performed in a final volume of 25 μl with the primers ITS1FI2 and ITS2 (0.6 μM each), 2 μl of DNA extract, 200 μM of each dNTP, 200 ng/ml BSA, GoTaq® DNA Polymerase (2 units) and 1X Green GoTaq® Reaction Buffer (Promega, Charbonnieres, France), with the following cycling conditions: 95°C for 15 min; 30 cycles of 95°C for 30 s, 58°C for 30 s, 72°C for 30 s; a final elongation step at 72°C for 5 min. To increase richness recovery and to limit PCR biases, three PCR replicates per sample were pooled and purified using an illustra GFX PCR DNA and Gel Band Purification Kit (GE Healthcare Life Sciences, Velizy-Villacoublay, France) following manufacturer’s guidelines. All amplicon products were subjected to paired-end Illumina MiSeq sequencing (2×300 bp) by Molecular Research LP (MR DNA, TX, USA).

### Bioinformatic data processing

Paired Illumina MiSeq reads were assembled with vsearch v1.11.1 [21] using the command fastq_mergepairs and the option fastq_allowmergestagger. Demultiplexing and primer clipping was performed with cutadapt v1.9 [22], enforcing a full-length match for sample tags and allowing a 2/3-length partial match for forward and reverse primers. Only reads containing both primers were retained. For each trimmed read, the expected error was estimated with vsearch’s command fastq_filter and the option eeout. Each sample was then dereplicated, i.e. strictly identical reads were merged, using vsearch’s command derep_fulllength, and converted to FASTA format.

To prepare the clustering, the samples were pooled and submitted to another round of dereplication with vsearch. Files containing expected error estimations were also dereplicated to retain only the lowest expected error for each unique sequence. Clustering was performed with swarm v2.1.8 [23], using a local threshold of one difference and the fastidious option. Molecular operational taxonomic unit (OTU) representative sequences were then searched for chimeras with vsearch’s command uchime_denovo [24]. In parallel, representative sequences received taxonomical assignments using the stampa pipeline (https://github.com/frederic-mahe/stampa) and a custom version of the fungal reference database UNITE v7 (https://unite.ut.ee/; [25]). In brief, the stampa pipeline requires the reference sequences to be trimmed with cutadapt, using the same primers as those used for the amplification of the environmental sequences. Using vsearch’s exact global pairwise comparisons, each environmental sequence is compared to all reference sequences and is assigned to the closest hit. In case of a tie, the environmental sequence is assigned to the last-common ancestor of the co-best hits. The abbreviation “cf.” is used throughout the text for interpretation at the species level because of potential taxonomic biases relative to length and variability of the amplified region (ITS1 region). As the UNITE database contains only fungal sequences, 20 sequences were added to our custom reference database to identify the plants also amplified by our ITS primers (see S1 File).

Clustering results, expected error values, taxonomic assignments and chimera detection results were used to build a raw OTU table (script available in **S1 File**). Up to that point, reads that could not be merged, reads without tags or primers, reads shorter than 32 nucleotides and reads with uncalled bases (“N”) were eliminated. To create the “cleaned” OTU table, additional filters were applied to retain: non-chimeric OTUs, OTUs with an expected error divided by length below 0.0002, OTUs containing more than 3 reads or seen in 2 samples, OTUs assigned to plant or fungal taxa with at least 80% similarity or containing more than 10,000 reads. All codes and representative sequences of OTUs can be found in HTML format (**S1 File**) and raw data are available under the BioPproject ID PRJNA378471 (https://www.ncbi.nlm.nih.gov/bioproject).

### Statistics

Diversity (Shannon, inverse Simpson [1/D]), richness (number of MOTUs, Chao1) and evenness (Pielou) indexes were estimated using R [26] and the R package vegan version 2.4–3 [27], and differences among cork oak habitats were assessed by non-parametric permutational multivariate analysis of variance (PERMANOVA), as implemented in the *perm*.*anova*() function from the R package RVAideMemoire version 0.9–65 [28].

Fungal community membership was assessed using venn diagram analysis with the R package VennDiagram version 1.6.17 [29]. The differences in fungal community structure among the three habitats were displayed with nonmetric multi-dimensional scaling (NMDS) implemented in the *metaMDS*() function. Significance in fungal community structure variation was also assessed using PERMANOVA in the *adonis*() function. Multivariate dispersion was estimated using the *betadisper*() function and *permutest*() as it can affect PERMANOVA results. Soil parameters were fitted to the NMDS using the *envfit*() function (9,999 permutations). Correlation among soil parameters was assessed using the Pearson correlation coefficient, as implemented in the *cor*.*test*() function. All functions are available in the R package vegan. Table transformations in R were performed with the tidyverse packages version 1.1.1 [30], and plots were visualized with the packages ggplot2 version 2.2.1 [31] for NMDS and ggtern version 2.2.1 [32] for ternary plots.

The presence of fungal indicator species of a specific type of habitat (Maâmora, Benslimane, Chefchaoun) was determined using the corrected Pearson’s phi coefficient of association (r.g; 9,999 permutations) implemented in the *multipatt*() function from the R package indicspecies [33]. Fungal indicator species with respect to soil properties (pH, C:N ratio, and available P) were determined using the indicator value (IndVal) index, as implemented in indicspecies’ *multipatt*(). Two different probabilities were calculated, *i*.*e*. A (specificity), representing the probability of a site to be defined by a given soil property, given that the species have been detected, and B (sensibility) representing the probability of finding the species in different sites characterized by a given soil property. We considered as valid indicators the OTUs showing both A (specificity) and B (fidelity) superior to 0.8 and 0.6 respectively, as recommended in [18].

## Results

### Composition of cork oak root-associated fungal community

A filtered dataset of 792,931 sequences was obtained from a raw dataset of 1,129,145 trimmed- and paired-sequences (see material and methods for more details). Sixty percent of sequences were affiliated to ITS sequences from plants (Streptophyta). Overall, a dataset of 315,597 fungal sequences (27 samples) was rarefied down to 3,447 sequences per sample to improve the robustness of fungal community comparison among the three habitats [34].

Analysis of taxonomic fungal community composition in the Moroccan cork oak forest detected 1,768 OTUs belonging to 4 known fungal phyla, 39 orders, 78 families, and 127 genera (**S1 Table**). Two percent of OTUs were uncharacterized at the phylum level (about 0.2% of sequences), and 43% at the genus level (about 30% of sequences). The root-associated fungal community of *Q*. *suber* was mostly composed of Basidiomycota and Ascomycota, 60% and 39%, respectively. However, a higher number of genera was found for Ascomycota compared to Basidiomycota, 94 and 31, respectively. The ten most abundant fungal orders (89% of sequences) were Thelephorales (17,084 sequences), Russulales (16,275), Agaricales (14,017), Hysteriales (12,815), Sebacinales (5,902), Chaetothyriales (5,622), Heliotales (4,796), Hypocreales (2,575), Capnodiales (1,895), and Eurotiales (1,690) (**S1 Table**). The ten most abundant fungal genera (55% of sequences) were *Cenococcum* (12,459 sequences), *Tomentella* (11,609), *Russula* (7,019), *Inocybe* (4,744), *Cortinarius* (4,568), *Cladophialophora* (3,046), *Hygrophorus* (2,643), *Lactarius* (2,196), *Sebacina* (1,667), and *Cryptosporiopsis* (1,317) (**S1 Table**).

### Habitat-related root-associated fungal indicators

No habitat-related impact (Maâmora, Benslimane, Chefchaoun) was detected on root-associated fungal community richness, diversity and equitability (**Table 1**), except for Chao1 estimation between Benslimane and Chefchaoun (*P* < 0.015).

**Table 1:** Alpha diversity of root-associated fungal communities of *Q*. *suber* in Moroccan cork oak forests.

	MOTUs number (Richness)	Chao1 (Richness)	Shannon’s index (diversity)	Inverse Simpson’s index (diversity)	Pielou’s index (Evenness)
Maâmora	211±30 a ^2^	476±67 ab	2.74±0.22 a	6.17±1.50 a	0.31±0.10 a
Benslimane	183±69 a	365±119 a	2.34±0.90 a	6.27±4.55 a	0.27±0.06 a
Chefchaoun	239±47 a	509±101 b	2.97±0.55 a	8.42±6.52 a	0.34±0.02 a
Forest	ns ^1^	*	ns	ns	ns

^1^ Statistics were performed using PERMANOVA (Habitat type as factor). Data in the same column followed by the same letter are not significantly different according to PERMANOVA (*P* < 0.05).

‘*’ *P* < 0.05; ‘ns’ non significant.

^2^ Values indicate mean ± standard deviation.

**Fig 1:**
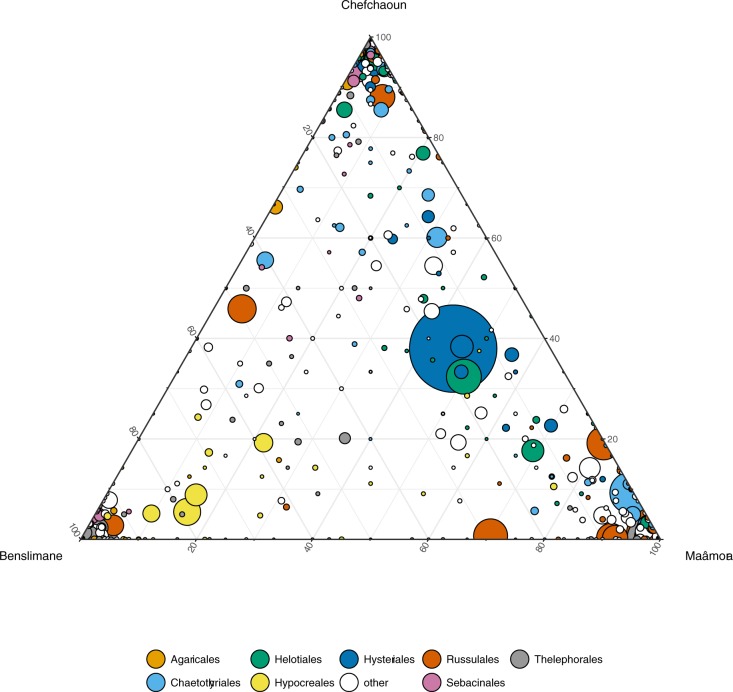
Distribution of fungal OTUs among habitats (Maâmora, Benslimane, Chefchaoun) in a ternary plot. A circle represents an OTU, with the circle size corresponding to the abundance (number of sequences) of the OTU. The color indicates the taxonomic affiliation of OTUs at order level. The category “others” corresponds to all OTUs, except the one belonging to the eight most abundant orders).

**Fig 2:**
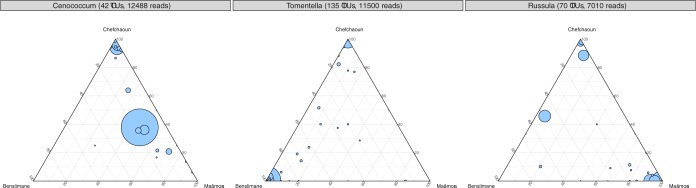
Distribution of fungal OTUs among habitats (Maâmora, Benslimane, Chefchaoun) at genus level in ternary plots. The three most abundant genera are indicated, (A) *Cenococcum* (45 OTUs, 12,516 sequences), (B) *Tomentella* (140 OTUs; 11,605 sequences), (C) *Russula* (64 OTUs, 7,027 sequences). A circle represents an OTU, with the circle size corresponding to the abundance (number of sequences) of the OTU.

By contrast, analysis of fungal OTU distribution in ternary plots revealed strong fungal community patterns (abundance and membership) among habitats (**Fig 1**). Russulales (40% of sequences) was the most abundant fungal order in Maâmora, Thelephorales (41%) in Benslimane, and Hysteriales (20% of sequences) and Agaricales (19%) in Chefchaoun. At least 65% of OTUs were habitat-specific (318 OTUs for Maâmora, 378 for Benslimane, 461 for Chefchaoun), but these accounted for only 6% of total sequences (**S1 Fig**). The most abundant taxonomic orders for each habitat (> 50% sequences) composed of habitat-specific OTUs were Trechisporales, Agaricales, Russulales in Maâmora, Thelephorales in Benslimane, and Agaricales, Cantharellales, Heliotales, Sebacinales in Chefchaoun. Meanwhile, 17% of OTUs were shared among the three habitats, representing 84% of total sequences (**S1 Fig**). However, strong differences were observed for shared OTUs among habitats in terms of relative abundance. For instance, few sequences belonging to Agaricales OTUs were present in Maâmora (). A high variability in OTU abundance and distribution was also observed among habitats for the most abundant genera (**Fig 2**). Among the three most abundant fungal genera, *Cenococcum* and *Russula* were predominant in Maâmora and Chefchaoun, whereas *Tomentella* was more predominant in Benslimane and Chefchaoun (**Fig 2**).

**Fig 3:**
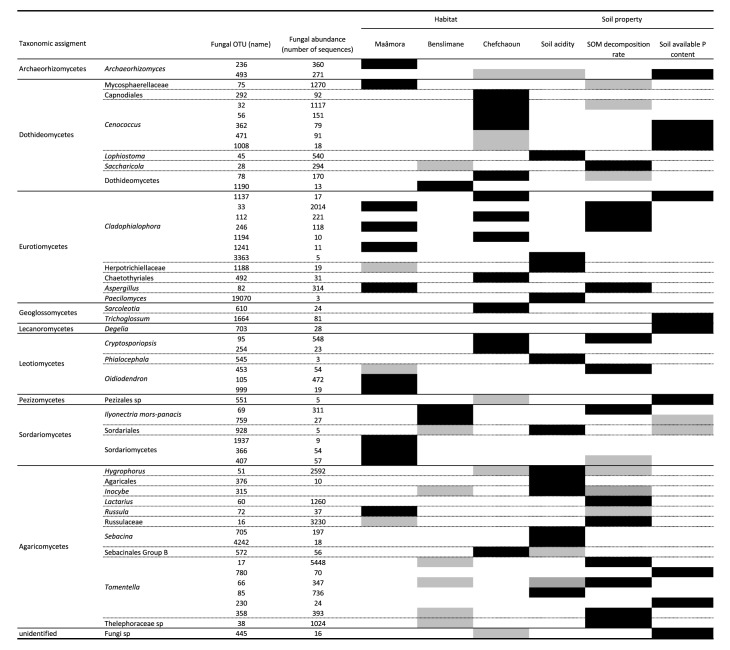
Major fungal indicators of habitat and soil property. Only fungal indicators with a significant association with at least one ecological condition (black box) are shown, i.e. fungal OTUs associated with a habitat with r.g > 0.5 and *P* < 0.01 and/or a soil property with A > 0.8, B > 0.6 and *P* < 0.01. For certain major fungal indicators, a low but significant association with a habitat (r.g > 0.5 and 0.01 < *P* < 0.05) and/or a soil property (A > 0.8, B > 0.6 and 0.01 < *P* < 0.05) was also revealed (gray box). See S3 and S6 Tables for details. The taxonomic assignment is provided until genus level, except for those with an unidentified genus (where higher taxonomic level is indicated).

Permutational multivariate analysis of variance (PERMANOVA) based on the Bray-Curtis dissimilarity matrix confirmed that cork-oak habitats are significant drivers of root-associated fungal community structures (**Table 2**). Non-significant multivariate dispersion of data was detected, emphasizing the robustness of PERMANOVA results (**Table 2**). Pairwise PERMANOVA comparisons of cork oak habitats showed significant differences between root-associated fungal communities (**S2 Table**). Indicator species analysis revealed 40 OTUs significantly associated with Maâmora, 21 to Benslimane and 49 to Chefchaoun (**S3 Table**). The most significant OTUs (r.g > 0.5; *P* < 0.01) (**Fig 3**) belonged almost exclusively to Ascomycota, and were affiliated to Archaeorhizomycetaceae, Herpotrichiellaceae, Mycosphaerellaceae, Myxotrichaceae, Trichocomaceae, and unidentified Sordariomycetes for Maâmora; unidentified Dothideomycetes and Sordariomycetes for Benslimane; Dermateaceae, Geoglossaceae, Gloniaceae, Herpotrichiellaceae, Dothideomycetes, and Eurotiomycetes for Chefchaoun (**S1 Table**).

**Table 2:** Impact of forest habitat on cork oak root-associated fungal community structures.

Model/Factors	Df	SS	MS	F. Model	R2	*P*-value ^1^
PERMANOVA						
Habitat	2	1.701	0.85356	2.3665	0.16473	0.001
Residuals	24	8.6563	0.36068		0.83527	
Total	26	10.3634			1	
BETADISPER						
Habitat	2	0.033575	0.0167876	2.9099		0.077
Residuals	24	0.138459	0.0057691			

Df, degrees of freedom; SS, sum of squares; MS, mean squares; R2, R-squared; F.Model, F value by permutation.

^1^ The significance of multivariate analysis of variance and dispersion was assessed with a permutational test (Iterations = 999).

### Soil-related drivers of root-associated fungal community structures

Soil characteristics in the Moroccan cork oak forest were investigated (**S4 Table**) in order to identify the main abiotic soil parameters driving root-associated fungal community structures. Correlation analysis between each of the soil parameters (**S5 Table**) revealed strong positive correlations among almost all of them, with the notable exceptions of (i) the C:N ratio, which is not correlated with pH, total N, total C, available P, Na^+^ and K^+^; and (ii) the available P, which is not correlated with pH and total N. Available P, Mg^2+^, K^+^ and Cation exchange capacity were the soil parameters the most strongly correlated (*r* > 0.75) with others.

**Fig 4:**
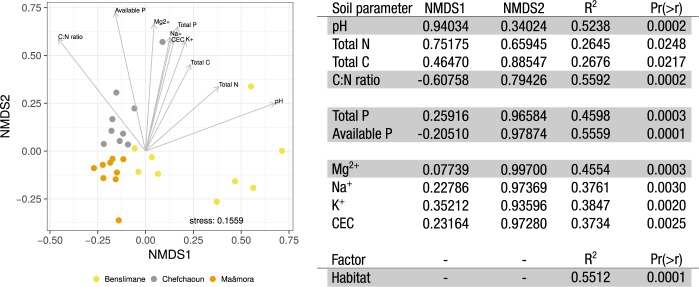
Nonmetric multidimensional scaling analysis of *Q*. *suber* root-associated fungal community structures (OTU level) in Moroccan cork oak habitats (Maâmora, Benslimane and Chefchaoun) and soil parameter fitting. The stress of the ordination is 0.1584. All significant soil parameters are shown by arrows (the length is proportional to the strength of the correlation. Factor fitting (Habitat) is indicated below the soil parameter. The most significant soil parameters and factor (*P* < 0.001) are indicated in bold in the table.

NMDS ordination showed the habitat effect on root-associated fungal community structure (**Fig 4**), which was confirmed by a significant correlation (R^2^ = 0.5512, *P* = 0.0001). All ten soil parameters fitted in NMDS were also significantly correlated with the root-associated fungal community structure (**Fig 4**). The Maâmora habitat appeared as the most different in terms of soil characteristics compared to Benslimane and Chefchaoun habitats. The three most significant drivers of root-associated fungal community structures were pH, C:N ratio (*P* = 0.0002), and available P (*P* = 0.0001). Analysis of fungal indicators with respect to these three main soil properties revealed the significant association (A [specificity] > 0.8; B [sensitivity] > 0.6; *P* < 0.05) of 27 OTUs with soil acidity levels (pH), 28 OTUs with soil organic decomposition rates (C:N ratio), and 60 OTUs with soil available P contents (**S6 Table**). As observed for the fungal indicators of habitat, a higher diversity of Ascomycota than Basidiomycota (only Agaricomycetes) was significantly associated with soil properties. Among the most significant OTUs (*P* < 0.01), seven OTUs affiliated to *Cenococcum*, *Cladophialophora*, *Aspergillus*, *Cryptosporiopsis*, and *Ilyonectria*, were also indicators of a habitat (**Fig 3**).

## Discussion

We conducted an in-depth investigation of the molecular diversity of the root-associated fungal community of *Q*. *suber* in the Moroccan cork oak forest to decipher the main environmental (habitats, soil parameters) drivers of this fungal community and to characterize fungal indicators associated with a type of habitat and/or a soil property.

### Ectomycorrhiza but also ericoid mycorrhiza are predominant in Moroccan cork oak root-associated fungal community

EcM fungi (notably *Cenococcum*, *Tomentella*, *Russula*, *Inocybe*, *Cortinarius*) were the most abundant fungal groups associated with the roots, as previously observed in most oak forests [13,18,35–39]. Fungi belonging to *Tomentella*, *Russula*, and *Cenococcum* were proposed as major actors of forest ecosystems under drought conditions [8], and the predominance of *Cenococcum* and *Tomentella* confirmed previous EcM surveys based on morphological identification in the Maâmora habitat [40]. *Cenococcum* is one of the most common and abundant fungi in forest ecosystems [41,42], but over 40 different OTUs were unequally distributed in the three forest habitats, confirming reports of intraspecific diversity at local and large geographical scales [43–45]. Remarkably, potential parasites of *Cenococcum* sclerotia [46], *Cladophialophora* and *Oidiodendron* (10% of Ascomycota sequences) were present along with *Cenoccocum*. An in-depth investigation of ecological interactions between sclerotia-associated fungi and *Cenococcum* may thus be important for a better understanding of fungal assembly rules affecting forest ecosystem functioning, particularly in Mediterranean drought scenarios.

By contrast, well-known oak-associated EcM fungal taxa, i.e. *Tuber* [47] and *Pisolithus* [15], represented a minor part of the root-associated fungal community of *Q*. *suber* (0.4% and < 0.00001% of total sequence number, respectively). The recently-characterized *Tuber* cf. *cistophilum*, isolated from Spanish acidic argillaceous soils [48], constituted 7% of *Tuber* abundance. Similarly, *Terfezia*, a genus of truffle-like fungi that is an important edible fungus for local Moroccan inhabitants, was also detected. The main species detected was *Terfezia* cf. *pini*, but at low abundance (0.0005% of total sequence number). Whereas, the low abundance of *Tuber* had been previously observed in the Sardinian cork oak forest [10], *Pisolithus* had been described as predominant in different Moroccan cork oak habitats [15]. Discrepancy in EcM community composition and abundance may be due to method-dependent biases as previously highlighted in other fungal surveys [9,10,38,49].

Surprisingly, ericoid mycorrhizal (ErM) fungi, *Oidiodendron* [50] and *Cryptosporiopsis* [51], were relatively abundant in cork oak roots analyzed in the current study. The presence of *Oidiodendron* members had been also evidenced in EcM roots of *Quercus ilex* [50]. Two principal ecological implications of EcM-ErM interactions in forest ecosystem functioning have been proposed by [50], (i) nutrient exchanges among EcM and ErM plants through hyphal links of shared mycorrhizal fungi, and (ii) EcM plants acting as an ErM fungal reservoir for the efficient land recolonization of ErM plants. The significance of the second hypothesis is of particular importance for cork oak ecosystems, strongly affected by fire events in the Mediterranean basin [52]. It also raises the question: could other fungi, such as arbuscular mycorrhizal (AM) fungi, play a role in EcM plants? Evidence of AM fungal colonization of EcM plants and their functional role has been demonstrated [53–55]. However, AM fungi generally constitute a minor part of the total fungal community in EcM-dominated ecosystems [14]. In the current work, AM fungi were almost absent from the raw data using the ITS-based approach. A more AM specific approach based on the 18S rRNA gene could address AM fungal community structures in EcM ecosystems [56,57].

### Ecological specificity of Moroccan cork oak root-associated fungal community

A worldwide fungal survey highlighted differences between Mediterranean forests and temperate deciduous forests regarding EcM fungal communities [2]. The current results strengthened the specificity of the Mediterranean oak-associated EcM fungal community compared to Northern European oak forests. Indeed, the predominance of the three main EcM families, Thelephoraceae, Russulaceae and Gloniaceae in different Mediterranean oak forests [8,9,58] were confirmed in the current study. These accounted for almost half of total root-associated fungal abundance, whereas Cortinariaceae constituted one of the three predominant EcM families in Northern European oak forests [18,59]. The stronger predominance of Gloniaceae (*Cenococcum* spp.) in Mediterranean forests may be linked to its resistance to drought conditions [60,61] and thus also to precipitation rates [35].

At the Mediterranean scale, results also highlighted the ecological specificity of Moroccan cork oak root-associated fungal community. *Lactarius chrysorrheus*, a predominant *Russulaceae* species in the Sardinian cork oak forest [10], widely distributed in Mediterranean oak forests [8,9,13,35,38], was not detected in the present study. Nevertheless, other known species of *Lactarius* were detected, notably *Lactarius* cf. *quietus*, which is considered as an oak specialist [18]. However, *L*. *quietus* distribution appears more related to Northern Europe oak ecosystems [18,59] than Mediterranean oak ecosystems [8,38]. A wide range of *Russula* (*Russula* cf. *heterophylla*, *Russula* cf. *olivobrunnea*, *Russula* cf. *violeipes*) and *Inocybe* (*Inocybe* cf. *bresadolae*, *Inocybe* cf. *glabripes*, *Inocybe* cf. *posterula*, *Inocybe* cf. *subporospora*) species were detected specifically in Morocco and not in other Mediterranean cork oak forests [9,10,13,15,62]. By contrast, *Russula* cf. *decipiens* was also described in Corsica [62], *Russula* cf. *foetens* in Portugal [8], and *Russula* cf. *odorata* in Corsica and Sardinia [10,14,62], and only *Inocybe* cf. *asterospora* was described in the Portuguese cork oak forest [8]. Surprisingly, *Laccaria* and *Tricholoma*, two EcM Agaricales frequently observed at above and below-ground levels in Mediterranean oak ecosystems [9,10,13,14,38,39] were not detected in the current work. These two fungal genera had been however observed in the Moroccan cork oak forest at above-ground level, reflecting, as for *Pisolithus*, the discrepancy between above- and below-ground fungal abundance. Remarkably, *Archaeorhizomyces*, a genus of ubiquitous root endophyte fungi [63], was relatively abundant (> 3% of Ascomycota) in Moroccan cork oak roots. The presence of this genus in Mediterranean ecosystem surveys is rarely described and few data are available regarding its ecological role, but a continuum from root endophytic to free-living saprophytic life strategies has been proposed [64].

### Land use and soil parameters are major drivers of Moroccan cork oak root-associated fungal community

More than 90 fungal OTUs were significantly (*P* < 0.01) associated with habitat and/or soil properties, notably EcM and ErM fungi, underlining the value of fungi as below-ground indicators of forest status and environmental conditions [18,65]. The high number of habitat-associated fungal OTUs for Maâmaora and Chefchaoun suggested their strong ecological specificity. Differences in human pressure levels may explain a part of these specificities since Maâmora is more disturbed (intensive cork exploitation, overgrazing, aging of tree population) than Chefchaoun (lower human pressure, natural tree regeneration). Remarkably, an intraspecific *Cenococcum* ecological preference related to land use and succession stages (association with Chefchaoun, low disturbances), and not only to drought resistance, was shown in the Moroccan cork oak forest. However, *Cenococcum* is usually described as a multi-stage fungus due to its worldwide predominance and stability in forest ecosystems [41,42,58]. Certain *Russula* OTUs might also be seen as indicators of disturbance levels (association with Maâmora, high disturbances). *Russula* is generally considered as a late-successional fungal genus [62,66,67], which may explain its high sensitivity to land use as observed in the current study and in the Portuguese cork oak forest (Azul et al., 2009), but also its reliability as an indicator of ecosystems characterized by the aging of cork oak populations (Maâmora being an open forest with low regeneration rate). *Russula* was also shown as highly sensitive to soil nitrogen levels, seasons, and cork oak decline [18,40,58,68], and whereas no decline symptoms were observed, N total content was significantly correlated to differences in the root-associated fungal community structure among the three cork oak habitats. The potential status of *Tomentella* as an indicator of land use [40] was unclear in the current study. *Tomentella* was associated with the habitat characterized by the lowest rainfall level, but its high sensitivity to seasonal fluctuations [58] makes it difficult to relate its resistance to environmental stresses such as drought.

ErM fungi were also among the most abundant fungal groups significantly associated with habitat disturbances, *Oidiodendron maius* [69,70] and *Cryptosporiopsis* [51], and were respectively associated with high and low disturbances. This observation strengthens the hypothesis that EcM plants can act as reservoirs of ErM fungi [50], but also suggests that certain ErM fungal members such as *Oidiodendron* might play a role for efficient Ericaceae re-colonization of habitats characterized by strong Ericaceae layer degradation such as found in Maâmora.

A wide range of soil characteristics, whether or not related to land use, were also described as strongly affecting soil fungal communities in forest ecosystems [2,17,18]. In the current work, pH, C:N ratio, and available P appeared as the most significant soil parameters correlated to differences in the root-associated fungal community structure. Soil pH is described as the most influential driver of fungal OTU richness and composition [2]. While a narrow soil pH range was observed in the current study for the Moroccan cork oak habitats, a wide range of fungal OTUs associated with high and low acidity were identified. The impact of phosphorus and C:N ratio was shown as more taxa-specific [2]. Some EcM fungi notably able to adopt a saprophytic lifestyle depending on environmental conditions [71] were associated with C and P cycling in the current study. *Tomentella* species were notably associated with a high SOM decomposition rate and low available soil P content. By contrast, the EcM *Cenococcum* appeared strongly associated with high P soils.

The present study sheds new light on the below-ground interactions taking place in the Moroccan cork oak forest, a severely threatened ecosystem. A highly diverse soil fungi community, both in Ascomycota and Basidiomycota, strongly structured by habitat type (related to disturbance levels) and the soil properties has been observed. However, the characterization of robust fungal indicators of environmental conditions remains a critical point depending of the taxonomic level considered. Indeed, high intraspecific variability (OTU level) were observed for certain fungal genera or species, reflecting not only a taxonomic variability but a probable functional variability since their association with environmental conditions differed. This has been pointed out for several EcM fungi (e.g *Tomentella*, *Russula*) [66], and its extent at the whole community level remains a major issue in fungal-based functional ecology [72]. At the biogeographic level, the results suggest a Southern Mediterranean fungal pattern, reinforcing the need to investigate the Southern Mediterranean fungal diversity more extensively. In addition, the strong geographical structure of *Q*. *suber* in the Mediterranean basin [73] may also affect fungal diversity associated with cork oak, probably contributing to the differences between fungal community structures in Morocco, as compared to Sardinia and Corsica. Indeed, intra-specific plant host diversity has been described as a soil fungal diversity driver [74] but remains poorly investigated. The success of future conservation strategies thus depends on joint initiatives on a Mediterranean scale, associating plant population geneticists, botanists, naturalists, microbiologists and pedologists to picture in a broader framework of the tremendous complexity of plant-fungal and fungal-fungal assembly rules in forest ecosystems [16,62,75,76].

## Supporting information

S1 File Data and code snippets used to produce the results presented in this study. (ZIP)

S1 Table Taxonomic affiliation of root-associated fungal OTUs of *Q*. *suber* in the Moroccan cork oak forest (Maâmora, Benslimane, Chefchaoun). (XLSX)

S2 Table Variation in *Q*. *Suber* root-associated fungal community structures among Moroccan cork oak habitats. (DOCX)

S3 Table Fungal indicators of Moroccan cork oak habitats (Maâmora, Benslimane and Chefchaoun). (DOCX)

S4 Table Soil parameters in the Moroccan cork oak forest. (XLSX)

S5 Table Correlation coefficient between soil physical-chemical parameters among Moroccan cork oak habitats. (XLSX)

S6 Table Fungal OTU indicators with respect to soil properties (pH, C:N ratio, available P). (DOCX)

S1 Fig Venn diagram representing shared and unique EcM-related fungal OTUs among habitats (Maâmora, Benslimane, Chefchaoun). (TIFF)
