# Supplementary material for: Habitat- and soil-related drivers of the root-associated fungal community of Quercus suber in the Northern Moroccan forest
Source: PLoS One. 2017 Nov 20;12(11):e0187758. doi: 10.1371/journal.pone.0187758 (PMC5695781; doi:10.1371/journal.pone.0187758)
Supplement: S1 File — (ZIP) [file pone.0187758.s001.zip › S1 File.html]

xml version="1.0" encoding="utf-8"?


Habitat specificity in soil fungal community associated to <i>Quercus suber</i> in Northern Moroccan forests (supplementary file)


# Habitat specificity in soil fungal community associated to *Quercus suber* in Northern Moroccan forests (supplementary file)

## Table of Contents

- 1. Disclaimer
- 2. Metadata
- 3. Data preparation
- 4. Quality encoding checking and Read Pairs assembly
- 5. Demultiplexing and trimming (cutadapt)
- 6. Dereplication (global), clustering, chimera detection
- 7. Length distribution
- 8. Taxonomic assignment with UNITE + INSDC (International Nucleotide Sequence Database Collaboration)
- 9. Improve taxonomic assignments
  - 9.1. Blast against GenBank NR
  - 9.2. Blast against Unite
  - 9.3. Construct a small dataset of plant ITS references (10 first OTUs)
  - 9.4. Extract the next 10 potential assignments to further improve
  - 9.5. 3rd round
  - 9.6. 4th round
  - 9.7. Monitor progress
- 10. Contingency tables (OTUs)
  - 10.1. Length ditribution
  - 10.2. Visualization of best qualities per length
  - 10.3. OTU table (full)
  - 10.4. OTU table filtering (fungi only)
  - 10.5. OTU table filtering (streptophyta only)
  - 10.6. OTU table filtering (non-streptophyta, non-fungi)
- 11. Stampa plots
  - - 11.0.1. All OTUs
    - 11.0.2. Fungi
    - 11.0.3. Streptophyta
    - 11.0.4. Other clades
  - 11.1. Distribution of quality-filtered OTUs
- 12. Statistical analyses
  - 12.1. Rarefaction of Quercus suber data
  - 12.2. Rarefaction of Quercus suber data (Fungi) and taxonomic profiles
  - 12.3. How many raw reads in the Quercus ND samples?
  - 12.4. Quercus suber ITS1 Fungi vs environment parameters
  - 12.5. Quercus suber ITS1 Fungi ternary plots (order level)
  - 12.6. Quercus suber ITS1 Fungi ternary plots (genus level)
  - 12.7. Compare Fungi and Quercus community distance tables
  - 12.8. Compare the distribution of Cenococcum with the distribution of its known parasits
- 13. OTU representatives

## 1 Disclaimer

The purpose of this document is too provide the reader with details on
the bioinformatics methods used to prepare this paper. The code
snippets and shell commands presented here were executed on a Debian
GNU/Linux 8, and might have to be adapted to your particular
system. Use them carefully.

## 2 Metadata

**primers (ITS1FI2-ITS2)**

PRIMER\_F="GAACCWGCGGARGGATCA"
PRIMER\_R="GCATCGATGAAGAACGCAGC"

PhiX spike-in is 20%

**Bioinformatics tools (base-calling and demultiplexing)**

RTA v1.18.54 and MCS 2.5, demultiplexing with bcl2fastq1.8

## 3 Data preparation

```
# lstm
cd ~/Science/projects/Quercus_suber/data/
# uncompress
find . -name "*.fastq.gz" -print0 | xargs -0 gunzip
# compress
find . -name "*.fastq" -print0 | xargs -0 -I {} bzip2 -9k {}
find . -name "*.fastq" -print0 | xargs -0 -I {} xz -9k {}
```

## 4 Quality encoding checking and Read Pairs assembly

```
# lstm
cd ${HOME}/Science/projects/Quercus_suber/data/

VSEARCH="/usr/local/bin/vsearch"
ENCODING=33

for FORWARD in ./ITS1_*/*_R1_001.fastq.bz2 ; do
    "${VSEARCH}" --fastq_mergepairs "${FORWARD}" \
                 --reverse "${FORWARD/_R1/_R2}" \
                 --fastq_ascii "${ENCODING}" \
                 --fastqout "${FORWARD/_R1*/_assembled.fastq}" \
                 --fastq_allowmergestagger \
                 --quiet 2> "${FORWARD/_R1*/_assembled.log}"
done
```

## 5 Demultiplexing and trimming (cutadapt)

Warning, half the reads are reverse-complemented. The plan is to trim
in two steps:

- identify and trim the tag (there could be a lot of false positives
  here, so I have to repeat the extraction for each tag individualy),
- **UPDATE** force the tag match to be full-length (cutadapt's default
  is 3 nucleotide). It is equivalent to an exact search,
- search for the forward primer and trim (false positives and wrongly
  trimmed tags should be filtered out),
- **UPDATE** force the primer match to be 2/3rd of full-length,
- search the reverse-complemented fastq file,
- export to fasta,
- dereplicate and hash with vsearch,
- produce a final fasta file per sample

Refactoring with vsearch 1.10

```
# lstm
cd ${HOME}/Science/projects/Quercus_suber/data/

function demultiplex_dereplicate() {
    # Define variables, temporary files and output files
    INPUT="${1}"
    PRIMER_F="GAACCWGCGGARGGATCA"
    PRIMER_R="GCATCGATGAAGAACGCAGC"
    MIN_LENGTH=32
    MIN_F=$(( ${#PRIMER_F} * 2 / 3 ))
    MIN_R=$(( ${#PRIMER_R} * 2 / 3 ))

    CUTADAPT="cutadapt --discard-untrimmed --minimum-length ${MIN_LENGTH}"
    VSEARCH="/usr/local/bin/vsearch"
    INPUT_REVCOMP=$(mktemp --tmpdir=".")
    TMP_FASTQ=$(mktemp --tmpdir=".")
    TMP_FASTQ2=$(mktemp --tmpdir=".")
    NULL="/dev/null"

    # Reverse complement fastq file
    "${VSEARCH}" --quiet \
                 --fastx_revcomp "${INPUT}" \
                 --fastqout "${INPUT_REVCOMP}"

    tail -n +2 mapping.txt | cut -f 1,2 | \
        while read TAG_NAME TAG_SEQ ; do
            LOG="${TAG_NAME}.log"
            FINAL_FASTA="${TAG_NAME}.fas"
            QUALITY="${TAG_NAME}.qual"

            # Trim tags, forward & reverse primers (search normal and antisens)
            cat "${INPUT}" "${INPUT_REVCOMP}" | \
                ${CUTADAPT} -g "${TAG_SEQ}" -O ${#TAG_SEQ} - 2> "${LOG}" | \
                ${CUTADAPT} -g "${PRIMER_F}" -O "${MIN_F}" - 2>> "${LOG}" | \
                ${CUTADAPT} -a "${PRIMER_R}" -O "${MIN_R}" - 2>> "${LOG}" > "${TMP_FASTQ}"

            # Discard erroneous sequences and add expected error rates
            "${VSEARCH}" \
                --quiet \
                --fastq_filter "${TMP_FASTQ}" \
                --fastq_maxns 0 \
                --relabel_sha1 \
                --eeout \
                --fastqout "${TMP_FASTQ2}" 2>> "${LOG}"

            # Dereplicate at the study level (vsearch)
            "${VSEARCH}" \
                --quiet \
                --derep_fulllength "${TMP_FASTQ2}" \
                --sizeout \
                --fasta_width 0 \
                --output "${FINAL_FASTA}" 2>> "${LOG}"

            # Eliminate expected error values
            sed -i '/^>/ s/;ee=[0-9][0-9.]*//' "${FINAL_FASTA}"

            # Discard quality lines, extract sha1, expected error rates and read length
            sed 'n;n;N;d' "${TMP_FASTQ2}" | \
                awk 'BEGIN {FS = "[;=]"}
                     {if (/^@/) {printf "%s\t%s\t", $1, $3} else {print length($1)}}' | \
                tr -d "@" >> "${QUALITY}"
        done

    # Clean
    rm -f "${INPUT_REVCOMP}" "${TMP_FASTQ}" "${TMP_FASTQ2}"
}

cd ./ITS1_CM/
demultiplex_dereplicate SAM137-169_S3_L001_assembled.fastq
cd ../ITS1_Q_CS/
INPUT=$(mktemp --tmpdir=".")
cat *_assembled.fastq > "${INPUT}"
demultiplex_dereplicate "${INPUT}" && rm -r "${INPUT}"
```

**Produce the final quality file**

```
# lstm
cd ${HOME}/Science/projects/Quercus_suber/data/

export LC_ALL=C
QUALITY_FILE="ITS1_Q_CM_CS.qual"

# Produce the final quality file
cat ./ITS1_*/*.qual | \
    sort -k3,3n -k1,1d -k2,2n | \
    uniq --check-chars=40 > "${QUALITY_FILE}"
```

## 6 Dereplication (global), clustering, chimera detection

Refactoring with vsearch 1.10

```
cd ~/Science/projects/Quercus_suber/data/

VSEARCH="/usr/local/bin/vsearch"
TMP_FASTA=$(mktemp --tmpdir=".")
FINAL_FASTA="ITS1_Q_CM_CS.fas"

# Pool sequences (remove the "ee" values)
cat ./ITS1_*/*.fas > "${TMP_FASTA}"

# Dereplicate (vsearch)
"${VSEARCH}" --quiet \
             --derep_fulllength "${TMP_FASTA}" \
             --sizein \
             --sizeout \
             --fasta_width 0 \
             --output "${FINAL_FASTA}" > /dev/null

rm -f "${TMP_FASTA}"

# Clustering
THREADS=16
TMP_REPRESENTATIVES=$(mktemp --tmpdir=".")
swarm \
    -d 1 -f -t ${THREADS} -z \
    -i ${FINAL_FASTA/.fas/_1f.struct} \
    -s ${FINAL_FASTA/.fas/_1f.stats} \
    -w ${TMP_REPRESENTATIVES} \
    -o ${FINAL_FASTA/.fas/_1f.swarms} < ${FINAL_FASTA}

# Sort representatives
"${VSEARCH}" --fasta_width 0 \
             --sortbysize ${TMP_REPRESENTATIVES} \
             --output ${FINAL_FASTA/.fas/_1f_representatives.fas}
rm ${TMP_REPRESENTATIVES}

# Chimera checking
REPRESENTATIVES=${FINAL_FASTA/.fas/_1f_representatives.fas}
UCHIME=${REPRESENTATIVES/.fas/.uchime}
"${VSEARCH}" --uchime_denovo "${REPRESENTATIVES}" \
             --uchimeout "${UCHIME}"
```

Number of swarms: 178447
Largest swarm: 60304
Max generations: 23

Found 83923 (47.0%) chimeras, 92628 (51.9%) non-chimeras,
and 1896 (1.1%) borderline sequences in 178447 unique sequences.
Taking abundance information into account, this corresponds to
264692 (4.0%) chimeras, 6378290 (95.6%) non-chimeras,
and 29094 (0.4%) borderline sequences in 6672076 total sequences.

So **48.1%** of OTUs are chimeras.

## 7 Length distribution

```
# lstm
cd ~/Science/projects/Quercus_suber/data/

awk '!/^>/ {print length($1)}' ITS1_Q_CM_CS.fas | \
    sort -n | uniq -c | awk '{print $2, $1}' | graph -T X
```

Two main peaks, maybe a plant and a fungi peak?

## 8 Taxonomic assignment with UNITE + INSDC (International Nucleotide Sequence Database Collaboration)

Unite (Unified system for the DNA based fungal species linked to the classification)
INSDC (International Nucleotide Sequence Database Collaboration)

UNITE follows the Index Fungorum classification in nearly all regards.

Unite's repository offers several versions and several flavors. I
should aim for the latest version (v7 [2015-09-18 ven.]). The
different flavors correspond to different clustering levels, expert
annotation, singleton handling and pipeline (QIIME, Mothur, Blast,
etc).

Following Kõljalg et al. (2013), each terminal fungal taxon for which
two or more ITS sequences are available is referred to as a species
hypothesis (SH). One sequence is chosen to represent each SH; these
sequences are called representative sequences (RepS) when chosen
automatically by the computer and reference sequences (RefS) when
those choices are overridden (or confirmed) by users with expert
knowledge of the taxon at hand.

There are four releases of the RepS/RefS set: special files
pre-formatted for QIIME, mothur, and CREST use, and one general FASTA
release for, e.g., local BLAST searches.

**General FASTA release** (download)

This release consists of a single FASTA file: the RepS/RefS of all
SHs, adopting the dynamically use of clustering thresholds whenever
available. The format of the FASTA header is:

>Glomeraceae|AM076560|SH146432.05FU|refs|k\_\_Fungi;p\_\_Glomeromycota;c\_\_Glomeromycetes;o\_\_Glomerales;f\_\_Glomeraceae;g\_\_;s\_\_uncultured\_Glomus

This specifies the hierarchical classification of the sequence:

- k = kingdom
- p = phylum
- c = class
- o = order
- f = family
- g = genus
- s = species

Missing information is indicated as "unidentified" item;
“f\_\_unidentified;” means that no family name for the sequence exists.

The latest version is 7.1. I decide to use the blast release, without
global singletons.

```
# kl
cd ~/Science/data/references/ITS/Unite/

# From https://unite.ut.ee/repository.php
VERSION="22.08.2016"
RELEASE="sh_general_release_${VERSION}.zip"

# Download and clean
[[ -f ${RELEASE} ]] || wget https://unite.ut.ee/sh_files/${RELEASE}
unzip -f ${RELEASE}
INPUT=$(echo *_${VERSION}.fasta)
rm -rf "${RELEASE}" ./developer/

# Prepare fasta file, modify headers and trim primers
DATE=${RELEASE/*_/}
DATE=${DATE/.fasta/}
YEAR=$(cut -d "." -f 3 <<< ${DATE})
MONTH=$(cut -d "." -f 2 <<< ${DATE})
DAY=$(cut -d "." -f 1 <<< ${DATE})
CUTADAPT="cutadapt --discard-untrimmed"
PRIMER_F="GAACCWGCGGARGGATCA"
PRIMER_F_NAME="ITS1FI2"
PRIMER_R="GCATCGATGAAGAACGCAGC"
FINAL_FASTA="unite_${PRIMER_F_NAME}_${YEAR}${MONTH}${DAY}.fasta"
LOG=${FINAL_FASTA/.fasta/.log}

sed 's/|/ /4 ; s/;/|/g' "${INPUT}" | \
    ${CUTADAPT} -g "${PRIMER_F}" - 2> "${LOG}" | \
    ${CUTADAPT} -a "${PRIMER_R}" - 2>> "${LOG}" > "${FINAL_FASTA}"

rm -f "${INPUT}"
```

Only 24% of the references contain the ITS1FI2 primer. Is this expected?

**copy to kl and launch stampa**

```
# lstm
cd ~/Science/data/references/ITS/Unite/
scp -C unite_ITS1FI2_20150801.fasta kl:/scratch/mahe/projects/Quercus_suber/data/references/ITS/

cd ~/Science/projects/Quercus_suber/data/
scp -C ITS1_Q_CM_CS_1f_representatives.fas kl:/scratch/mahe/projects/Quercus_suber/data/

# kl
cd /scratch/mahe/src/
bash stampa.sh ../projects/Quercus_suber/data/ITS1_Q_CM_CS_1f_representatives.fas ITS1FI2
sleep 15m
cd ../projects/Quercus_suber/data/
rm -rf ./stampa_ITS1_Q_CM_CS_1f_representatives/
```

**collect results**

```
# lstm
cd ~/Science/projects/Quercus_suber/data/
scp -C kl:/scratch/mahe/projects/Quercus_suber/data/ITS1_Q_CM_CS_1f_representatives.results .
```

## 9 Improve taxonomic assignments

### 9.1 Blast against GenBank NR

It seems that the GenBank databases available on Elwe were not
compiled with the accession number option.

```
kl
cd /scratch/mahe/src/
FASTA="../projects/Quercus_suber/data/ITS1_Q_CM_CS_1f_representatives.fas"
bsub -q long \
     -n 16 \
     -R "span[hosts=1] rusage[mem=15000]" \
     bash blastn.sh "${FASTA}" megablast
```

### 9.2 Blast against Unite

```
# lstm
cd ~/Science/data/references/ITS/Unite/

INPUT="sh_general_release_dynamic_01.08.2015.fasta"
TITLE="unite_20150801"

makeblastdb \
    -in "${INPUT}" \
    -dbtype "nucl" \
    -input_type "fasta" \
    -logfile "${INPUT%.*}.log" \
    -title "${TITLE}" \
    -out "${TITLE}_gb"
```

**Quick test**

```
# lstm
# cd ~/Science/projects/Quercus_suber/results/ITS1/
# mkdir blast_against_unite
# ln -s ../../data/ITS1_Q_CM_CS_1f_representatives.fas ITS1_Q_CM_CS_1f_representatives.fas
# ln -s ../../../../data/references/ITS/Unite/unite_20150801_gb.nhr unite_20150801_gb.nhr
# ln -s ../../../../data/references/ITS/Unite/unite_20150801_gb.nin unite_20150801_gb.nin
# ln -s ../../../../data/references/ITS/Unite/unite_20150801_gb.nsq unite_20150801_gb.nsq

cd ~/Science/projects/Quercus_suber/results/blast_against_unite/

QUERY="ITS1_Q_CM_CS_1f_representatives.fas"
FLAVOR="megablast"  # FLAVOR = blastn or megablast
PATH_TO_DB="${HOME}/Science/data/references/ITS/Unite"
DATABASE="unite_20150801_gb"
OUTPUT="${QUERY%.*}.table"
NUMBER_OF_HITS=50
THREADS=8
MIN_IDENTITY=70

blastn \
    -query "${QUERY}" \
    -task "${FLAVOR}" \
    -db "${PATH_TO_DB}/${DATABASE}" \
    -out "${OUTPUT}" \
    -num_threads "${THREADS}" \
    -outfmt "7 qseqid sseqid pident length mismatch gapopen qstart qend sstart send evalue bitscore stitle" \
    -max_target_seqs "${NUMBER_OF_HITS}" \
    -perc_identity "${MIN_IDENTITY}"
```

**search for improvable assignments**

Only try for the top 1,000 OTUs.

```
# lstm
cd ~/Science/projects/Quercus_suber/data/
export LC_ALL=C

UNITE="${HOME}/Science/data/references/ITS/Unite"
ORIGINAL="sh_general_release_dynamic_01.08.2015.fasta"
MODIFIED="unite_ITS1FI2_20150801.fasta"
BLAST_TABLE="../results/blast_against_unite/ITS1_Q_CM_CS_1f_representatives.table"
OTU_TABLE="ITS1_Q_CM_CS.OTU.table"

awk 'BEGIN {FS = OFS = "\t"} {print $1, $2, $(NF-2), $(NF-1), $NF}' "${OTU_TABLE}" | \
head -n 1000 | \
while read otu hash_string identity taxonomy references ; do
    hits=$(grep "^${hash_string}" "${BLAST_TABLE}")
    if [[ "${hits}" ]] ; then
        blast_identity=$(echo ${hits} | head -n 1 | cut -d " " -f 3)
        accession=$(echo ${hits} | head -n 1 | cut -d " " -f 2 | cut -d "|" -f 2)
        status=$(bc <<< "${blast_identity}>${identity}")
        if [[ ${status} -eq 1 ]] ; then
            # is it already in our database?
            already=$(grep -m 1 "${accession}" "${UNITE}/${MODIFIED}")
            if [[ -z "${already}" ]] ; then
                start=$(echo ${hits} | head -n 1 | cut -d " " -f 9)
                end=$(echo ${hits} | head -n 1 | cut -d " " -f 10)
                length=$(( $end - $start ))
                if [[ ${length} -gt 100 ]] ; then
                    grep -m 1 "${accession}" "${UNITE}/${ORIGINAL}"
                    grep -m 1 -A 1 "${accession}" "${UNITE}/${ORIGINAL}" | tail -n 1 | cut -c ${start}-${end}
                fi
            fi
        fi
    fi
done | sed 's/|/ /4 ; s/;/|/g' > ITS_new_fungi_references.fas
```

**Add that to the my unite database and relaunch stampa**

```
# lstm
cd ~/Science/projects/Quercus_suber/data/

FOLDER="${HOME}/Science/data/references/ITS/Unite"
UNITE="unite_ITS1FI2_20150801.fasta"
MODIFIED="${UNITE/.fasta/_modified.fasta}"
NEW_REFERENCES="ITS_new_fungi_references.fas"
PLANT_REFERENCES="ITS1_streptophyta_references.fasta"

# Merge with the reference fasta file
cat "${FOLDER}/${UNITE}" > "${FOLDER}/${MODIFIED}"
paste - - < "${NEW_REFERENCES}" | \
    awk 'BEGIN {FS = "\t"}
         {if (length($2) > length(a[$1])) {a[$1] = $2}
         } END {for (i in a) {print i"\n"a[i]}}' >> "${FOLDER}/${MODIFIED}"
# and plant reference sequences
cat "${FOLDER}/${PLANT_REFERENCES}" >> "${FOLDER}/${MODIFIED}"

# Copy to kl
cd "${FOLDER}"
scp "${MODIFIED}" kl:/scratch/mahe/projects/Quercus_suber/data/references/ITS/${UNITE}

# kl
cd /scratch/mahe/src/
bash stampa.sh ../projects/Quercus_suber/data/ITS1_Q_CM_CS_1f_representatives.fas ITS1FI2
sleep 60m
cd ../projects/Quercus_suber/data/
rm -rf ./stampa_ITS1_Q_CM_CS_1f_representatives/
```

**transfer the results**

```
# lstm
cd ~/Science/projects/Quercus_suber/data/
scp kl:/scratch/mahe/projects/Quercus_suber/data/ITS1_Q_CM_CS_1f_representatives.results .
```

### 9.3 Construct a small dataset of plant ITS references (10 first OTUs)

```
>DQ092945.1 Eukaryota|Viridiplantae|Streptophyta|Embryophyta|Tracheophyta|Spermatophyta|Magnoliophyta|eudicotyledons|Gunneridae|Pentapetalae|rosids|malvids|Malvales|Cistaceae|Cistus|salviifolius|6PV03
CGATTCCTGCCTAGCAGACAGACCCGCGAACTGGTTATAAACAACACGTCGCGGCCGTCGTCGGGCGAGAGCCCCGCGACGGCCCGGCGCAACCGCCGCGTGAGCGCGGCATTCCCGTCGTCCCCTGCGGGATCGGCGGTGCGTCGTGCTTTCCGCGGCACACAAACGAACCCCGGCGTGGATAACGCCAAGGAAAACAAAAGGGAAGTCGCGTCCCCGCGCAACGCGGGGCTCGCGACTCCTGTCGTACTTACAATAACGACTCTCGGCAACGGATATCTCGGCTCTC
>DQ092966.1 Eukaryota|Viridiplantae|Streptophyta|Embryophyta|Tracheophyta|Spermatophyta|Magnoliophyta|eudicotyledons|Gunneridae|Pentapetalae|rosids|malvids|Malvales|Cistaceae|Cistus|monspeliensis|35BGA04
CGATTCCTGCCTAGCAGACAGACCCGCGAACTGGTTATACACAACGCGTGGCGGCCGTCGTCGGGCGAGAGCCCCGCAGCGGCCCAACGCACCCGCCGCGTGAGCGCGGCCTTCCCGTCGTCCCCTGCGGGATCGGCGGTGCGTCGTGCTTTCCGCGGCACACAAACGAACCCCGGCGTGGATAACGCCAAGGAAAACAAAAGGGAAGCCGCGTCCCCGCGCAACGCGGGGCTCGCGACTCCTGTCGTACTTACAATAACGACTCTCGGCAACGGATATCTCGGCTCTC
>FM243908.1 Eukaryota|Viridiplantae|Streptophyta|Embryophyta|Tracheophyta|Spermatophyta|Magnoliophyta|eudicotyledons|Gunneridae|Pentapetalae|rosids|fabids|Fagales|Fagaceae|Quercus|suber|su3118
TATCATTTAGAGGAAGGAGAAGTCGTAACAAGGTTTCCGTAGGTGAACCTGCGGAAGGATCATTGTCGAAACCTGCACAGCAGAACGACCCGCGAATTGGTGACAACCGACGGAGGGCGGGGGGCGCTCGTCGCCCCCTCGTCCCCCACGCAGGCGGGGACCTTGCGTCTCTTGCCTGCAAACCGAACCCCGGCGCGGAACGCGCCAAGGAAATCGAACCAAAAGAGCCGCGCCGGAGGCCCCGGACACGGTGCGCC
>FM243911.1 Eukaryota|Viridiplantae|Streptophyta|Embryophyta|Tracheophyta|Spermatophyta|Magnoliophyta|eudicotyledons|Gunneridae|Pentapetalae|rosids|fabids|Fagales|Fagaceae|Quercus|suber|su3443
TATCATTTAGAGGAAGGAGAAGTCGTAACAAGGTTTCCGTAGGTGAACCTGCGGAAGGATCATTGTCGAAACCTGCACAGCAGAACGACCCGCGAATTGGTGACAACCGACGGAGGGCGGGGGGCGCTCGTCGCCCCCTCGCCCCCCACGCAGGCGGGGACCTCGCGTCTCTTGCCTGCAAACCGAACCCCGGCGCGGAACGCGCCAAGGAAATCGAACCAAAAGAGCCGCGCCGGAGGCCCCGGACACGGTGCGCC
>FM243914.1 Eukaryota|Viridiplantae|Streptophyta|Embryophyta|Tracheophyta|Spermatophyta|Magnoliophyta|eudicotyledons|Gunneridae|Pentapetalae|rosids|fabids|Fagales|Fagaceae|Quercus|suber|su3611
TATCATTTAGAGGAAGGAGAAGTCGTAACAAGGTTTCCGTAGGTGAACCTGCGGAAGGATCATTGTCGAAACCTGCACAGCAGAACGACCCGCGAATTGGTGACAACCGACGGGGGGCGCTCGTCGCCCCCTCGCCCCCCACGCAGGCGGGGACCTCGCGTCTCTTGCCTGCAAACCGAACCCCGGCGCGGAACGCGCCAAGGAAATCGAACCAAGAGAGCCGCGCCGGAGGCCCCGGACACGGTGCGCC
>EF193082.1 Eukaryota|Viridiplantae|Streptophyta|Embryophyta|Tracheophyta|Spermatophyta|Magnoliophyta|eudicotyledons|Gunneridae|Pentapetalae|rosids|malvids|Sapindales|Anacardiaceae|Pistacia|lentiscus|Golan_1.1009
TTGTCGAAACCTGCCAAGCAGAACGACCCGTGAACCTGTCATAACATCGGGGGCCCATGGGCTTCGTGCCTGTGTGCCTCCACCCGTGCTTCGTCGGGTGTTGGTCGTATGTTTGCGCATGCGACTGCCTCGTCGTTGCGCATTAACGAACCCCGGCGCGAATTGCGTCAAGGAAATCTTAACGAGAGAGCTCGATCCTGTTGCCCCGGACACGGTGCGCGTACGGGATGTGTGGCCTTCTTTCATAATCAATAACGACTCTCGGCAACGGATATCTCGGCTCTC
>HQ858911.1 Eukaryota|Viridiplantae|Streptophyta|Embryophyta|Tracheophyta|Spermatophyta|Magnoliophyta|eudicotyledons|Gunneridae|Pentapetalae|asterids|Ericales|Ericaceae|Ericoideae|Ericeae|Erica|arborea|SANBI_S890
TTGTCAAAACCTGACATCTAGAAAACTCGTGAACTTGTTCATAAAGAATGGGGAATGCATCGGTTTGGCCCAGTGCCTCTCCTTTTGCTTTCCCCTCGCGAGCAGACATGCCTGGAGCTTTCGAGCGACGTGTTTGTCTGCTTGTCGAACAACGAACCCCGGCGCAAATCGTGCCAAGGATAATGGAATGAGTTTGTGCATGCATGTCCCTGCCCGTCTATGGGTGGCGTTGGCTTGCACATCTTTTGTATAACTAAACGACTCTCGGCAACGGATATCTCGGCTCTT
>FM243889.1 Eukaryota|Viridiplantae|Streptophyta|Embryophyta|Tracheophyta|Spermatophyta|Magnoliophyta|eudicotyledons|Gunneridae|Pentapetalae|rosids|fabids|Fagales|Fagaceae|Quercus|suber|su1465
TTGTCGAAACCTGCACAGCAGAACGACCCGCGAATTGGTGACAACCGACGGGGGGCGGGGGGCGCTCGTCGCCCCCTCGCCCCTCACGCAGGCGGGGACCTCGCGTCTCTTGCCTGCAAACCGAACCCCGGCGCGGAACGCGTCAAGGAAATCGAACCAAGAGAGCCGCGCCGGAGGCCCCGGACACGGTGCGCCCCCGGCGTCGGCGTCTTACGAATTATTTAAAACGACTCTCGGCAACGGATATCTAGGCTCTC
```

### 9.4 Extract the next 10 potential assignments to further improve

```
kl
cd /scratch/mahe/projects/Quercus_suber/data/

RESULTS="ITS1_Q_CM_CS_1f_representatives.results"
TABLE="ITS1_Q_CM_CS_1f_representatives.table"

awk '$3 < 95.0' "${RESULTS}" | \
    head -n 10 | \
    while read SEED MASS ID TAXO REF ; do
        echo "${SEED}" "${MASS}" "${ID}" "${TAXO}" "${REF}"
        grep -m 51 "${SEED}" "${TABLE}"
        echo
    done > tmp.blast
```

discard fungi

```
>FM243907.1 Eukaryota|Viridiplantae|Streptophyta|Embryophyta|Tracheophyta|Spermatophyta|Magnoliophyta|eudicotyledons|Gunneridae|Pentapetalae|rosids|fabids|Fagales|Fagaceae|Quercus|suber|su3109
TTGTCGAAACCTGCACAGCAGAACAACCCGCGAATTGGTGATAACCGACGGGGGGCGCTCGTCACCCCCTCGCCCCCCACGCAGGCGGGGACCTCGCGTCTCTTGCCTGCAAACCGAACCCCGGCGCGGAACGCGCCAAGGAAATCGAACCAAGAGAGCCGCGCTGGAGGCCCCGGACACGGTGCGCCCCCGGTGTCAGCGTCTTACGAATTATTTAAAACGACTCTCGGCAACGGATATCTAGGCTCTC
>FM243877.1 Eukaryota|Viridiplantae|Streptophyta|Embryophyta|Tracheophyta|Spermatophyta|Magnoliophyta|eudicotyledons|Gunneridae|Pentapetalae|rosids|fabids|Fagales|Fagaceae|Quercus|suber|su0306
TTGTCGAAACCTGCACAGCAGAACAACCCGCGAATTGGTGATAACCGACGGGGGGCGCTCGTCACCCCCTCGCCCCCCACGCAGGCGGGGACCTCGCGTCTCTTGCCTGCAAACCGAACCCCGGCGCGGAACGCGCCAAGGAAATCGAACCAAGAGAGCCGCGCCGGAGGCCCCGGACACGGTGCGCCCCCGGCGTCGGCGTCTTGCAAATTATTTAAAACGACTCTCGGCAACGGATATCTAGGCTCTC
>FM243912.1 Eukaryota|Viridiplantae|Streptophyta|Embryophyta|Tracheophyta|Spermatophyta|Magnoliophyta|eudicotyledons|Gunneridae|Pentapetalae|rosids|fabids|Fagales|Fagaceae|Quercus|suber|su3449
TTGTCGAAACCTGCACAGCAGAACGACCCGCGAATTGGTGATAACCGACGGGGGGCGCTCGTTGCCCCCTCGCCCCCCACGCAGGCGGGACCTCGCGTCTCTTGCCTGCAAACCGAACCCCGGCGCGGAACGCGCCAAGGAAATCGAACCAAGAAAGCCGCGCTGGAGGCCCCGGACACGGTGCGCCCCCGGCGTCGGCGTCTTACGAATTATTTAAAACGACTCTCGGCAACGGATATCTAGGCTCTC
>AY598644.2 Eukaryota|Stramenopiles|Oomycetes|Pythiales|Pythiaceae|Pythium|paroecandrum 
TTACCACACCTAAAAAACTTTCCACGTGAACTGTCGTTATTTGTTGTGTGTGTGCGCGTTGCTGGCGTGCGTTTGCTTACGCTTCGGTGTTTGCGAGTGCGTGCTGTCGGTGCGCGGACTGAACGAAGGTCGTGTGTTTGCTGTGTGCCTGCTGCACCGCTGACTTTGCATTCATTTGCATGGTCTTGGCGGAGCGGCGGGTGCTGTGCGTGCGCGGCTGACTTATCTTTTTCAAACCCCATACCTAAATGACTGATTATACTGTGAGAACGAAAGTTCTTGCTTTAAACTAGATAACAACTTTCAGCAGTGGATGTCTAGGCTCGC
```

### 9.5 3rd round

```
>AF091952.1 Eukaryota|Viridiplantae|Streptophyta|Embryophyta|Tracheophyta|Spermatophyta|Magnoliophyta|eudicotyledons|Gunneridae|Pentapetalae|asterids|Ericales|Ericaceae|Arbutoideae|Arbutus|unedo
TCGAATGCCAAGCAGAAAGACCCGCGAACGTGTTACGTAATACACCTCCGGGAACAATTGAGCGGCCGACCCAGTTGTCGCCTTCCATTTGTTCCTCCTCGAGCGGGTGCACGGTCCTTCGGGAAACGTGTTCATTCRCTCGTSAAATAACGAAACCCGGCGCAAACCGCGCCAAGGAAACTTGGAAAAACAGATGCACGTCTCCCACCCATTCGTGGGCTGTGTTTGGCGCTTGCATATTTCGCAAAACTGAACGACTCTCGGCAACGGATATCTCGGCTCTT
>AM234149.1 Eukaryota|Viridiplantae|Streptophyta|Embryophyta|Tracheophyta|Spermatophyta|Magnoliophyta|eudicotyledons|Gunneridae|Pentapetalae|rosids|malvids|Myrtales|Myrtaceae|Myrtus|communis
TTGTCGAATCCTGCAAAGCAGAACGACCAGAGAACTCGTAACGAACTCAATGGGGGCGATGGGTTCTCTCCCGTCGTCCCTCGACGCTTGGACAGCGCGGGCGCCTAGAGCGCTCGGGCTTTCTGGGCGGCACAACGAACCCCGGCGCGGAATGCGCCAAGGAACTTGAACAAGAGAGCGATGCTCCCATCACCCCAGACATGGTGCGTGCGTGGGATGCCATGCAATCTCCTATTACTCATAACGACTCTCGGCAACGGATATCTCGGCTCTC
>DQ312004.1 Eukaryota|Viridiplantae|Streptophyta|Embryophyta|Tracheophyta|Spermatophyta|Magnoliophyta|eudicotyledons|Gunneridae|Pentapetalae|rosids|fabids|Fabales|Fabaceae|Papilionoideae|Trifolieae|Trifolium|arvense
TTGTCGATGCCTTACATGCAGACCAACACGTGAATTAGTTTGAACACATAGGGTTGGTTTGAGGTGTTCGACACCTCGGCTTGCCCATGGTTCGGAGGATGACGACACGCGCGTCGTCCTTTGTTCCGAAACACAAACCCCGACGCTGAATGCGTCAAGGAATATAAAATTTGCTCTGAGCACACCTGCATGCCACCGGAGACGGTTTTCGTGCGGGTTGTGTTCTGACACATAATATAGAATGACTCTCGGCAACGGATATCTAGGCTCTT
>AF218535.1 Eukaryota|Viridiplantae|Streptophyta|Embryophyta|Tracheophyta|Spermatophyta|Magnoliophyta|eudicotyledons|Gunneridae|Pentapetalae|rosids|fabids|Fabales|Fabaceae|Papilionoideae|Loteae|Scorpiurus|sulcatus
TCGCATCCTACAAACAAACAAACCCGCGAACTTGTTTCATTACTCGGGTAGGGTTGAGGTGAACTTCACCTCAACAATACTCAGTTAGGAGGAACCCACTCTGTGGGTCTCCTCGTGACAAAAAACAAACCCCGGCGCTTAATGCGTCAAGGAATTGAAATTGTTCAGTGCACTCTTGTTGGCATCGGAGACGGTGCCGCGCAGGTTGTGCCATGACACATTTTACACAAAATGACTCTCGGCAACGGATATCTCGGCTCTC
>AF218536.1 Eukaryota|Viridiplantae|Streptophyta|Embryophyta|Tracheophyta|Spermatophyta|Magnoliophyta|eudicotyledons|Gunneridae|Pentapetalae|rosids|fabids|Fabales|Fabaceae|Papilionoideae|Loteae|Scorpiurus|vermiculatus
TCGCATCCTACAAACAAACAAACCCGCGAACTTGTTTCATTACTCGGGTAGGGTTGAGGTGAACTTCACCTCAACAATACTCAGTTAGGAGGAACCCACTCTGTGGGTCTCCTCGTGACAAAAAACAAACCCCGGCGCTTAATGCGTCAAGGAATTGAAATTGTTCAGTGCACTCTTGTTGGCATCGGAGACGGTGCCGCGCAGGTTGTGCCATGACACATTTTACACAAAATGACTCTCGGCAACGGATATCTCGGCTCTC
>KF713216.1 Eukaryota|Viridiplantae|Streptophyta|Embryophyta|Tracheophyta|Spermatophyta|Magnoliophyta|Liliopsida|Poales|Poaceae|BEP_clade|Pooideae|Poeae|Dactylidinae|Dactylis|glomerata
TTGTCGTGACCCTGACCAAAATAGACCGCGCACCCGTAATCCTGCCCGCCGGGCGGCGCACCGTTTGTCGCCCGGCCAAAAGTCCTCGAGAACCTCGGAAATGGCTCTCGGGGTAAAAGAACCCACGGCGCCGAAGGCGTCAAGGAACACTGTGCGTAACTTGGGGACGCGGCTGGCTCGCTGGCCGCCCCTCGTGTTGCAATGCTATATAATCCACACGACTCTCGGCAACGGATATCTCGGCTCTC
>AF538363.1 Eukaryota|Viridiplantae|Streptophyta|Embryophyta|Tracheophyta|Spermatophyta|Magnoliophyta|Liliopsida|Poales|Poaceae|BEP_clade|Pooideae|Poeae|Loliinae|Festuca|caerulescens
TCGTGATCCTGACCAAAACAGACCGCGCACGAGTCATCTAGCCCGCCGGGCGGCGGCACCGTTCGTCGCTCGGCCAAAGTCCTCGACAACCTCCTCTCTTCGGAGTGGGGGCTCGGGGTAAAAAGAACCCACGGCGCCGAAGGCGTCAAGGAACACTGTGCCTAACCCGGGGACGCGGCCGGCTTGCTGGCCGCTCCCCGTGTTGCAATGCTATTTAATCCACACGACTCTCGGCAACGGATATCTCGGCTCTC
```

### 9.6 4th round

We still have some abundant and badly assigned OTUs. I'll try a last
round of blast to improve that, using the blast results from *Blast
against GenBank NR (GenBank v206)*. I select the top 20 badly assigned
OTUs.

```
# lstm
cd ~/Science/projects/Quercus_suber/data/
awk '$3 < 85.0' ITS1_Q_CM_CS_1f_representatives.results | head -n 20 | less -S
```

| ? | OTU | Abundance | Identity |  |  |  |  |  |  |  |  |  |  |  |  |  |
| --- | --- | --- | --- | --- | --- | --- | --- | --- | --- | --- | --- | --- | --- | --- | --- | --- |
| no | 512b41a9c13c9b8a13e7abfff5c585ba55a6f0ba | 29170 | 82.5 | k\_\_Fungi | p\_\_Basidiomycota | c\_\_Agaricomycetes | o\_\_Agaricales | f\_\_Inocybaceae | g\_\_Inocybe | s\_\_Inocybe\_subporospora | Inocybe\_subporospora | JF908245 | SH185 |  |  |  |
| no | e563755eacd23a803689fe970c24437851789723 | 17446 | 73.4 | k\_\_Fungi | p\_\_Ascomycota | c\_\_Dothideomycetes | o\_\_Pleosporales | f\_\_Massarinaceae | g\_\_Saccharicola | s\_\_Saccharicola\_sp\_CBMAI\_1030 | Saccharicola\_sp\_CBM |  |  |  |  |  |
| EF437225 | 15a2646ab49b3d5c67972c0ccfcc1631593ddb0d | 15624 | 63.4 | Eukaryota | Viridiplantae | Streptophyta | Embryophyta | Tracheophyta | Spermatophyta | Magnoliophyta | eudicotyledons | Gunneridae | Pentapetalae | rosids | malvids | Myr |
| no | 7482065af35e243d5371fcc162ca74bf7bd53ac7 | 11836 | 82.6 | k\_\_Fungi | p\_\_Ascomycota | c\_\_Dothideomycetes | o\_\_Capnodiales | f\_\_Mycosphaerellaceae | \* | \* | Passalora\_sp\_CPC\_11147 | AY752162 | SH206849.07FU | reps,Mycovell |  |  |
| EF185410 | 71f9a6b711fd7feb4ffbe88e8614badd977ee005 | 11710 | 69.2 | Eukaryota | Viridiplantae | Streptophyta | Embryophyta | Tracheophyta | Spermatophyta | Magnoliophyta | eudicotyledons | Gunneridae | Pentapetalae | rosids | fabids | Faga |
| AJ633434 | 1db2621bdad728a22b21843ea23499f32088f52e | 6909 | 65.4 | Eukaryota | Viridiplantae | Streptophyta | Embryophyta | Tracheophyta | Spermatophyta | Magnoliophyta | eudicotyledons | Gunneridae | Pentapetalae | rosids | malvids | Sap |
| EF436990 | 0a212d97af97f7d708620685603f10f6951e133a | 6176 | 63.9 | Eukaryota | Viridiplantae | Streptophyta | Embryophyta | Tracheophyta | Spermatophyta | Magnoliophyta | eudicotyledons | Gunneridae | Pentapetalae | rosids | fabids | Faga |
| KF305721 | c1020d9e889dcd06781bab050e802b543faed952 | 5913 | 66.9 | k\_\_Fungi | p\_\_Ascomycota | c\_\_Pezizomycetes | o\_\_Pezizales | f\_\_Sarcosomataceae | g\_\_Pseudoplectania | s\_\_Pseudoplectania\_ericae | Pseudoplectania\_ericae | JX66 |  |  |  |  |
| fragment of Cistus | de364aed425981ef76390f2250d2972cc6e8664d | 5598 | 0.0 | No\_hit | No\_hit |  |  |  |  |  |  |  |  |  |  |  |
| -- | 6bdbc63889e2bd388a5b1d38a5f44ca3c7d730bf | 3726 | 52.9 | Eukaryota | Viridiplantae | Streptophyta | Embryophyta | Tracheophyta | Spermatophyta | Magnoliophyta | eudicotyledons | Gunneridae | Pentapetalae | rosids | fabids | Faba |
| -- | e6963e8cdcf963e5a1c12fa705e9e339b7068592 | 2990 | 73.1 | k\_\_Fungi | p\_\_Ascomycota | c\_\_Dothideomycetes | o\_\_Pleosporales | f\_\_Massarinaceae | g\_\_Saccharicola | s\_\_Saccharicola\_sp\_CBMAI\_1030 | Saccharicola\_sp\_CBM |  |  |  |  |  |
| -- | ca69e78c8514c37135a8c9267a49b5e971464918 | 2841 | 57.1 | k\_\_Fungi | p\_\_Basidiomycota | c\_\_Agaricomycetes | o\_\_Auriculariales | f\_\_unidentified | g\_\_unidentified | s\_\_Auriculariales\_sp | Auriculariales\_sp | JX998755 |  |  |  |  |
| AY641518 | d02220dfa7285001eec48701386101e29d3c922b | 2800 | 82.1 | Eukaryota | Viridiplantae | Streptophyta | Embryophyta | Tracheophyta | Spermatophyta | Magnoliophyta | eudicotyledons | Gunneridae | Pentapetalae | rosids | malvids | Sap |
| -- | 4ba3370ed7f2b43cf6343c37e9d5eb0306809ac0 | 2792 | 74.6 | k\_\_Fungi | p\_\_Ascomycota | c\_\_Dothideomycetes | o\_\_Pleosporales | f\_\_Massarinaceae | g\_\_Saccharicola | s\_\_Saccharicola\_sp\_CBMAI\_1030 | Saccharicola\_sp\_CBM |  |  |  |  |  |
| -- | 01ba9d1bedbfc9a5fe3f13d97d25d8cce51ce7d1 | 2463 | 77.4 | k\_\_Fungi | p\_\_Basidiomycota | c\_\_Agaricomycetes | o\_\_Agaricales | f\_\_Inocybaceae | g\_\_unidentified | s\_\_Inocybaceae\_sp | Inocybaceae\_sp | JN197727 | SH218204.07 |  |  |  |
| -- | 4daacf9b1b33521796bc59277947599363f0c2bc | 2403 | 56.7 | k\_\_Fungi | p\_\_Basidiomycota | c\_\_Agaricomycetes | o\_\_Auriculariales | f\_\_unidentified | g\_\_unidentified | s\_\_Auriculariales\_sp | Auriculariales\_sp | JX998755 |  |  |  |  |
| -- | f5d3f630d9e8c90ede5e8ccc97dff587352fa3e0 | 2346 | 65.9 | Eukaryota | Viridiplantae | Streptophyta | Embryophyta | Tracheophyta | Spermatophyta | Magnoliophyta | eudicotyledons | Gunneridae | Pentapetalae | rosids | fabids | Faga |
| -- | 237601386965676584a984e9cfeabd71aacd7085 | 2121 | 69.5 | Eukaryota | Viridiplantae | Streptophyta | Embryophyta | Tracheophyta | Spermatophyta | Magnoliophyta | eudicotyledons | Gunneridae | Pentapetalae | rosids | malvids | Mal |
| uncultured | a84d3d4f5c11a4b90d5a6fd387c5e8c727639852 | 2075 | 84.9 | k\_\_Fungi | p\_\_Basidiomycota | c\_\_Agaricomycetes | o\_\_Agaricales | f\_\_Inocybaceae | g\_\_Inocybe | s\_\_Inocybe\_sp | Inocybe\_sp | UDB005786 | SH208567.07FU | refs |  |  |
| uncultured | 693afe7ddf5c9e12ddba0e0ac75315a38657a6fb | 2068 | 82.4 | k\_\_Fungi | p\_\_Basidiomycota | c\_\_Agaricomycetes | o\_\_Agaricales | f\_\_Mycenaceae | g\_\_Mycena | s\_\_Mycena\_speirea | Mycena\_speirea | UDB016257 | SH178372.07FU | refs |  |  |

```
>EF437225.1 Eukaryota|Viridiplantae|Streptophyta|Embryophyta|Tracheophyta|Spermatophyta|Magnoliophyta|eudicotyledons|Gunneridae|Pentapetalae|asterids|lamiids|Lamiales|Lamiaceae|Nepetoideae|Lavanduleae|Lavandula|angustifolia
TTGTCGAAACCTGCAAGGCAGACCGCGAACACGTGTTTAACTCCGCGGGACCGTTGTGCGGCGCGAGCCCCACGCCGATCCCGCTCCCGTCGTTGCACGCGCACCCGTGCGCCGCAGCGTTCGGGCTTAACTCACCCCGGCGCGGAATGCGCCAAGGAAAACAAAATGAAGCGTCGGCCCTCCGCATCCCGTTCGCGGGGCRTGCGGGGGCAGCGGGCGTCTATCGAATGTCATAACGACTCTCGGCAACGGATATCTCGGCTCT
>EF185410.1 Eukaryota|Viridiplantae|Streptophyta|Embryophyta|Tracheophyta|Spermatophyta|Magnoliophyta|eudicotyledons|Gunneridae|Pentapetalae|rosids|malvids|Geraniales|Geraniaceae|Erodium|touchyanum
TCGAAACCTGCACAGCAGAACGACCCGCGAACCCGTTATCCAACCCCGGGGATCGGGGGGCAACCTCCGAGCCCCGACGCCGTGCGGGGAGGGCGAGAGTCCGAACCGCTCGGCGAACAACGTACCCCGGCGCGGCATGCGCCAAGGAACCGAAACGAAGCGATGTGCGCCGCCCGGCCCGTCCGCGGGACGTCGGCGGCAGCACGGTCTTCCACTGTATACTAAACGACTCTCGGCAACGGATATCTCGGCTCTC
>AJ633434.1 Eukaryota|Viridiplantae|Streptophyta|Embryophyta|Tracheophyta|Spermatophyta|Magnoliophyta|eudicotyledons|Gunneridae|Pentapetalae|asterids|campanulids|Asterales|Asteraceae|Cichorioideae|Cichorieae|Cichoriinae|Tolpis|barbata
TCGAACCCTGCAAAGCAGAACGACCCGTGAACATGTACCAACAACCGGGAGCCGGAGAGATGGGCCTCGGTCCCTTTCTTCGGCGCCCTGCCGGCGTGTGTTTTTGGTCTCCCAATCGGGGCGCCATGGATATCATGCCGGCACCTTAACAAACCCCGGCACGGAATGTGCCAAGGAAAACAATAAATGAGAAGGATGCGTCCAGCATCGCCCCGTTCGCGGTGTGCGTGCTGGTCGTGGCCTCCTTGGAATCACAAACGACTCTCGGCAACGGATATCTCGGCTCAC
>EF436990.1 Eukaryota|Viridiplantae|Streptophyta|Embryophyta|Tracheophyta|Spermatophyta|Magnoliophyta|eudicotyledons|Gunneridae|Pentapetalae|asterids|Ericales|Primulaceae|Anagallis|arvensis
TCGAAACCTGCCAAGCAGAACGACCCGTGAACTTGTTTCTCATGCGGGGATGCGCCGTCAGGGTCCCCGATCCTGCGGTGCCATCCTCCGTCGGGGGCGGGAGCTCTGCTCACGTCCCCGGCCAACTAACGAACCCCGGCGCAAACCGCGCCAAGGATTATCTAACAAAGTGATCGAGCCGTCGCTCCCGTTCTCGGGCTGCGATCGGCGAGAGAATCTTGCATATAACAAAACGACTCTCGGCAACGGATATCTAGGCTCTC
>Pseudoplectania_ericae|KF305721|SH206578.07FU|reps k__Fungi|p__Ascomycota|c__Pezizomycetes|o__Pezizales|f__Sarcosomataceae|g__Pseudoplectania|s__Pseudoplectania_ericae
TTAATGAACTCAATTCAGTACCTTGGAGAACTGTTAAGGCCTAGTGCCGTTAAAGACGGTCCCGTGGTGGTGAATTTCATTACCATCCCTTCTGTGCACATATCTCATGTTGCTTCCGTGCGTCTGCAGTCCATCTGTATGGACATGCCTGAAAAGCCTTGGTGGTTCCGAGGGCGCACGGGGACGAAAAACTGTACTCTGGTTTCTGTATTTCATCGTCTGAGTGGTAACCACATTGTAACCAATCAAAACTTTCAACAACGGATCTCTTGGTTCTC
>AY641518.1 Eukaryota|Viridiplantae|Streptophyta|Embryophyta|Tracheophyta|Spermatophyta|Magnoliophyta|eudicotyledons|Gunneridae|Pentapetalae|rosids|malvids|Sapindales|Anacardiaceae|Searsia|quartiniana
ATTGTCGAAACCTGCCCAGCAGAACGACCCGCGAACCTGTCTTAAACACCGGGGGCCTGCGGGCCTCGTGCCCGTGCGCTCCCGCCCGCGCCGCGAAGCGAGTCTGCCGCACGCGTCCGCGTGCGTCCTTCCCCTCGCGGCGCGTTAACGAACCCCGGCGCGAATCGCGCCAAGGAAATCTCAACGAGAGAGCCCGCTCCCGTCGCCCCGGACACGGTGCGCGCGCGGGATGCGTGGCCTTCTTTCAATATCTATAACGACTCTCGGCAACGGATATCTCGGCTCTC
>DQ092929.1 Eukaryota|Viridiplantae|Streptophyta|Embryophyta|Tracheophyta|Spermatophyta|Magnoliophyta|eudicotyledons|Gunneridae|Pentapetalae|rosids|malvids|Malvales|Cistaceae|Tuberaria|guttata
CGATTCCTGCCTAGCAGACAGACCCGCGAACTGGTTATACAACACAATCGCCGCGTGAGCGTCGCAGCCTCTTGTTTATAGCAGGGGGCTCGGCGCTTTCCGCGGCACTCAAACGAACCCCGGCGTGGCATACGCCAAGGAAAACAAATTGGAGGCCGCGTCCCCGCGCYTWGCGGGGCTCGCGACTCCTGTCGTATATACAATAACGACTCTCGGCAACGGATATCTCGGCTCTC
```

**Add manually to the my unite database and relaunch stampa**

```
# kl
cd ~/Science/Databases/ITS/UNITE/

UNITE="unite_ITS1FI2_20160822.fasta"
MODIFIED="${UNITE/.fasta/_modified.fasta}"
NEW_REFERENCES="ITS_new_fungi_references.fas"
PLANT_REFERENCES="ITS1_streptophyta_references.fasta"

# Merge
cat "${UNITE}" "${NEW_REFERENCES}" "${PLANT_REFERENCES}" > "${MODIFIED}"

# Copy to kl
scp "${MODIFIED}" kl:/scratch/mahe/projects/Quercus_suber/data/references/ITS/${UNITE}
```

**transfer the results**

```
# lstm
cd ~/Science/projects/Quercus_suber/data/
scp kl:/scratch/mahe/projects/Quercus_suber/data/ITS1_Q_CM_CS_1f_representatives.results .
```

### 9.7 Monitor progress

```
# lstm
cd ~/Science/projects/Quercus_suber/data/
awk '{t += $2 ; if ($3 >= 95.0) s += $2} END {print s, t, 100*s/t}' ITS1_Q_CM_CS_1f_representatives.results
```

| reads > 95% | all reads | % | iteration |
| --- | --- | --- | --- |
| 776252 | 7190360 | 10.8 | 0 |
| 5393709 | 7190360 | 75.0 | 10 refs |
| 5926307 | 7190360 | 82.4 | 19 refs |
| 6396388 | 7190360 | 89.0 | 282 refs |
| 6438813 | 7190360 | 89.6 | 289 refs |

## 10 Contingency tables (OTUs)

### 10.1 Length ditribution

```
# lstm
cd ~/Science/projects/Quercus_suber/data/

FASTA="ITS1_Q_CM_CS.fas"
OUTPUT="${FASTA/.fas/_length_distribution.data}"
FOLDER="../results"

paste - - < "${FASTA}" | \
    awk 'BEGIN {FS = "[=;\t]"}
         {
          a[length($NF)] += $3
         } END {
          for (i in a) {
              print i, a[i]
          }
         }' | sort -k1,1n > "${FOLDER}/${OUTPUT}"
```

### 10.2 Visualization of best qualities per length

```
# lstm
cd ~/Science/projects/Quercus_suber/data/

QUALITY="ITS1_Q_CM_CS.qual"
FOLDER="../results"
OUTPUT="ITS1_Q_CM_CS_minimal_qualities.data"

mkdir -p "${FOLDER}"

awk '{r = $2 / $3
      if (! a[$3] || a[$3] > r) {a[$3] = r}
      } END {
      for (l in a) {
           print l, a[l]
      }
     }' "${QUALITY}" | sort -t " " -k1,1n > "${FOLDER}/${OUTPUT}"
```

**import**

```
# aragorn
cd ~/Science/Projects/Quercus_suber/results/Quality_filtering/

scp lstm:~/Science/projects/Quercus_suber/results/ITS1_Q_CM_CS_minimal_qualities.data .
scp lstm:~/Science/projects/Quercus_suber/results/ITS1_Q_CM_CS_length_distribution.data .
```

**plot**

```
library(cowplot)
library(ggplot2)
library(tidyr)
library(dplyr)
library(scales)

## Set working directory
setwd("~/Science/Projects/Quercus_suber/results/Quality_filtering")

## Plot title, labels, and important variables
input <- "ITS1_Q_CM_CS_minimal_qualities.data"
input2 <- "ITS1_Q_CM_CS_length_distribution.data"
output <- gsub(".data", ".png", input)
title <- "Minimal expected error rates observed in a ITS1 MiSeq run"
x_axis_label <- "sequence length (nucleotides)"
y_axis_label <- "minimal expected error rate / length"
max_ee <- 0.0002

## Load data
d <- read.table(input, sep = " ", header = FALSE, dec = ".")
colnames(d) <- c("length", "min_ee")
x_max <- max(d$length)
x_min <- min(d$length)

## Plot
p1 <- ggplot(data = filter(d, min_ee < max_ee),
             aes(x = length, y = min_ee)) +
    geom_point(shape = 20, colour = "steelblue") +
    scale_x_continuous(limits = c(x_min, x_max)) +
    xlab(x_axis_label) +
    ylab(y_axis_label) +
    theme_bw() +
    labs(title = title) +
    theme(legend.justification=c(1,0),
          legend.position=c(1,0),
          axis.title.x = element_blank(),
          legend.title = element_text(face="bold"))

## Load data
d2 <- read.table(input2, sep = " ", header = FALSE, dec = ".")
colnames(d2) <- c("length", "frequency")

## Plot
p2 <- ggplot(d2, aes(x = length, y = frequency)) +
    geom_segment(aes(xend = length, yend = 0), colour = "firebrick", size = 1) +
    xlab(x_axis_label) +
    scale_x_continuous(limits = c(x_min, x_max)) +
    scale_y_continuous(labels = comma) +
    theme_bw()

## Join plots
split <- 1/4
p3 <- ggdraw() +
    draw_plot(p1, 0, split, 1, 1 - split) +
    draw_plot(p2, 0, 0, 1, split)##  +
    ## draw_plot_label(c("A", "B"), c(0, 0), c(1, split), size = 15)

## Save
ggsave(file = gsub(".data", "2.png", input), plot = p3, width = 8, height = 5)
ggsave(file = gsub(".data", "2.svg", input), plot = p3, width = 8, height = 5)

quit(save = "no")
```

### 10.3 OTU table (full)

```
# lstm
cd ~/Science/projects/Quercus_suber/data/

DATASET="ITS1_Q_CM_CS"
FOLDER="../src"
SCRIPT="OTU_contingency_table.py"
FASTA="${DATASET}.fas"
STATS="${DATASET}_1f.stats"
SWARMS="${DATASET}_1f.swarms"
UCHIME="${DATASET}_1f_representatives.uchime"
ASSIGNMENTS="${DATASET}_1f_representatives.results"
QUALITY="${DATASET}.qual"
REPRESENTATIVES="${DATASET}_1f_representatives.fas"
OTU_TABLE="${DATASET}.OTU.table"

python \
    "${FOLDER}/${SCRIPT}" \
    "${REPRESENTATIVES}" \
    "${STATS}" \
    "${SWARMS}" \
    "${UCHIME}" \
    "${QUALITY}" \
    "${ASSIGNMENTS}" \
    ./ITS1_{CM,Q_CS}/*.fas > "${OTU_TABLE}"
```

(takes 2 minutes)

**Sanity check**

```
# lstm
cd ~/Science/projects/Quercus_suber/data/

DATASET="ITS1_Q_CM_CS"

# OTUs
wc -l ${DATASET}.OTU.table \
   ${DATASET}_1f.stats \
   ${DATASET}_1f.swarms

# reads
TOTAL=$(head -n 1 ${DATASET}.OTU.table | \
               tr "\t" "\n" | nl -n ln | \
               grep "total" | cut -f 1)

tail -n +2 ${DATASET}.OTU.table | \
    awk -v TOTAL=${TOTAL} '{s += $(TOTAL)} END {print s}'

awk -F "[=;]" '/^>/ {s += $3} END {print s}' ${DATASET}.fas
```

### 10.4 OTU table filtering (fungi only)

```
# lstm
cd ~/Science/projects/Quercus_suber/data/

TABLE="ITS1_Q_CM_CS.OTU.table"
FILTERED="${TABLE/.table/.filtered.fungi.table}"
head -n 1 "${TABLE}" > "${FILTERED}"
grep "k__Fungi" "${TABLE}" | \
    awk '$7 == "N" && \
         (($5 >= 300 && $9 <= 0.0002) || ($5 < 300 && $9 <= 0.0001)) && \
         ($2 >= 3 || $8 >= 2) && \
         (($11 >= 80.0) || ($11 < 80.0 && $2 > 10000))' >> "${FILTERED}"

## head -n 1 ITS1_Q_CM_CS.OTU.table | tr "\t" "\n" | nl | head -n 13
#  1  OTU
#  2  total
#  3  cloud
#  4  amplicon
#  5  length
#  6  abundance
#  7  chimera
#  8  spread
#  9  quality
# 10  sequence
# 11  identity
# 12  taxonomy
# 13  references
```

### 10.5 OTU table filtering (streptophyta only)

```
# lstm
cd ~/Science/projects/Quercus_suber/data/

TABLE="ITS1_Q_CM_CS.OTU.table"
FILTERED="${TABLE/.table/.filtered.streptophyta.table}"
head -n 1 "${TABLE}" > "${FILTERED}"
grep "Streptophyta" "${TABLE}" | \
    awk '$7 == "N" && \
         (($5 >= 300 && $9 <= 0.0002) || ($5 < 300 && $9 <= 0.0001)) && \
         ($2 >= 3 || $8 >= 2) && \
         (($11 >= 80.0) || ($11 < 80.0 && $2 > 10000))' >> "${FILTERED}"

## head -n 1 ITS1_Q_CM_CS.OTU.table | tr "\t" "\n" | nl | head -n 13
#  1  OTU
#  2  total
#  3  cloud
#  4  amplicon
#  5  length
#  6  abundance
#  7  chimera
#  8  spread
#  9  quality
# 10  sequence
# 11  identity
# 12  taxonomy
# 13  references
```

2,488 OTUs remain.

### 10.6 OTU table filtering (non-streptophyta, non-fungi)

To give a precise taxonomic breakdown, I need to evaluate the share of
OTUs that are not assigned to Fungi or Streptophyta. Interestingly, if
we put aside the 40,845 "No hit" OTUs, the 468 "unassigned" OTUs and
one conflictual OTU (only stars), the only remaining taxa is the
Oomycete *Pythium paroecandrum*.

This is an effect of the low coverage of the Unite ITS database. I
added Oomycete references because they were abundant in our
data.

Maybe I should check again for the new ITS1 datasets if I am not
missing something important, just because the references are not
present in my database.

```
# lstm
cd ~/Science/Projects/Quercus_suber/results/ITS1/

TABLE="ITS1_Q_CM_CS.OTU.table"
FILTERED="${TABLE/.table/.filtered.misc.table}"
head -n 1 "${TABLE}" > "${FILTERED}"
tail -n +2 "${TABLE}" | \
    grep -vE "Streptophyta|Fungi" "${TABLE}" | \
    awk '$7 == "N" && \
         (($5 >= 300 && $9 <= 0.0002) || ($5 < 300 && $9 <= 0.0001)) && \
         ($2 >= 3 || $8 >= 2) && \
         (($11 >= 80.0) || ($11 < 80.0 && $2 > 10000))' >> "${FILTERED}"

## head -n 1 ITS1_Q_CM_CS.OTU.table | tr "\t" "\n" | nl | head -n 13
#  1  OTU
#  2  total
#  3  cloud
#  4  amplicon
#  5  length
#  6  abundance
#  7  chimera
#  8  spread
#  9  quality
# 10  sequence
# 11  identity
# 12  taxonomy
# 13  references
```

833 OTUs remain.

## 11 Stampa plots

#### 11.0.1 All OTUs

```
# lstm
cd ~/Science/projects/Quercus_suber/data/

export LC_ALL=C
DATASET="ITS1_Q_CM_CS"
TABLE="${DATASET}.OTU.table"

# Get starting column
TOTAL=$(head -n 1 "${TABLE}" | tr "\t" "\n" | nl | grep "total" | awk '{print $1}')
IDENTITY=$(head -n 1 "${TABLE}" | tr "\t" "\n" | nl | grep "identity" | awk '{print $1}')

echo $IDENTITY

# Sum reads
awk -v TOTAL="${TOTAL}" \
    -v IDENTITY="${IDENTITY}" \
    'BEGIN {FS = "\t"}
     {if (NR == 1) {next}
      stampa[$IDENTITY] += $TOTAL
     } END {
      for (id in stampa) {
          print id, stampa[id]
      }
     }' "${TABLE}" | sort -k1,1n > "${TABLE/.table/.stampa}"
```

Inject the results in ggplot2

```
library(ggplot2)
library(scales)

setwd("~/Science/projects/Quercus_suber/data/")
input <- "ITS1_Q_CM_CS.OTU.stampa" 
TITLE <- "All OTUs"

## Load the data
d <- read.table(input, sep = " ", dec = ".")
colnames(d) <- c("identities", "abundance")
d$identities <- d$identities / 100

## Get the max abundance value
y_max <- max(d$abundance)

## Plot
ggplot(d, aes(x = identities, y = abundance)) +
    geom_segment(aes(xend = identities, yend = 0), colour = "darkred", size = 1) +
    scale_x_continuous(labels = percent, limits = c(0.5, 1)) +
    scale_y_continuous(labels=comma) +
    xlab("identity with a reference sequence") +
    ylab("number of environmental sequences") +
    annotate("text", x = 0.50, y = y_max * 0.9, hjust = 0, colour = "grey", size = 8, label = TITLE)

## Output to PDF
output <- gsub(".stampa", "_stampa.pdf", input, fixed = TRUE)
ggsave(file = output, width = 8 , height = 5)

quit(save="no")
```

#### 11.0.2 Fungi

```
# lstm
cd ~/Science/projects/Quercus_suber/data/

export LC_ALL=C
DATASET="ITS1_Q_CM_CS"
TABLE="${DATASET}.OTU.filtered.fungi.table"

# Get starting column
TOTAL=$(head -n 1 "${TABLE}" | tr "\t" "\n" | nl | grep "total" | awk '{print $1}')
IDENTITY=$(head -n 1 "${TABLE}" | tr "\t" "\n" | nl | grep "identity" | awk '{print $1}')

# Sum reads
awk -v TOTAL="${TOTAL}" \
    -v IDENTITY="${IDENTITY}" \
    'BEGIN {FS = "\t"}
     {if (NR == 1) {next}
      stampa[$IDENTITY] += $TOTAL
     } END {
      for (id in stampa) {
          print id, stampa[id]
      }
     }' "${TABLE}" | sort -k1,1n > "${TABLE/.table/.stampa}"
```

Inject the results in ggplot2

```
library(ggplot2)
library(scales)

setwd("~/Science/projects/Quercus_suber/data/")
input <- "ITS1_Q_CM_CS.OTU.filtered.fungi.stampa" 
TITLE <- "Fungi"

## Load the data
d <- read.table(input, sep = " ", dec = ".")
colnames(d) <- c("identities", "abundance")
d$identities <- d$identities / 100

## Get the max abundance value
y_max <- max(d$abundance)

## Plot
ggplot(d, aes(x = identities, y = abundance)) +
    geom_segment(aes(xend = identities, yend = 0), colour = "darkred", size = 1) +
    scale_x_continuous(labels = percent, limits = c(0.5, 1)) +
    scale_y_continuous(labels=comma) +
    xlab("identity with a reference sequence") +
    ylab("number of environmental sequences") +
    annotate("text", x = 0.50, y = y_max * 0.9, hjust = 0, colour = "grey", size = 8, label = TITLE)

## Output to PDF
output <- gsub(".stampa", "_stampa.pdf", input, fixed = TRUE)
ggsave(file = output, width = 8 , height = 5)

quit(save="no")
```

**transfer**

```
# aragorn
cd ~/Science/Projects/Quercus_suber/results/ITS1/
scp lstm:/home/lstm/Science/projects/Quercus_suber/data/ITS1_Q_CM_CS.OTU.*.pdf .
```

#### 11.0.3 Streptophyta

```
# lstm
cd ~/Science/projects/Quercus_suber/data/

export LC_ALL=C
DATASET="ITS1_Q_CM_CS"
TABLE="${DATASET}.OTU.filtered.streptophyta.table"

# Get starting column
TOTAL=$(head -n 1 "${TABLE}" | tr "\t" "\n" | nl | grep "total" | awk '{print $1}')
IDENTITY=$(head -n 1 "${TABLE}" | tr "\t" "\n" | nl | grep "identity" | awk '{print $1}')

# Sum reads
awk -v TOTAL="${TOTAL}" \
    -v IDENTITY="${IDENTITY}" \
    'BEGIN {FS = "\t"}
     {if (NR == 1) {next}
      stampa[$IDENTITY] += $TOTAL
     } END {
      for (id in stampa) {
          print id, stampa[id]
      }
     }' "${TABLE}" | sort -k1,1n > "${TABLE/.table/.stampa}"
```

Inject the results in ggplot2

```
library(ggplot2)
library(scales)

setwd("~/Science/projects/Quercus_suber/data/")
input <- "ITS1_Q_CM_CS.OTU.filtered.streptophyta.stampa"
TITLE <- "Streptophyta"

## Load the data
d <- read.table(input, sep = " ", dec = ".")
colnames(d) <- c("identities", "abundance")
d$identities <- d$identities / 100

## Get the max abundance value
y_max <- max(d$abundance)

## Plot
ggplot(d, aes(x = identities, y = abundance)) +
    geom_segment(aes(xend = identities, yend = 0), colour = "darkred", size = 1) +
    scale_x_continuous(labels = percent, limits = c(0.5, 1)) +
    scale_y_continuous(labels=comma) +
    xlab("identity with a reference sequence") +
    ylab("number of environmental sequences") +
    annotate("text", x = 0.50, y = y_max * 0.9, hjust = 0, colour = "grey", size = 8, label = TITLE)

## Output to PDF
output <- gsub(".stampa", "_stampa.pdf", input, fixed = TRUE)
ggsave(file = output, width = 8 , height = 5)

quit(save="no")
```

#### 11.0.4 Other clades

We have 53,515 reads assigned to *Pythium paroecandrum*, a plant
pathogen Stramenopiles infecting carrots and impatiens.

### 11.1 Distribution of quality-filtered OTUs

```
# aragorn
cd ~/Science/Projects/Quercus_suber/results/
scp -C kl:/scratch/mahe/projects/Quercus_suber/data/ITS1_Q_CM_CS.OTU.table .
```

```
library(dplyr)
library(tidyr)
library(ggplot2)
library(scales)

setwd("~/Science/Projects/Quercus_suber/results/")

## Study name (change here)
input <- "ITS1_Q_CM_CS.OTU.table"
title <- "Quercus suber (ITS1)"

## Limits
min_abundance <- 2
min_cloud <- 2
lowest_quality <- 0.0001
quality_threshold <- 0.00007

## Load stats
stats <- read.table(input, sep = "\t", header = TRUE) %>%
    tbl_df() %>%
    select(cloud, length, abundance, quality) %>%
    filter(cloud >= min_cloud & abundance >= min_abundance) %>%
        mutate(new_quality = ifelse(quality > lowest_quality, lowest_quality, quality)) %>%
    select(-quality) %>%
    rename(quality = new_quality) %>%
    arrange(quality)

## Limits
max_abundance <- max(stats$abundance)
max_cloud <- max(stats$cloud)

## Express gradient on a 0 to 1 scale
high <- min(stats$quality)
low <- max(stats$quality)  ## = lowest_quality
a <- 1 / (high - low)
b <- 1 - (high / (high - low))
threshold <- a * quality_threshold + b  ## y = ax + b
threshold

## Plot
ggplot(stats, aes(x = abundance, y = cloud, colour = quality)) +
    geom_point(shape = 21) +
    labs(title = title) +
    scale_x_log10(name = "abundance of central amplicon",
                  breaks = trans_breaks("log10", function(x) 10^x),
                  labels = trans_format("log10", math_format(10^.x)),
                  limits = c(min_abundance, max_abundance)) +
    scale_y_log10(name = "number of amplicons in the OTU",
                  breaks = trans_breaks("log10", function(x) 10^x),
                  labels = trans_format("log10", math_format(10^.x)),
                  limits = c(min_cloud, max_cloud)) +
    scale_colour_gradientn(name = "expected error\nper nucleotide",
                           colours = c("dodgerblue4", "white", "darkred"),
                           values = c(0, threshold, 1)) +
    theme(legend.background = element_rect(colour = "grey"),
          legend.justification = c(1, 0),
          legend.position = c(1, 0))

## Output to PDF
output <- gsub(".table", "_OTU_qualities.pdf", input, fixed = TRUE)
ggsave(file = output, width = 5 , height = 5)

quit(save="no")
```

I forced the redness of low quality OTUs to show what exactly we loose
if we filter on quality.

## 12 Statistical analyses

### 12.1 Rarefaction of Quercus suber data

```
library(tidyr)
library(dplyr)
library(vegan)
library(ggplot2)
library(ggrepel)
library(scales)

## Variables
setwd("~/Science/Projects/Quercus_suber/results/")
input <- "../data/ITS_samples.list"
input2 <- "ITS1_Q_CM_CS.OTU.filtered.quercus.table"
output <- sub(".table", "", input2)
taxonomic_levels <- c("kingdom", "phylum", "class", "order", "family", "genus", "species")
plant <- "Quercus suber"
sample_code <- "Q"
percentage_threshold <- 1
ggrepel_iterations <- 10000

## Import and format sample data
samples <- read.table(input, sep = "\t", header = TRUE, dec = ".") %>%
    tbl_df() %>%
    select(-dataset) %>%
    filter(plant == plant & grepl("^Q", sample))

## Import and format OTU data
OTUs <- read.table(input2, sep = "\t", header = TRUE, dec = ".") %>%
    tbl_df() %>%
    select(OTU, matches("^Q|^CM|^CS", ignore.case = FALSE)) %>%
    gather("sample", "reads", matches("^Q|^CM|^CS", ignore.case = FALSE)) %>%
    filter(grepl("^Q", sample))        

## Merge the two tables (OTUs + samples), keep only target samples
quercus <- left_join(OTUs, samples, by = "sample") %>%
    filter(status == "ND") %>%
    select(-plant, -forest, -status, -plot) %>%
    spread(sample, reads) %>%
    select(-OTU)

#------------------------ Richness and Diversity -----------------------------#

glimpse(quercus)

## transpose (to get OTUs in columns)
quercus <- t(quercus)

## Identify low samples and outliers
quantile(rowSums(quercus))
min(rowSums(quercus))
sort(rowSums(quercus))

## Randomly subsample the table, so all samples have the same number of reads
quercus_rarefied <- rrarefy(quercus, min(rowSums(quercus)))

## richness
estimateR(quercus_rarefied)

## Shannon index H (richness + evenness)
H <- diversity(quercus_rarefied, index = "shannon", MARGIN = 1, base = exp(1))
H

## Pielou’s index of evenness: (0-1, 1 = max. evenness)
J <- H/log(dim(quercus_rarefied)[2])
J

## Simpson's D index: (richness + evenness, 0-1; 1 - D rises as evenness increases)
D <- diversity(quercus_rarefied, "simpson")
D
inv_D <- diversity(quercus_rarefied, "invsimpson")
inv_D

#------------------------------------ NMDS ------------------------------------#

## Bray Curtis dissimilarity matrix
quercus_rarefied.bray <- vegdist(quercus_rarefied, method = "bray")

## NMDS analyses (1,000 replicates)
quercus_rarefied.bray.nmds <- metaMDS(quercus_rarefied.bray)
stress <- quercus_rarefied.bray.nmds$stress

## Extract data scores (https://chrischizinski.github.io/rstats/2014/04/13/vegan-ggplot2/)
sample <- rownames(quercus_rarefied)
data.scores <- as.data.frame(scores(quercus_rarefied.bray.nmds))
data.scores$site <- rownames(data.scores)
data.scores$sample <- sample
x_min <- min(data.scores$NMDS1)
y_max <- max(data.scores$NMDS2)
x_max <- max(data.scores$NMDS1)
y_min <- min(data.scores$NMDS2)
head(data.scores)

## Merge the two tables (OTUs + samples), keep only target samples
data.scores <- left_join(data.scores, samples, by = "sample") %>%
    select(-site, -plant, -status)
data.scores$sample <- as.factor(data.scores$sample)
head(data.scores)
stress_annotation <- paste("stress: ", round(stress, digits = 4), sep = "")

## Plot
cbbPalette <- c("#000000", "#E69F00", "#56B4E9", "#009E73", "#F0E442", "#0072B2", "#D55E00", "#CC79A7")
ggplot(data = data.scores, aes(x = NMDS1, y = NMDS2, label = sample)) +
    geom_point(aes(colour = forest), size = 3) +
    theme_bw(base_size = 16) +
    guides(colour = guide_legend(title = NULL)) +
    scale_colour_manual(values = cbbPalette) +
    coord_equal() +
    annotate("text", x = x_min, y = y_min, label = stress_annotation, hjust = 0) +
    theme(legend.justification = c(1,0), legend.position = c(1,0.1)) +
    geom_text_repel(alpha = 0.5,
                    size = 2.75,
                    segment.size = 0.25,
                    segment.color = "grey",
                    max.iter = ggrepel_iterations)

output_file <- paste(output, "_MDS.pdf", sep = "")
ggsave(output_file, width = 6, height = 5)

## Plot (no sample names)
ggplot(data = data.scores, aes(x = NMDS1, y = NMDS2, label = sample)) +
    geom_point(aes(colour = forest), size = 3) +
    theme_bw(base_size = 16) +
    guides(colour = guide_legend(title = NULL)) +
    scale_colour_manual(values = cbbPalette) +
    coord_equal() +
    annotate("text", x = x_min, y = y_min, label = stress_annotation, hjust = 0) +
    theme(legend.justification = c(1,0), legend.position = c(1,0.1))

output_file <- paste(output, "_MDS_no_labels.pdf", sep = "")
ggsave(output_file, width = 6, height = 5)

glimpse(quercus)


#------------------------ Absolute barplots -----------------------------------#

## Barcharts (reads)
p1 <- ggplot(quercus, aes(x = status, y = sum, fill = order)) +
    geom_bar(stat = "identity") +
    facet_grid(. ~ forest) +
    scale_y_continuous(labels = comma) +
    scale_fill_discrete(name = legend_title, guide = guide_legend(reverse=TRUE)) +
    scale_x_discrete() +
    ylab("number of observed reads") +
    xlab("forest status (non-target or target)") +
    theme_bw() +
    theme(axis.text  = element_text(size = 11),
          axis.title.x = element_text(vjust = 0),
          strip.text.x = element_text(size=12, face="bold"))

## Barcharts (OTUs)

## Merge the two tables (OTUs + samples) and count OTUs per condition
## (remove zero values first)
quercus2 <- left_join(OTUs, samples, by = "sample")
quercus2$order <- as.factor(quercus2$order)
quercus2$sample <- as.factor(quercus2$sample)
quercus2 <- quercus2 %>%
    filter(reads > 0) %>%
    group_by(forest, status, order) %>%
    summarize(sum = n())

## List clades that have significative abundances
main_taxa <- quercus2 %>%
    select(-forest, -status) %>%
    group_by(order) %>%
    tally(wt = sum, sort = TRUE) %>%
    mutate(percentage = 100 * n / sum(quercus2$sum)) %>%
    filter(percentage > percentage_threshold) %>%
    select(-n, -percentage)

## All rows in quercus2 that have a match in main_taxa
quercus2 <- ungroup(quercus2)
quercus2 <- semi_join(quercus2, main_taxa) %>% arrange(order)

## Add accent to Maâmora
levels(quercus2$forest)[levels(quercus2$forest) == "Maamora"] <- "Maâmora"

## Barcharts
p2 <- ggplot(quercus2, aes(x = status, y = sum, fill = order)) +
    geom_bar(stat = "identity") +
    facet_grid(. ~ forest) +
    scale_y_continuous(labels = comma) +
    scale_fill_discrete(name = legend_title, guide = guide_legend(reverse=TRUE)) +
    ylab("number of observed OTUs") +
    xlab("forest status (non-target or target)") +
    theme_bw() +
    theme(axis.text  = element_text(size = 11),
          axis.title.x = element_text(vjust = 0),
          strip.text.x = element_text(size=12, face="bold"))

## Output to PDF (multiplot)
pdf(file = output, width = 11 , height = 10)
multiplot(p1, p2)
dev.off()


#-------------------------- Percentage barplots -------------------------------#

## Barcharts (reads)
p1 <- ggplot(quercus, aes(x = status, y = sum, fill = order)) +
    geom_bar(stat = "identity", position = "fill") +
    facet_grid(. ~ forest) +
    scale_y_continuous(labels = percent_format()) +
    scale_fill_discrete(name = legend_title, guide = guide_legend(reverse=TRUE)) +
    scale_x_discrete() +
    ylab("number of observed reads") +
    xlab("forest status (non-target or target)") +
    theme_bw() +
    theme(axis.text  = element_text(size = 11),
          axis.title.x = element_text(vjust = 0),
          strip.text.x = element_text(size=12, face="bold"))

## Barcharts (OTUs)
p2 <- ggplot(quercus2, aes(x = status, y = sum, fill = order)) +
    geom_bar(stat = "identity", position = "fill") +
    facet_grid(. ~ forest) +
    scale_y_continuous(labels = percent_format()) +
    scale_fill_discrete(name = legend_title, guide = guide_legend(reverse=TRUE)) +
    ylab("number of observed OTUs") +
    xlab("forest status (non-target or target)") +
    theme_bw() +
    theme(axis.text  = element_text(size = 11),
          axis.title.x = element_text(vjust = 0),
          strip.text.x = element_text(size=12, face="bold"))

## Output to PDF (multiplot)
pdf(file = output2, width = 11 , height = 10)
multiplot(p1, p2)
dev.off()

quit(save="no")
```

NMDS varies a little, but it is pointless to multiple the metaMDS
runs. That function already searches for the lowest stress using 20
random starting points. Additional runs only marginally improve the
stress value.

On the other hand, replaying the rarefaction step has a stronger
effect on the stress value.

### 12.2 Rarefaction of Quercus suber data (Fungi) and taxonomic profiles

```
library(tidyr)
library(dplyr)
library(vegan)
library(ggplot2)
library(scales)
library(cowplot)
library(grid)
library(gridExtra)

## Variables
setwd("~/Science/Projects/Quercus_suber/results/")
input <- "ITS_samples.list"
input2 <- "ITS1_Q_CM_CS.OTU.filtered.fungi.table"
taxonomic_levels <- c("kingdom", "phylum", "class", "order", "family", "genus", "species")
target_plant <- "Quercus suber"
sample_code <- "Q"
percentage_threshold <- 1
seed <- 1
legend_title <- paste("orders (>", percentage_threshold, "%)", sep = "")
basename <- gsub(".table", "", input2)
description <- "_rarefied_taxonomic_profiles"
output_absolute <- paste(basename, description, "_absolute.pdf", sep = "")
output_relative <- paste(basename, description, "_relative.pdf", sep = "")

## Import and format sample data
samples <- read.table(input, sep = "\t", header = TRUE, dec = ".") %>%
    tbl_df() %>%
    filter(plant == target_plant) %>%
    select(-dataset, -plot) %>%
    droplevels()

## Import and format OTU data (it's not possible to retain forest origin at that stage)
raw_table <- read.table(input2, sep = "\t", header = TRUE, dec = ".") %>%
    tbl_df() %>%
    select(OTU, taxonomy, starts_with(sample_code, ignore.case = FALSE)) %>%
    separate(taxonomy, taxonomic_levels, sep = "[|]", extra = "drop") %>%
    select(-one_of("kingdom", "phylum", "class", "family", "genus", "species")) %>%
    gather("sample", "reads", starts_with(sample_code)) %>%
    left_join(samples, by = "sample") %>%
    filter(status == "ND") %>%
    select(-plant, -status, -forest) %>%
    spread(sample, reads)

## Clean taxonomic data
raw_table$order[raw_table$order == "*"] <- "unidentified"
raw_table$order <- sub("o__", "", raw_table$order) ## remove "o__" bits
raw_table$order <- as.factor(raw_table$order)

##-------------------------- rarefy -----------------------------------#

## transpose (to get OTUs in columns)
quercus_t <- t(select(raw_table, -OTU, -order))

## Identify low samples and outliers
head(sort(rowSums(quercus_t)))
smallest <- min(rowSums(quercus_t))

## Rarefy the table (fixed seed)
set.seed(seed)
quercus_rarefied_t <- rrarefy(quercus_t, smallest)

## Reduce sample list
samples <- filter(samples, status == "ND") %>% droplevels()

## Merge back with metadata
quercus_rarefied <- bind_cols(select(raw_table, OTU, order),
                              as.data.frame(t(quercus_rarefied_t))) %>%
    gather("sample", "reads", starts_with(sample_code)) %>%
    left_join(samples, by = "sample") %>%
    select(-plant, -status)

##-------------------------- taxonomic profiles ------------------------#

## List clades that have significative abundances (reads)
main_taxa_reads <- quercus_rarefied %>%
    select(-forest) %>%
    count(order, wt = reads, sort = TRUE) %>%
    mutate(percentage = 100 * n / sum(quercus_rarefied$reads)) %>%
    filter(percentage > percentage_threshold) %>%
    select(-n, -percentage)

## List clades that have significative abundances (OTUs)
main_taxa_OTUs <- quercus_rarefied %>%
    select(-forest) %>%
    filter(reads > 0) %>%
    count(order, sort = TRUE) %>%
    mutate(percentage = 100 * n / nrow(raw_table)) %>%
    filter(percentage > percentage_threshold) %>%
    select(-n, -percentage)

## Synchronize list of significant taxa
main_taxa <- bind_rows(main_taxa_reads, main_taxa_OTUs) %>%
    distinct()

## All rows in quercus that have a match in main_taxa (reads)
quercus_rarefied_reads <- quercus_rarefied %>%
    count(forest, order, wt = reads)  %>%
    rename(reads = n) %>%
    semi_join(main_taxa, by = "order") %>%
    arrange(order)

## All rows in quercus that have a match in main_taxa (OTUs)
quercus_rarefied_OTUs <- quercus_rarefied %>%
    filter(reads > 0) %>%
    count(forest, order)  %>%
    rename(OTUs = n) %>%
    semi_join(main_taxa, by = "order") %>%
    arrange(order)

##------------------------ Absolute barplots ---------------------------------#

## Barcharts (reads)
p1 <- ggplot(quercus_rarefied_reads, aes(x = forest, y = reads, fill = order)) +
    geom_bar(stat = "identity", colour = "white", size = 0.1) +
    scale_y_continuous(labels = comma) +
    scale_fill_discrete(name = legend_title,
                        guide = guide_legend(reverse = TRUE)) +
    scale_x_discrete() +
    ylab("number of observed reads") +
    xlab("forest") +
    theme_bw(base_size = 16) +
    theme(axis.title.x = element_text(vjust = 0))

## Barcharts (OTUs)
p2 <- ggplot(quercus_rarefied_OTUs, aes(x = forest, y = OTUs, fill = order)) +
    geom_bar(stat = "identity", colour = "white", size = 0.1) +
    scale_y_continuous(labels = comma) +
    scale_fill_discrete(name = legend_title,
                        guide = guide_legend(reverse = TRUE)) +
    ylab("number of observed OTUs") +
    xlab("forest") +
    theme_bw(base_size = 16) +
    theme(axis.title.x = element_text(vjust = 0))

## Align plots vertically (https://gist.github.com/tomhopper/faa24797bb44addeba79)
gA <- ggplot_gtable(ggplot_build(p1))
gB <- ggplot_gtable(ggplot_build(p2))
maxWidth = grid::unit.pmax(gA$widths, gB$widths)
gA$widths <- as.list(maxWidth)
gB$widths <- as.list(maxWidth)
grid.newpage()

## Save
split <- 1/2
pdf(file = output_absolute, width = 14, height = 10)
grid.arrange(arrangeGrob(gA, gB, nrow = 1, widths = c(1 - split, split)))
dev.off()

##------------------------ Absolute barplots ---------------------------------#

## Barcharts (reads)
p1 <- ggplot(quercus_rarefied_reads, aes(x = forest, y = reads, fill = order)) +
    geom_bar(stat = "identity", position = "fill", colour = "white", size = 0.1) +
    scale_y_continuous(labels = comma) +
    scale_fill_discrete(name = legend_title,
                        guide = guide_legend(reverse = TRUE)) +
    scale_x_discrete() +
    ylab("number of observed reads") +
    xlab("forest") +
    theme_bw(base_size = 16) +
    theme(axis.title.x = element_text(vjust = 0))

## Barcharts (OTUs)
p2 <- ggplot(quercus_rarefied_OTUs, aes(x = forest, y = OTUs, fill = order)) +
    geom_bar(stat = "identity", position = "fill", colour = "white", size = 0.1) +
    scale_y_continuous(labels = comma) +
    scale_fill_discrete(name = legend_title,
                        guide = guide_legend(reverse = TRUE)) +
    ylab("number of observed OTUs") +
    xlab("forest") +
    theme_bw(base_size = 16) +
    theme(axis.title.x = element_text(vjust = 0))

## Align plots vertically (https://gist.github.com/tomhopper/faa24797bb44addeba79)
gA <- ggplot_gtable(ggplot_build(p1))
gB <- ggplot_gtable(ggplot_build(p2))
maxWidth = grid::unit.pmax(gA$widths, gB$widths)
gA$widths <- as.list(maxWidth)
gB$widths <- as.list(maxWidth)
grid.newpage()

## Save
split <- 1/2
pdf(file = output_relative, width = 14, height = 10)
grid.arrange(arrangeGrob(gA, gB, nrow = 1, widths = c(1 - split, split)))
dev.off()

quit(save="no")
```

There is a redundant section of code. That script needs a major
investigation.

### 12.3 How many raw reads in the Quercus ND samples?

```
# kl
cd /scratch/mahe/projects/Quercus_suber/data/

grep "Quercus" ITS_samples.list | \
    awk '{if ($NF == "ND") {print $2}}' | \
    while read SAMPLE ; do
        find ./ITS1_Q_CS/ -name "${SAMPLE}.fas" -execdir cat {} \;
    done | awk 'BEGIN {FS = "[;=]"} {if (/^>/) {s += $3 ; c += 1}} END {print s, c}'
```

We started with 1,129,145 reads (325,378 unique sequences) containing
the expected barcodes and primers, and of length equal or greater than
32 nucleotides.

```
library(tidyr)
library(dplyr)

## Variables
setwd("~/Science/Projects/Quercus_suber/results/ITS1/")
input <- "../../data/ITS_samples.list"
input2 <- "ITS1_Q_CM_CS.OTU.filtered.quercus.table"
input3 <- "ITS1_Q_CM_CS.OTU.filtered.fungi.table"
input4 <- "ITS1_Q_CM_CS.OTU.filtered.misc.table"
plant <- "Quercus suber"
sample_code <- "Q"

## Import and format sample data
samples <- read.table(input, sep = "\t", header = TRUE, dec = ".") %>%
    tbl_df() %>%
    select(-dataset) %>%
    filter(plant == plant & grepl("^Q", sample)) %>%
    droplevels()

## Import and format OTU data
quercus <- read.table(input2, sep = "\t", header = TRUE, dec = ".") %>%
    tbl_df() %>%
    select(OTU, matches("^Q", ignore.case = FALSE)) %>%
    gather("sample", "reads", matches("^Q", ignore.case = FALSE)) %>%
    left_join(samples, by = "sample") %>%
    filter(status == "ND" & reads > 0) %>%
    select(-plant, -forest, -status, -plot) %>%
    droplevels()

## how many reads?
summarise(quercus, sum = sum(reads))
## how many OTUs?
summarise(quercus, distinct = n_distinct(OTU))

## Import and format OTU data
fungi <- read.table(input3, sep = "\t", header = TRUE, dec = ".") %>%
    tbl_df() %>%
    select(OTU, matches("^Q", ignore.case = FALSE)) %>%
    gather("sample", "reads", matches("^Q", ignore.case = FALSE)) %>%
    left_join(samples, by = "sample") %>%
    filter(status == "ND" & reads > 0) %>%
    select(-plant, -forest, -status, -plot) %>%
    droplevels()

## how many reads?
summarise(fungi, sum = sum(reads))
## how many OTUs?
summarise(fungi, distinct = n_distinct(OTU))

## Import and format OTU data
misc <- read.table(input4, sep = "\t", header = TRUE, dec = ".") %>%
    tbl_df() %>%
    select(OTU, matches("^Q", ignore.case = FALSE)) %>%
    gather("sample", "reads", matches("^Q", ignore.case = FALSE)) %>%
    left_join(samples, by = "sample") %>%
    filter(status == "ND" & reads > 0) %>%
    select(-plant, -forest, -status, -plot) %>%
    droplevels()

## how many reads?
summarise(misc, sum = sum(reads))
## how many OTUs?
summarise(misc, distinct = n_distinct(OTU))

quit(save="no")
```

Here are the results post-filtering:

| taxa | OTUs | reads |
| --- | --- | --- |
| Streptophyta | 924 | 477110 |
| Fungi | 2517 | 315597 |
| Other (Oomycetes) | 13 | 224 |

The "Other" category is probably a bit underestimated. Adding more
references for groups other than Fungi and Streptophyta would likely
increase a bit the volume of "Other".

### 12.4 Quercus suber ITS1 Fungi vs environment parameters

```
library(tidyr)
library(dplyr)
library(vegan)
library(ggplot2)
library(ggrepel)
library(scales)

## Variables
setwd("~/Science/Projects/Quercus_suber/results/ITS1/")
input1 <- "ITS1_Q_CM_CS.OTU.filtered.fungi.table"
input2 <- "ITS_samples.list"
input3 <- "../../data/Quercus_suber_ND_external_parameters.csv"
output_NMDS <- paste(sub(".table", "", input2), "_NMDS_colored.pdf", sep = "")
output_NMDS_labels <- paste(sub(".table", "", input2), "_NMDS_colored_labels.pdf", sep = "")
output_NMDS_envfit <- paste(sub(".table", "", input2), "_NMDS_colored_envfit.pdf", sep = "")
title_NMDS <- "Fungi communities associated with cork oaks\nin Moroccan forests (Bray-Curtis)"
width <- 8
height <- 6
set.seed(1)
taxonomic_levels <- c("kingdom", "phylum", "class", "order", "family", "genus", "species")
target_plant <- "Quercus suber"
target_status <- "ND"
sample_code <- "Q"
percentage_threshold <- 1
cbPalette <- c("#F0E442", "#999999", "#E69F00", "#56B4E9", "#009E73", "#0072B2", "#D55E00", "#CC79A7")

## Import and format OTU data
OTUs <- read.table(input1, sep = "\t", header = TRUE, dec = ".") %>%
    tbl_df() %>%
    select(OTU, starts_with(sample_code)) %>%
    select(-quality) %>%
    gather("sample", "reads", starts_with(sample_code)) %>%
    droplevels()

## Import and format sample data
metadata <- read.table(input2, sep = "\t", header = TRUE, dec = ".") %>%
    tbl_df() %>%
    select(-dataset, -plot) %>%
    filter(plant == target_plant) %>%
    droplevels()

## Import and format additional sample data
parameters <- read.table(input3, sep = "\t", header = TRUE, dec = ".", quote = "\"") %>%
    tbl_df() %>%
    select(-species, -status, -latitude, -longitude,
           -altitude, -starts_with("tree_"), -Ca_.)

## Visualize correlations among environmental parameters
pdf(file = "Quercus_suber_ND_external_parameters.pdf", width = 10, height = 10)
pairs(select(parameters, -sample, -forest, -lot))
dev.off()

## Test correlations between pairs of environmental parameters
p <- select(parameters, -sample, -forest, -lot)
pairs <- combn(colnames(p), 2)
for (method in c("kendall", "pearson", "spearman")) {
    for (i in 1:(length(pairs) / 2)) {
        p1 <- pairs[1, i]
        p2 <- pairs[2, i]
        print(paste(p1, p2))
        print(cor.test(unname(unlist(p[p1])),
                       unname(unlist(p[p2])),
                       method = method))
    }
}

## Merge the two tables (OTUs + samples), keep only target samples
fungi <- left_join(OTUs, metadata, by = "sample") %>%
    filter(status == target_status) %>%
    select(-plant, -forest, -status) %>%
    spread(sample, reads) %>%
    select(-OTU)

##-------------------------- Vegan and NMDS -----------------------------------#

## transpose (to get OTUs in columns)
fungi <- t(fungi)

## Identify low samples and outliers
quantile(rowSums(fungi))
min(rowSums(fungi))
sort(rowSums(fungi))

## Randomly subsample the table, so all samples have the same number of reads
fungi_rarefied <- rrarefy(fungi, min(rowSums(fungi)))

## richness
estimateR(fungi_rarefied)

## Shannon index H (richness + evenness)
H <- diversity(fungi_rarefied, index = "shannon", MARGIN = 1, base = exp(1))
H

## Pielou’s index of evenness: (0-1, 1 = max. evenness)
J <- H/log(dim(fungi_rarefied)[2])
J

## Simpson's D index: (richness + evenness, 0-1; 1 - D rises as evenness increases)
D <- diversity(fungi_rarefied, "simpson")
D
inv_D <- diversity(fungi_rarefied, "invsimpson")
inv_D

## Bray Curtis dissimilarity matrix
fungi_rarefied.bray <- vegdist(fungi_rarefied, method = "bray")

## NMDS analyses
fungi_rarefied.bray.nmds <- metaMDS(fungi_rarefied.bray)

## Extract sample coordinates (https://chrischizinski.github.io/rstats/2014/04/13/vegan-ggplot2/)
stress <- fungi_rarefied.bray.nmds$stress
data.scores <- as.data.frame(scores(fungi_rarefied.bray.nmds))
data.scores$sample <- as.factor(rownames(data.scores))

## Merge the two tables to add forest names (only target samples)
metadata <- metadata %>%
    filter(status == target_status) %>%
    select(-plant, -status) %>%
    droplevels()
data.scores <- left_join(data.scores, metadata, by = "sample")
x_min <- min(data.scores$NMDS1)
x_max <- min(data.scores$NMDS1)
y_max <- max(data.scores$NMDS2)
stress_annotation <- paste("stress: ", round(stress, digits = 4), sep = "")

## Plot (without sample labels)
ggplot(data = data.scores, aes(x = NMDS1, y = NMDS2, label = sample)) +
    geom_point(aes(colour = forest), size = 3) +
    theme_bw(base_size = 16) +
    theme(legend.title = element_blank(),
          legend.position = "bottom",
          legend.text = element_text(size = 10)) +
    ggtitle(title_NMDS) +
    annotate("text", x = x_min, y = y_max, hjust = 0, label = stress_annotation) +
    scale_colour_manual(values = cbPalette) +
        coord_equal()

ggsave(output_NMDS, width = width, height = height)

## Plot (with sample labels)
ggplot(data = data.scores, aes(x = NMDS1, y = NMDS2, label = sample)) +
   geom_text_repel(alpha = 0.5,
                   size = 2.75,
                   segment.size = 0.25,
                   segment.color = "grey",
                   max.iter = 1000) +
    geom_point(aes(colour = forest), size = 3) +
    theme_bw(base_size = 16) +
    theme(legend.title = element_blank(),
          legend.position = "bottom",
          legend.text = element_text(size = 10)) +
    ggtitle(title_NMDS) +
    annotate("text", x = x_min, y = y_max, hjust = 0, label = stress_annotation) +
    scale_colour_manual(values = cbPalette) +
    coord_equal()

ggsave(output_NMDS_labels, width = width, height = height)

##-------------------------- Vegan and envfit --------------------------------#

glimpse(fungi_rarefied.bray.nmds)
glimpse(parameters)

## Add environmental parameters to the ordination results
##
## http://stackoverflow.com/questions/14711470/plotting-envfit-vectors-vegan-package-in-ggplot2
vf <- envfit(fungi_rarefied.bray.nmds, parameters, permutations = 9999)
vf
parameters.scores <- as.data.frame(scores(vf, display = "vectors"))
parameters.scores$parameter <- as.factor(rownames(parameters.scores))

## Plot
ggplot() +
    geom_point(data = data.scores,
               aes(x = NMDS1, y = NMDS2, colour = forest),
               size = 3) +
    theme_bw(base_size = 16) +
    theme(legend.title = element_blank(),
          legend.position = "bottom",
          legend.text = element_text(size = 10)) +
    ggtitle(title_NMDS) +
    scale_colour_manual(values = cbPalette) +
    coord_equal() +
    geom_segment(data = parameters.scores,
                 aes(x = 0, xend = NMDS1, y = 0, yend = NMDS2),
                 arrow = arrow(length = unit(0.25, "cm")),
                 colour = "grey") +
    geom_text(data = parameters.scores,
              aes(x = NMDS1, y = NMDS2, label = parameter),
              size = 2.75,
              hjust = 0, vjust = 0)

## output
ggsave(output_NMDS_envfit, width = width, height = height)

quit(save="no")
```

Increasing the number of permutations produces more precise p-values.

```
***VECTORS

                   NMDS1    NMDS2     r2 Pr(>r)    
pH               0.94034  0.34024 0.5238 0.0002 ***
N_total_.        0.75175  0.65945 0.2645 0.0248 *  
C_total_.        0.46470  0.88547 0.2676 0.0217 *  
CN_ratio        -0.60758  0.79426 0.5592 0.0002 ***
P_total          0.25916  0.96584 0.4598 0.0003 ***
P_available     -0.20510  0.97874 0.5559 0.0001 ***
Mg_.             0.07739  0.99700 0.4554 0.0003 ***
Na_.             0.22786  0.97369 0.3761 0.0030 ** 
K_.              0.35212  0.93596 0.3847 0.0020 ** 
cation_exchange  0.23164  0.97280 0.3734 0.0025 ** 
---
Signif. codes:  0 ‘***’ 0.001 ‘**’ 0.01 ‘*’ 0.05 ‘.’ 0.1 ‘ ’ 1
P values based on 9999 permutations.

***FACTORS:

Goodness of fit:
           r2 Pr(>r)    
sample 1.0000 1.0000    
forest 0.5512 0.0001 ***
lot    0.6300 0.0017 ** 
---
Signif. codes:  0 ‘***’ 0.001 ‘**’ 0.01 ‘*’ 0.05 ‘.’ 0.1 ‘ ’ 1
P values based on 9999 permutations.
```

As expected, forest and lot are strong descriptors of the diversity
profiles observed. Measured chemical parameters all have a significant
influence on fungi diversity.

### 12.5 Quercus suber ITS1 Fungi ternary plots (order level)

**install ggtern and depencies**

```
# aragorn
su
apt-get install r-cran-rcpparmadillo
R
install.packages('ggtern')
```

- pool OTUs per forest,
- rarefy at the forest level,
- plot with ggtern

```
library(tidyr)
library(dplyr)
library(ggplot2)
library(ggtern)
library(scales)
library(vegan)

## Variables
setwd("~/Science/Projects/Quercus_suber/results/ITS1/")
input1 <- "ITS1_Q_CM_CS.OTU.filtered.fungi.table"
input2 <- "ITS_samples.list"
output_tern <- paste(sub(".table", "", input1), "_ternary_plot.pdf", sep = "")
title_tern <- "Fungi communities associated with cork oaks\nin Moroccan forests (Bray-Curtis)"
width <- 10
height <- 8
set.seed(1)
taxonomic_levels <- c("kingdom", "phylum", "class", "order", "family", "genus", "species")
target_plant <- "Quercus suber"
target_status <- "ND"
sample_code <- "Q"
percentage_threshold <- 1

## Import and format OTU data
OTUs <- read.table(input1, sep = "\t", header = TRUE, dec = ".") %>%
    tbl_df() %>%
    select(OTU, taxonomy, starts_with(sample_code, ignore.case = FALSE)) %>%
    gather("sample", "reads", starts_with(sample_code)) %>%
    droplevels()

## Store taxonomic assignment
taxo_assignments <- read.table(input1, sep = "\t", header = TRUE, dec = ".") %>%
    tbl_df() %>%
    select(OTU, taxonomy) %>%
    droplevels()

## Import and format sample data
metadata <- read.table(input2, sep = "\t", header = TRUE, dec = ".") %>%
    tbl_df() %>%
    select(-dataset, -plot) %>%
    filter(plant == target_plant) %>%
    droplevels()

## Merge the two tables (OTUs + samples), keep only target samples
fungi <- left_join(OTUs, metadata, by = "sample") %>%
    filter(status == target_status) %>%
    select(-plant, -taxonomy, -forest, -status) %>%
    spread(sample, reads) %>%
    select(-OTU) %>%
    droplevels() %>%
    t()

## Remove non-target from sample data (avoid chr conversion)
metadata <- filter(metadata, status == target_status) %>%
    droplevels()

## Randomly subsample the table, so all samples have the same number
## of reads. Reinject taxonomic data. Merge the two tables (fungi +
## samples).
fungi_rarefied <- rrarefy(fungi, min(rowSums(fungi))) %>%
    t() %>%
    as.data.frame() %>%
    bind_cols(taxo_assignments) %>%
    gather("sample", "reads", -OTU, -taxonomy) %>%
    left_join(metadata, by = "sample") %>%
    select(-plant, -status) %>%
    count(OTU, forest, taxonomy, wt = reads) %>%
    spread(forest, n) %>%
    separate(taxonomy, taxonomic_levels, sep = "[|]", extra = "drop") %>%
    select(-one_of("kingdom", "phylum", "class", "family", "genus", "species")) %>%
    mutate(order = as.factor(sub("o__", "", order))) %>%
    filter(order != "*") %>%
    mutate(total = Benslimane + Chefchaoun + Maâmora) %>%
    filter(total > 0) %>%
    arrange(desc(total)) %>%
    mutate(Benslimane = Benslimane / total) %>%
    mutate(Chefchaoun = Chefchaoun / total) %>%
    mutate(Maâmora = Maâmora / total) %>%
    droplevels()

## How many reads per order?
count(fungi_rarefied, order, wt = total) %>% arrange(desc(n))

## Plot (all fungi orders)
ggtern(data = fungi_rarefied,
       aes(Benslimane, Chefchaoun, Maâmora, color = order)) +
    geom_point(aes(fill = order, size = total), colour = "black", shape = 21) +
    theme_bw(base_size = 9) +
    scale_size_area(max_size = 30, guide = FALSE) +
    theme(legend.title = element_blank(),
          legend.position = "bottom",
          legend.text = element_text(size = 8)) +
    guides(fill = guide_legend(override.aes = list(size = 7)))
ggsave(output_tern, width = width, height = height)

## Plot (each fungi order)
multiplier <- 0.7
width <- 10 * multiplier
height <- 8 * multiplier
list_of_orders <- levels(fungi_rarefied$order)
n_orders <- length(list_of_orders)
list_of_colors <- hue_pal()(n_orders)
grand_max <- max(fungi_rarefied$total)
for (i in 1:n_orders) {
    ## Create plot title
    nbr_of_OTUs <- filter(fungi_rarefied, order == list_of_orders[i]) %>% count()
    nbr_of_reads <- filter(fungi_rarefied, order == list_of_orders[i]) %>% count(wt = total)
    OTUs <- "OTUs"
    if (nbr_of_OTUs$n == 1) {OTUs <- "OTU"}
    title <- paste(list_of_orders[i],
                   " (", format(nbr_of_OTUs$n), " ", OTUs, ", ",
                   format(nbr_of_reads$n), " reads)", sep = "")
    ## How large is the largest OTU for that taxa?
    local_max <- filter(fungi_rarefied, order == list_of_orders[i]) %>%
        select(total) %>%
        max()
    ## Plot and save to a pdf
    ggtern(data = fungi_rarefied,
           aes(Benslimane, Chefchaoun, Maâmora, size = total)) +
        geom_point(data = filter(fungi_rarefied, order == list_of_orders[i]),
                   fill = list_of_colors[i],
                   colour = "black", shape = 21) +
        theme_bw(base_size = 9) +
        scale_size_area(max_size = local_max * 30 / grand_max, guide = FALSE) +
        ggtitle(title) +
        theme(legend.position = "none") +
        guides(fill = guide_legend(override.aes = list(size = 7)))
    output_tern <- paste(sub(".table", "", input1), "_",
                         tolower(list_of_orders[i]),
                         "_ternary_plot.pdf", sep = "")
    ggsave(output_tern, width = width, height = height)    
}

quit(save="no")
```

**Make a nice pdf document**

```
# aragorn
cd ~/Science/Projects/Quercus_suber/results/ITS1/
pdfjoin ITS1_Q_CM_CS.OTU.filtered.fungi_ternary_plot.pdf ITS1_Q_CM_CS.OTU.filtered.fungi_*_ternary_plot.pdf
mv *-joined.pdf ITS1_Q_CM_CS.OTU.filtered.fungi_ternary_plots.pdf
rm *_ternary_plot.pdf
```

### 12.6 Quercus suber ITS1 Fungi ternary plots (genus level)

Write results to a sub-directory

```
# aragorn
cd ~/Science/Projects/Quercus_suber/results/ITS1/
mkdir -p ./Quercus_ND_ternary_plots_genus/
```

```
library(tidyr)
library(dplyr)
library(ggplot2)
library(ggtern)
library(scales)
library(vegan)

## Variables
setwd("~/Science/Projects/Quercus_suber/results/ITS1/")
input1 <- "ITS1_Q_CM_CS.OTU.filtered.fungi.table"
input2 <- "ITS_samples.list"
output_folder <- "./Quercus_ND_ternary_plots_genus/"
output_tern <- paste(output_folder,
                     sub(".table", "", input1),
                     "_ternary_plot.pdf", sep = "")
title_tern <- "Fungi communities associated with cork oaks\nin Moroccan forests (Bray-Curtis)"
width <- 10
height <- 8
set.seed(1)
taxonomic_levels <- c("kingdom", "phylum", "class", "order", "family", "genus", "species")
target_plant <- "Quercus suber"
target_status <- "ND"
sample_code <- "Q"
percentage_threshold <- 1

## Import and format OTU data
OTUs <- read.table(input1, sep = "\t", header = TRUE, dec = ".") %>%
    tbl_df() %>%
    select(OTU, taxonomy, starts_with(sample_code, ignore.case = FALSE)) %>%
    gather("sample", "reads", starts_with(sample_code)) %>%
    droplevels()

## Store taxonomic assignment
taxo_assignments <- read.table(input1, sep = "\t", header = TRUE, dec = ".") %>%
    tbl_df() %>%
    select(OTU, taxonomy) %>%
    droplevels()

## Import and format sample data
metadata <- read.table(input2, sep = "\t", header = TRUE, dec = ".") %>%
    tbl_df() %>%
    select(-dataset, -plot) %>%
    filter(plant == target_plant) %>%
    droplevels()

## Merge the two tables (OTUs + samples), keep only target samples
fungi <- left_join(OTUs, metadata, by = "sample") %>%
    filter(status == target_status) %>%
    select(-plant, -taxonomy, -forest, -status) %>%
    spread(sample, reads) %>%
    select(-OTU) %>%
    droplevels() %>%
    t()

## Remove non-target from sample data (avoid chr conversion)
metadata <- filter(metadata, status == target_status) %>%
    droplevels()

## Randomly subsample the table, so all samples have the same number
## of reads. Reinject taxonomic data. Merge the two tables (fungi +
## samples).
fungi_rarefied <- rrarefy(fungi, min(rowSums(fungi))) %>%
    t() %>%
    as.data.frame() %>%
    bind_cols(taxo_assignments) %>%
    gather("sample", "reads", -OTU, -taxonomy) %>%
    left_join(metadata, by = "sample") %>%
    select(-plant, -status) %>%
    count(OTU, forest, taxonomy, wt = reads) %>%
    spread(forest, n) %>%
    separate(taxonomy, taxonomic_levels, sep = "[|]", extra = "drop") %>%
    select(-one_of("kingdom", "phylum", "class", "order", "family", "species")) %>%
    mutate(genus = as.factor(sub("g__", "", genus))) %>%
    filter(genus != "*") %>%
    mutate(total = Benslimane + Chefchaoun + Maâmora) %>%
    filter(total > 0) %>%
    arrange(desc(total)) %>%
    mutate(Benslimane = Benslimane / total) %>%
    mutate(Chefchaoun = Chefchaoun / total) %>%
    mutate(Maâmora = Maâmora / total) %>%
    droplevels()

## Produce Table S1
Table_S1  <- rrarefy(fungi, min(rowSums(fungi))) %>%
    t() %>%
    as.data.frame() %>%
    bind_cols(taxo_assignments) %>%
    mutate(OTU = as.integer(OTU)) %>%
    gather("sample", "reads", -OTU, -kingdom, -phylum, -class, -family, -order, -genus, -species) %>%
    left_join(metadata, by = "sample") %>%
    select(-sample, -plant, -status) %>%
    count(OTU, kingdom, phylum, class, family, order, genus, species, forest, wt = reads) %>%
    spread(forest, n) %>%
    mutate(total = Benslimane + Chefchaoun + Maâmora)
write.table(Table_S1, "Table_S1.tsv", sep = "\t", row.names = FALSE)
rm(Table_S1)

## How many reads per genus?
count(fungi_rarefied, genus, wt = total) %>% arrange(desc(n))

## Plot (all fungi genuses)
ggtern(data = fungi_rarefied,
       aes(Benslimane, Chefchaoun, Maâmora, color = genus)) +
    geom_point(aes(fill = genus, size = total), colour = "black", shape = 21) +
    theme_bw(base_size = 9) +
    scale_size_area(max_size = 30, guide = FALSE) +
    theme(legend.title = element_blank(),
          legend.position = "bottom",
          legend.text = element_text(size = 8)) +
    guides(fill = guide_legend(override.aes = list(size = 7)))
ggsave(output_tern, width = width, height = height)

## Plot (each fungi genus)
multiplier <- 0.7
width <- 10 * multiplier
height <- 8 * multiplier
list_of_genuses <- levels(fungi_rarefied$genus)
n_genuses <- length(list_of_genuses)
list_of_colors <- hue_pal()(n_genuses)
grand_max <- max(fungi_rarefied$total)
for (i in 1:n_genuses) {
    ## Create plot title
    nbr_of_OTUs <- filter(fungi_rarefied, genus == list_of_genuses[i]) %>% count()
    nbr_of_reads <- filter(fungi_rarefied, genus == list_of_genuses[i]) %>% count(wt = total)
    OTUs <- "OTUs"
    if (nbr_of_OTUs$n == 1) {OTUs <- "OTU"}
    title <- paste(list_of_genuses[i],
                   " (", format(nbr_of_OTUs$n), " ", OTUs, ", ",
                   format(nbr_of_reads$n), " reads)", sep = "")
    ## How large is the largest OTU for that taxa?
    local_max <- filter(fungi_rarefied, genus == list_of_genuses[i]) %>%
        select(total) %>%
        max()
    ## Plot and save to a pdf
    ggtern(data = fungi_rarefied,
           aes(Benslimane, Chefchaoun, Maâmora, size = total)) +
        geom_point(data = filter(fungi_rarefied, genus == list_of_genuses[i]),
                   fill = list_of_colors[i],
                   colour = "black", shape = 21) +
        theme_bw(base_size = 9) +
        scale_size_area(max_size = local_max * 30 / grand_max, guide = FALSE) +
        ggtitle(title) +
        theme(legend.position = "none") +
        guides(fill = guide_legend(override.aes = list(size = 7)))
    output_tern <- paste(output_folder,
                         sub(".table", "", input1), "_",
                         tolower(list_of_genuses[i]),
                         "_ternary_plot.pdf", sep = "")
    ggsave(output_tern, width = width, height = height)    
}

quit(save="no")
```

**Make a nice pdf document**

```
# aragorn
cd ~/Science/Projects/Quercus_suber/results/ITS1/
pdfjoin ITS1_Q_CM_CS.OTU.filtered.fungi_ternary_plot.pdf ITS1_Q_CM_CS.OTU.filtered.fungi_*_ternary_plot.pdf
mv *-joined.pdf ITS1_Q_CM_CS.OTU.filtered.fungi_ternary_plots.pdf
rm *_ternary_plot.pdf
```

### 12.7 Compare Fungi and Quercus community distance tables

```
library(tidyr)
library(dplyr)
library(ggplot2)
library(ggtern)
library(scales)
library(vegan)

## Variables
setwd("~/Science/Projects/Quercus_suber/results/ITS1/")
input1 <- "ITS_samples.list"
input2 <- "ITS1_Q_CM_CS.OTU.filtered.fungi.table"
input3 <- "ITS1_Q_CM_CS.OTU.filtered.streptophyta.table"
target_plant <- "Quercus suber"
target_status <- "ND"
sample_code <- "Q"

## Import and format sample data
metadata <- read.table(input1, sep = "\t", header = TRUE, dec = ".") %>%
    tbl_df() %>%
    select(-dataset, -plot) %>%
    filter(plant == target_plant) %>%
    droplevels()

## Import and format fungi data
fungi <- read.table(input2, sep = "\t", header = TRUE, dec = ".") %>%
    tbl_df() %>%
    select(OTU, starts_with(sample_code, ignore.case = FALSE)) %>%
    gather("sample", "reads", starts_with(sample_code)) %>%
    left_join(metadata, by = "sample") %>%
    filter(status == target_status) %>%
    select(-plant, -forest, -status) %>%
    spread(sample, reads) %>%
    droplevels()

## Import and format fungi data
plants <- read.table(input3, sep = "\t", header = TRUE, dec = ".") %>%
    tbl_df() %>%
    select(OTU, starts_with(sample_code, ignore.case = FALSE)) %>%
    gather("sample", "reads", starts_with(sample_code)) %>%
    left_join(metadata, by = "sample") %>%
    filter(status == target_status) %>%
    select(-plant, -forest, -status) %>%
    spread(sample, reads) %>%
    droplevels()


## Randomly subsample the table, so all samples have the same number
## of reads. Bray Curtis dissimilarity matrix
fungi_t <- select(fungi, -OTU) %>% t()
set.seed(1)
fungi_rarefied <- rrarefy(fungi_t, min(rowSums(fungi_t)))
fungi_rarefied.bray <- vegdist(fungi_rarefied, method = "bray")

## Randomly subsample the table, so all samples have the same number
## of reads. Bray Curtis dissimilarity matrix
plants_t <- select(plants, -OTU) %>% t()
set.seed(1)
plants_rarefied <- rrarefy(plants_t, min(rowSums(plants_t)))
plants_rarefied.bray <- vegdist(plants_rarefied, method = "bray")

## Is there a correlation between the fungi and streptophyta
## dissimilarity matrices?
for (method in c("pearson", "spearman", "kendall")) {
    print(mantel(xdis = fungi_rarefied.bray,
                 ydis = plants_rarefied.bray,
                 method = method,
                 permutations = 9999))
}

quit(save="no")
```

| Mantel | statistic | Significance | 90% | 95% | 97.5% | 99% |
| --- | --- | --- | --- | --- | --- | --- |
| Pearson | 0.04645 | 0.271 | 0.103 | 0.135 | 0.163 | 0.199 |
| Spearman | 0.05838 | 0.2682 | 0.128 | 0.165 | 0.197 | 0.237 |
| Kendall | 0.03907 | 0.2695 | 0.0845 | 0.1101 | 0.1336 | 0.1579 |

Based on these Mantel test results, we cannot reject the null
hypothesis that the two dissimilarity matrices, fungi communities and
streptophyta communities, are unrelated with alpha = 0.05.

A plant community distant from other plant communities is not
associated with a fungi community also distant from other fungi
communities.

### 12.8 Compare the distribution of Cenococcum with the distribution of its known parasits

Work on rarefied data. Cenococcum's known parasits are
Cladophialophora and Oidiodendron.

```
library(tidyr)
library(dplyr)
library(vegan)
library(ggplot2)
library(scales)

## Variables
setwd("~/Science/Projects/Quercus_suber/results/ITS1/")
input1 <- "ITS1_Q_CM_CS.OTU.filtered.fungi.table"
input2 <- "ITS_samples.list"
set.seed(1)
taxonomic_levels <- c("kingdom", "phylum", "class", "order", "family", "genus", "species")
target_plant <- "Quercus suber"
target_status <- "ND"
sample_code <- "Q"
percentage_threshold <- 1

## Import and format OTU data
OTUs <- read.table(input1, sep = "\t", header = TRUE, dec = ".") %>%
    tbl_df() %>%
    select(OTU, taxonomy, starts_with(sample_code, ignore.case = FALSE)) %>%
    gather("sample", "reads", starts_with(sample_code)) %>%
    droplevels()

## Store taxonomic assignment
taxo_assignments <- read.table(input1, sep = "\t", header = TRUE, dec = ".") %>%
    tbl_df() %>%
    select(OTU, taxonomy) %>%
    droplevels()

## Import and format sample data
metadata <- read.table(input2, sep = "\t", header = TRUE, dec = ".") %>%
    tbl_df() %>%
    select(-dataset, -plot) %>%
    filter(plant == target_plant) %>%
    droplevels()

## Merge the two tables (OTUs + samples), keep only target samples
fungi <- left_join(OTUs, metadata, by = "sample") %>%
    filter(status == target_status) %>%
    select(-plant, -taxonomy, -forest, -status) %>%
    spread(sample, reads) %>%
    select(-OTU) %>%
    droplevels() %>%
    t()

## Remove non-target from sample data (to avoid chr conversion)
metadata <- filter(metadata, status == target_status) %>%
    droplevels()

## Randomly subsample the table, so all samples have the same number
## of reads. Reinject taxonomic data. Merge the two tables (fungi +
## samples).
fungi_rarefied <- rrarefy(fungi, min(rowSums(fungi))) %>%
    t() %>%
    as.data.frame() %>%
    bind_cols(taxo_assignments) %>%
    gather("sample", "reads", -OTU, -taxonomy) %>%
    left_join(metadata, by = "sample") %>%
    select(-plant, -status) %>%
    separate(taxonomy, taxonomic_levels, sep = "[|]", extra = "drop") %>%
    select(-one_of("kingdom", "phylum", "class", "family", "order", "species")) %>%
    mutate(genus = as.factor(sub("g__", "", genus))) %>%
    filter(genus %in% c("Cenococcum", "Cladophialophora", "Oidiodendron")) %>%
    count(genus, sample, forest, wt = reads) %>%
    spread(genus, n)

## ------------------- Cenococcum vs. Cladophialophora ---------------------- #

## Plot (Cenococcum vs. Cladophialophora, global)
ggplot(data = fungi_rarefied,
       aes(x = Cenococcum, y = Cladophialophora)) +
    geom_point(shape = 1) +
    geom_smooth(method = lm, se = FALSE)

## Tests of no correlation (alternative hypothesis: positive correlation)
cor.test(fungi_rarefied$Cenococcum,
         fungi_rarefied$Cladophialophora,
         method = "kendall", alternative = "greater", exact = FALSE)
cor.test(fungi_rarefied$Cenococcum,
         fungi_rarefied$Cladophialophora,
         method = "spearman", alternative = "greater", exact = FALSE)
cor.test(fungi_rarefied$Cenococcum,
         fungi_rarefied$Cladophialophora,
         alternative = "greater")

## Plot (Cenococcum vs. Cladophialophora, by forest)
ggplot(data = fungi_rarefied,
       aes(x = Cenococcum, y = Cladophialophora, colour = forest)) +
    geom_point(shape = 1) +
    geom_smooth(method = lm, se = FALSE)

## -------------------- Cenococcum vs. Oidiodendron ------------------------ #

## Plot (Cenococcum vs. Oidiodendron, global)
ggplot(data = fungi_rarefied,
       aes(x = Cenococcum, y = Oidiodendron)) +
    geom_point(shape = 1) +
    geom_smooth(method = lm, se = FALSE)

## Tests of no correlation (alternative hypothesis: positive correlation)
cor.test(fungi_rarefied$Cenococcum,
         fungi_rarefied$Oidiodendron,
         method = "kendall", alternative = "greater", exact = FALSE)
cor.test(fungi_rarefied$Cenococcum,
         fungi_rarefied$Oidiodendron,
         method = "spearman", alternative = "greater", exact = FALSE)
cor.test(fungi_rarefied$Cenococcum,
         fungi_rarefied$Oidiodendron,
         alternative = "greater")

## Plot (Cenococcum vs. Oidiodendron, by forest)
ggplot(data = fungi_rarefied,
       aes(x = Cenococcum, y = Oidiodendron, colour = forest)) +
    geom_point(shape = 1) +
    geom_smooth(method = lm, se = FALSE)

quit(save="no")
```

## 13 OTU representatives

Export OTU representatives as fasta file.

```
library(tidyr)
library(dplyr)

## Variables
setwd("~/Science/Projects/Quercus_suber/results/ITS1/")
input <- "ITS_samples.list"
input2 <- "ITS1_Q_CM_CS.OTU.filtered.fungi.table"
target_plant <- "Quercus suber"
sample_code <- "Q"
target_status <- "ND"

## Import and format sample data
samples <- read.table(input, sep = "\t", header = TRUE, dec = ".") %>%
    tbl_df() %>%
    filter(plant == target_plant) %>%
    select(-dataset, -plot) %>%
    droplevels()

## Import and format OTU data (it's not possible to retain forest origin at that stage)
raw_table <- read.table(input2, sep = "\t", header = TRUE, dec = ".") %>%
    tbl_df() %>%
    select(OTU, sequence, starts_with(sample_code, ignore.case = FALSE)) %>%
    gather("sample", "reads", starts_with(sample_code)) %>%
    left_join(samples, by = "sample") %>%
    filter(status == target_status) %>%
    select(-plant, -status, -forest) %>%
    count(OTU, sequence, wt = reads) %>%
    filter(n > 0)

## Export sequences in fasta format
fasta <- sprintf(">OTU_%s_%d\n%s",
                 raw_table$OTU, raw_table$n, raw_table$sequence)
fasta_file <- file("ITS1_Q_CM_CS.OTU.filtered.fungi.representatives.fasta")
writeLines(fasta, fasta_file)
close(fasta_file)

quit(save="no")
```

```
>OTU_11_25170
ttacagaaagtaaacgcggatcaatccgcgaacttctaaacctttggcgattgactcatgttgcctcggcgggttctctcgccagaggatacatcaaaactcctgttttaacggtgttgtctgagctacaagcaacgaatcaaaactttcaacaacggatctcttggttctg
>OTU_14_381
ttactgaattgtcaaacgggttgttgctggccctcatagggggcatgtgcacgctctgtttacacatccactcacacctgtgcaccctctgtagttctatggtttgggggaccctgtcttccttctgtggttctacgtctttacacacacactgtaataaagttttatggaatgtacatcgcgtctaacgcaatacaatacaactttcagcaacggatctcttggctctc
>OTU_16_15829
ttatcgtacaatggaggtgctggggttgtcgctgacctttgaaagggtcgtgcacgcctcggtgctttcacacacaatccatctcaccccttttgtgcatcaccgcgtgggggtcccttttagctagttctgaagggggctttcgcgtttttacaaacacacccttttaatgcaatatgtagaatgtcttactttttgcgatcacacgcaatcaatacaactttcaacaacggatctcttggctctc
>OTU_17_20573
ttactgaattgtcaacatgaagttgttgctggccctcatgtgggggcatgtgcacgctctgtttacacatccattcacacctgtgcactctctgtagttctgtggtttgggggctctgtcctcctaccgtggtcctgcatatttacacatacacactgtgataaagtctcatggaatgtatgccgcgtttaacgcaatacaatataactttcagcaacggatctcttggctctc
>OTU_20_60
ttattgaataaacctgatgtgattgttagctggcttttcgaagcatgtgctcgtccgtcatctttatctctccacctgtgcacattttgtagtcttggatacctctcgaggaaactcggattttaggatcgctgtgctgtacaagtcggcttttctttcatttccaagactatgtttttatatatacaccaaagtatgtttatagaatgtcatcaatgggaacttgtttcctataaaattatacaactttcagcaacggatctcttggctctc
>OTU_25_9227
ttattgtataaccgaggtgctagggctgtcgctgaccctttgaagggtcgtgcacgcccaagtgctctctcacatccatctcacccctttgtgcatcaccgcgtgggctacctttttggctttattcaaaaaggttggttcgcgtttttacacacacacctttatgtatagaatgtcttaatttttgcggtcatacgcaataaataaataatacaactttcaacaacggatctcttggctctc
>OTU_26_4048
ttaccgagttcatgcccttacgggtagatctcccaccctgtgttatcattacctttgttgctttggcgggccgccaggctccggtcaggctatcggcttcggctggtacgcgcccgccagaggaccctaacattctgattatcagtgtcgtctgagtactatataatagttaaaactttcaacaacggatctcttggttctg
>OTU_28_923
ttaccaaactttggttttcccccttttaggagggggttgagccactgccctttgtctatgtatacctttgttgtttcctcggtaggcttgcctgccgctaggaatctcctaaacccttgcatcttaagtatttaccttctgataactacctaaataatcacaactttcaacaatggatctcttggttctg
>OTU_29_2686
ttattgtaacaatggaggtgctggggttgtcgctgacttttgaaatggtcgtgcacacctcggtgctctcatatacaatccatctcacccctcttgtgcatcaccgcgtggaggccccttttggctagttctaaagggggttttcacgtttttatacatacaccattttaatgcaatgcgtagaatgtcatacttttgcgatcacacgcaattaatacaactttcaacaacggatctcttggctctc
>OTU_30_6370
ttatcgaataaacttgaacaggctgttgctggtccaccacctccctggctgggcatgtgcacgcttgtcatctttatcatttctccaactgtgcacctcttgtagacctggggcatgtttgaaatatgacgagtttgggactgccattcggctttgccttgtattctccaggtctatgtttcacacaaacaaaaaacttgtttagaatgtgttggcaataataataaagttatacaactttcagcaacggatctcttggctctc
>OTU_32_2808
ttacagaaagtaaacgcggatcgaaccgcgaacttctaaacctttgacgattgactcgcgttgcctcggcgggttctcccgccagaggatacatcaaaactcctgttttaacggtgttgtctgagctacaagcaacgaatcaaaactttcaacaacggatctcttggttctg
>OTU_33_8095
ttaacgagttagggtctctcacggcccgacctcccaaccctttgtttatcgaacctctgttgcttcggcggatccgtccctcgggaccgccggaggatcgtcgtaaggcgtcctctggcccgtatccgtcgatagccaaccacttaaactcagaataaatcgtgttataattgtctaagtttatcataaaaattaaagcgaaaactttcaacaacggatctcttggttctg
>OTU_34_93
ttaatgattacgaacggttgccttcagtgctggctcttaggagcaagtgcacgttggtggctttcatccaacaccctgtgaacctttggcctcttgctagcttcggctggcagaggatttttataacacactcgaatgtaatgagaactattgtcgtgcgcaagcactaatgtacaactttcaacaacggatctcttggctctc
>OTU_36_1342
ttaccgagtttacaactcccaaacccctgtgaacatacctattgttgcctcggcggtgcctgttccgacagcccgccagaggaccccaaaccctgattacatttaagaagtcttctgagtaaccgattaaataaatcaaaactttcaacaacggatctcttggttctg
>OTU_37_3371
ttagtgattgtgaatcgttaccttcagtgctggctggttccagcaagtgcacgttggtgactttcatccaaatacccttgtgaacctttggcctcttgctggcttcggctgacagaggattttacacaccaactcgaatgtaatgaaaactactgtcgtgcgcaagcactaatgtacaactttcaacaacggatctcttggctctc
>OTU_38_3057
ttactgaattgttaacaagagttgttgctggtccctatacgggggcatgtgcacgctctgtttacacatccactcacacctgtgcaccctctgtagttctatggcctggggggctctgtcctcctgctgtggctctacgtctttacacacacactgtaacaaagtctcatggaatgtatgtcgcgtttaacgcaatgaaatacaactttcagcaacggatctcttggctctc
>OTU_44_835
ttaccgagtttacaactcccaaacccctgtgaacataccacttgttgcctcggcggatcagcccgctcccggtaaaacgggacggcccgccagaggacccctaaactctgtttctatatgtaacttctgagtaaaaccataaataaatcaaaactttcaacaacggatctcttggttctg
>OTU_45_839
ttactagggggcttcggccccatcaagataccaccccttgcattttgagtaccttctgtttcctcggcgggcctgcccgccaacggggaccacaccaaacccttctgtagtcgcagtaaacgtctaaaaacataatttaaatcaaaactttcaacaacggatctcttggttctg
>OTU_46_4043
ttaacgagttagggtcttctcggcccgacctcccaaccctttgtttactgaacctttgttgcttcggcggacccgtctcacgaccgccgggggaccgtaagacgtcctctggcccgtgtccgccggtggccaaaccgaacaaattctgattaaaatgtgtcaatgtctgagtagaattcataattaaaacaaaactttcaacaacggatctcttggttctg
>OTU_47_84
ttaccgaattgtcaacacgagttgttgctggtcctcaaaacgggggcatgtgcacgctctgtttacacatccactcacacctgtgcaccctctgtagttctgtggcatgggggactctgtcctcctgttatggtcctacgtctttacacacactctgtaacaaagtctcatggaatgcatgacgcgtttaacgcaatacaatacaactttcagcaacggatctcttggctctc
>OTU_49_10421
ttattgaaataaacctgatgagttgctgctggctctctagagagcatgtgcacacttgtcgtctttatatctccacctgtgcacctattgtagacctggatgactctctgaatggctatcattcaggtatgaggattgactttctgcctctccttacatttccaggcctatgttctttcatataacctcaatgtatgttatggaatgtaataattatggccttctgtgccttataaacctatacaactttcagcaacggatctcttggctctc
>OTU_51_6002
ttattgaattttgaaagaggttgtagctgatcaaaaaagatatgtgcacacctcatttccaaattttacacccatgtgcactctttgtaggccatttggcctatgtcttcataatacactcaaaaggtttcagaatgtgaaatgtatatcttaaattaatacaactttcaacaacggatctcttggctctc
>OTU_52_4558
ttatcgtacaaccgaggtgcaaaggctgtcgctgaccctcaaaggtcgtgcacgcctgagcgctctcacacaatccatctcacctttgtgcatcaccgcgtgggtcccctttgcgggagggcttgcgttttcacataaaacttgatacagtgtagaatgtttttcttttgcggtcacacgcaatcaatacaactttcaacaacggatctcttggctctc
>OTU_53_222
ttaccgagttcatgccctcacgggtagatctcccaccctatgttatcattacctttgttgctttggcgggccgccaggcttcggctaggctaccggctccggctggtaagcgcccgccagaggaccccaaactctgaatgttagtgtcgtctgagtactatctaatagttaaaactttcaacaacggatctcttggttctg
>OTU_55_4466
ttactgagttgttgacacgagctgttgctggtcctcaaagcaaggggggcatgtgcacgctctgttcacacatccactcacacctgtgcaccccccgtagttctatggtctggagggcttgccgtcttcttcccgtagttctacgtctttacacatacaccgtagtaaagtcttatggaatgtgcgccgcgtttaacgcaataaaatacaactttcagcaacggatctcttggctctc
>OTU_56_368
ttacagaaagtaaacgcggatcaaaccgcgaacttctaaacctttgacgattgacttatgttgcctcggcgggttctcccgccagaggatatatcaaaactcctgttttaacggtgctgtctgagctacaagcaacgaatcaaaactttcaacaacggatctcttggttctg
>OTU_58_5087
ttatcgtaaaaccgaggtgcgagggctgtcgctgaccttttttggtcgtgcacgcccgagcgctctcacacaatccatctcaccccttgtgcaccaccgcgtgggttccctttctggcttgtccgaaggggggctcgcgttttcacacaaacttgaattggtgtagaatgtccttttttgcgataacacgcaattaatacaactttcaacaacggatctcttggctctc
>OTU_59_454
ttacagagactctgccctttgggtagacctcccaccctgtgtcgttatacctctgttgctttggcgggccgtggggccccggccctgcccctggctccggctagggcgcgcccgccagaggacctcaaaacctgaatgttagtgtcgtctgagtactatataatagttaaaactttcaacaacggatctcttggttctg
>OTU_60_5486
ttatcgtacaaaatgtgagagaggcatgcaagggctgtcgctgactccaagtcgtgcacgccggagtgtgccctctcacataataatccatctcaccctttgtgcaccaccgcgtgggcaccctttgggatcagactgatctcggaggatgctcgcgttttcacacaaaccccccttttaaaagtgtagagtgacctcatttatgcgctaacccgcaatcaatacaactttcaacaacggatctcttggctctc
>OTU_61_28
ttactgaaaaactattttcaagttttttatatcccattgtttacttaccccgttgcttccactggacagatttcatcatgtgtgggatcttttggcttttgtgtataatacttgccaaagagtttgccagtggcaagatcttttttcaccaaaacttgattaaaacacattgtctgaatatatttcttgaatgaaacaaaactttcaacaacggatctctaggctctt
>OTU_62_650
ttacagaaagtaaacgcgggtcaaaccgtgaacttttaaacctttgacgattgactcatgttgcctcggcgggctcgcccgccagaggatacatcaaaactcctgttttaacggcgttgtctgagctacaagcaacgaatcaaaactttcaacaacggatctcttggttctg
>OTU_64_125
ttaccgaattgtcaaacacgggttgttgctggcctccaaacgggggcacgtgcacgctctgtttacacatccactcacacctgtgcaccctctgtagtcctatggttcggaagaccccgtcttccttctgtagctctacgtctttacacacacactgtagcgatgtctcatggaatgtttttatgcgtttaacgcgatacaatacaactttcagcaacggatctcttggctctc
>OTU_65_101
ttaccgaattgtcaacaagagctgttgctggtccctggatgggggcatgtgcacgctctgttaacacatccactcacaccctgtgcaccctctgtagttctatggttgggggggggacctgtcctctcctgctgtggttctgcgtctttacatacacactgtaacaaagtctcatggaatgcatgctgcgtttaacgcaataaaatacaactttcagcaacggatctcttggctctc
>OTU_66_545
ttactgaattgtcaacaagagttgttgctggtccccagatgggggcatgtgcacgctctgttaacacatccactcacaccctgtgcaccctctgtagttctatggttggggggacctgtcctctcctgccgtggttctgcatctttacatacacaccgtaacaaagtcttatggaatgcatgccgcgtttaacgcaataaaatacaactttcagcaacggatctcttggctctc
>OTU_67_80
ttactgaattgtcaacacgagttgttgctggtcctcaaatgggggcatgtgcacgctctgtttacacatccactcacacctgtgcaccctctgtagttctgtggctagggggactctgtccttctgctgtggttctatgtctttacacatactctgtattaaagtctcatggaatgtatcctgcgtttaacgcaatacaatacaactttcagcaacggatctcttggctctc
>OTU_69_896
ttaccgagtttacaactcccaaacccctgtgaacataccatttgttgcctcggcggtgcctgcttcggcagcccgccagaggacccaaacccttgattttatacagtatcttctgagtaaatgattaaataaatcaaaactttcaacaacggatctcttggttctg
>OTU_70_21
ttattgattgcgaatcgttgtctccagtgctggccaggtcactctggcaagtgcacgtcgacggctttcatccaataccctgtgaacctttggcctcttgctagcttcggcgagcagaggattttacacccactcgaatgtaatgaaattattgttgtgcacaagcactaatatacaactttcaacaacggatctcttggctctc
>OTU_71_44
ttaccgaaccgtcgacacgagttgctgctggccctcaaaaggggcatgtgcgcgctctgtttacacatccactcacacctgtgcaccctctgtagttctatggccttggggaccctgtccccttgccgtggtcctacgtctttacacacacgccgtaacaaagtctcgtggaatgtatgtcgcgtttaacgcaatacaatacaactttcagcaacggatctcttggctctc
>OTU_72_123
ttattgtacaacggaggtgcgagggctgtcgctgaccttcaaaggtcgtgcacgcccgagccctctcacaatccatctcaccctttgtgcatcaccgcgtgggtccccctttagcggggagggctcacgtttttacataaaactcgatgcagtgtagaatgtttatttttgcggtcacacgcaatcaatacaactttcaacaacggatctcttggctctc
>OTU_73_4
ttatcgaataaacttgaacaggctgttgctggtcttccttgagggggacatgtgcacgcttgtcatctttatatctccaactgtgcacatattgtagacctggagtcgtctttgttgattcatttcgagttggcactgcagcgctcacaaaacctgctttgccttttgcatttccaggtctatgtcattttcacaacctcaatgtgtttagaatgttgaatcaggtcatacgtacctataaagttaaatatacaactttcagcaacggatctcttggctctc
>OTU_74_257
ttaacgttggggactaacaatccctcagcgagatagaacccttgctttttcgagtaccacacgtttcctcggcaggtacgcctgccaatggggaccattaaaaaccttttgtaatagcagtaaacgtctaaaacaacaaaaatttaaaactttcaacaacggatctcttggttctg
>OTU_75_7670
ttaccgagtgagggccccgtgcccgacctccaaccctttgaagttaaccaactctgttgcctcgggggcgacccggtcgtcgcgccggcgcccccggaggaccacacaccctgcgttttgtgcgtcggagttttaagcgaaatcttaatcaaaactttcaacaacggatctcttggttctg
>OTU_76_2567
ttaccgaactgtcgacacgagttgttgctggtcctcaaacgggggggcatgtgcacactctgtttgcatatccactcacacctgtgcaccctccatagttctatagccctgggggccttaaacccccagctgtggtcctatgtatttacacatacactgtaataaagtcttatggaatgtatgccgcgtttaacgcaatacaatacaactttcagcaacggatctcttggctctc
>OTU_78_316
ttatcgagtaccgaggcccccgcgcctctgctcccaccctgtgttgacctgcaatgttgctttggcgggccggcgggtacacccgccgccgggccgcgcctggagagcgcccgccagagaaccacgcgaaactctttgcaactttgagcgtctgagcactttgagaattgaccaaaactttcaacaacggatctcttggttctg
>OTU_79_8817
ttaccgaattgtcaacacgagctgttgctggtcctcaaacgggggcatgtgcacgctctgtttacacatccacttacacctgtgcacccttcatagttctgtggcctgggggcaccgtcccccctgctgtggtcctatggatttaacacacacacactgtaaccaagtctcatggaatgtacaccgcgtttaacgcaatacaatacaactttcagcaacggatctcttggctctc
>OTU_81_8756
ttactgaactgtcgacacgagttgttgctggtcctcaaatgggggcatgtgcacgctctgtttgcatatccactcacacctgtgcaccctatgtagttctatggcttgggggaccctgtcctcctgctgtggttctatatctttacacacactctgcaataaagtcttatggaatgtataccgcgtttaacgcaatacaatacaactttcagcaacggatctcttggctctc
>OTU_82_1541
ttactgagtgcgggccctctgggtccaacctcccacccgtgtttatcgtaccttgttgcttcggcgggcctccgtgcccgccggagacacctttgaacgctgtctgaaggttgcagtctgagtcgattttttaaatcattaaaactttcaacaacggatctcttggttccg
>OTU_83_11
ttattgaaataaacctgatgagttgttgctggctctctctctagagagcattgtgcacacttgtcatctttatatctccacctgtgcacattttgtagacctggatcattctctgaaatgctaacccagcattttaggttggaggaatttgactttgttgtctttccttgcatttctaggtctatgttctttttcatataatcttattgtgatataatggaccctatataatataaacctatacaactttcagcaacggatctcttggctctc
>OTU_85_1108
ttactgaactgtcgacacgagttgttgctggtcctcgagagggggcatgtgcacactctgtttacacacccactcacacctgtgcaccctctgtagctctgtggtgtgggggccctgtcctcccactgtggttctacgtctttacacacacacagtgataagtctcatggaatgtatgtagcgtttaacgcaatacaatacaactttcagcaacggatctcttggctctc
>OTU_86_16
ttaatgaactcaattcagtaccttggagaactgttaaggcctagtgccgttaaagacggtcccgtggtggtgaatttcattaccatcccttctgtgcacatatctcatgttgcttccgtgcgtctgcagtccatctgtatggacatgtctgaaaagccttggtggttccgagggcgcacggggacgaaaaactgtactctggtttctgtatttcatcgtctgagtggtaaccacattgtaaccaatcaaaactttcaacaacggatctcttggttctc
>OTU_87_2104
ttaccgagtttacaactcccaaacccaatgtgaaccataccaaactgttgcctcggcggggtcacgccccgggtgcgtcgcagccccggaaccaggcgcccgccggagggaccaaccaaactcttttctgtagtcccctcgcggacgttatttcttacagctctgagcaaaaattcaaaatgaatcaaaactttcaacaacggatctcttggttctg
>OTU_88_1706
ttaccgagttcatgcccttacgggtagatctcccaccctatgttatcattacctttgttgctttggcgggccgccaggctccggtcaggctatcggcttcggctggtacgcgcccgccagaggaccctaacattctgattatcagtgtcgtctgagtactatataataatagttaaaactttcaacaacggatctcttggttctg
>OTU_90_19
ttatcgtataacagaggtgtaagggctgtcgctgacctttaaaggttgtgcacgcctaagccctctcacacaatccatctcaccccttttgtgcatcaccgcgtgggtcctccctttgccgggagggcctgcgtttttatataaaacttgacacaatgtagaatgttttctttttttgcaattatacgcaaatcaatacaactttcaacaacggatctcttggctctc
>OTU_91_57
ttaatgattgcgaatcgttgccttctgtgctggccagccactggcaagtgcacgtcggtgactttcatccaataccctgtgaacctttggcctcttgctagctttggccggcagaggaattttacacacactcgcatgtaatgaaatatactgtcgtgcgcaagcactaatgtacaactttcaacaacggatctcttggctctc
>OTU_92_1868
ttatcgtacaatggaggtgctggggttgtcgctgacctttgaaagggtcgtgcacgcctcggtgctttcgctttcacacacaatccatctcaccccttttgtgcatcaccgcgtgggggtcccttttagctagttctgaagggggctttcgcgtttttacaaacacacccttttaatgcaatatgtagaatgtcttactttttgcgatcacacgcaatcaatacaactttcaacaacggatctcttggctctc
>OTU_93_5147
ttatcatacaaccgaggtgcgagggctgtcgctgaccccgtagaaaggtcgtgcacgcccaagtgctctcacacaatccatcacacccccttgtgcatcaccgcgtggggtccccctttgccgggagaacctgcgtttccacattaaactcgataaagtgtagaatgtttatttttgcgatatcacacgcaatcagtacaactttcaacaacggatctcttggctctc
>OTU_94_14
ttaatgattgcgaatggttgccttcggtgctggctcttgcaagtgcacgttggtggctttcatccaatacccctgtgaacctttggcctcttgctagcttcggctggcagaggatttttacacactcgaatgtaatgaaaactattgtcgtgcgcaagcactaatgtacaactttcaacaacggatctcttggctctc
>OTU_95_839
ttacagagactctgccctttgggtagacctcccaccctgtgtcgttatacctttgttgctttggcgggccgcggggcttaggccctgcccctggctccggctagggcgcgcccgccagaggacctcaaaacctgaatgttagtgtcgtctgagtactatataatagttaaaactttcaacaacggatctcttggttctg
>OTU_97_1604
ttaacgagttagggtctcctcggcccgacctcccaaccctatgtttactgaacctttgttgcttcggcggacccgttcttacgaccgccgggggaccgtaagacgtcctctggcccgtgtccgccggtggcccaactgaacaaattctgattaaaatgtgtcaatgtctgagtagaattcataattaaaacaaaactttcaacaacggatctcttggttctg
>OTU_98_6028
ttattgaaataaacctgatgggttgttgctggctctctagggagcatgtgcacaccttgtcatctttatatctccacctgtgcactttttgtagacctttcaggtctatgttgcttcatttaccccaatgtatgttaatagaatgttgtgcctatataatatatacaactttcagcaacggatctcttggctctc
>OTU_100_1564
ttattgtaaaaccgaggtgcaagggctgtcgctgaccttccacggtcgtgcacgcccgagcgctctcaccacaatccatctcacccctttgtgcatcaccgcgtgggtccccctttgcaggagggctcgcgttttcacataaaacttgacacagtctagaatgatcttttttgcggtaacacgcaatcaatacaactttcaacaacggatctcttggctctc
>OTU_101_11
ttaatgagttgtgatggggttttgatgctggtatccgattttttttggatgcatgtgctcgctctaccaatcatcttccaaacacccgtgcacatttttgagggattttcgagttgatcgccccccccactgtttgggtgattttcttgcattccccttaaaatccattatatacgccgttaacaatgctgaacgtgctttgtgccgcaaggcctttaatataatacaacttttaacaacggatctcttggctctc
>OTU_102_49
ttacagagttcatgcccttcggggtagatctcccacccttgtgtatcattatagaatgttgctttggcgggccgcgtgcctagcacgcctcgattcgcgtcgagcgtgtgcccgccagaggacccctaaactctgaatgttaatgtcgtctgaagtactattcaatagttaaaactttcaacaacggatctcttggttctg
>OTU_103_237
ttagcgagtatggccctcacgggtctaatccaccataaacacctttgtgaaccagtcagggggactcggccgtgaaagcccaggcgattagcggaagggccctcctttgcaaacccgtttagaagtcttgaaagtacttttgtataatctagtaaaacttatagcaacggatctcttggctctc
>OTU_104_6842
ttaccgaacgtcgacacgagttgttgctggtcctcgaatgggggcatgtgcacgctctgtttacacatccactcacacctgtgcaccctccgtagttctatggccttgggggcctctgtcccctttgcccacggttctacgtctttacacatacaccgtaacaaagtctcatggaatgtatgcagcgtttaacgcaatacaatacaactttcagcaacggatctcttggctctc
>OTU_105_1846
ttacagagttcatgccctccgggtagatctcccacccattgctatcactactctcgttgctttggcgggccgctgggccctgcccggccgccggccccggctggcgcgcgcccgccagagacctcacagactctgaatgttagtgtcgtccgagtaactatataatcgttaaaactttcaacaacggatctcttggttctg
>OTU_106_911
ttaccgagtgcgggtcccctcgcggggcccaacctcccacccgtgtctaccgtcacctgttgcttcggcgggcccgccttcgtgccgccggggggccctctgcgcccccgggcccgcgcccgccgaagaccctaggaacactggatgaaggatgccgtctgagtcaacgacacaatcgttaaaactttcaacaacggatctcttggttccg
>OTU_108_1287
ttaccgagttagggtcttctcaggcccgacctccttacctttgtctaccttacctcacgttgcttcggcgggcccgtcctcttttggaccgccggagggttcaccccctctggcccgttgcccgccgacagccccccaaccaaaaaactcttgcaataactgtgaaattgtctgaattttgcttctaaaataaccaaaaactttcaacaacggatctcttggttctg
>OTU_110_4
ttattgaaataaatctgatgagttgttgctggctctctctctctagagagcattgtgcacacttgtcatctttatatctccacctgtgcacattttgtagacctggatcactctctgaaatgctaaccccagcatttgaggttggaggaatttgactttgttgtctttccttgcatttccaggtctatgttctttttcatataatcttattgtgatataatggaccctatataatataaacctatacaactttcagcaacggatctcttggctctc
>OTU_111_9
ttattgattgcgaatcgttgtcttcagtgctggccagctctggcaagtgcacgttggccgcttttcatccaataccctgtgaacctttggcctcttgctggccttggccggcagaggaatttttacacccactcgaatgtaatgaaatattgttgtgcgtaagcactaatgtacaactttcaacaacggatctcttggctctc
>OTU_112_657
ttaaagagtaagggtcttctaggcccgatctcccaaccctttgtttattgaacctctgttgcttcggcggatccgtctcacggccgccggaggaccgctgaaaggcgtcctctggccagcatccgccgatagccaaccacttaaactctgaataaatcgtgtcatatgtctaagtctatgattaaattaaagcaaaactttcaacaacggatctcttggttctg
>OTU_115_2235
ttaatgattgcgaatcgtcgccttcagtgctggccggcttcggcaagtgcacgtcggtgactttcatccaataccctgtgcacctttggcctcttgctagcttcggccggcagaggatttttacacacactcgaatgtaatgaaatttattgtcgtgcgcaagcactaatgtacaactttcaacaacggatctcttggctctc
>OTU_116_28
ttattgaatgaacttggtgtggttgtcgctggccctctcgggggtatgtgcacgcccatcatcctcatctctccacctgtgcacctcttgtagacttggattgactttccgaggtaactcggtcgggaggactgctagcaattagctggctttccttgcatttccaagcctatgtttttcatataccccattgtatgtaacagaatgtatcattgggcccttgtgcctataaaacctatacaactttcagcaacggatctcttggctctc
>OTU_118_2
ttatcgtactacagaggcggcgagggctgtcgctgaccttttcaaaaggtcgtgcacgcccgagcgttctcatctacaatccacctcaccccttgtgcatcaccgcgcggggcccctccctcttcaggggagaaggctcgcgtttttcacacaaacgtccttatagtttagaatgtcattcatttgcgatcacacgcaatcaatacaactttcaacaacggatctcttggctctc
>OTU_120_15
ttactgaactgtcgacacgagttgttgctggtcctcgagagggggcatgtgcacactctgtttacacacccactcacacctgtgcaccctctgtagctctgtggtgtgggggccctgtcctcccactgtggttctacgtctttacacacacacacagtgataagtctcatggaatgtatgtagcgtttaacgcaatacaatacaactttcagcaacggatctcttggctctc
>OTU_123_47
ttattgtataaccgaggtgctagggctgtcgctgaccctttgaagggtcgtgcacgcccaagtgctctctcacatccatctcacccctttgtgcatcaccgcgtgggctacctttttggctttattcaaaaaggttggttcgcgtttttacacacacacctttatgtatagaatgtcttgatttttgcggtcatacgcaataaataaataaataatacaactttcaacaacggatctcttggctctc
>OTU_124_1635
ttacagaaagtaaacgcggatcaaaccgcgaacttctaaacctttgacgattgactcatgttgcctcggcgggtcctcccgccagaggatacatcaaaactcctgttttaacggtgttgtctgagctacaagcaacgaatcaaaactttcaacaacggatctcttggttctg
>OTU_126_1034
ttaccgagtgcgggtcccctcgcggggcccaacctcccacccgtgtctaccgtcacctgttgcttcggcgggcccgccttcgtgccgccggggggccctctgcgcccccgggcccgcgcccgccgaggactctaggaacactgaatgaaggatgccgtctgagtcaacgacacaatcgttaaaactttcaacaacggatctcttggttccg
>OTU_127_27
ttattgattttgatttgttaccttctgtgctggctccggcatgtgcacgttggtgactttcatacaatacccttttgtgaacctttggcctcatgccggctttggctggcagaggatttacacaaacttgaatgtaacagaaagaaattgttgtgcgcaagcacgaatgtacaactttcaacaacggatctcttggctctc
>OTU_128_22
ttactgaactgtcgacacgagttgttgctggtcctcgaaagggggcatgtgcacgctctgtttacacatccactcacacctgtgcaccctctgtagttctgtggcattggggactctgtcctcttgccgtggtcctacgtctttacacacacaccgtaatacagtctcatggaatgtatgtcgcgtttaacgcaatacaatacaactttcagcaacggatctcttggctctc
>OTU_129_61
ttattgattgcgaatcgttgtctccagtgctggccaggtcactctggcaagtgcacgtcgacagctttcatccaataccctgtgaacctttggcctcttgctagcttcggcgagcagaggattttacacccactcgaatgtaatgaaattattgttgtgcacaagcactactatacaactttcaacaacggatctcttggctctc
>OTU_130_747
ttaccgagttagggtcttctaagggcccgacctccttacctttgtctaccttacctcacgttgcttcggcgggcccgtcctcttttggaccgccggagggttgaacccctctggcccgttgcccgccgacagcccccaaccaaaaaactcttgcaataactgtggaattgtctgaatcttgcttctaaaataaccaaaaactttcaacaacggatctcttggttctg
>OTU_131_3
ttaccgaattgtcgacacgagttgttgctggtcctcaaatgggggcatgtgcacgctctgtttacacatccactcacacctgtgcaccctctgtagttctgtgacatgggggactctgtcctcctgttgtggctctacgtctttacacacactctgtaacaaagtatcatggaatgtatgccgcgtttaacgcaatacaatacaactttcagcaacggatctcttggctctc
>OTU_132_3920
ttatcgtacaaccgaggtgcaaaggctgtcgctgaccctcaaaggtcgtgcacgcccgagcgctctcgcacaatccatctcacctttgtgcatcaccgcgtgggtcccctttgcgggagggcttgcgttttcacataaaacttgatacagtgtagaatgtttttcttttgcggtcacacgcaatcaatacaactttcaacaacggatctcttggctctc
>OTU_133_261
ttcataataagtgttttatggcactttttaaatccatatccaccttgtgtgcaatgtcagtcgatcttcttcatggagatcgaccaaacatcaacctttattttttaactctttgtctgaaaaatattatgaataaacaattcaaaatacaactttcaacaacggatctcttggctctc
>OTU_137_499
ttattgactgtgaatcgttgcctccagtgctggctccggcaagtgcacgttggtgactttcatccaacaccctgtgaacctttggcctcttgctcgcttcggctggcagaggatttttacacccactcgaatgtaatgaaattattgtcgtgcgcaagcactaatgtacaactttcaacaacggatctcttggctctc
>OTU_138_4601
ttaccatacaacgaggggggcccgagggctgtcgctgatcccttccgaggggtcgtgcacgcccttgacgtcgtcctcacgcaatcaatccatctcaccttttgtgcatcaccgcgtgggcctcgaaaggggcctgtgcctttttcacacacactcgtacgattcagtctagaatgtctttgcctttgtatgcaatcaatacaactttcaacaacggatctcttggctctc
>OTU_139_1888
ttacagaaagtaaacgcggatcaatccgcgaacttctaaacctttggcgattgactcatgttgcctcggcgggttctctcgccagaggatacatcaaaactcctgttttaacggtgttgtctgagctacaagcaacgaatcaaaactttcaacaacggatctcttggctctc
>OTU_141_277
ttactgagaatgctctccggagccctcaaatcttgcgtacccatctcgtacttgcgggttgcccccgggcgcccgcgaggattcaaactgtttgactgtcgtctgattcttacaaagaaaacaactttcaacaatggatctcttggctccg
>OTU_142_136
ttaccgagtttacaactcccaaacccctgtgaacatacctatttgttgcctcggcggtgcctgttccgacagcccgccagaggaccccaaaccctgattacatttaagaagtcttctgagtaaaccgattaaataaatcaaaactttcaacaacggatctcttggttctg
>OTU_143_15
ttactgaactgttgacacgagctgttgctggtcctcaaatgagggggcatgtgcacgctctgtttacacatccactcacacctgtgcaccctctgtagttctacatggcctgggggacacgctgtcttcctcgtgtagctctacgtctttacacatacaccgtacaaagtcttatggaatgtgcaccgcgttcaacgcaatacaatacaactttcagcaacggatctcttggctctc
>OTU_145_387
ttaatgattgcgaatgggtcaccttcagtgctggctctcaagagcaagtgcacgttggtggctttcatccaattccacaccctgtgaacctttggcctcttgctagcttcggctggcagaggattttacatactcgaatgtaatgaaaactcttgttgtgcgcaagcactaatatacaactttcaacaacggatctcttggctctc
>OTU_146_8
ttaccgaattgtcaacaagagttgttgctggtccctgcataggggcatgtgcacactctgtttacacatccactcacacctgtgcaccctctgtagttctgtggcctggggggctctgtcctcctgctgtggttctacgtctttacacacacacactgtaacaaagtctcgtggaatgtatgtcgcgtttaacgcaaagaaatacaactttcagcaacggatctcttggctctc
>OTU_147_708
ttagtgaacgccctcacgggcttataactattccaaacctctgtgaaccgtgcccttcggggctattttacaaacatggtgtaatgaacgtcatatatcataacaaaacaaaactttcaacaacggatctcttggctctc
>OTU_148_857
ttaccgaattgtcaacacgagttgttgctggtcctcaaacggggacatgtgcacgctctgtttacacatccactcacacctgtgcaccctccatagttctgcagcctgggggctctgtccccctgatgcggttctatgtatttacacacacacactgtagtaaagtctcatggaatgcataccgcgtttaacgcaatataatacaactttcagcaacggatctcttggctctc
>OTU_149_5
ttaatgattgcgaatggttgccttcagtgctggccggcttcggctttggcaagtgcacgtcggcgactttcatccaataccctgtgaacctttggcctctgctagcttcggccggcagaggaattttacacaaactcgaatgtaatgaaatttattgtcgtgcgcaagcactaatgtacaactttcaacaacggatctcttggctctc
>OTU_151_15
ttaaaagtaatatatattcattcgaatatacatttctaaccccttgtctaaaccacctgttgcttccggctgtttgggctgtatagttagctgtatagcctggccggaaggttaacataaaatattgtatttagagtcttctgaattttaaaataaaagttaaaactttcaacaacggatctcttggttctc
>OTU_152_9
ttaccgaattgtcaacacgagttgttgctggtccccgaatgggggcatgtgcacgctctgtttgtacatccactcacacctgtgcaccctccatagttctgcagcctgggggctctgtcccccctgctgtggccctatgtatttacacatacacactgtaatgaagtctcatggaatgtgtgacgcgtttaacgcaatacaatacaactttcagcaacggatctcttggctctc
>OTU_154_601
ttcataataagtgttttatggcactttttaaatccatatccaccttgtgtgcaatgtcagtcgatcttcttcatggagatcgaccaaacatcaaccttatcttttaactctttgtctgaaaaatattatgaataaacaattcaaaatacaactttcaacaacggatctcttggctctc
>OTU_155_3
ttatcgtacaacggaggtgcaagggctgtcgctgacctttcaaaggtcgtgcacgtccgagtgctctctcacacaatccatctcacaccccctttgtgcatcaccgcgtgggtccccgcatttttttgtggaagggctcgcgttttcacataaaactcgatacagtgtagaatgtcttttttcttcttttgcggtcacacgcaatcaatacaactttcaacaacggatctcttggctctc
>OTU_158_871
ttattgaaataaacctgatgggttgttgctggttctctagggagcatgtgcacgccttgtcatctttatacctccacctgtgcactttttgtagaccttttaggtctatgttgcttcatttaccccaatgtatgttaatagaatgttgtgcctatgtaataaatacaactttcagcaacggatctcttggctctc
>OTU_159_72
ttacctggccttgggccgctcgcgggagctagtcgcttgcgacgacgctgccgagggtgcttagcccttgacttatcaccttgactatgtgcaccttttgttgtttcctcggcaggtcatctgccgccaggaaccctctaaacctttttgcaaacagcgtctaaacttctgaaaacaaaccaaattatttacaacttttaacaatggatctcttggttctg
>OTU_160_3367
ttactgattgcgaatcgttgccttcagtgctggccggcttcggcaagtgcacgttggtgactttcatccaataccctgtgaacctttggcctcttgctagcttcggccggcagaggatttttacacacactcgaatgtaatgaaatttattgtcgtgcgcaagcactaatgtacaactttcaacaacggatctcttggctctc
>OTU_161_15
ttaaagatttcgacctttgggtcaatctctcttcagctgtgcgctttggctgcacgctgttgaaacctcccacacctgtgcaccattcggttgcggcctaggtcgcttccgcttttacacatacaactacagtctagaatgttaaatactataacaaataacaactttcaacaacggatctcttggctctc
>OTU_162_12
ttatcgtatcaagtgtgtgaggcatgcgagggctgtcgctgaccctttggtcgtgcacgccggagtgtgtcctctcacataacaatccatctcaccctttgtgcaccaccgcgtgggcaccctttgggatcacacccatctcagatgggggctcacgttttcacacaaaaacccccttttaaaaagtgtagagaatgtcctaatttatgcaatcaatacaactttcaacaacggatctcttggctctc
>OTU_163_165
ttactgaaaaacttctctagttttttatatcccattgtttacctaccatgttgcttccactggacatgttttaccttgtgtaagaccttttggcttgtgtgaataacccaagtctgggagttgccagtggcaagactatttaccaaaacttgatcaaaacatattgtctgaatttttttgcttcaataaaataaaactttcaacaacggatctctaggctctt
>OTU_164_3619
ttatcgattccattctgaagaggtagagagactgtggctggccgtctatatacctatataagaccgcatgtgcacgtcccctcgccttttctatctacacacacctgtgcacctattgtagatccccatccccttttgcctttgaaagagggagggaacctatgtttttcatcacatcacacgcaattgtatgtctagaatgtaatttcattgatcattgatcattgataaaaatataatacaactttcagcaacggatctcttggctctc
>OTU_165_11
ttaatgatgtgaaattgtcttaccttcagtgctggctcttgcaagagcatgtgcacgttgggggacatatatccaacaccctcttgtgaaccattggcctttgctggcttcggctggtagaggatttacacaaacacgaatgtaatgaaataatgttgtgctgagcactaatatacaactttcaacaacggatctcttggctctc
>OTU_166_19
ttaatgattgcgaacggctgccttcagtgctggctcttaggagcaagtgcacgttggtggctttcatccaacaccctgtgaacctttggcctcttgctagcttcggctggcagaggatttttataacacactcgaatgtaatgagaactattgtcgtgcgcaagcactaatgtacaactttcaacaacggatctcttggctctc
>OTU_167_3
ttagtgattttgaactgttaccctcagtgctggctggtctccagcaagtgcacgttggtgactttcatccaaatacccctgtgcacctttggcctcttgctggcttcggctggcagaggattttacaccaactcgaatgtaatgaaaactactgttgtgcgcaagcactaatgtacaactttcaacaacggatctcttggctctc
>OTU_169_7
ttattgaataaacatttgataggctgttgctggcccttctagggtagtgtgcacgcttgtcatttatttatttatttccacctgtgcacctttttgtagaccttagagaagatttctatgcctttcaaatgaagtgcaaaagtctctctaggttctatgttatactcacatactcctatgaatgtaatagaatgttgatctgggtctttgatgccccataaagttatatacaactttcagcaatggatctcttggctctc
>OTU_170_204
ttaaagagtaccggagctctcgggttcctactcccaccctatgttgactttgaatgttgctttggcggaccggcagtattgccaccggcctaggctggatagcgtccgccagaggatttttaaattcctttaaaaggtgaattctgagtctttgaaaattgaatcaaaactttcaacaacggatctcttggttctg
>OTU_171_232
ttagtgattatgaaaggttgccctctgtgctggctccggcaggtgcacgatggtgactttcatccaacacccctgtgaacctttggcctcttgctggcttcggctggctgaggatttacacaaactcgcatgtattgaggacccttcatgtgcgcaagcactactatatacaactttcaacaacggatctcttggctctc
>OTU_172_1
ttaacgagttagggtcttctcggcccgacctcccaaccctgtgtttactgaacctttgttgcttcggcgggcccgtctcacgaccgccgggggaccgtaagacgtcctctggtccgtgtccgccggtggccaaactgaacaaattctgattaaaaacgtgtcaatgtctgagtacaattcataattaaaacaaaactttcaacaacggatctcttggttctg
>OTU_173_91
ttaccgagttagggtagtcactcactgcccgacctcccaaccctgtgtctaccacactttgtcgttgcttcggcggaccggttgaccaactggtcatgaccgccgggggctggctctgcccccctggagagcgtccgccgatggcccaaccacaaaactcttgtaccgaaacgtgtcgtctgaatcatgtttgagaaatcaaaaacaaaactttcaacaacggatctcttggttctg
>OTU_176_11
ttacaggactcgcaagactccttaaacccctgtgaacttactgtttatacgttgcttcggcgggtgctccggggtccgccccggggcgctgcgcccgccggcagcctacttaattctgtttctctgcgttggcatctcgagtaagcaaaataagttaaaactttcaacaacggatctcttggttctg
>OTU_177_139
ttaccgagtgagggccctctgggtccaacctcccacccgtgtttatcgtaccttgttgcttcggcgggcccgcctcacggccgccggggggcttctgccctctggcccgcgcccgccgaagacacccattgaacgctgtctgaagattgcagtctgagcaattagctaaataagttaaaactttcaacaacggatctcttggttccg
>OTU_179_12
ttactgggtggcttcggcctaaaccccctaaccattgtgaacgataccagttgcttagctggtggcggtgaaaatcgcagtaccggcggcacaaaactcttttaatgttatctctgagttacttttaaataagtcaaaactttcaacaacggatctcttggttctg
>OTU_180_33
ttattggaatcgcaagaggctgtagctctggcccccttgggtatatgtgcacgtctcttgttcctttatcacccacctgtgcactgattgtagaccccttgtgtctatgatatacattatacagtttttgaatgtattatgttggctttataaacaatatatataactttcaacaatggatctcttggctctc
>OTU_182_154
ttatcgtacaaaatgtgaggggcatgcaagggctgtcgctgactgaatgtcgtgcacgcccgggtgtgtttcctcacataataatccatctcaccctttgtgcatcaccgcgtgggcaccctttgggatcatctcggagggggctcgcgttttcacacaaacaccccttttaaaaagtgtagaatgacctcatttatgcgctaacccgcaatcaatacaactttcaacaacggatctcttggctctc
>OTU_183_54
ttactgagttgtcgacatgagctgttgctggtcctcaaaacaagggggcacgtgcacgctctgttcacacatccactcacacctgtgcacccaccgtagttctatggtccagaggacttatcgtcctcttcccgtggttctacgtctttacatatacaccttagtgaagtcttatggaatgtgcgccgcgtttaacgcaatacaatacaactttcagcaacggatctcttggctctc
>OTU_184_78
ttattgaaataaacctgatgagttgctgctggctctctagagagcatgtgcacgcttgtcgtctttatatctccacctgtgcacctattgtagtcctggatgactctctgaatggctatcattcaggtatgaggattgactttctgcctctccttacatttccaggcctatgttctttcatataacctcaatgtatgttatggaatgtaataattatggccttctgtgccttataaacctatacaactttcagcaacggatctcttggctctc
>OTU_187_223
ttactgagttagggtcttccaggcccgacctccaaccctctgcctaccttacctcttgttgcttcggccggcccgtcccccctagaaataggggtcgaccgccggagggctcacaccctctggtccgcgcccgccgatggccctcaaaccaaaactcttgttcaatcgtgaattgtctgagtatacaaaacaaaataaaccaaaaactttcaacaacggatctcttggttctg
>OTU_192_34
ttatcgaaacagaacgcccgtgggggaagggggagcgccgacggccggagactgtcgcacggcctcgtgcacgtcactcgccgtcgctccttccgaccctttgaggcttcgaccttctcaacacccgtgcacccactgtaggtccttcgggatctacgtccttcttcgaactcgcatgtctacagaacgtattcgtcgtgtcccggcctcgaccctcagggtcccgcgtcggcgaccgcaaaacctataatacaactttcagcaacggatctcttggctctc
>OTU_193_20
ttaccgaaccgtcgacacgagctgttgctggccctcgaaaggggacatgtgcacgctctgtctacacatccactcacacctgtgcaccctctgtagttccatggcctcggggaccccgtccccttgctgtggtcctacgtatttacacacacacagtaacaaagtctcatggaatgcagtcgcgtttaacgcaatacaatacaactttcagcaacggatctcttggctctc
>OTU_195_695
ttactgaatggccttcgggccttcaaccttgcaaacctgtggaagcaagatgtgcttcggcgcttcggcgccgcttttatgccttaatcagtctatgtgtctgaatcaaatacaagaaataaaactttcaacaaaggatctcttggctctc
>OTU_198_69
ttaccgagttagggtagtattcactgcccgacctcccaaccctgtgtctaccacactttatcgttgcttcggcggaccggttgaccaactggtcatgaccgccgggggctggctctgtcccccctggagagcgtccgccgatggcccaaccacaaaactctttgtaccgaaacgtgtcgtctgaattattgagaaatcaaaaacaaaactttcaacaacggatctcttggttctg
>OTU_203_220
ttactgagttagggtcttccaggcccgacctccaaccctttgcctaccttacctcttgttgcttcggccggcccgtcccccctagaaataggggtcgaccgccggagggctcacaccctctggcccgcgcccgccgatggccctcaaccaaaactcttgttcaatcgtgaattgtctgagtatacaaaacaaaataaaccaaaaactttcaacaacggatctcttggttctg
>OTU_205_40
ttactgaactgtcaacacgagttgttgctggtcctcaaatgggggcatgtgcacgctctgtttacatacccactcacacctgtgcaccctctgtagttctgtggtgtgggggactctgtcctcccactgtggttctacgtctttacacacacacagtctcatagaatgtatgtcgcgtttaacgcaatacaatacaactttcagcaacggatctcttggctctc
>OTU_207_7
ttaatgattgcgaatcgtcgccttcagtgctggccggcttcggcaagtgcacgtcggtgactttcatccaataccccctgtgcacctttggcctcttgctagcttcggcctgcagaggattttacacacactcgaatgtaatgaaatttattgtcgtgcgcaagcactaatgtacaactttcaacaacggatctcttggctctc
>OTU_208_20
ttaccgaatcgtcaaacacgggttgttgctggcctccaaacgggggcacgtgcacgctctgtttacgcatccactcacacctgtgcaccctctgtagtcctatggttcggaagaccccgtcttccttctgtagctctacgtctttacacacacactgtagcgatgtctcatggaatgtttttatgcgtttaatgcgatacaatacaactttcagcaacggatctcttggctctc
>OTU_209_21
ttaccgaattgtcaacacgggttgttgctggtcctcatatgggggcatgtgcacgctctgttcacacatccactcacacctgtgcaccctccgtagttctgtggcatgggggactctgtcctcttgccgtggttctacatctttacacacacgctgtaataaagtcttatggaatgcatatcgcgtttaacgcaatacaatacaactttcagcaacggatctcttggctctc
>OTU_211_2591
ttaacgaataaacttgatcaggctgttgctggcccccttgcaggggtatgtgcacgcttgtcatatttatcatttctccaactgtgcacatattgtagacctggatcttttttgaagaaattcaagttgggggactgctgtgctctttgtttttttttcaagagtcggctttccctcgtatttttcaggtctatgtcattttcacaacctctagaatgtgtttagaatgttgaatcatatataaagttaatatacaactttcagcaacggatctcttggctctc
>OTU_215_330
ttaccgaattgtcaacaagagttgttgctggtcctcaaatgggggcatgtgcacgctctgttcacacatccactcacaccctgtgcaccctctgtagttctatggtcagggggcctgtcctcctgctgtggttctgcatctttacacacacacactgtaacaaagtctcatggaatgcatgccgcgtttaacgcaatataatacaactttcagcaacggatctcttggctctc
>OTU_216_19
ttaccgaattccaaggagcataggaggggggatgaccaccagtatgccggagtgctgtcgctggccttcgggcaatgtgcacgtctcccgagtccgacggtatcccctgccgaagctccgatacctctcgaacacccgtgtgcacctgttgtaggtctcgtcagtgggacctatgtattccattataaactcgcatgtatacagaacgttgtctagtcataatataactttcagcaacggatctcttggctctc
>OTU_218_70
ttacaaatatctgggttgtcctatcgggctcccatgcaaaacacattcatgtgtattcttccatattttaaacttttgaatcaatttagttgtctgagaaggccatgtgccataaaatttaaatacttcaaaactttcaacaacggatctcttggctctc
>OTU_224_4
ttactgaagcagttagggttgtagctgactctgtcaaaggagtactgtgctcgctctattcttctacttttccccctgtgcaccttttgtaggctatgatatccatcgtgtgtgagttcgcgctcattaccggttcaagggattgctggtttactccggctgctcttgcccttcatatgccttatgtctttatacactctttacaagtctagaatgtcttttgtgggtctattgacctataaacttaatacaactttcaacaacggatctcttggctctc
>OTU_227_1
ttattgaataaacttggttgggttgctgctggctttttaggagcatgtgcacactcactccattttaaccacctgtgcacatactgtaggcctgaatggaactgctcgaggaaactcggttggagaattgctgtgaaaatcagctgttcttgcatttcaggtctatgtttttaaacatatactccataacaagttatagaatgttaatcatgggccttctgtgcctttaaaatgtaatacaactttcaacaacggatctcttggctctc
>OTU_228_8
ttatcgtaaaaccgaggtgcgagggctgtcgctgaccttttttttttggtcgtgcacgcccgagcgctctcacacaatccatctcaccccttgtgcaccaccgcgtgggttccctttctggcttgtccgaaggggggctcgcgttttcacacaaacttgaattagtgtagaatgtccttttttgcgataacacgcaattaatacaactttcaacaacggatctcttggctctc
>OTU_230_66
ttactgaactgtcgacacgagttgttgctggtcctcgaaagggggcatgtgcacgctctgtttacacatccactcacacctgtgcaccctctgtagttctgtggccttggggactcccgtcctcttgctgcggtcctacgtctttacacacacaccgtaatacagtctcatggaatgtatgtcgcgtttaacgcaatacaattacaactttcagcaacggatctcttggctctc
>OTU_231_1
ttattgaatgaatttggtgtggttgttgctggccttcttggaggcatgtgcacacctaccatcctcatctctccacctgtgcacctcttgtagatttggaattgattatccgaggcaactcggtcgtgaggactgctagcatttagctggctttctttgcaattccaggtctatgtttttcatataccccattgtatggaacagaatgtatcattgggccttgtgcctataaaccttatacaactttcagcaacggatctcttggctctc
>OTU_232_3
ttacagagttgcaaaactcccaaaccattgtggacgatacccgtaccgttgcttcggcgggccggctcccggagccgcagcccctcctcgggggggcggcccgccggacgacatcaaaactctttgtcttatacaggcctctctgagaacttatacaaatgagtcaaaactttcaacaacggatctcttggttctg
>OTU_233_1124
ttattgaaataaacctgatgggttgttgctggttctctagggagcatgtgcacaccttgtcatctttatatctccacctgtgcaccttttgtagacctttcaggtctatgttgcttcatttaccccaatgtatgttaatagaatgttgtgcctatataatatatacaactttcagcaacggatctcttggctctc
>OTU_234_16
ttaaagagtaccggagctctcgggttcctacttccaccctatgttgactttaaatgttgctttggcggaccggcgatctcgccaccggcctaggctggacagcgtctgccggaggatttttaaattcctttgactgtgaactccgagtctttgaaaattgaatcaaaactttcaacaacggatctcttggttctg
>OTU_235_4
ttacagagttgaaaaactcccaaaccattgtgaacgtcaccgcatcgttgcttcggcgggcggctctcaacaggagccgcgcctccccctcggggtgaggcagcccgccggaggcgtcaaaactctcgatatcatatggcatctctgagtaacttgcaaaataagttaaaactttcaacaacggatctcttggttctg
>OTU_236_1811
ttaacgaatggtcatccttgatcttcttaccctcaaaccactgtggacacacaagatgtgcttcggcgtcgagaggcgccgccttcatgcttcatcaagtctttcttgtctgtaaaaaacaaatgtcaaaactttcaacaaaggatctcttggctctc
>OTU_237_430
ttaccgagttagggtcttctcaggcccgacctccttacctttgtctaccttacctcacgttgcttcggcgggcccgtcctcttttggaccgccggagggttcacccccctctggcccgttgcccgccgacagccccaaccaaaaaactcttgcaataactgtgaaattgtctgaattttgcttctaaaataaccaaaaactttcaacaacggatctcttggttctg
>OTU_238_58
ttaaagaatgccccgttttttgaaatgggttctattcccaaaccgtgtatacatacctttgttgctttggcaggccgccttcgggcgtcggctcacgctgacccgcgcctgccagaggatccaaactcgtttgtttagtgacgtctgagtactatataatagttaaaactttcaacaacggatctcttggttctg
>OTU_239_241
ttattgaataaacctgatggactgttagctggcttttcgaagcatgtgctcgtctgtcatctttatctctccacctgtgcacattttgtagtcttggatacctctcgaggcaactcggattttaggatcgccgtgctgtacaagttggcttttctttcatttccaagactatgtttttatatacaccaaagtatgtttaaagaatgtcatcaataggaacttgtttcctataaaattatacaactttcagcaacggatctcttggctctc
>OTU_240_958
ttaaagagttagggtcttctaggcccgatctcccaaccctttgtttattgaacctctgttgcttcggcggacccgtctcacggccgctggaggaccgctgaaaggcgtcctctagtcagcgtccgccgatagccaaccacttaaactctgaataaatcgtgtcatatgtctaagtctatgattaaattaaagcaaaactttcaacaacggatctcttggttctg
>OTU_241_71
ttaccgagttttcaactcccaaacccctgtgaacataccatgttgcttcggcggatcaccccaagcccctcggggcgcggggcccgccagaggacccaaaactcaactgtattttttgtatcaaaacgtattctgagtggaattttaaataaatcaaaactttcaacaacggatctcttggctctg
>OTU_242_2015
ttatcgtacaacggaggcacaagggctgtcgctgaccttcgagggtcgtgcacgcccatgcgccctctcacacatccacctcacccctttgtgcatcaccgcgtgggtcccccttacggagggctcgcgttttcacataaaactcgacacaatatagaatgttttctttttcatttgcggtcacacgcaatcaatacaactttcaacaacggatctcttggctctc
>OTU_243_1008
ttaccgagtgagcgtctccgggcgcgacctccaaccctttgtgattatacctctgttgcctcggggtgacccggccccgcccggcccccgaaggacgatttgacccaactctgcatctttgcgtcggattgaaaagattaattgatgaaaactttcaacaacggatctcttggttctg
>OTU_245_89
ttattgaaataaacctgatgggttgttgctggttctctagggagcatgtgcacaccttgtcatctttatatctccacctgtgcactttttgtaggccttttaggtctatgttgcttcatttaccccaatgtatgttaatagaatgttgtgcctttgtaatatatacaactttcagcaacggatctcttggctctc
>OTU_246_503
ttaaagagttagggtcctctgggcccgacctcccaaccctttgtttactgaacctctgttgcttcggcggacccgtctcacgaccgccggaggatcgcccgtccaggcgtcctctggccagcgtccgccgatagccaacccttcaaactcctgaataaatcatgttatatgtctaagtcttatgattcaaatcaataaagcaaaactttcaacaacggatctcttggttctg
>OTU_249_34
ttacagagttcatgcccctcggggtagatctcccacccttgtgtatcattatagaatgttgctttggcgggtcgcgcctcgtgcgcctagattcgcgtctagcgtgcccgccagaggacccctaaactctgaatattggtgtcgtctgagtactatgaaatagttaaaactttcaacaacggatctcttggttctg
>OTU_250_28
ttattgattgcgaatcgttgccttctgtgctggccagctctggcaagtgcacgtcggtgactttcatccaataccctgtgaacctttggcctctgctagctttggccggcaggggaatttacacacactcgaatgtaatgaaatttattgtcgtgcgcaagcactaatgtacaactttcaacaacggatctcttggctctc
>OTU_251_540
ttaaagagtaccggagctctcgggttcctactcccaccctatgttgactttaaatgttgctttggcggaccggcaatcttgccactggcctaggctggacagcgtccgccagaggatttttaaattcctttgactgaattctgagtctttaaaaattgaatcaaaactttcaacaacggatctcttggttctg
>OTU_252_2
ttattgatattaattgtctaccttcagtgctggctcttgcaagtgcacgacggggacataatataaatctaataccctcttgtgaaccattggcctttgctagcttcggctggtagaggatttttacacgcacgaatgtaatgaaataatcttgtgctgagcactataaatatacaactttcaacaacggatctcttggctctc
>OTU_254_35
ttacagagactctgccctttgggtagacctcccaccctgtgtcgttatacctttgttgctttggcgggccgcggggctccggccctgcccctggctccggctagggcgcgcccgccagaggatctcaaaacctgaacgttagtgtcgtctgagtactatataatagttaaaactttcaacaacggatctcttggttctg
>OTU_256_7
ttatcgaaacagaacgcccgggggggaagggggagcgccgacggccggagactgtcgcacggcctcgtgcacgtccctcgccgtcgctccttccgaccctttgaggcttcgaccttctcaacacccgtgcacccactgtaggtccttcgggatctacgtccttcttcgaactcgcatgtctacagaacgtattcgtcgtgtcccggcctcgaccctcagggtcccgcgtcggcgaccgcaaaacctataatacaactttcagcaacggatctcttggctctc
>OTU_259_29
ttatcgaattttgaaagggctgttgctgatcttttagatacgtgcacgccttgttccaaatttctacaccttgtgcacattttgtagaccggtttcattagaaatcggtttatgttttactatatatacttgttttagaatgtcatataataagcttgttagcaagcttaacaaatttaatacaactttcaacaacggatctcttggctctc
>OTU_260_38
ttacagagttcatgcccttcggggtagatctcccacccttgtgtatcattatagaatgttgctttggcgggccgcgtgcctagcacgcctcgattcgcgtcgagcgtgcccgccagaggacccctaaactctgaatattaatgtcgtctgagtactattcaatagttaaaactttcaacaacggatctcttggttctg
>OTU_261_22
ttattgtggggacctcggtccttccaagatgcaacccttgcctttttcaagcgtctctctgtttcctcggcagctgtgcctgccagcgaggaccttcaaaaaccttttgcaatccccgtacaaacttctgaaaacaccaaaaaaacgttaaaactttcaacaatggatctcttggttctg
>OTU_262_10
ttattgactgtgaatcgttaccttctgtgctggctccggcaagtgcacgttggtgactttcattcccaacacccttgtgaacctttggcctcttgctagcctcggctggcagaggatttacacacagactcgaatgtaatgaaaaacctcttgttgtgcgcaagcacgaatgtacaactttcaacaacggatctcttggctctc
>OTU_265_34
ttaccgagttcatgccctgacgggtagatctcccatcctctgttatcattacctttgttgctttggcgggccgtcaggccccggtcagactaccggctccagctggtaagcgcccgccagaggaccctaaaccctgaatatcagtgtcgtctgagtcctatgtaatcgttaaaactttcaacaacggatctcttggttctg
>OTU_266_9
ttactgaataactgagaggttgtagctgtctcttcggagaatgtgcacgccgctcaaattcatcttaacctcctgtgcactgttgtagactatgataactctcagctacctagtagttggattgaaggacttgcgctttagctgtctttcaatatcgtagtctatgttttatctatatacaaaaagtctagaatgtcgttcatgggtcttgtacctataaactttatacaactttcaacaacggatctcttggctctc
>OTU_267_3
ttattgaataaacttggttgggttgctgctggctttttaggagcatgtgcacactcactccattttacccacctgtgcacatactgtaggcctgaatggaactgctcgaggaaactcggttggagaattgctgtgaaaatcagctgttctttcatttcaaggtctatgtttttaaacatatactccataacaagttatagaatgttaatcatgggccttctgtgcctttaaaatgtaatacaactttcaacaacggatctcttggctctc
>OTU_268_715
ttatcgtataacagaggtgtaagggctgtcgctgacctttaaaggttgtgcacgcctaagccctctcacacaatccatctcaccccttttgtgcatcaccgcgtgggtcctccctttgccgggagggcctgcgtttttatataaaacttgacacaatgtagaatgttttcttttttgcaattatatgcaaatcaatacaactttcaacaacggatctcttggctctc
>OTU_269_1
ttatagaatacacttgaatgggctgttgctctcccatagggtggtgcacgcctgtcatatttatctctcccactgtgcacattttgtagacctggtgtttgggaattgctgtgggtcagctctgcccttttggcctttgccagtctatgttattttatcacaacctctgaatgttttgaactttataatgatggaattttatacaactttcagcaacggatctcttggctctc
>OTU_270_1696
ttatcgtaccaaatgtgtcaggcatgctcgggctgttgctgactcaaaaagtcgtgcacgctggagggtgtcctctcacataataatccatctcaccctttgtgcatcaccgcgtgggcaccctttgggatcggaccgatctcggaagggggcccgcgttttcacacaaacccccccatctaaaagtgtagaatgtcctcatttatgcaataaatacaactttcaacaacggatctcttggctctc
>OTU_271_307
ttactgagaacatgccctttagggtatatctcccaccctttgtttacaataccattgttgctttggtgggcccgtcatatgaccaccggctttggctggtttgtgcctgccagaggaccccaaaactctttattatgtcgtctgagtactatgtaatagttaaaaaactttcaacaacggatctcttggttctg
>OTU_272_61
ttactgaaaaactattttcaagttttttatatcccattgtttacttaccctgttgcttccactggacagatttcatcatgtgtgggatcttttggcttgtgtgtataatacttgccaaagagttgccagtggcaagatttttttttaccaaaacttgattaaaaacacattgtctgaatatatttcttgaatgaaacaaaactttcaacaacggatctctaggctctt
>OTU_273_16
ttaccgaattgtcaacacgggttgttgctggtcctcatatgggggcatgtgcacactctgttcacacatccactcacacctgtgcaccctccgtagttctgtggcatgggggactctgtcctcttgccgtggttctacatctttacacacacactgtaataaagtcttatggaatgcatatcgcgtttaacgcaatacaatacaactttcagcaacggatctcttggctctc
>OTU_275_9
ttactgaagcagttagggttgtagctgactctgtcaaaggagtactgtgctcgctctattcttctacttttccccctgtgcaccttttgtaggctatgatatccatcgtgtgtgagttcgcgctcattaccggttcaagggattgctgggtttactccggctgctcttgcccttcatatgcctatgtctttatacactctttacaagtctagaatgtcttttgtgggtctattgacctataaacttaatacaactttcaacaacggatctcttggctctc
>OTU_276_1704
ttattgaataaacttggttgggttgctgctggctcttaggagcatgtgcacgctcactccattttaaccacctgtgcacatactgtagacctgaatgaaacttttcgaggtaactcggtttgaggactgttgtgaaaatcagctgttcttgcattccaggtctatgtttttaaaaatatactccataacaagttatagaatgttaataatgggccttctgtgcctttaaattataatacaactttcaacaacggatctcttggctctc
>OTU_277_610
ttatcgtacaatggaggtgctggggttgtcgctgacctttgaaagggtcgtgcacgcctcggtgctttcacacacaatccatctcaccccttttgtgcatcaccgcgtgggggtcccttttagctagttctgaagggggctttcgcgtttttacaaacacacccttttaatgcaatatgtagaatgtcttactttttgcgatcacacgcaatcaatacaactttcaacaacggatctcttggttctg
>OTU_278_760
ttaaagagtaccggagctctcgggttcctactcccaccctatgatgactttaaatgttgctttggcggaccggcaatattgccaccggcctaggctggatagcgtccgccagaggatttttaaattcctttgaatgtgaattctgagtctttgaaaattgaatcaaaactttcaacaacggatctcttggttctg
>OTU_281_11
ttactgaattgtcaaacgggttgttgctggccctcatagggggcatgtgcacgctctgtttacacatccactcacacctgtgcaccctctgtagttctatggtttgggggaccctgtcttccttctgtggttctacgtctttacacacacactgtaataaagttttatggaatgtacatcgcgtctaacgcaatacaatacaactttcagcaacggatatctcggctctc
>OTU_282_22
ttatcgaataaacttgaacaggttgttgctgaccctcatggggtatgtgcacgcttgtccacctttatcatttctccaattgtgcacattttgtagaccctggaggttttgattttagtcgagttgggaccgctgtgttctctttcaagaggccagctttaccttgcattttccaggtctatgtcactttttcacaaccttatatatgtttagaatgtgttggatcatacctataaaaagttgaaatatacaactttcagcaatggatctcttggctctc
>OTU_283_1614
ttattgaataaaaacttgaacaggctgtttttttgctggccctcctctcagagggcatgtgcacgcttgtcattttttgtttctccaactgtgtgcacacattgtagacccgcccctggtgtagagtctcgtatgggatttgattgaggattgcttatgctttccttgcaggtggtctatattcttcactacctttttatgttttgaatgggataataataaagtttatacaactttcagcaacggatctcttggctctc
>OTU_284_33
ttaaaggtttcgggtacttagtgcccaaacttcaaccctatgttttaactaaacctgtttctttgccggtttcggccggcagaagttttctcaaaactcatttataaacgtgtcttctgaatcaaaaccaaataaattaaaactttcaacaacggatctcttggttctg
>OTU_285_14
ttaccgagttagggtctaaacaggcccgacctccaaccctttgtttactataccatgttgctttggcgggcccgcctttcggggctgccgggggcttacatacccctggtcagtgcccgccagtagccttattaaattcttccataattatgttgtctgagtataactataaaatcgttaaaactttcaacaacggatctcttggttctg
>OTU_286_4
ttatcgaattttgaaagggctgttgctgatcttttagatacgtgcacgccttgttccaaatttctacaccttgtgcacattttgtagaccggtttcattagaaatcggtttatgttttattatatatacacctgttttagaatgtcatataataagcttgttagcaagcttaacaaatttaatacaactttcaacaacggatctcttggctctc
>OTU_288_135
ttagtgattgtgaatcgttgcctttggtgctggctcttgtacaagtgcaagggcaagtgcacattggtgactttcatccaataacccatgtgaacctttggcctcttgttggcttcggctgacagaggattattttacacattaactcgaatgtaatgaaaactactgttgtgcgcaagcacgaatgtacaactttcaacaacggatctcttggctctc
>OTU_291_28
ttacagagttcatgcccttcggggtagatctcccacccttgtgtatcattatagaatgttgctttggcgggccgcgtgcctcgcacgcctggattcgcgtccggcgtgcccgccagaggacccctaaaactctgaatgttagtgtcgtctgagtactattcaatagttaaaactttcaacaacggatctcttggttctg
>OTU_292_236
ttaccgagtgagggctctcggcccgacctcctaccctttgttgaccaacacctgttgcctcgggggcgacccggcctccgcgccggcccccggcggaccactttaacgctgtttctatacgtcggagtacttgatgaatcaatcaaaactttcaacaacggatctcttggttctg
>OTU_293_29
ttaacgcatctctggcggcgagggtgcgcccagctccggaccgaaaggccgtgcagcgggctatcccggaccgccgaacctttgcaaacccactgtgcacttttgtcggcaaagggccttagcgggcctcgagccggatcacaaacactttgttaattgaatgcaagcggacattgagccgcctaaactttaacaactttcaacaacggatctcttggctctc
>OTU_295_21
ttacatggcgtgcgacccgcatgcggacgcgcccgggctccggcctcgggggatggtcgcgtgcggctcctcgctctatatacccttgcctatttgtaccctcttgttgtttcctcggcgagtcctgactcgccggcgggaattttataaacctcttgcagtagtatccaatcgttctgatcaaaagtttttaaatcgttacaactttcaacaatggatctcttggttctg
>OTU_296_1371
ttattgattgcgaatcgttgtctccagtgctggccaggtcactctggcaagtgcacgtagacagctttcatccaataccctgtgaacctttggcctcttgctagcttcggcgagcagaggattttacacccactcgcatgtaatgaaattattgttgtgcacacgcactactatacaactttcaacaacggatctcttggctctc
>OTU_298_63
ttacaaaatgtatgggatgccctatcggactcccaagcaaaacacattcctgtgtactctcccccattaaacatttgaaccaattagtagtctgagaaggccatgtgccgtaaaatttaaacatgttaaaactttcaacaacggatctcttggctctc
>OTU_299_20
ttaacgagctagggtcttcttggcccgacctcccaaccctatgtttactgaacctttgttgcttcggcggacccgtctcacgaccgccgggggaccgtaagacgtcctctggcccgtgtccgccggtggcccaactgaacaaattctgattaaaacgtgtcaatgtctgagtagaattcataattaaaacaaaactttcaacaacggatctcttggttctg
>OTU_301_487
ttacagaaagtaaacgcggatcaatccgcgaacttctaaacctttggcgattgactcatgttgcctcggcgggttctctcgccagaggatacatcaaaactcctgttttaacggtgttgtctgagctacaagcaacgaatcaaaactttcaacaacggatatctaggctctc
>OTU_302_16
ttattgaatttgatttgttaccttctgtgctggccccggcatgtgcacgttggtgactttcatgcaatacccttttgtgaacctttggcctcatgctggctttagctggcagaggattttacacaaactcgaatgtaacagaaagaacttgttgtgcgcaagcactaatgtacaactttcaacaacggatctcttggctctc
>OTU_303_436
ttacaggactcgcaaggctcccgtaaccactgtgaacttacctatattcgttgcctcggcgggtgccctagactcatatctagggcattcaacccgccggtggcctacaactctgtctcgcgcgttggcatctccgagtcattttaaacgagtcaaaactttcaacaacggatctcttggttctg
>OTU_305_125
ttagtgaattaaacatgcttggtgtcttcgcttcggcaaggcccttgcttaaatcacatcctaacacctgtgaactgtaagacgtatgatgaggtctttggccaagtcatcgtctgcccttttttaacaaacaattaatgtaacaaacgtagtcttattataacctaataaaactttcaacaacggatctcttggctctc
>OTU_306_459
ttaacaaatgtctgggatgctcaatatgagctcccaacaaaacacatccatgtgtatcctcccatgttgctttcccaggccagtggccactgctgccagccagtccgaaattcggttacaaggttgaggtgcttgggaaagggttaaatatcaaactttatatgaacctattgtctgacaaggccatatgccgtaatatttaaacaagttaaaactttcaacaacggatctcttggctctc
>OTU_309_7
ttactgaaaaacttctaaagttttttatatcccattgtttacttaccccgttgcttccactggacagatttcatcttgtgtggaatcttttggcttgtatgaacaatacttgccaaagagttgccagtggtgagacttttttaccaaaacttgattaaaacacattgtctgaatatatttcttgaatgaaataaaactttcaacaacggatctctaggctctt
>OTU_310_63
ttaccgaattgtcaacaagagttgttgctggtcctcaaacaggggacatgtgcacgctctgttcacacatccacccacaccatgtgcaccctctgtagttctatggtcaagggggcctgttcctcttgctgtggttctgcttctttacacacacaccgtaacaaagtctcatggaatgcatgccgcgtttaacgcaatacaatacaactttcagcaacggatctcttggctctc
>OTU_312_381
ttaccgagtgagggccctctgggtccaacctcccacccgtgtttatcgtaccttgttgcttcggcgggcccgccacttgtggccgccggggggcacctgcccccgggcccgcgcccgccgaagacaccattgaacgctgtatgaagattgcagtctgagcttattagctaaattagttaaaactttcaacaacggatctcttggttccg
>OTU_313_3
ttattgattgcgaatcgttgtctccagtgctggccaggtcactctggcaagtgcacgtcgacggctttcatccaataccctgtgaaccctttggcctcttgctagcttcggcgagcagaggattttacacccactcgaatgtaatgaaatcattgttgtgcacaagcactaatatacaactttcaacaacggatctcttggctctc
>OTU_315_1363
ttattgaataaacttgaacaggctgttgctggtccctcttcagggacacatgtgcacgcttgtcatctttatctctcctcatgtgcacattttgtagaccctggtgtttgaggattgcttattttgctctccttgtttgggttatatcccaggtctatgttatttttcacaatctctttgaaatgtattggaatgtcaataataataaaaagttgatacaactttcagcaacggatctcttggctctc
>OTU_318_1344
ttaacgtatcctggcggggtgcggtctgcccagctccggtcccctcgggccgtgcagcgggcgtccgtccctgtccacacctcacaaacccactgtgaaccttcacggcggatggggcctccggttaacgctggtgcgcctctgatgccgcataccaaactccaagtttaaagaatgtcgtgcggacattgtgccgcctaaaacactaaacaactttcaacaacggatctcttggctctc
>OTU_319_14
ttaatgaattgtgatggggtttgatgctggcagccaacttttggatgtatgtgctcgccctaacaatcatcttccaaacacctgtgcacatttttgagggagttttgagttgattgccgcttttggtgatcttcttgcattcccttaaatcattatacgctgttatcaatgctgaacgtgctttgtgccgcaaggccattaatataatacaacttttaacaacggatctcttggctctc
>OTU_320_1
ttaacgagctagggtcttctcggcccgacctcccaaccctatgtttactgaacctttgttgcttcggcgggcccgtctcacgaccgccgggggaccgtaagacgtcctctggtccgtgtccgccggtggccaaactgaacaaattctgattaaaaatgtgtcaatgtctgagtacaattcataattaaaacaaaaactttcaacaacggatctcttggttctg
>OTU_321_61
ttactgagttagggtcttataggcccgatctccaaccctttgttaactataccatgttgctttggcgggcccgcctttcagggccgccgggggctttcatacccttggtcagtgcccgccagtagccttattaaattctttcataattatgtttgtctgagttataaaattaatcgttaaaactttcaacaacggatctcttggttctg
>OTU_322_51
ttaaagagtaccggagctctcgggttcctactcccaccctatgatgactttaaatgttgctttggcggaccggcaatattgccaccggcctaggctgggtagcgtccgccagaggatttttaaattcctttgaatgtcaactctgagtctttgaaaattgaatcaaaactttcaacaacggatctcttggttctg
>OTU_325_1296
ttattgaaattatcggtgagggttgctgctggcctttcggggcatgtgcacgcccgagcctttaaatccacatacacctgtgaacccaatgtaagggcccgtaacaaggcccctacgtctttatcatcaacccatcgcatgtctcatagaatgtcattcaatatcttcgccttaacaaagcgtcggtaaacttatacaactttcaacaacggatctcttggctctc
>OTU_329_244
ttactgaattactgagaggttgtagctgtctcttcggagaatgtgcacgccgctcaaattcatcttaacctcctgtgcactgttgtagactatgataactctcaactacctagtagttggattgaaggacgtgcgcttttagctgtctttcaatatcgtagtctatgttttatctatatacaaaaagtctagaatgtcgttcatgggtcttgtacctataaactttatacaactttcaacaacggatctcttggctctc
>OTU_330_8
ttaacgagtaccggtgccttcgggtgcctactcccaccaaatgtgaaccatttaactgttgctttggcggaccggtagctctctaccctccggctccggctggagagcgtccgccggaggatttttaaacccgcttaaccgtggcatctgagtcttgataaaaagtcaaaaaaactttcaacaacggatctcttggttctg
>OTU_332_4
ttacagagttgaaagactcccaaaccactgtgaacatacccgtgagcgttgcctcggcgggcggccccagggcggggccgcaacctccccaagctagcggggaggtgcccgccgcaggcccatacaaactatcttgaaacttagtggcctctctgagtcggttaccaataattaaaactttcaacaacggatctcttggctctg
>OTU_335_697
ttactgaactgtcaacacgagttgttgctggtcctcaaatgggggcatgtgcacgctctgtttacatacccactcacacctgtgcaccctctgtagttctgtggtgtgggggactctgtcctcccgctgtggttctacgtctttacacacacacagtttcatagaatgtatgtcgcgtttaacgcaatacaatacaactttcagcaacggatctcttggctctc
>OTU_337_22
ttactgaattgtcaacaagagttgttgctggtccccagatgggggcatgtgcacgctctgttaacacatccactcacaccctgtgcaccctctgtagttctatggttgggggggggacctgtccccctcctgctgtggctctgcatctttacatacacactgtaacaaagtctaatggaatgcatgccgcgtttaacgcaataaaatacaactttcagcaacggatctcttggctctc
>OTU_338_5
ttaccgagttagggtagtcactcactgcccgacctcccaaccctgtgtctaccacactttgtcgttgcttcggcggaccggttgaccaactggtcgtgaccgccgggggttggctctgtcccccctggagagcgtccgtcgatggcccaaccacaaaatctcttgtaccgaaacgtgtcgtctgaattattgagaaatcaaaaaaacaaaactttcaacaacggatctcttggttctg
>OTU_341_16
ttaccgaatcgtcaaacacgggttgttgctggcctccaaacgggggcacgtgcacgctctgtttacgcatccactcacacctgtgcaccctctgtagtcctatggttcggaagaccccgtcttccttctgtagctctacgtctttacacacacactgtagtgatgtctcatggaatgtttttatgcgtttaacgcgatacaatacaactttcagcaacggatctcttggctctc
>OTU_342_53
ttaccgagtttacaactcccaaacccaatgtgaacgttaccaaactgttgcctcggcggggtcacgccccgggtgcgtaaaagccccggaaccaggcgcccgccggaggaaccaaccaaactctttctgtagtcccctcgcggacgtatttcttacagctctgagcaaaaattcaaaatgaatcaaaactttcaacaacggatctcttggttctg
>OTU_349_86
ttaaagagtaccggagctctcgggttcctactcccaccctatgttgactttaaatgttgctttggcggaccggcaatcttgccactggcctagggctagacagcgtccgccagaggatttttaaattcctttgactgaattctgagtctttgaaaattgaatcaaaactttcaacaacggatctcttggttctg
>OTU_350_37
ttaccgaactgtcgacaggagctgttgctggccctcggaagggggcacgtgcacgctctgtttacacatccactcacacctgtgcaccctctgtagttctatggccttggggaccccgtccccttgccgtggtcctacgtctttacacacacaccgtaacaaagtatcatggaatgtatgtcgcgtttaacgcaatacaatacaactttcagcaacggatctcttggctctc
>OTU_351_116
ttaaagagttagggtcttctaggcccgacctcccaaccctatgtttattgaacctctgttgcttcggcggacccgcctcacggccgccggaggaccgctgaaaagcgtcctctggccagcgtctgtcgacagccaaccacttaaactctgaatgaatcgtgtcatatgtctaagtctatgattaaattaaagcaaaactttcaacaacggatctcttggttctg
>OTU_353_927
ttaccaaaagataatctttcaacattgaaagatcttacctttgtgctggctttgacagttttgtacttttggggctttaaaatggttcagtagttaaaggagggagcaatccctttttttcttgctactgggtcggccccaaataatcatatcatccttaaaaaatttttctgataaattaacacatgattttaataatctgtttaaaacaactttcaacaacggatctcttggttctc
>OTU_354_137
ttaccgagtttacaactcccaaacccaatgtgaacgttaccaaactgttgcctcggcgggatctctgccccgggtgcgtcgcagccccggaccaaggcgcccgccggaggaccaaccaaaaactcttttgtataccccctcgcgggttttttatatctgagccatctcggcgcctctcgtaggcgtttcgaaaatgaatcaaaactttcaacaacggatctcttggttctg
>OTU_355_21
ttacagagttcatgccctcacgggtagacctcccacccttgtgtatctataccatgttgctttggcaggctgctggccccctcgggggacagccccagcgccctcgggcctgagagtcgcctgccggaggaaaaacaaactctgaactgttagtgtcgtctgagtactatattttaatagttaaaactttcaacaacggatctcttggttctg
>OTU_356_22
ttaaaaatatgaaccggaccggtccgctgcttgcagcgggctgcgaagggagatattatacaccctgtactttgtttacctttgttgctttggcgggccgtccgtttaggcgttggctccggccgatcgtgcccgccagaggacccaaactcttttattagtgatgtctgagtactatataatagttaaaactttcaacaacggatctcttggttctg
>OTU_358_1011
ttactgaattgtaaacaagggttgttgctggtcctcaaacggggacatgtgcacgctctgttcacacatccaatcacacctgtgcaccctctgcggttctgtggtcaggggggctttgccttcctgtgctgtggttctgcttctttacacacacacactgtaacaaagtcttgtggaatgtatgctgcgtttaacgcaatacaatacaactttcagcaacggatctcttggctctc
>OTU_359_859
ttacagagttcatgcccagtagggtagatctcccaccctgtgttgatatatacgtctgttgctttggcaagcccgctgggctttggttcggccaccggcttcggctggtgagcgcttgtcagaggaccctaaaacccttgaatgttagtatcgtctaagcaccatatgaatagttaaaactttcaacaacggatctcttggttctg
>OTU_361_20
ttaccgagtttacaactcccaaacccctgtgaacataccttactgttgcctcggcggatcagcccgctcccggtaaaacgggacggcccgccagaggacccctaaactctgtttctatatgtaacttctgagtaaaaccataaataaatcaaaactttcaacaacggatctcttggttctg
>OTU_362_192
ttacagaaagtaaacgcggatcgaaccgcgaacttctaaacctttgacgattgactcgcgttgcctcggcgggttctcccgccagaggatacatcaaaactcctgttttaacggtgttgtctgagctacaagcaacgaatcaaaactttcaacaacggatctcttggctctc
>OTU_363_1
ttaatcgaacaaacatgcttctcggcatgatgtttcaaaccccacctgtgtatcttacctgttgcttccgtgctgcacacgctgacgaggtgcctcagggtacggtctaccgagtactcgggacccccggtaccctggggagtcggcacgggagggataaccacaaactctttcctttgaatgccttctgtctgaactgtaatacatgaaaagttaaaactttcaacaacggatctcttggttctc
>OTU_364_49
ttatagagagcttcggctccctgtatgctattaccacaatgttattcaattctatgttgctttcactgcctgtacggtggtggggggttttcatactattctttttgcttagacctttaatctgtctgaatatgggaaattattacaaaactttcaacaacggatctcttggctcct
>OTU_365_549
ttatagagttcttgcccagtagggtagatctcccaccctgtgttgatatatacgtttgttgctttggcaagcccgctgggctttggttcagccaccggcttcggctggtgagcgcttgtcagaggaccctaaaacccttgaatattagtatcgtctaagcaccatatgaatagttaaaactttcaacaacggatctcttggttctg
>OTU_366_186
ttactgagaatgctctccggagccctcaaaccttgcgtacccatcttgtacttgcgggttgctctcgggcgcccgcgaggattcaaactgtttgaccatcgtcagattcttacaaaagaaaacaactttcaacaatggatctcttggctccg
>OTU_369_161
ttaccgagaaactgccctttgggtagatcttccaccctttgtttacattacctttgttgctttggcaggcccgtctttggaccgccggcttaggctggtctgtgcctgccagaggatcctaaaactcttgatttttgtattgtctgagtaatattataataagttaaaactttcaacaacggatctcttggttctg
>OTU_371_6
ttattgaataaacctgatgtggctgttagctggcttttttgaagcatgtgctcgtccgtcatttttatctccacctgtgcacattttgtagtcttggatacctctcgaggcaactcggattttaggattgccgtgctgttcaagtcggctttcctttcatttccaagactatgtttttatatacaccaaagcatgtttatagaatgtcatcaataggaacttgtttcctataaaattatacaactttcagcaacggatctcttggctctc
>OTU_372_429
ttacagagattgacatacctagtgtgtcacctccaaccccttgtacaatcaaccataaagttgctttggtgtttcatcgccagaggctccatataatctttttaatcaatgctgtctgagtaaaatataaaatcgttaaaactttcaacaacggatctcttggttccc
>OTU_373_2
ttattgaataaacctgatgggttgttgctggttctctagggagcatgtgcacgccttgtcatctttatacctccacctgtgcactttttgtagaccttttaggtctatgttgcttcatttaccccaatgtatgttaatagaatgttgtgcctatgtaataatatacaactttcagcaacggatctcttggctctc
>OTU_374_62
ttatcgagtaccgaggcccctgtgcctctgctcccaccctatgttgacctgcaacgttgctttggcgggccggcgggtacacccgccgccgggccgcgcctggagagcgcccgccagaggaccacgcgaaactctttgcaactttgagcgtctgagcacttttgagaattgaccaaaactttcaacaacggatctcttggttctg
>OTU_376_12
ttattgaagttgactcgggttgtcgctggctctcaggagcatgtgcacgctcgttcatattcatattctcttgtgcacctttagctagactattgaatacctctcgagtgaaaatctcggattgggggttgcgccttcagcttcccttgcattttcatagtctatgccttcatatacccttttgtatgtctctgaatgtcttttttgtgggttctttgtgacctataaaatctagtacaactttcaacaacggatctcttggctctc
>OTU_377_321
ttaccgaatcgtcaaacacgggttgttgctggctccccaaacggggggcatgtgcacgctctgtttacgcatccactcacacctgtgcaccctctgtagttctatggtttgggagacatcgtcttccttctgtggctctacgtctttacacacacaccgtagcgatgtctcatggaatgtttttctgcgtttaacgcaatacaatacaactttcagcaacggatctcttggctctc
>OTU_378_158
ttacagaaagtaaacgcggatcaatccgcgaacttctaaacctttgacgattgactcatgttgcctcggcgggctcgcccgccagaggatacatcaaaactcctgttttaacggcgttgtctgagctacaagcaacgaatcaaaactttcaacaacggatctcttggttctg
>OTU_381_989
ttatcgattccattctgaagaggtagagagactgtggctggccgtctatatacctatataagaccgcatgtgcacgtcccctcgccttttctatctacacacacctgtgcacctattgtagatccccatccccttttgcctttgaaagagggagggaacctatgtttttcatcacatcacacacaattgtatgtctagaatgtaatttcattgatcattgatcattgataaaaatataatacaactttcagcaatggatctcttggctctc
>OTU_382_4
ttacagagctgcgactccctcgggagccatctccaccccttgactaccaaatattgttgctttggcgggccgcggccctgccgccgccggccccggctggcgagcgcccgccagaggcttttaaaatcttgcttgcaatgagtctgagcgatacaaaaaatgcgaaaaactttcaacaacggatctcttggttctg
>OTU_383_112
ttatcgtacaaaatgtgtgaggcatgcgagggctgtagctgactcaaagttgtgcacgccggagtgtgtcctctcacataacaatccatctcaccctttgtgcaccaccgcgtgggcaccctccgatctcggagggggctcgcgttttcacacaaaaccccccccctttaaaaagtgtagaatgacctcatttatgcaatcaatacaactttcaacaacggatctcttggctctc
>OTU_388_2
ttacctagagttgtaggctttgcctaccatctcttacccatgtcttttgagtaccttcgtttcctcggcgggtccgcccgccgattggacaaacttaaaccctttgtaattgaaatcagcgtctgaaaaaacataatagttacaactttcaacaacggatctcttggttctg
>OTU_390_254
ttactgaattgtcaacatgaagttgttgctggccctcatgtgggggcatgtgcacgctctgtttacacatccattcacacctgtgcactctctgtagttctgtggtttgggggctctgtcctcctaccgtggtcctgcatatttacacatacacactgtgataaagtctcatggaatgtatgccgcgtttaacgcaatacaatataactttcagcaacggatatctcggctctc
>OTU_393_2
ttaccgaactgccaacacgggttgttgctggccctcaaattgggggcaagtgcacgctctgtttgcacatccactcacacctgtgcaccctctgtagttctatggcctgggggggactctgttcttcctgcgctgtggttctatatctttacacacacacactgtaaataaagtctcatggaatgtatgccgcgttcaacgcaatacaatacaactttcagcaacggatctcttggctctc
>OTU_397_1
ttatcgtacaaaatgtgtgaggcatgcgagggctgtcgccgacttaaagttgtgcacgccagagtgtgtcctctcacataaaatccatctcacccttttgtgcaccaccacgtgggcaccctttgggatcgaacccgatcttggagggggcttgcgttttcacacaaaccccattttaaaagtgtagaatgaccacatttttgcgataacacgcaatcaatacaactttcaacaacggatctcttggctctc
>OTU_398_35
ttatcaaaagtcaagtcgggggctgtaaagctctcgtctacacccatgtcttttgcgtactcttgtttcctcggtggcgcaagctgccgattggacaaaccaaaaccttttttgtaattgcaatcagcgtctgaaaataatctaattatttacaactttcaacaacggatctcttggttctg
>OTU_399_1
ttattgattgcgaatcgttgtctccagtgctggccaggtcactctggcaagtgcacggagacagctttcatccaataccctgtgaacctttggcctcttgctagcttcggcgagcagaggattttacacgcactcgaatgtaatgaaattattgttgtgcacaagcactactatacaactttcaacaacggatctcttggctctc
>OTU_401_46
ttaaagagtaccggagctctcgggttcctactcccaccctatgttgactttaaatgttgctttggcggaccggcaatcttgccactggcctagggctagacagcgtccgccagaggatttttaaattcttttgactgaatttctgagtctttgaaaattgaatcaaaactttcaacaacggatctcttggttctg
>OTU_403_612
ttgctggaacgcgccccaggcgcacccagaaaccctttgtgaacttataccttactgttgcctcggcgcatgccggcccccaggggcccctcggagacgaggagcaggcacgccggcggccaagctaactcttgtttttacactgaaactctgagaaaaaaacaaaatgaatcaaaactttcaacaacggatctcttggttctg
>OTU_404_29
ttactgagtgagggccctctgggtccaacctcccacccgtgtttattgtaccttgttgcttcggtgcgcccgcctcacggccgccggggggcttctgcccccgggtccgcgcgcaccggagacactattgaactctgtctgaagattgcagtctgagcataaactaaataagttaaaactttcaacaacggatctcttggttccg
>OTU_405_302
ttaacgagttagggtcttctcggcccgacctcccaaccctttgtttactgaacctttgttgcttcggcggacccgtctcacgaccgccgggggaccgtaagacgtcctctggcccgtgtccgccggtggcccaactgaacaaattctgattaaaatgtgtcaatgtctgagtagaattcataattaaaacaaaactttcaacaacggatctcttggttctg
>OTU_407_211
ttactgagaatgctctccggagccctcaaaccttgcgtatttatcttgtacttgcgggtggccctcgggtgcccgcgaggattcaaactgtttgactgccgtcagatttttgtctaaaaaacaactttcaacaatggatctcttggctccg
>OTU_408_13
ttaccgagttagggtagtattcactgcccgacctcccaaccctgtgtctaccacactttatcgttgcttcggcggaccggttgaccaactggtcatgaccgccgggggctggctctgtcccccctggagagcgtccgccgatggcccaaccacaaaactcttgtaccgaaacgtgtcgtctgaatcatgtttgagaaatcaaaaacaaaactttcaacaacggatctcttggttctg
>OTU_409_90
ttacagaaagtaaacgcgggtcaaaccgtgaacttttaaacctttgacgattgactcatgttgcctcggcgggttctctcgccagaggatacatcaaaactcctgttttaacggtgttgtctgagctacaagcaacgaatcaaaactttcaacaacggatctcttggttctg
>OTU_411_16
ttaatgaatggcctctgggccttcaaccttgcaaacctgtggaagcaacatgtgcttcggcgcctcggcgtcgcttttatgctacgaatcagtctttgtgtctgaatcaaacaaaagaaataaaactttcaacaaaggatctcttggctctc
>OTU_413_144
ttacagagactctgccctttgggtagacctcccaccctgtgtcgttatacctttgttgctttggcgggccgcggggccccggccctgcccctggctccggctagggcgcgcccgccagaggacctcaaaacctgaatgttagtgtcgtctgagtactatataatagttaaaactttcaacaacggatctcttggttctg
>OTU_414_2
ttaccgagttatcaactcccaaacccctgtgaacatacctgaacgttgcttcggcggggccgccccggcgcccacctcggcccggaaccaggcgcccgccggaggacccaaactcttgcttcatcagtggcattctctgagttacaaacaagaaaatgaatcaaaactttcaacaacggatctcttggctctg
>OTU_415_12
ttaccgagttcatgcccttacgggtagacctcccaccctatgttatcattacctttgttgctttggcgggccgccaggcttcggtcaggctatcggcttcggctggtaagcgcccgccagaggaccccaacatcctgattattagtgtcgtctgagtactatgcaatagttaaaactttcaacaacggatctcttggttctg
>OTU_416_31
ttaccgaattgtcaacaagagttgttgctggtcctcaaatgggggcatgtgcacactctgttcacacatccactcacaccctgtgcaccctctgtagttctatggttgggggacctgtcctcctgctgtggttctgcatctttacacacacattgtaacaaagtctaatggaatgcatgtcgcgtttaacgcaatacaatacaactttcagcaacggatctcttggctctc
>OTU_417_206
ttaccgagttagggtctcctaggcccgatctcccaaccctttgtttaccgaacctctgttgcttcggcggacccgcctcgcggccgccggaggactgcccctaagccgtcctctggccagcgtccgccgacagccaacccttctcaactctgaataaatcgtgttgtaaatgtctaagtctatgattcaaatcaaataaaagcaaaactttcaacaacggatctcttggttctg
>OTU_420_1
ttaacgagttagggttccttttgggcccgatctcccaaccctttgtctacttgaccatcgttgcttcggcgagcccgtcctcacggaccgccggagggaccttcactggccctctggtccgcgctcgtcggtggccctaactttaaaatctttaactaaacgtgtcttgatctaagcattatttgttaaataaaagcaaaaactttcaacaacggatctcttggttctg
>OTU_421_870
ttactgaactgtcaacacgagttgttgctggtcctcaaatgggggcatgtgcacgctctgtttacatacccactcacacctgtgcaccctctgtagttctgtggtgtgggggactctgtcctcccgctgtggttctacgtctttacacacacacacacagtttcatagaatgtatgtcgcgtttaacgcaatacaatacaactttcagcaacggatctcttggctctc
>OTU_424_148
ttatcgtacaatggaggtgctggggttgtcgctgacctttgaaagggtcgtgcacgcctcggtgctttcacacacaatccatctcaccccttttgtgcatcaccgcgtgggggtcccttttagctagttctgaagggggctttcgcgtttttacaaacacacccttttaatgcaatatgtagaatgtcttactttttgcgatcacacgcaatcaatacaactttcaacaacggatatctaggctctc
>OTU_426_2
ttattgaaataaatctgatgagttgttgctggctctctctctctagagagcattgtgcacacttgtcatctttatatctccacctgtgcacattttgtagacctggatcattctctgaaatgctaacccagcattttaggttggaggaatttgactttgttgtctttccttgcatttctaggtctatgttctttttcatataatcttattgtgatataatggaccctatataatataaacctatacaactttcagcaacggatctcttggctctc
>OTU_427_55
ttacagagttcatgccctcacgggtagatctcccaccctttgagtactatacttttgttgctttggcaggccgcttcggctaccggcttcggctggtgagtgcctgccagaggaccccaaactctgaattatagtgtcgtctgagaactatataatagttaaaactttcaacaacggatctcttggttctg
>OTU_428_38
ttactgagtgcaaactctccaaccattgtttatctacctattttgcttctccggtaggcggttccagggagggaccgagcctatggcctagcctgggcacctgccgaaggggtacctactgctctatattatactgtctgagaaacaaaaaaatatcaaaattttcaattgtggatctcttggttctg
>OTU_429_5
ttaaagaatttgccccgtcctctcgaggatgggtcctcttctaaaccgtgtatacatacctttgttgctttggcgggccgccgtctcggcgttggcttcggctgactgtgcccgccagaggaccccaactcttctgtttagtattgtctgagtactatataatagttaaaactttcaacaacggatctcttggttctg
>OTU_430_3
ttaacgagtgagggtcttctcggcccgacctcccaaccctttgtttactgaacctttgttgcttcggcggacccgtctcacgaccgccgggggaccgtaagacgtcctctggcccgtgtccgccggtggccaaaccgaacaaattctgactaaaatgtgtcaatgtctgagtagaattcataattaaaacaaaactttcaacaacggatctcttggttctg
>OTU_431_824
ttacaggaagccgaaaggcaacttcaaaccattgcgaacgtatccaagttgcttcggcggcgcgggacccctcgggggaccgcagcccacgccctccggggcgaggggcgcccgccggaggtcacaaactctcgtgtattacggtggcgtctctgagtaaaaaaataaataagtcaaaactttcaacaacggatctcttggttctg
>OTU_433_48
ttaacgagttagggtctctctggcccgacctcccaaccctttgtctacttgaccatcgttgcttcggcgagcccgtcctcacggaccgccggagggatctttactggccctctggtccgcgctcgtcggtagcccaacctttaaaatctttaactaaacgtgccttaatctaagtacaattattaaataaaagcaaaactttcaacaacggatctcttggttctg
>OTU_434_44
ttagaaagatgggtcctctgggacccttcatccacaaacctgcggaagtgagatgtgcttcggcgcctctgggcgtcgagtctatgctgtttagctttgtgtctgaagaaaaacaaaatttaaaactttcaacaaaggatctcttggctctc
>OTU_435_15
ttattgaatgaacttggtgtggttgtggctgaccctctcgggggtatgtgcacgcccgccattctcatctctccacctgtgcacctcttgtagacttggattgactttccgaggaaactcggtcgggaggactgctggcaatagctggctttccttgcatttccaagcctatgtttttcatataccccattgtatgtaacagaatgtatcattgggccttgtgcctataaaacctatacaactttcagcaacggatctcttggctctc
>OTU_436_23
ttaccgagttcctgccctcgcgggtagatctcccaccctgtgttatcattacctttgttgctttggcgggccgtcaggcttcggtcaggctaccggctccggctggtacgcgcccgccagaggaccccaaactctgaatattagtgtcgtctgagtactatctaatagttaaaactttcaacaacggatctcttggttctg
>OTU_440_17
ttactagagcaaaggataggcagcgccccacagaagcttgcttcgtggcgggctaccctacttcggtagggtttagagtcgtcgaacctctcggagaagttcggtcctgaactccacccttgaataaattacctttgttgctttggcgggccgcctcgtgccagcggcttcggctgttgagtgcccgccagaggaccacaactcttgttttttgtgatgtctgagtactatataatagttaaaactttcaacaacggatctcttggttctg
>OTU_441_145
ttaccgagttcatgcccttacgggtagatctcccaccctgtgttatcattacctttgttgctttggcgggccgccaggctccggtcaggctatcggcttcggctggtacgcgcccgccagaggaccctaacattctgattatcagtgtcgtctgagtactatataatagttaaaactttcaacaacggatctcttggctctc
>OTU_442_107
ttaccgagcgagaattctctgaattcaacctcccacccgtgtttattgtaccttgttgcttcggcgggcccgcctcacggccgccggggggcatctgcccccgggcccgcgcccgccgaagacaccttgaactctgtatgaaaattgcagtctgagtctaaatataaattatttaaaactttcaacaacggatctcttggttccg
>OTU_444_287
ttaacgagtgagggtcgtccaggcccgacctcccaaccctttgtttaccgaacctctgttgcttcggcggacccgcctcacggccgccggaggattgccgacaggcgtcctctggcccgcgtccgccgacggccaaccactaaaccctgaatcaaccgtgtcgtgtgtctcagtctatgattaaattaaatcaaaactttcaacaacggatctcttggttctg
>OTU_445_24
ttaccgagttagggtctaaaaaggcccgacctccaaccctttgtttactataccatgttgctttggcgggcccgcctttcggggctgccgggggctttcatgcccatggtcagtgcccgccagtagccttattaaattcttccataattatgttgtctgagtataaccataaaatcgttaaaactttcaacaacggatctcttggttctg
>OTU_446_158
ttaccgagtgagggccctctgggtccaacctcccacccgtgtttaacgaaccttgttgcttcggcgggcccgcctcacggccgccggggggcatccgcccccgggcccgcgcccgccgaagacacctgtgaacactgtctgaagttgcagtctgagaaactagctaaattagttaaaactttcaacaacggatctcttggttccg
>OTU_449_6
ttactgggtggcttcggcctaaaccccttaaccattgtgaacgataccagttgcttagctggtggcggtgaaaatcgcagtaccggcggcacaaaactcttttaatgtcatctctgagttacttttaaataagtcaaaactttcaacaacggatctcttggttctg
>OTU_450_2
ttaacgagtgagggtcttctcggcccgacctcccaaccctttgtttactgaacctttgttgcttcggcggacccgtaagacgtcctctggcccgtgtccgccggtggccaaaccgaacaaattctgactaaaatgtgtcaatgtctgagtagaattcataattaaaacaaaactttcaacaacggatctcttggttctg
>OTU_453_269
ttacagagttctcgccctcgcgggtagatctcccacccactgtaattgctaccgtgttgctttggcgggccgccgggcccagcccgccgccggccccggccggcgtgtgcccgccagaagccccgcaaactctgaatgtcagcgtcgtctgagtactatataatagttaaaactttcaacaacggatctcttggttctg
>OTU_455_481
ttactgagatcatgcccttaacggggtagacctcccaccctgtgttattacgattgttgctttggcaggccgccctcgggcgctcggcttcggctgaggcgtgcctgccagaagacccaaaccctgtatttagtgttgtccgagtactattttaatacgttaaaactttcaacaacggatctcttggttctg
>OTU_456_63
ttaccgagtttacaactcccaaacccctgtgaacatacctattgttgcctcggcggtgcctgttccgacagcccgccagaggaccccaaaccctgattacatttaagaagtcttctgagtaaccgattaaataaatcaaaactttcaacaacggatctcttggctctc
>OTU_458_37
ttaccgagtgagggccccccggcccgacctccaaccctttgtcgatttctaactgttgcctcggggggcgacccggacgtcagcgcccggacgtcccccagaggacccatcaactctgcatctttgcgtcggagtattgaataccaatcaatcaaaacttttaacaacggatctcttggttctg
>OTU_462_8
ttaccgagttagggtagtcactcactgcccgacctcccaaccctgtgtctaccacactttgtcgttgcttcggcggaccggttgaccaactggtcatgaccgccgggggctggctctgcccccctggagagcgtccgccgatggcccaaccacaaaactcttgtaccgaaacgtgtcgtctgaattattgagaaatcaaaaacaaaactttcaacaacggatctcttggttctg
>OTU_463_310
ttaatgattgcgaatggctgccttcagtgctggctcttatgagcatgtgcacgttggtggctttcatccaatacccatgtgaacctttggcctcttgctagctttggccggcagaggatttttatacacacactcgaatgtaatgaaaactattgtcgtgcgcaagcactaatgtacaactttcaacaacggatctcttggctctc
>OTU_464_3
ttaccgaattgtcaacacgagttgttgctggtcctcaaatggggacatgtgcacgctctgtttacacatccactcacacctgtgcaccctccatagttctgcagcctgggggggctctgttcccctgccgtggttctatgtatttacacacacacacacactgtagtaaagtctcatggaatgcataccgcgtttaacgcaatataatacaactttcagcaacggatctcttggctctc
>OTU_465_48
ttatcgtacaaaatgtgtgaggcatgcgagggctgtagctgactcaaagtcgtgcacgccggagtgtgtcctctcacataacaatccatctcaccctttgtgcaccaccgcgtgggcaccctccgatctcggagggggctcgcgttttcacacaaaacccccccccctttaaaaagtgtagaatgacctcatttatgcaatcaatacaactttcaacaacggatctcttggctctc
>OTU_467_226
ttacagaaagtaaacgcggatcaatccgcgaacttctaaacctttgacgattgactcgcgttgcctcggcgggttctcccgccagaggatacatcaaaactcctgttttaacggtgttgtctgagctacaagcaacgaatcaaaactttcaacaacggatctcttggttctg
>OTU_468_3
ttgctggaactgtgtctgcaaagacacaaccccagataccctttgtgaacttatttatatcgttgcctcggcgttgagctgggggcttctttccaagaagctctcccatcctctctcgggaagatggagcaagcccgccggcggccctcttaaactcttgtttttattacgtatctcttctgagttattcaaaacaaaatgaatcaaaactttcaacaacggatctcttggttctg
>OTU_469_152
ttaccgagttagggtcttctcaggcccgacctccttacctttgtctaccttacctcacgttgcttcggcgggcccgtcctcttttggaccgccggagggttgaacccctctggcccgttgcccgccgacagcccccaaccaaaaaactcttgcaataactgtggaattgtctgaatcttgcttctaaaataaccaaaaactttcaacaacggatctcttggttctg
>OTU_470_2
ttattgattgcgaatcgttgtctccagtgctggccaggtcactctggcaagtgcacggagacagctttcatccaataccctgtgaacctttggcctcttgctagcttcggcgagcagaggattttacacccactcgaatgtaatgaaattattgttgtgcacacgcactactatacaactttcaacaacggatctcttggctctc
>OTU_471_227
ttacagaaagtaaacgcggatcgaaccgcgaacttctaaacctttgacgattgactcatgttgcctcggcgggttctctcgccagaggatacatcaaaactcctgttttaacggtgttgtctgagctacaagcaacgaatcaaaactttcaacaacggatctcttggttctg
>OTU_477_8
ttactgaattgtcaaacgggttgttgctggccctcatagggggcatgtgcacgctctgtttacacatccactcacacctgtgcaccctctgtagttctatggtttgggggaccctgtcttccttctgtggttctacgtctttacacacactgtaataaagttttatggaatgtacatcgcgtctaacgcaatacaatacaactttcagcaacggatctcttggctctc
>OTU_479_3
ttaccgagtgagggtcttcacggcccgacctcccaccctttgttgactataccatgttgctttggcgggcccgccctttcggggccgccgggggcttttcaacgctcctggcccgtgcctgccagtagccttcttaaattcttttgataaccgtgtcgtctgagtcctaagatttaaatcataaaactttcaacaacggatctcttggttctg
>OTU_481_72
ttactgaattgttgacatgagttgttgctggtctccagatgggggcatgtgcacgctctgttcacacatccactcacacctgtgcaccctccgtagttctatggcgtgggggactctgtcctctcaccgtggttctacgtatttacacacacactgtaataaagtcctatggaatgtatgtcgcgtttaacgcaatacaatacaactttcagcaacggatctcttggctctc
>OTU_482_23
ttacagagttcatgccctcacgggtagatctcccacccttgaatactatacctttgttgctttggcgggccgcttcggctactggctctggctagtgagtgcccgccaaaggatcccaaaactctgaatatttgtgtcgtctgagtactatgtaatagttaaaactttcaacaacggatctcttggttctg
>OTU_484_7
ttactgagaaattgccctttgggtagatctcccaccctttgtttacattacctttgttgctttggcaggcccgtctttggaccaccggcttaggctggtctgcgcctgccaaaggaccctaaactcttgattttttgtgttgtctgagtaatatttataataagttaaaactttcaacaacggatctcttggttctg
>OTU_486_1
ttatcgtacaacggaggtgcaagggctgtcgctgacctttcaaaggtcgtgcacgtccgagtgctctctcacacaatccatctcaacacccccctttgtgcatcaccgcgtgggtccccgcatttttttgtggaagggctcgcgttttcacataaaactcgatacagtgtagaatgtcttttttcttcttttgcggtcacacgcaatcaatacaactttcaacaacggatctcttggctctc
>OTU_490_98
ttacagagttctcgccctcgcgggtagatctcccacccactgttattgctaccgtgttgctttggcgggctgctgggcctagcccggccgccggctccggccgccgcgcgcccgccagaggcccgacaaattctgattgtcagtgtcgtctgagtactatataatagttaaaactttcaacaacggatctcttggttctg
>OTU_492_66
ttatcgagttagggtcttctaggcccgacctcccaaccctttgtttactgaaccactgttgcttcggcgggcccgtctcacgaccgccggaggagcgctgaaaagcgtgtcctctggcccgcgtccgccgatggccaaccacaaaactctgtgtaaatcgtgtctttatgtctaagtcaatgattgaattaaaagcaaaactttcaacaacggatctcttggttctg
>OTU_493_303
ttactgaatggcctttgggccttcaaccttgcaaacctgtggaagcaagatgtgcttcggcgcttcagcgccgcttttatgccttaatcagtctatgtgtctgaatcaaatacaagaaataaaactttcaacaaaggatctcttggctctc
>OTU_495_83
ttaacgagtaacgggtccttctgggctcgcactccaaccctttgtttacctgctctgtgcctcggcagggtgcctctcaggtacacctgccgggagagaggccgcgacagacagctggttttggcttgcttgagacgcggcttctgctaaataaactctgtaaaacttaaagagtctgatctaaaatatatggaactaaaactttcaacaacggatctcttggttctg
>OTU_498_33
ttaacgagttagggtcttctaggcccgacctcccaaccctatgtttattgaacctctgttgcttcggcggacccgcctcacggccgccggaggaccgctgcaaggcgtcctctggccagcgcccgccgatggccaaccacttaaactctgaatgaatcgtgtcatatgtctaagtctatgattaaattaaagcaaaactttcaacaacggatctcttggttctg
>OTU_500_2
ttaatgattacgaacggttgccttcagtgctggctcttaggagcaagtgcacgttggtggctttcatccaacaccctgtgaacctttggcctcttgctagcttcggctggcagaggatttttataacacactcgaatgtaatgagaactattgtcgtgcgcaagcactaatgtacaactttcaacaacggatctcttggttctg
>OTU_504_23
ttatagtagtagggacttcggtccctccgtaatggaacccttgcctttttttagcgtctctccgtttcctcggcagctccgcctgccagcgaggacccccaaaaaacctttgcaatccagtaaaatattcagaattacacaaacgttaaaactttcaacaatggatctcttggctctg
>OTU_505_34
ttacagagttgcaagactccctaaaccatcgtgaacgttacccaaaccgttgcttcggcgggcggcgcccctgcgcgcccccgggccccaccgcgggcgcccgccggaggtcaccgaactcctgataatttatggcctctctgagtattctttactgaataagtcaaaactttcaacaacggatctcttggttctg
>OTU_508_5
ttaacgaatggtcttggtaccttcaaaacccctcaaacctgtggacatgaaaaaatgtgcttcggcgctccggcgccgcttttatgccgtaattctgtcgtttgtgtctgaatacaaagcaaagaaattaaaactttcaacaaaggatctcttggctctc
>OTU_511_2
ttaatgagttgcaaaactccaaccatcgcgaatgtaaccacttgcagttgcttcggcgagtggtccttctcaggaccacgttcctcccggggagcacactcgccgaagtcgtacaaactctttgtatttgttggcatctctgagtaactttataataagttaaaactttcaacaacggatctcttggttctg
>OTU_512_6
ttaccgagttcatgccctcacgggtagatctcccaccctatgttatcattacctttgttgctttggcgggccgccaggcttcggctaggctaccggctccggctggtaagcgcccgccagaggaccccaaactctgaatgttagtgtcgtctgagtactatctaatagttaaaactttcaacaacggatctcttggctctc
>OTU_513_10
ttactgagaacatgccctttagggtatatctcccaccctttgtttataataccattgttgctttggtgggcccgtcgtatgaccaccggctttggctggtttgtgcctgccagaggaccccaaaattctttattatgttgtctgagtactatgtaatagttaaaaaactttcaacaacggatctcttggttctg
>OTU_514_3
ttactagagcaaaggatagacactaccctacttcggtagggtttagagtcgtcgggcctctcggagaagctcggtcctgaactccacccttgaataaattacctttgttgctttggcgggccgcctcgtgccagcggcttcggctgttgagtgcccgccagaggaccacaactcttgtttttagtgatgtctgagtactatataatagttaaaactttcaacaacggatctcttggttctg
>OTU_515_221
ttaaagagttagggtcttctaggcccgatctcccaaccctttgtttattgaacctctgttgcttcggcggacccgcctcacggccgctggaggaccgctgaaaggcgtcctctggccagcgtccgccgatagccaaccacttaaactctgaataaatcgtgtcatatgtctaagtttatgattaaattaaagcaaaactttcaacaacggatctcttggttctg
>OTU_516_100
ttaccgagttagggtcttctcaggcccgacctccttacctttgtctaccttacctcacgttgcttcggcgggcccgtcctcttttggaccgccggagggttccaccccctctggcccgttgcccgccgacagcccccaaccaaaaaactcttgcaataactgtggaattgtctgaatcttgcttctaaaataaccaaaaactttcaacaacggatctcttggttctg
>OTU_517_17
ttataggaagccgaaaggcaacttcaaaccattgcgaacgtatccaagttgcttcggcggcgcgggacccctcgggggaccgcagcccacgccctccggggcgaggggcgcccgccggaggtcacaaactctcgtgtattacagtggcgtctctgagtggaaaaaaataagtcaaaactttcaacaacggatctcttggttctg
>OTU_525_3
ttacagagttgcaaaactcccaaaccattgtgaacattaccgtaactattgcttcggcaggtagttcccactagggttctgcatctccccctcggggtggataacctgccgaagttatttcaactcttgattttaattggtatctctgagtaacttataaaataagtcaaaactttcaacaacggatctcttggttctg
>OTU_526_99
ttaccgagttagggtcttctaagggcccgacctccttacctttgtctaccttacctcacgttgcttcggcgggcccgtcctcttttggaccgccggagggttgaacccctctggcccgttgcccgccgacagcccccaaccaaaaaactcttgcaataactgtgaaattgtctgaattttgcttctaaaataaccaaaaactttcaacaacggatctcttggttctg
>OTU_528_66
ttaccgagtgagggccctctgggtccaacctcccacccgtgtttaccataccctgttgcttcggcgggcccgccctcagcggccgccgggagggccccttgcgcctccgggcccgcgcccgccgaagacacctgtgaacgcttctattgaagattgcagtctgagtgattttttccaaatcaatcaaaactttcaacaacggatctcttggttccg
>OTU_529_2
ttaccgaattgtcaacatgggttgttgctggtcctcaactgggggcacgtgcacgctctgtttacacatccactcacacctgtgcacccactgtagttctatggcctggggggctacgtcccttttgccgtggctctacgtctttacacacacacactgtaataaagtttcacggaatgcatcatgcgtttaacgcaatacaatacaactttcagcaacggatctcttggctctc
>OTU_531_15
ttacagtgttccctgccctcacgggtagaaacgcccacccttgtgtattatatctttgttgctttggcaggccgccttcgggcaccggctccggctggatcgtgtcggccagaggacccccaaactctgaatgttagtgtcgtctgagtactatctaatagttaaaactttcaacaacggatctcttggttctg
>OTU_532_6
ttactgaattgtcaaacgggttgttgctggccctcatagggggcatgtgcacgctctgtttacacatccactcacacctgtgcaccctctgtagttctatggtttgggggaccctgtcttccttctgtggttctacgtctttacacacacactgtaataaagttttatggaatgtacatcgcgtctaacgcaatacaatacaactttcagcaacggatctcttggttctg
>OTU_533_25
ttaccgagtttacaactcccaaacccctgtgaacataccacttgttgcctcggcggatcagcccgctcccggtaaaacgggacggcccgccagaggacccctaaactctgtttctatatgtaacttctgagtaaaaccataaataaatcaaaactttcaacaacggatctcttggctctc
>OTU_536_117
ttactgaattgtcaacatgaagttgttgctggccctcatgtgggggcatgtgcacgctctgtttacacatccattcacacctgtgcactctctgtagttctgtggtttgggggctctgtcctcctaccgtggtcctgcatatttacacatacacactgtgataaagtctcatggaatgtatgccgcgtttaacgcaatacaatataactttcagcaacggatctcttggttctg
>OTU_537_24
ttaacgagtgagggtcgtccaggcccgacctcccaaaccctttgtttaccgaacctctgttgcttcggcggacccgcctcacggccgccggaggattgccgccaaggcgtcctctggcccgcgtccgccgatggccaacccctcaaaccctgaaccaaccgtgtcgtgtgtctcagttcatgaattaaattaaagcaaaactttcaacaacggatctcttggttctg
>OTU_542_1
ttaccgagtttacaactcccaaacccaatgtgaaccatacctagacgttgcttcggcggtaccgccccggcgccctcgggcccggaaccaggcgcccgccgggggaccaaccaaaccctttcagtgaatcccgggcgacccccggcggcattatctgagtacagcgaaataaaaaacgcatcaaaactttcaacaacggatctcttggttctg
>OTU_544_88
ttactgagatcatgcccttcacggggtagacctcccaccctgtattattacgattgttgctttggcaggccgccttcgggcactcggctccggctgaggcgcgcctgccagaagactcaaaccctgtatttagtgtcgtccgagtactattttaatacgttaaaactttcaacaacggatctcttggttctg
>OTU_545_8
ttacaagtgaggctaccgaacgttggaaacagcggttaggagcttacacccacccgtgtttacatactattgttgctttggcgggccgtggcctccactgcgggctctgctcgtgtgtgcccgccagagaaccaaactctgaatgttagtgatgtctgagtactatctaatagttaaaactttcaacaacggatctcttggttctg
>OTU_547_39
ttatcgtaaaacagaggtgcgcgggctgtcgctgactttttaagtcgtgcacgcccgagcgctctcacacaatccacgtcacccctatgtgcaccaccgcgtgggtcccccttgaaaggggggctcgcgttttcacacaaacttgaagtagtgtagaatgtctttttttgcgatgacacgcaatcaatacaactttcaacaacggatctcttggctctc
>OTU_550_307
ttattgagtgaagggttctctaagaacctgacctcccaacccttgtgttctttaccactttgctttggcgcgcccgtcactgtgaccgccgaagggttatgttttaactaacctttgggcccgtgcccgccagagatatctgaactcttgagatgaaataggttgtctgagttataaagaaaaataaatcaaaactttcaacaacggatctcttggttccg
>OTU_551_10
ttatcgaaaaaggctccggctttttttctgttgccataccttgaccccatttgttgacataacctttgttgcttccgtcggccgaaatgcctgcccccttgccactggcagttggtggcatcgaccggcggagggattttttgggaaaccagtattttagtctgaaaatgtttacggagatgaaattctcaaaaacaaaactttcaacaacggatctcttggctcct
>OTU_552_1
ttacagagttgcaaaactcccaaccctttgtgaacgaactgaacagttgcttcggcggaaggcccttcactgggtggcccgccggaggcctacaaacacctgtatttttagcatctctgagtaacttaccaataagttaaaactttcaacaacggatctcttggttctg
>OTU_553_87
ttacagagttctcgccctcgcgggtagatctcccacccactgtgattgctaccgtgttgctttggcgggctgctgggcctagcctggccgccggctccggccggcgcgcgcccgccagaggcccgacaaattctgattgtcagtgtcgtctgagtactatataatagttaaaactttcaacaacggatctcttggttctg
>OTU_554_8
ttattgtggggacctcggtccttccaagatgcaacccttgcctttttcaagcgtctctctgtttcctcggcagctgcgcctgccagcgaggaccttcaaaaaccttttgcaatccccgtacaaacttctgaaaacaccaaaaaaaacgttaaaactttcaacaatggatctcttggttctg
>OTU_555_2
ttaccagagcaaaggatagacagcgccccacagaagcttgcttcgtggcgggctaccctacttcggtagggtttaaagtcgtcgagcctctcggagaagctcggtcctgaactccacccttgaataaattacctttgttgctttggcgggccgcctcgtgccagcggcttcggctgttgagtgcccgccagaggaccacaactcttgtttttagtgatgtctgagtactatataatagttaaaactttcaacaacggatctcttggttctg
>OTU_558_558
ttattgaataaacttggttgggttgctgctggctcttaggagcatgtgcacactcactccattttaaccacctgtgcacatactgtagacctgaatgaaacttttcgaggtaactcggtttgaggactgttgtgaaaatcagctgttcttgcattccaggtctatgtttttaaaaatatactccataacaagtcatagaatgttaataatgggccttctgtgcctttaaattataatacaactttcaacaacggatctcttggctctc
>OTU_564_98
ttaaagagtaccggagctctcgggttcctactcccaccctatgttgactttaaatgttgctttggcggaccggcgatcttgccactggcctagggctagacagcgtccgccagaggatttttaaattcctttgactgaattctgagtcttgaaaattgaatcaaaactttcaacaacggatctcttggttctg
>OTU_566_3
ttaatcgaacaaacatgcttctcggcgtgatgtttcaaaccccacctgtgtatcttacctgttgcttccgtgctgcacacgctgtcaaggtgcttcagggtacggtctaccgagtacttgggacccccggtaccctggggagtcggtacgggagggataaccacaaactctttcctttgaatgccttctgtctgaactgtaatacatgaaaagttaaaactttcaacaacggatctcttggttctc
>OTU_568_7
ttaaagaattgccccgtcttctcgaagatgggttctattccaaaccgtgtttacatacctttgttgctttggcgggccgccgtttcggcgttggctccggctgactgcgcccgccagagaaccacaactcttttgtttagtaatgtctgagtactatataatagttaaaactttcaacaacggatctcttggttctg
>OTU_569_1
ttactgaaaaactattttcaagttttttatatcccattgtttacttaccccgttgcttccactggacagatttcatcttgtgtggaatcttttggcttgtatgaacaatacttgccaaagagttgccagtggtgagacttttttaccaaaacttgattaaaacacattgtctgaatatatttcttgaatgaaataaaactttcaacaacggatctctaggctctt
>OTU_570_27
ttaacgagttagggtcttctcggcccgacctcccaaccctatgtttactgaacctttgttgcttcggcgggcccgtctcacgaccgccgggggaccgtaagacgtcctctggtccgtgtccgccggtggccaaactgaacaaattctgattaaaactgtgtcaatgtctgagtacaattcataattagaacaaaactttcaacaacggatctcttggttctg
>OTU_571_485
ttatcgtaacaaaagaggtgcaagggctgtcgctgaccccccaaaggtcgtgcacgcccaagtgctttttacacaatccatcttcacccctttgtgcatcaccgcgtgggttcctcctttgcaggaaggacctgcgttttatcacaaaacttgatacggtgtagaatgtttctttttgcggtcacacgcaatcaatacaactttcaacaacggatctcttggctctc
>OTU_572_184
ttagtgaaacttcaaaagtcggctcgtcccgtactgtgctggtggagacacatgtgcacgttcgaggcgagtcgcaaatccacacacctgtgcatctatgactctgagtgccgctttgcatggccttgaccggcctggcgctcgagtactttcacacactctcgaatgtaatggaatgtcttgttgtgcgtaacgtacaaaacgaaacaactttcaacaacggatctcttggctctc
>OTU_577_48
ttaacgagttagggtctttctcggcccgacctcccaaccctatgtttactgaacctttgttgcttcggcggacccgttcttacgaccgccgggggaccgtaagacgtcctctggcccgtgtccgccggtggcccaactgaacaaattctgattaaaatgtgtcaatgtctgagtagaattcaattaaaacaaaactttcaacaacggatctcttggttctg
>OTU_579_1
ttgctggaacaaacgccctcacgggtgctacccagaaaccctttgtgaattatacctatactgttgcctcggcattggttggcctcttcgggggacccctttctcgcaaaggagcagaccggccggtggccctataaactcttgtttttgtaatatcatctgagtaaacaactataaatgaatcaaaactttcaacaacggatctcttggttctg
>OTU_581_100
ttaccgagtttacaactcccaaaccccctgtgaacttataccatttactgttgcttcggcgggtcacggccccggggaaggacagcggtcgccgtcaggcctcagctgcccgcccccggaaacaggcgcccgccggggaactcaaactcttctgtatttctttatctgatatatactgtctgagtaaaaactaaaatgaatcaaaactttcaacaacggatctcttggttctg
>OTU_582_23
ttactgagttagggtcttccaggcccgacctccaaccctttgcctaccttacctcttgttgcttcggccggcccgtcccccctagaaataggggtcgaccgccggagggctcacaccctctggtccgcgcccgccgatggccctcaaaccaaaactcttgttcaattgtgaattgtctgagtatacaaaacaaaataaacaaaaaactttcaacaacggatctcttggttctg
>OTU_586_29
ttacagagttcatgcccttacgggtagatctccccacccttgaatactatacctttgttgctttggcgggccgcttcggctactggctctggctggtgagtgcccgccaaaggatcccaaaactctgaatatttgtgtcgtctgagtactatgtaatagttaaaactttcaacaacggatctcttggttctg
>OTU_587_81
ttaccgagttagggtcttctcaggcccgacctccttacctttgtctaccttacctcacgttgcttcggcgggcccgtcctcttttggaccgccggagggttcaccccctctggcccgttgcccgccgacagccccccaaccaaaaaactcttgcaataactgtggaattgtctgaatcttgcttctaaaataaccaaaaactttcaacaacggatctcttggttctg
>OTU_589_2
ttaaaaagatgggtcctctgggacccttcatccacaaacctgcggaagtgagatgtgcttcggcgtctctgggcgtcgagtctatgctgtttagctttgtgtctgaagaaaaacaaaatttaaaactttcaacaaaggatctcttggctctc
>OTU_590_17
ttactgagttagggtcttccaggcccgacctccaaccctttgcctaccttacctcttgttgcttcggccggcccgtcccccctagaaatagggatcgaccgccggagggctcacgccctctggcccgcgccggccgatggcccacaaccaaaactcttgttcaatcgtgaattgtctgagtatacaaaacaaaataaaccaaaaactttcaacaacggatctcttggttctg
>OTU_591_268
ttaacgagttagggtctttctcggcccgacctcccaaccctatgtttactgaacctttgttgcttcggcggacccgttcttacgaccgccgggggaccgtaagacgtcctctggcccgtgtccgccggtggccaaaccgaacaaattctgattaaaatgtgtcaatgtctgagtagaattcataattaaaacaaaactttcaacaacggatctcttggttctg
>OTU_593_216
ttaacgagttagggtcttctcggcccgacctcccaaccctttgtttactgaacctttgttgcttcggcggacccgtctcacgaccgccgggggaccgtaagacgtcctctggcccgtgtccgccggtggccaaaccgaacaaattctgattaaaatgtgtcaatgtctgagtagaattcataattaaaacaaaactttcaacaacggatctcttggctctc
>OTU_595_11
ttattgattgcgaatcgttgtcttcagtgctggccagctctggcaagtgcacgttggcggcttttcatccaaataccctgtgaacctttggcctcttgctagccttggccggcagaggaatttttacaccaactcgaatgtaatgaaattattgttgtgcgtaagcactaatgtacaactttcaacaacggatctcttggctctc
>OTU_596_18
ttaaagagttgcaaaactcccaaaccattgtgaacataccttcaacgttgcttcggcgggttggccccggtctccggggtccccggccctactcgggcgcccgccggaggtatctaactcttgaacttttatggcctctctgagtctttgtacttaataagtcaaaactttcaacaacggatctcttggttctg
>OTU_598_129
ttactgagtgaaaggcttaaacagagccctatctccaacccttgtcttctgtaccactttgctttggcaggcccgccactatttgtggccgctgaaggatttttagttataaattattagctaaaccttgggcctgtgcctgccagggaacttaaatacatgttatgaaactggtttgtctgagaatccaaagaaaataactaaaactttcaacaacggatctcttggttctg
>OTU_599_13
ttaccgagttgtaacactcacaaacccactgcaaacgtacccattttggcgactccggcaggcggccccagggcggggcctcagccttttcaggcgcctgccgaaggtgccgcaactttactctataatcatgtattctgagtcctaagacaaaatatcaaaattttcaattgtggatctcttggttctg
>OTU_603_99
ttaccgagttagggtcttctcaggcccgacctccttacctttgtctaccttacctcacgttgcttcggcgggcccgtcctcttttggaccgccggagggttcactctcctctggcccgttgcccgccgacagcccccaaccaaaaaactcttgcaataactgtgaaattgtctgaattttgcttctaaaaataaccaaaaactttcaacaacggatctcttggttctg
>OTU_607_2
ttatcgtacaaccgaggtgcaagggctgtcgctgacctttgaaaggtcgtgcacgcccgagcgctctcgccacaatccatctcacccctttgtgcatcaccgcgtgggtccccctttgcggggagggctcgcgttttcacacaaaacttgatacagtctagaatgtttatttttgcggtaacacgcaatcaatacaactttcaacaacggatctcttggctctc
>OTU_609_2
ttattgaataagcttggttgggttgttgctggctctctggagcatgtgcacgcctgacaaccaacctttttaccacctgtgcaccttttgtagacctggatatctctcgaggaaactcggttttttgaggattgccgtgcgcaagctggctttccttgcacttccggtctatgtctttatatacccctacacatgttttagaatgtcattaatgggcttaactgcctttaaaatctatacaactttcaacaacggatctcttggctctc
>OTU_610_59
ttacagagattgacatacctagtgtgtcacctccaaccccttgtacaatcaaccataaagttgctttggcgtttcaccgccagaggctccatataatctttttaatcaatgctgtctgagtaaaatataaaatcgttaaaactttcaacaacggatctcttggttccc
>OTU_611_1
ttaatgaacggcctctgggccttcaaccttgcaaacctgtggaagcaacatgtgcttcggcgctttggcgtcgcttttatgctacgaatcagtctttgtgtctgaatcaaacaaaagaaataaaactttcaacaaaggatctcttggctctc
>OTU_615_123
ttgctggaacgcgcttcggcgcacccagaaaccctttgtgaacttataccttactgttgcctcggcgcaggccggcctcactgaggcccctcggaaacgaggagcagcccgccggcggccaaccaaactcttgtttcttagtgaatctctgagtaaaaaacataaatgaatcaaaactttcaacaacggatctcttggttctg
>OTU_619_22
ttacagaaagtaaacgcggatcaaaccgcgaacttctaaacctttgacgattgacttatgttgcctcggcgggttctcccgccagaggatatatcaaaactcctgttttaacggtgctgtctgagctacaagcaacgaatcaaaactttcaacaacggatctcttggctctc
>OTU_622_25
ttaccgagttagggtctaaaacaggcccgacctccaaccctttgtttactataccatgttgctttggcgggcccgcctttcggggctgccggggactttcatgcccctggtcagtgcccgccagtagccttattaaattcttccataattatgttgtctgagtataaacataaaaatcgttaaaactttcaacaacggatctcttggttctg
>OTU_624_29
ttactgaattgtcaacaagagttgttgctggtccccagatgggggcatgtgcacgctctgttaacacatccactcacaccctgtgcaccctctgtagttctatggttggggggggacctgtcccctcctgctgtggctctgcatctttacatacacactgtaacaaagtctaatggaatgcatgccgcgtctaacgcaataaaatacaactttcagcaacggatctcttggctctc
>OTU_629_4
ttaacgagttagggtcttctcggcccgacctcccaaccctgtgtttactgaacctttgttgcttcggcgggcccgtctcacgaccgccgggggaccgtaagacgtcctctggtccgtgtccgccggtggccaaactgaacaaattctgattaaaatgtgtcaatgtctgagtagaattcataattaaaacaaaactttcaacaacggatctcttggttctg
>OTU_630_8
ttaccgagtgcgggccctcgcggcccaacctcccacccttgtctctatacacctgttgctttggcgggcccaccggggccacctggtcgccgggggacgtcgtctccgggcccgcgcccgccgaagcgctctgtgaaccctgatgaagatgggctgtctgagtactatgaaaattgtcaaaactttcaacaatggatctcttggttccg
>OTU_632_7
ttacagagttcatgccctcacgggtagacctcccacccttgtgtatctataccatgttgctttggcaggctgctggacccctcgggggacagcctcagcgccctcgggcctgagagtcgcctgccggaggaaaaacaaactctgaattgttagtgtcgtctgagtactatattttaatagttaaaactttcaacaacggatctcttggttctg
>OTU_634_1
ttattgaaataaacctgatgggttgttgctggttctctagggagcatgtgcacaccttgtcatctttatacctccacctgtgcactttttgtagaccttttaggtctatgttgcttcatttaccccaaatgtatgttaatagaatgttgtgcctatataatatatacaactttcagcaacggatctcttggctctc
>OTU_635_3
ttaacgagttcaaagtcgaacgaccgtgctggcggaaacgcatgtgcacgtcggtcgcgaacccatccacacaccgtgaacctatggcctggggtcttttgactctgggcaccattttttacactctgtctgtaaaggaatgtctattatcataaaatatacaactttcaacaacggatctcttggctctc
>OTU_640_6
ttactgaaaaactttaaccagttttttcatatcccattgtttacctaccctgttgcttccattggacatgttttacctttgtggtaaaacctttggcttgtgtgaataaccctagccagagagctgccagtggtaagatcatcaaacaaaacttgattgaaacacatttgtctgaatcttttgcttcaatgaaataaaactttcaacaacggatctctaggctctt
>OTU_644_16
ttagcgagtatggccctcacgggtctaatccaccataaacacctttgtgaaccagtcagggggactcggccgtgaaagcccaggcgattagcggaagggccctcctttgcaaacccgtttagaagtcttgaaagtacttttgtataatctagtaaaacttatagcaacggatatctcggctctc
>OTU_646_145
ttatcgagttagggtcttctcaggcccgacctccttacctttgtctaccttacctcacgttgcttcggcgggcccgtcctgtttttggaccgccggaggcttcaccccctctggcccgttgcccgccgacagcccccaaccaaaaactcttgcaataaccgtggaattgtctgaatcttgcttctaaaataaccaaaaactttcaacaacggatctcttggttctg
>OTU_647_6
ttacagagaacatgccccttggggtatatctcccatcctttgtttacaatacctttgttgctttggcaggcccgtcacttgaccactggctctagctggtctgtgcctgccagagaaccctaaaattctgattatgttgtctgagtatgatgcaatgatttaaaactttcaacaacggatctcttggttctg
>OTU_648_2
ttaacgagtgagggtcttctcggcccgacctcccaaccctatgtttactgaacctttgttgcttcggcgggcccgtctcacgaccgccgggggaccgtaagacgtcctctggtccgtgtccgccggtggccaaactgaacaaattctgattaaaaatgtgtcaatgtctgagtccaattcataattaaaacaaaactttcaacaacggatctcttggttctg
>OTU_649_17
ttcataatcaagtgtttttatggcactttcaaaaatccatatccaccttgtgtgcaatgtcatctctctgggggctgccggctgtcaaaagccgtgtggtcacctttgggatttatatctactcagaactttagtgattttgtctgaaacatattatgaatacttaattcaaaatacaactttcaacaacggatctcttggctctc
>OTU_650_62
ttaccgagttagggtcttctaagggcccgacctccttacctttgtctaccttacctcacgttgcttcggcgggcccgtcctcttttggaccgccggagggttcaccccctctggcccgttgcccgccgacagccccccaaccaaaaaactcttgcaataactgtgaaattgtctgaattttgcttctaaaataaccaaaaactttcaacaacggatctcttggttctg
>OTU_651_16
ttattgtataaccgaggtgctagggctgtcgctgaccctttgaagggtcgtgcacgcccaagtgctctctcacatccatctcacccctttgtgcatcaccgcgtgggctacctttttggctttattcaaaaaggttggttcgcgtttttacacacacacctttatgtatagaatgtcttgatttttgcggtcatacgcaataaataaataaataaataatacaactttcaacaacggatctcttggctctc
>OTU_653_30
ttaccgaattgtcaacaagagttgttgctggctcctgtatgggggcatgtgcacactctgtttacacatccactcacacctgtgcaccctctgtagttctatggcctgggggggtccgtccccccgctgtggttctacgtctttacacacacactgtaataaagtctcatggaatgtatgtcgcgtttaacgcaatgaaatacaactttcagcaacggatctcttggctctc
>OTU_654_8
ttaccgaattccaaggaggaaagcaggggggatatttccagtagtatttcggtagtgctgttgctggccttcgggcatgtgcacgtctccggagttgtaatggtgttattattccccttgttccctctaatacctcttgaacacctgtgtgcacctgttgtaggtctcgtcactgggacctatgtattccacattaaactcgcatgtatacagaacgctgtctagtcgtgtcgtccggccccgagggtcggcgaccgaaaaatgataatataatataactttcagcaacggatctcttggctctc
>OTU_656_184
ttaaagagtaccggagctctcgggttcctactcccaccctatgttgactttaaatgttgctttggcggaccggctgtactgccaccggcctaggctggatagcgtccgccagaggatctttaaattccttcgaatgtgaattccgagtctttgaaaattgaatcaaaactttcaacaacggatctcttggttctg
>OTU_657_125
ttaccgagcgagcgcctccgggcgcgacctccaacccttttgtgaatccaacctctgttgcctcggggtgacccggcctcgcccggcccccggaggacgatttgacccaactctgcatctttgcgtcggatgtgaaaagtcaattgatttaaaactttcaacaacggatctcttggttctg
>OTU_659_52
ttacagagaaaattgccctttggggtagatctcccaccctttgtttacattacctttgttgctttggcaggcccgtctttggaccactggcttaggctggtctgcgcctgccaaaggaccctaaactcttgattttttgtgttgtctgagtaacattataataagttaaaactttcaacaacggatctcttggttctg
>OTU_661_1
ttactgaaaaacttctaaagttttttatatcccattgtttacttaccccgttgcttccactggacagatttcatcatgtgtgggatcttttggcttttgtgtataatacttgccaaagagtttgccagtggcaagatcttttttcaccaaaacttgattaaaacacattgtctgaatatatttcttgaatgaaacaaaactttcaacaacggatctctaggctctt
>OTU_663_2
ttattgaatatgggttgttgctggcctttgaggcatgtgcacgccttttattatttctccaacttgtgcatcatattgggactgctgtgctttatgtttagttcagctttccctttttattatcataatcacattaaaatgttgaatgtatgaaagaaacaaaataatacaactttcagcaacggatctcttggctctc
>OTU_664_1
ttactagagcaaaggatagacagcgcccgcggagctcgctcccggggctaccctactcccgtagggtttagagtcgtcgggcctctcggagaagctcggtcctgaactccacccttgaataaactacctttgttgctttggcgggccgcctcgtgccagcggcttcggctgttgagtgcccgccagaggaccccaactcttgtttttagtgatgtctgagtactatataatagttaaaactttcaacaacggatctcttggttctg
>OTU_667_67
ttacagaaagtaaacgcgggtcaaaccgtgaacttttaaacctttgacgattgactcatgttgcctcggcgggctcgcccgccagaggatacatcaaaactcctgttttaacggcgttgtctgagctacaagcaacgaatcaaaactttcaacaacggatctcttggctctc
>OTU_672_2
ttaccgaattgtcaacaagagctgttgctggtccccggatgggggcacgtgcacgctctgttaacacatccactcacaccctgtgcaccctctgtagttctatggttgggggggggacctgtcctctcctgctgtggttctgcatctttacatacacactgtaacaaagtctcatggaatgcatgctgcgtttaacgcaataaaatacaactttcagcaacggatctcttggctctc
>OTU_674_5
ttactgaaaaacttcttttcaaagttttttataacccattgtttactttaccctgttgcttccactggacagatttcatcttgtgtgggatcttttggcttgtgtgaataatacttgccaaagagttgccagtggctagactttttaaccaaaacttgattaaaacacattgtctgaaatatatttcttgaatgaaaataaaactttcaacaacggatctctaggctctt
>OTU_678_84
ttagtgattgtgaatcgttaccttcagtgctggctggttccagcaagtgcacgttggtgactttcatccaaatacccttgtgaacctttggcctcttgctggcttcggctgacagaggattttacacaccaactcgaatgtaatgaaaactactgtcgtgcgcaagcactaatgtacaactttcaacaacggatctcttggttctg
>OTU_680_408
ttattgaaatttaaatttggctgcgttgttgctggctcttaggagcatgtgcacatgcaccattttttttttttaaaaaaaaccacctgtgcacacactgtagatctggattgaactttttcgaggattattaactcggttttgagaactgctttggctgttcttgtgttctccaggtctatgttttaaacaatatactccaacaagtaatagaatgtcaaattatgggccttttgtgcctttaaaatctaatacaactttcaacaacggatctcttggctctc
>OTU_681_20
ttaaaaatatgaaccggaccggttcgctgcttgcagcgggctgcgaagggagatattatacaccctgtacttgtttacctttgttgctttggcgggccgtccgtttaggcgttggctccggccgatcgtgcccgctagaggacccaaactcttttgttagtgatgtctgagtactatataatagttaaaactttcaacaacggatctcttggttctg
>OTU_697_2
ttactgagctgtcgacacgagctgttgctggtcctcaaacaagggggcatgtgcacgctctgttcacacatccactcacacctgtgcaccctctgtagttctatggtctgggggacacacccgtcttcctcccgtagctctacgtctttacacatacaccgtagtgaagttttatggaatgtgcgccgcgtttaacgcaatacaatacaactttcagcaacggatctcttggctctc
>OTU_698_60
ttaccgagtgcgggccctcgcggcccaacctcccacccttgtctctatacacctgttgctttggcgggcccaccggggccacccggtcgccgggggacgtcgtccccgggcccgcgtccgccgaagcgccctgtgaaccctgatgaagatgggctgtctgagtactatgaaaattgtcaaaactttcaacaatggatctcttggttccg
>OTU_700_13
ttactgagttgcaaaactccaaaccattgtgttctttgctgttcttgttgcctcggcaggccgtcccagggcgggacctctgtccagcctgccgaaagcacaccataaaactcttctgaaacctgtcttctgaagcactataaaaataagtcaaaactttcaacaacggatctcttggttctg
>OTU_701_214
ttattgaaataaacctgatgagttgctgctggctctctagagagcatgtgcacacttgtcgtctttatatctccacctgtgcacctattgtagacctggatgactctctgaatggctatcattcaggtatgaggattgactttctgcctctccttacatttccaggcctatgttctttcatataacctcaatgtatgttatggaatgtaataattatggccttctgtgccttataaacctatacaactttcagcaacggatatctaggctctc
>OTU_703_40
ttaccgagtgagggccccgggcccgacctccaaccctttgttgaccaacacctgttgcctcgggggcgacccggacgccgacgcgtcggccgtcgggcaccccggtggaccattccaactctgttgtctgtacgtcggagtactttgcgaatcaatcaaaactttcaacaacggatctcttggttctg
>OTU_704_50
ttaccgagtgagggcccccggcccgacctccaaccctttgttgacctcacccgttgcctcgggggcgacccggacgccgacgcgtcggccgtcgggcaccccggtggaccattccaactctgttgtctgtacgtcggagtactttgcgaatcaatcaaaactttcaacaacggatctcttggttctg
>OTU_705_319
ttattgattgcgaatcgttgtcttcagtgctggccagctctggcaagtgcacgttggcggcttttcatccaatacccctgtgaacctttggcctcttgctggctttggctggcagaggatttttttacacccgctcgaatgtaatgaaattattgttgtgcgcaagcactactgtacaactttcaacaacggatctcttggctctc
>OTU_711_5
ttaccgagggcccctccccggcgcagcaatgcgtcgccgaaggggctattctcaccacccttttttatacgtacctcttattcttcctcggcggggcaacctgccgatggaacacacatcataacctctttgcatccagcattacttgttctgataccctaaattttacaactttcaacaatggatctcttggctctg
>OTU_716_95
ttatcgtaaaaccgaggtgcgagggctgtcgctgaccttttttggtcgtgcacgcccgagcgctctcacacaatccatctcaccccttgtgcaccaccgcgtgggttccctttctggcttgtccgaaggggggctcgcgttttcacacaaacttgaattggtgtagaatgtccttttttgcgataacacgcaattaatacaactttcaacaacggatctcttggttctg
>OTU_717_6
ttacagagaacatgccctctagggtagatctccaaccctatgtttatttacctttgttgctttggcaggcccgtctcacgaccaccggcttcagctggtcagtgcctgccggaggacccaaactctaaattttagtcgtctgagaatcataaaatagttaaaactttcaacaacggatctcttggttctg
>OTU_720_32
ttcataatcaaagtgtttttatggcacttttaaaaaaatccatatccaccttgtgtgcaatgtcatctcactggaggccagctggctgtcaaaagcccgtttggtcacctttgggatttatatctactcagaactttagtgattttgtctgaaaaatattatgaataacttaattcaaaatacaactttcaacaacggatctcttggctctc
>OTU_721_25
ttaaagagtaagggtctcacggcccgacctccaaccctctgttgttataactataccgttgcttcggcggccgcagcccgcaagggctgagccagtctcacgaccggcgagcgccgccggagtctaaccaaactcttgtttttaaccagtcgtctgagtaaaaattttaattaaattaaaactttcaacaacggatctcttggttctc
>OTU_726_356
ttaccgaattgtcaacaagagttgttgctggtcctcgaatgggggcatgtgcacgctctgttcacacatccactcacaccctgtgcaccctctgtagttctatggtcagggggcctgtcctcctgctgtggttctgcatctttacacacacacacacacagtaacaaagtctcatggaatgcatgccgcgtttaacgcaatataatacaactttcagcaacggatctcttggctctc
>OTU_727_84
ttactagtttagggtattctgtgcccaaacttcaaccctatgatttaccaacttgtttctttgccggcgtcaagccggcaaaaggattctcaaactcattgatgaaaaattgcaatctgaaaatttgatcaaaaatcaaaagcaaaactttcaacaacggatctcttggttctg
>OTU_728_81
ttactgagtgagggcctccggcccgacctccaaccctgtgtcgattccaacctctgttgcctcgggggtcacctggtcctccgctgtcagggcccccggtggaccactccaactctgcatttgagcgtccgagtccatcgttaaatgaatgaaaactttcaacaacggatctcttggttctg
>OTU_729_1
ttattgactgtgaatcgttgcctccagtgctggctccggcaagtgcacgttggtgactttcatccaacaccctgtgaacctttggcctcttgctagcttcggctggcagaggatttttataacacactcgaatgtaatgagaactattgtcgtgcgcaagcactaatgtacaactttcaacaacggatctcttggctctc
>OTU_730_133
ttaccgagtttctactctaaaccctgcgaacctacctatacgtgcttcggcggccggccccggggggcgcctaacggccgccgaaagtacccaaaaatcacttttcagagtcttctgagtgacaaaaaaaaaacaagtcaaaaccttcaacaacggatctcttggttctg
>OTU_731_1
ttattgagttaccgaactcccaaacccgtgtgaacttaccactgttgcttcggcggtcacgccccgggagccccgcgcggcccggagtcaggcgtccgccggaggaccaaaactcttgattttacagcgtctcttctgagtggattatacaaatgaatcaaaactttcaacaacggatctcttggttctg
>OTU_733_3
ttactgaattgtcaacacgagttgctgctggtcctcaaatgggggcatgtgcacgctctgttcacacatccactcacacctgtgcaccctctgtagttctatggcttgggggactctttccccattctgtggttctatatctttacacacactctgtaatgaagtctcatggaatgtacgccgcgtttaacgcaatacaatacaactttcagcaacggatctcttggctctc
>OTU_734_313
ttatagacgtgattggggccttaattggtcgtccgttactgcacccttgcctttttgcgtaccgtttcgtttcctcggcagcttcgcctgccagcgaggaccccctaaaacacttttgcaatcttaggtataacgttctgaaaaccctcaatattaaaactttcaacaatggatctcttggctctg
>OTU_736_18
ttaccgagtttacaactcccaaacccctgtgaacatacctattgttgcctcggcggtgcctgttccgacagcccgccagaggaccccaaaccctgattacatttaagaagtcttctgagtaaccgattaaataaatcaaaactttcaacaacggatatctcggctctc
>OTU_738_3
ttatcgtggggcttcggccctctcgagatagaacccttgcctttttgagtacctttcgtttcctcggcgggctcgcccgccaatggggaccacaataaaccctttgtaatacctgtaatcgtctgacaaaacaaacaaaaattaaaactttcaacaacggatctcttggttctg
>OTU_740_19
ttactgagctgtcgacacgagctgttgctggtcctcaaacaaggggggcatgtgcacgctctgttcacacatccactcacacctgtgcaccctctgtagttctatggtctgggggacacacccgtcttcctcctgtagctctacgtctttacacatacactgtagtgaagttttatggaatgtgtgccgcgtttaacgcaatacaatacaactttcagcaacggatctcttggctctc
>OTU_741_238
ttagtgaatcttcaaagtcggcccgtcggattgtgctggtggagacacatgtgcacgtctacgggtcgcaaatccacacacctgtgcatctatgactctgagtgccgctttgcatggtccttgattggacctggtgctcgagtacttttcacacactcttgaatgtaacggaatgtcaatgttgtgcacaatgtacaaacgaaacaactttcaacaacggatctcttggctctc
>OTU_742_16
ttactgagtgccggtccctcgtggaccgaacctcccacccttgtctcttgtataccctgttgctttggcgggcccactgggaatccccagtcgccgaggggcactgtgcccctgggcccgtgcccgccagagcgcccttgaaccctaatgaagatggactgtctgagcatgattgataataatcaaaactttcaacaatggatctcttggttccg
>OTU_747_9
ttaccgagttagggtcttctcagggcccgacctccttacctttgtctaccttacctcacgttgcttcggcgggcccgtcctcttttggactgccggagggttgaacccctcgggcccgttgcccgccgacagcccccaaccaaaaaactcttgcaataactgtggaattgtctgaatcttgcttctaaaataaccaaaaactttcaacaacggatctcttggttctg
>OTU_755_329
ttaccgaattgtcaacacgagctgttgctggtcctcaaacgggggcatgtgcacgctctgtttacacatccacttacacctgtgcacccttcatagttctgtggcctgggggcaccgtcccccctgctgtggtcctatggatttaacacacacactgtaaccaagtctcatggaatgtacaccgcgtttaacgcaatacaatacaactttcagcaacggatctcttggctctc
>OTU_756_71
ataccgaattgttgacacgagttgttgctggccttcaaacgggggcatgtgcacgctctgtttatacatccacccacccctgtgcaccttttgtagttctgtggtctggaggctccgcttcccttctgcggtcctacgtctttacacacacacgttaaagaagtctgtggaatgtacgccgcgtttaacgcaatacaatacaactttcagcaacggatctcttggctctc
>OTU_757_24
ttaacaaatccaataattgctggagaacacatgttctttttgcaaatattaatccgctggccgctcttagtagcagctggctgtgatataaatgcgtgttatgttgacagtgtgtgtataagttaatattgacaacttttaacaatggatctctaggctctt
>OTU_758_9
ttactgagtgcgggccctctgggtccaacctcccacccgtgtttatcgtaccttgttgcttcggcgggcacggaggcccgccggagacacctttgaacgctgtctgaaggttgcagtctgagtcgattttttaaatcattaaaactttcaacaacggatctcttggttccg
>OTU_759_65
ttaccgagtttacaactcccaaacccctgtgaacataccatttgttgcctcggcggtgcctgcttcggcagcccgccagaggacccaaacccttgattttatacagtatcttctgagtaaatgattaaataaatcaaaactttcaacaacggatctcttggctctc
>OTU_761_298
ttatcgagtgaagggttctctaagaacctgacctcccaacccttgtgttctttaccactttgctttggcgggcccgtcactgctgtgaccgccaaagggttatgtttttaactaaccttttaggcccgtgcccgccagagatatctgaacgcttgttatgaaataggttgtctgagttataaagaaaaataagtcaaaactttcaacaacggatctcttggttccg
>OTU_762_4
ttaacgaattacaaagtcggccttgctgttgctggtggaaacacatgtgcacgctcggtcgcaaatccacacacctgtgcatctatgactcgaggttgtcgtcgtatgaccgagagtaacttttacacactcttgaatgtaatggaatgtctcttgtgcataatgtacaattaaacaactttcaacaacggatctcttggctctc
>OTU_763_4
ttaccgaattccaaggaggaaagcaggggggatttccagtagtatttcggtagtgctgttgctggccttcgggcatgtgcacgtctccggagttgtaatatggttattccccttgttcctctaatacctcttgaacacctgtgtgcacctgttgtaggtctcgtcactgggacctatgtattccacattaaactcgcatgtatacagaaagctgtctagtcgtgtcgtccggccgagggtcggcgaccgaaaaatgataatataatataactttcagcaacggatctcttggctctc
>OTU_768_307
ttattgaaataaacctgacgggttgttgctggttctctagggagcatgtgcacaccttgtcatctttatatctccacctgtgcaccttttgtagacctgaaaaggtctatgttgcttcatttaccccaatgtatgttaatagaatgttgtgcctatataatatatacaactttcagcaacggatctcttggctctc
>OTU_769_41
ttaccgagtgagggccctctgggtccaacctcccacccgtgtttatcgtaccttgttgcttcggcgggcccgccgcaaggccgccggggggcttccgtccccgggtccgcgcccgccgaagacacctgtgaacgctgtatgaagattgcagtctgagcgaaaagctaaatcgttaaaactttcaacaacggatctcttggttccg
>OTU_770_4
ttacagagaaaattgccctttggggtagatctcccaccctttgtttacattacctttgttgctttggcaggcccgtctttggaccaccggcttaggctggtctgcgcctgccaaaggaccctaaactcttgatttttgtgttgtctgagtaacattataataagttaaaactttcaacaacggatctcttggttctg
>OTU_772_7
ttaaagagttagggtcttctaggcccgatctcccaaccctttgtttattgaacctctgttgcttcggcggacccgtctcacggccgccggaggaccgccgaaaggcgtcctctggccagcgtccgccgatagccaaccacttaaactctgaataaatcgtgtcatatgtctaagtctatgattaaattaaagcaaaactttcaacaacggatctcttggttctg
>OTU_773_2
ttaccgagaaattgccctttgggtagatctcccaccctttgtttacattacctttgttgctttggcaggcccgtctttggaccaccggcttaggctggtctgcgcctgccaaaggaccctaaactcttgattttttgtgttgtctgagtaatattataataagttaaaactttcaacaacggatctcttggttctg
>OTU_779_149
ttaaagagtaccggagctctcgggttcctactcccaccctatgttgactttaaatgttgctttggcggaccggcgatctcgccactggcctaggctggacagcgtccgccagaggatttttaaattcctttgactgaattctgagtcttgaaaattgaatcaaaactttcaacaacggatctcttggttctg
>OTU_780_242
ttactgaattgtcaacatgaagttgtcgctggccctcatgtgggggcatgtgcacgctctgtttacacatccattcacacctgtgcactctctgtagttctgtggtttgggggctctgtcctcctaccgtggtcctgcatatttacacatacacactgtgataaagtctcatggaatgtatgccgcgtttaacgcaatacaatataactttcagcaacggatatctaggctctc
>OTU_781_2
ttaatgattgcgaatcgttgccttctgtgctggccagccactggcaagtgcacgtcggtgactttcatccaataccctgtgaacctttggcctcttgctagctttggccggcagaggaattttacacacactcgcatgtaatgaaatatactgtcgtgcgcaagcactaatgtacaactttcaacaacggatatctcggctctc
>OTU_783_22
ttaatgattgcgaatgggtcaccttcagtgctggctctcaagagcaagtgcacgttggtggctttcatccaattccacaccctgtgaacctttggcctcttgctagcttcggctggcagaggattttacatactcgaatgtaatgaaaactcttgttgtgcgcaagcactaatatacaactttcaacaacggatctcttggttctg
>OTU_784_171
ttatcgtacaaaatgtgagagaggcatgcaagggctgtcgctgactccaagtcgtgcacgccggagtgtgccctctcacataataatccatctcaccctttgtgcaccaccgcgtgggcaccctttgggatcagactgatctcggaggatgctcgcgttttcacacaaaccccccttttaaaagtgtagagtgacctcatttatgcgctaacccgcaatcaatacaactttcaacaacggatctcttggttctg
>OTU_785_3
ttaccgaattgtcaacaagagctgttgctggtccctggatgggggcatgtgcacgctctgttaacacatccactcacaccctgtgcaccctctgtagttctatggttgggggggggacctgtcctctcctgctgtggttctgcgtctttacatacacactgtaacaaagtctcatggaatgcatgctgcgtttaacgcaataaaatacaactttcagcaacggatatctaggctctc
>OTU_791_15
ttatcgtaaaacagaggtgcgagggctgtcgctgactttctaagtcgtgcacgcccgagcgctctcacacaatccacgtcacccctatgtgcaccaccgcgtgggtcccccccttgaaaggggggctcgcgttttcacacaaacttgaagtagtgtagaatgtctttttttgcgatgacacgcaatcaatacaactttcaacaacggatctcttggctctc
>OTU_792_104
ttatcgtacaaccgaggtgcaaaggctgtcgctgaccctcaaaggtcgtgcacgcctgagcgctctcacacaatccatctcacctttgtgcatcaccgcgtgggtcccctttgcgggagggcttgcgttttcacataaaacttgatacagtgtagaatgtttttcttttgcggtcacacgcaatcaatacaactttcaacaacggatctcttggttctg
>OTU_794_13
ttatcgaataaacttgaacaggctgttttttgctggcccttgaaagagggcatgtgcacgcttgttcatctttgtcatttctccaactgtgcacatactgtagacctggggggagcattatgaatggaatttccattcgagttggggattgctgtgctttacaaaagaaaaaagtcggctttcccttgtattttttcaggtctatgttgtttttcacaatctctacaacaatgtttagaatgttgaataaggtctttgtacctatataaaagttaatacaactttcagcaacggatctcttggctctc
>OTU_800_34
ttaacgaactgtcgacacgagttgttgctggtccccaaaccgggggcatgtgcacgctctgtttacaaatccactaacacctgtgcaccctccgtagttctgtggcctgggggtctccgccctcttgccgtggttctacgtctttacacacacactgtaatgaagtcttatgggatgtatgtcgcgtttaacgcaatacaatacaactttcagcaacggatctcttggctctc
>OTU_802_124
ttatcgaataaacttgaacaggctgttgctggtccaccacctccctggctgggcatgtgcacgcttgtcatctttatcatttctccaactgtgcacctcttgtagacctggggcatgtttgaaatatgacgagtttgggactgccattcggctttgccttgtattctccaggtctatgtttcacacaaacaaaaaacttgtttagaatgtgttggcaataataataaagttatacaactttcagcaacggatatctaggctctc
>OTU_805_5
ttattgtacaacggaggtgcgagggctgtcgctgaccttcaaaggtcgtgcacgcccgagccctctcacaatccatctcaccctttgtgcatcaccgcgtgggtccccctttagcggggagggctcacgtttttacataaaactcgatgcagtgtagaatgtttatttttgcggtcacacgcaatcaatacaactttcaacaacggatctcttggttctg
>OTU_806_21
ttactgaattgtcgacacgagttgttgctggtcctcaaacgggggcacgtgcacgctctgtttacacatccactcacacctgtgcaccctctgtagttctgtggcatgggggactctgtcctcctgtcgtggttctacgtttttacacacactccgtaataaagtctcatggaatgcatgccgcgtttaacgcaatacaatacaactttcagcaacggatctcttggctctc
>OTU_811_11
ttatcgatttttaaagatgtgctggcactccttcactggtttgcatgtgcactctgcaaaatatccttctctttacccccactgtgcacacccaagtagacaaacagagttacagagcaatctgtgtaattcctgatttgcctatgtttttatcaaacaccaatgacaatctcagaatgatctatgtagtaagcttttaacaagctgaaatttaatacaactttcaacaatggatctcttggttctc
>OTU_814_1
ttaatcgaacaaacgtgcttctcggcatgatgtttcaaaccccacctgtgtatcttacctgttgcttccgtgctgcacacgctgtcaaggtgcttcagggtacggtctaccgagtacttgggacccccggtaccctggggagtcggtacgggagggataaccacaaactctttcctttgaatgccttctgtctgaactgtaatacatgaaaagttaaaactttcaacaacggatctcttggttctc
>OTU_815_2
ttaccgagttcatgccctcacgggtagatctcccaccctatgttatcattacctttgttgctttggcgggccgccaggcttcggctaggctaccggctccggctggtaagcgcccgccagaggaccctaacattctgattatcagtgtcgtctgagtactatataatagttaaaactttcaacaacggatctcttggttctg
>OTU_816_25
ttaccgagttttcaactcccaaaccccatgtgaacttataccatttacaaccgttgcttcggcgggttctcgccccgggctttacaccccggaaccaggcggcccgccgggggacccaaactctagatttttattttagcatgtctgagtggaatcattacaaaatgaatcaaaactttcaacaacggatctcttggttctg
>OTU_818_1
ttatcgaaagctctggcttttgctgtgccttgaaccctttgatgtataaacctttgttgcttccgtcggccgacgacacaaaactgcggcatcggctgatggagggtatttgagaactttggctgtttaaatgttattagtctgaaagatttgagatgaaaacgctcaaataataaaactttcaacaacggatctcttggctcct
>OTU_820_4
ttaccgaattccaaggaggaaagcaggggggatatttccagtagtatttcggtagtgctgttgctggccttcgggcatgtgcacgtctccggagttgtaatggtgttattattccccttgttccctctaatacctcttgaacacctgtgtgcacctgttgtaggtctcgtcactgggacctatgtattccacattaaactcgcatgtatacagaaagctgtctagtcgtgtcgtccggccgagggttggcgaccgaaaaatgataatataatataactttcagcaacggatctcttggctctc
>OTU_822_18
ttaccgagttagggtttcgtctgacgagcccgacctcccaaccctttgtctattataccttgtcgttgcttcggtggaccggtcgcgaccaactggtcgtgaccgccggggtcccgtccctggagagcgtccgccgacggcccaaccacaaactcttgtaccaaaccatgtcgtctgaatttacttgattaaaatcaaaaacaaaactttcaacaacggatctcttggttctg
>OTU_825_189
ttaccgagtaccggagccttcgggttcctgctcccaccctatgttgactttaaatgttgctttggcggaccggtggttctcaccgctggcctaggctggagagcgtccgccagaggattttaaactctgtttaacggtgaattctgagtcttgataaattgaatcaaaactttcaacaacggatctcttggttctg
>OTU_826_24
ttaccagagatggcgggcctccgtgcccccgtccccaacccattgttgatcaaacctttttgcctcgggggtagagcgtccgccgccgcccccggaggaccaccgaacgctgtctctgtgtgtcggagtattgattaaatgaatgaaaactttcaacaacggatctcttggttctg
>OTU_829_17
ttaccgagtgagggttccttgcgagcccaacctcccacccgtgtttaactataccgtgttgcttcggcgggcccactggggcccgttcctggtcgcctgggggcgtgaaaccccccgggcccgtgcccgccggagaccccttgaaccctgagtgaatcgagtgtcgtctgagtttgagttaaatcattaaaactttcaacaacggatctcttggttccg
>OTU_831_1
ttaccgagtgagggccctcgcgcccgacctccaaccctttgtcgatgaatactttgttgcctcggggggcgacccggattccgcgccggcgagctccccggaggaccaccagacactgcgtctctgcgtcgagtattgaatacaaatcaattaaaactttcaacaacggatctcttggttctg
>OTU_832_241
ttatcgtacaatggaggtgctggggttgtcgctgacttttgaaagggtcgtgcacacctcggtgctctcgcacataatccatctcaccccttttgtgaatcaccgcgtggaaacccccttttggctagttctgaggggggtcttccacgtttttacacagacaccctttttatgcaatgtgtagaatgtcttactttttgcgatcacacgcaatcaatacaactttcaacaacggatctcttggctctc
>OTU_833_159
ttaccagagatggcgggcccccgcgcccccgtccccaaccctttgttgaccaacaccattttgcctcgggggcagagcgtccgccgccgcccccggaggaccaccgaacgctgtctctgtacgtcggagtattgattaaatgaatgaaaactttcaacaacggatctcttggttctg
>OTU_835_95
ttattgtataaccgaggtgctagggctgtcgctgaccctttgaagggtcgtgcacgcccaagtgctctctcacatccatctcacccctttgtgcatcaccgcgtgggctacctttttggctttattcaaaaaggttggttcgcgtttttacacacacacctttatgtatagaatgtcttaatttttgcggtcatacgcaataaataaataatacaactttcaacaacggatatctaggctctc
>OTU_836_134
ttatcgtacaatggaggtgctggggttgtcgctgacctttgaaagggtcgtgcacgcctcggtgctttcgctttcacacacaatccatctcaccccttttgtgcatcaccgcgtgggggtcccttttagctagttctgaagggggctttcgcgtttttacaaacacacccttttaatgcaatatgtagaatgtcttactttttgcgatcacacgcaatcaatacaactttcaacaacggatctcttggttctg
>OTU_837_20
ttaaagagttagggtcttctaggcccgacctcccaaccctatgtttattgaacctctgttgcttcggcggacccgcctcacggccgccggaggatcgctgaaaggcgtcctctggccagcgtccgccgacagccaaccacttaaactctgaataaatcgtgtcatatgtctaagtttatgattaaattaaagcaaaactttcaacaacggatctcttggttctg
>OTU_838_20
ttaccaagagaaatctttcaacactgaaagatcttttcctttgtgctggctttgaccgtatgtaattttgggactttaaaatggttcgcaagggccggtcccaaaaacaatatatcatccttatgaaattttttctgaacaattaaacaaaatgattttaataatctgtttaaaacaactttcaacaacggatctcttggttctc
>OTU_839_101
ttattgaaataaaactgatgggttgttgctggttctctagggagcatgtgcacacttgtcatctttatatctccaccccgtgcaccttttgtagacctggatatctctctgagtgctagtcactcaggtttgagtattgacttcatgtcttttcttacatttccaggcctatgtttttccatataccccaatgtatgtcttagaatgtattcaatgggcctctgtgcctataaaatctatatacaactttcagcaacggatctcttggctctc
>OTU_840_12
ttacagtgttccctgccctcacgggtagaaacgcccacccttgtgtattatatctttgttgctttggcaggccgccttcgggtaccggctccggctggatcgtgtcggccagaggacccccaaactctgaatgttaagtgtcgtctgagtactatctaatagttaaaactttcaacaacggatctcttggttctg
>OTU_842_141
ttaccgagttcatgcccttacgggtagacctcccaccctatgttatattacctttgttgctttggcgggccgcccggcttcggccaggctaccggctccagctggtaagcgctcgccagaggacccccaactctgaatatcagtgtcgtccgagtactataaaatagttaaaactttcaacaacggatctcttggttctg
>OTU_844_42
ttaccgagttcatgcccttacgggtagatctcccaccctgtgttatcattacctttgttgctttggcgggccgccaggctccggtcaggctatcggcttcggctggtacgcgcccgccagaggaccctaacattctgattatcagtgtcgtctgagtactatataatagttaaaactttcaacaacggatatctaggctctc
>OTU_847_24
ttaccgagtttttattactcaaaaccctgcaaacctaccttaatatgcttcggtggccggccccgggggggcgctataacggtcaccgacagtattcaaaatctctgtcattgtattctgagtaacaaaaaaaaaatcaaaaccttcaacaacggatctcttggttctg
>OTU_854_2
ttaatgattgcgaatggttgccttcggtgctggctcttgcaagtgcacgttggtggctttcatccaacaccctgtgaacctttggcctcttgctagcttcggctggcagaggatttttataacacactcgaatgtaatgagaactattgtcgtgcgcaagcactaatgtacaactttcaacaacggatctcttggctctc
>OTU_855_49
ttaccgagcgagggcctcgcgcccgacctccaaccctttgaagtgaccaactctgttgccttgggggcgacccggccgccgcgtcggggcccccaacggaccacatagaccctgcgtttttgcgtcggagtttaaagaaaatcaattaaaactttcaacaacggatctcttggttcta
>OTU_857_3
ttactgaagcagttagggttgtagctgactctgtcaaaggggtactgtgctcgccctattcttctacttttccccctgtgcaccttttgtaggctatgatatccatcgtgtgtgagttcacgctcattaccggttcaagagattgctgttttattatggctgcttcttacccttcatatgcctatgtctttatacactctttaaacaagtctagaatgtcttttgtgggtctattgacctataaacttaatacaactttcaacaacggatctcttggctctc
>OTU_861_128
ttaacgaatatctatggcgttggttgtagctggctcctaggagcattgtgcacgcccgtcattcatatcatctttccacctgtgaaccatgtgtaggcctggatacccctcgctttggcaacaaagcggatgcaaggattgctgcgtcgacaaggccggctctctttgaatttccaggttctatgtcttttacacaccccatttgaatgatttagaatgtagtcaatgggctttcatgcctataaaaaactatacaactttcagcaacggatctcttggctctc
>OTU_863_3
ttaccgagttcatgcccttacgggtagacctcccaccctatgtgatattacctttgttgctttggcaggccgtcaggcttcggtccggctaccggctcaggctggtaagcgcctgccagaggaccccaaactctgaatattagtgttgtctgagtactataaaatagttaaaactttcaacaacggatctcttggttctg
>OTU_868_63
ttatcgaataaacttgaacaggctgttgctggtccaccacctccctggctgggcatgtgcacgcttgtcatctttatcatttctccaactgtgcacctcttgtagacctggggcatgtttgaaatatgacgagtttgggactgccattcggctttgccttgtattctccaggtctatgtttcacacaaacaaaaaacttgtttagaatgtgttggcaataataataaagttatacaactttcagcaacggatctcttggttctg
>OTU_872_50
ttaccaaagatggcgggcctccgtgcccccgtccccaacccattgttgaccaaaacccttttgcctcgggggcagagcgtcccccgccgcccccggaggaccatttaacgctgtctctttgcgtcggagtattgattaaataaatgaaaactttcaacaacggatctcttggttctg
>OTU_874_107
ttacagaatatacttattctccaccctatgtataccgaactctagttgcctcggcgcgcctctgcgtcagaggacctaaacgctgaaattatactgtctgagtcgctatgcaaatagtcaaaactttcaacaacggatctcttggttcgg
>OTU_877_25
ttaattattcatcggaagagtccaggtgggctgtggaggcatgggcaaccatgcttaccacaagccccttcttcatgaacatcccttctgtgtatcaaacctctgttgcttcccgcatggcgtggcctgcaagggctacactgcggggaggtcattataaactctggttgttgtattgttagtctgagtggtaaacacaataaacaagttaaaactttcaacaacggatctcttggttctc
>OTU_880_5
ttaacgagtgagggtcgtccaggcccgacctcccaaccctttgtttactgaacctctgttgcttcggcggacccgcctcacggccgccggaggattgccgacaggcgtcctctggcccgcgtccgccgacggccaaccactaaaccctgaatcaaccgtgtcgtgtgtctcagtttatgattaaattaaagcaaaactttcaacaacggatctcttggttctg
>OTU_885_36
ttaacgagttagggtcttctcggcccgacctcccaaccctatgtttgctgaacctttgttgcttcggcggacccgtctcacgaccgccgggggaccgtaagacgtcctctggcccgtgtccgccggtggcccaactgaacaaattctgattaaaattgtcaatgtctgagtagtattcataattaaaacaaaactttcaacaacggatctcttggttctg
>OTU_888_7
ttaccgaattgtcaaacacgggttgttgctggcctccaaacgggggcacgtgcacgctctgtttacacatccactcacacctgtgcaccctctgtagtcctatggttcggaagaccccgtcttccttctgtagctctacgtctttacacacacactgtagcgatgtctcatggaatgtttttatgcgtttaacgcgatacaatacaactttcagcaacggatatctaggctctc
>OTU_889_4
ttacaagaagccgaaaggctacttcaaaccatcgcgaactcgtccaagttgcttcggcggtgcggctcccccccgggggtccgcggcgccgcgcctccccggaggtgcggggcgcccgccggaggtcaaaaactcttatgtattatagtggcatctctgagtaaaacacaaataagttaaaactttcaacaacggatctcttggttctg
>OTU_890_15
ttaccgagttagggtctttcaggcccgacctccaaccctttgttgactataccatgttgctttggtgggcccgcctttagatgccgctggggactttcacatccctggtcagtgcccgccagtagccttcttaaattctattataattatgttgtctgaataaaaaatataaatcgttaaaaactttcaacaacggatctcttggttctg
>OTU_892_2
ttaccgagttcatgccctcatgggtagacctcccaccctatgttattattacctttgttgctttggcgggccgtcaggcttctggccaggctactggctccggctggtaagcgcccgccaaaggacccccccaactctgaatattagtgtcgtctgagtactatcttaatagttaaaactttcaacaacggatctcttggttctg
>OTU_898_1
ttactgaattgtcaacacgagttgttgctggtcctcaaatgggggcatgtgcacgctctgtttacacatccactcacacctgtgcaccctctgtagttctgtggctagggggactctgtccttctgctgtggttctatgtctttacacatactctgtattaaagtctcatggaatgtatcctgcgtttaacgcaatacaatacaactttcagcaacggatctcttggttctg
>OTU_900_1
ttactgattgcgaatcgttgccttctgtgctggccagacctggcaagtgcacgtcggtgactttcatccaataccctgtgaacctttggcctcttgctagcttcggccggcagaggaattttacacacactcgaatgtaatgaaatatattgtcgtgcgcaagcactaatgtacaactttcaacaacggatctcttggctctc
>OTU_903_32
ttactgaattgttaacaagagttgttgctggtccctatacgggggcatgtgcacgctctgtttacacatccactcacacctgtgcaccctctgtagttctatggcctggggggctctgtcctcctgctgtggctctacgtctttacacacactgtaacaaagtctcatggaatgtatgtcgcgtttaacgcaatgaaatacaactttcagcaacggatctcttggctctc
>OTU_905_10
ttatagagtgttaaaactccctaaacccgtgtgaacaataccttttgttgcttcggcgagtcgtcccgggcgccgctgcgtgccccggacccaggcgctcgccggaggactccaactcttgttttgcaagtggtatctctgagtgtaccaaacaaataaatcaaaactttcaacaacggatctcttggttctg
>OTU_913_217
ttaccagagatggcgggccttcgcgcccccgtccccatccctttgttgaccaaacccttttgcctcgggggcagagcgtccgccgccgcccccggaggaccgccgaacgctgtctctgtacgtcagagtattgattaaatgaatgaaaactttcaacaacggatctcttggttctg
>OTU_914_10
ttaacgagctagggtcttcttggcccgacctcccaaccctatgtttactgaacctttgttgcttcggcggacccgtctcacgaccgccgggggaccgtaagacgtcctctggcccgtgtccgccggtggccaaaccgaacaaattctgattaaaatgtgtcaatgtctgagtagaattcataattaaaacaaaactttcaacaacggatctcttggttctg
>OTU_918_32
ttatcgtacaaccgaggtgcaagggctgtcgctgaccttccacggtcgtgcacgcccgagcgctctcaccacaatccatctcacccctttgtgcatcaccgcgtgggtccccctttgcaggagggctcgcgttttcacataaaacttgacacagtctagaatgatctttttttgcggtaacacgcaatcaatacaactttcaacaacggatctcttggctctc
>OTU_922_26
ttactgaactgtcgacacgagttgttgctggtcctcgagagggggcatgtgcacactctgtttacacacccactcacacctgtgcaccctctgtagctctgtggtgtgggggccctgtcctcccactgtggttctacgtctttacacacacagtgataagtctcatggaatgtatgtagcgtttaacgcaatacaatacaactttcagcaacggatctcttggctctc
>OTU_923_18
ttaaagagtaccggagctctcgggttcctactcccaccctatgatgactttaaatgttgctttggcggaccggcaatattgccaccggcctaggctgggtagcgtccgccagaggatttttaaattcctttaaaaggtgaattctgagtctttgaaaattgaatcaaaactttcaacaacggatctcttggttctg
>OTU_925_7
ttactgaagtgagtcttcatcattgaagcttcttcccattgtgctggctttgaccgtatgttatgttggggattaaacatggttaggatttattctagccgtccccaacaattatctcatcctttattaaattgaactgaactcattttgagattacataaaaaataatcttttaatacaactttcaacaacggatctcttggttctc
>OTU_927_17
ttactgagaacatgccctttagggtatatctcccaccctttgtttacaataccattgttgctttggtgggcccgtcttatgaccaccggctttggctggtttgtgcctgccagaggaccccaaaactctttattatgtcgtctgagtactatgtaatagttaaaaactttcaacaacggatctcttggttctg
>OTU_928_15
ttactgagtgcaaactctccaaccattgtttatctacctattttgcttctccggcaggcggttccagggagggaccaagcctatggcctagcctaagcacctgccgaaggggtacctactgctctatattatactgtctgagaaaacaaaaaaaatatcaaaattttcaattgtggatctcttggttctg
>OTU_929_9
ttaacgaatggtcttggtaccttcaaaacccctcaaaccagtggacgtttaaaaacgtgcttcggcgctccggcgccgtctttatgccgtaattctgccatttgtgtctgaatgcaaagcaaagaatgaaaactttcaacaaaggatctcttggctctc
>OTU_931_26
ttcataatcaagtgtttttatggcactttcaaaaatccatatccaccttgtgtgcaatgtcatctcactgggggccaccggctgtcaaaagccgtctggtcacctttgggatttatatctactcagaactttagtgattttgtctgaaacatattatgaatacttaattcaaaatacaactttcaacaacggatctcttggctctc
>OTU_932_1
ttaacgagtaacgggtccttctgggctcgcactccaaccctttgtttacctgctctgtgcctcggcagggtgcctttcaggtacacctgccgggagagaggccgcgacagacagctggtttcggcttgcttaagacgcggcttctgccaattaaactctgtgtaaaattttagagtctgatttaaaatatatggaattaaaactttcaacaacggatctcttggttctg
>OTU_933_40
ttacagagttgcaaaactcccacaaccatcgcgaacgttacccacaactctattatctcatggtctcttctgagtacagcacataataagttaaaactttcaacaacggatctcttggttctg
>OTU_934_2
ttaccgagttagggtctaaataggcccgacctccaaccctttgtttactataccatgttgctttggcgggcccgcctttcggggctgccggggactttcatgcccctggtcagtgcccgccagtagccttattaaattcttccataattatgttgtctgagtataaacataaaatcgttaaaactttcaacaacggatctcttggttctg
>OTU_935_10
ttaccgagggcccctccccgacgcagcaatgcgtcgccgaaggggctattctcaccacccttttttatacgtacctcttattcttcctcggcggggcaacctgccgatggaacccatatcataacctctttgcatccagcattacttgttctgataccctaaattttacaactttcaacaatggatctcttggctctg
>OTU_936_53
ttaccgagtttacaactcccaaacccaatgtgaaccataccaaactgttgcctcggcggggtcacgccccgggtgcgtcgcagccccggaaccaggcgcccgccggagggaccaaccaaactcttttctgtagtcccctcgcggacgttatttcttacagctctgagcaaaaattcaaaatgaatcaaaactttcaacaacggatctcttggctctc
>OTU_944_1
ttaccgagttagggtagtcactcactgcccgacctcccaaccctgtgtctaccacactttgtcgttgcttcggcggaccggttgaccaactggtcgtgaccgccgggggttggctctgtcccccctggagagcgtccgtcgatggcccaaccacaaaatctcttgtaccgaaacgtgtcgtctgaattattgagaaatcaaaaacaaaactttcaacaacggatctcttggttctg
>OTU_946_4
ttacagagttgcaaaactccctaaaccattgtgaacgttacctaaaccgttgcttcggcgggcggccccggggtttaccccccgggcgcccctgggccccaccgcgggcgcccgccggaggtcaccaaactcttgataatttatggcctctctgagtcttctgtactgaataagtcaaaactttcaacaacggatctcttggttctg
>OTU_947_106
ttactgaattgttaacaagagttgttgctggtccctatacgggggcatgtgcacgctctgtttacacatccactcacacctgtgcaccctctgtagttctatggcctggggggctctgtcctcctgctgtggctctacgtctttacacacacactgtaacaaagtctcatggaatgtatgtcgcgtttaacgcaatgaaatacaactttcagcaacggatatctaggctctc
>OTU_949_15
ttaccgagtttacaactcccaaacccctgtgaacatacctattgttgcctcggcggtgcctgcttcggcagcccgccagaggacccaaacccttgattttatacagtatcttctgagtaaatgattaaataaatcaaaactttcaacaacggatctcttggttctg
>OTU_954_7
ttacagagttagggtcttccaggcccgacctcccaaccctttgcttattgaacctctgttgcttcggcggacccgtctcacggccgccggaggaccgctgaaaggccgtcctctggccagcgtccgccgatagcctctcacttaaaactccgaatgaatcgtgtcatatgtctaagtctatgattaaattaaagcaaaactttcaacaacggatctcttggttctg
>OTU_960_14
ttaccgagtttacaactcccaaacccctgtgaacataccacttgttgcctcggcggatcagcccgctcccggtaaaacgggacggcccgccagaggacccctaaactctgtttctatatgtaacttctgagtaaaaccataaataaatcaaaactttcaacaacggatatctcggctctc
>OTU_961_146
ttaccgaactgtcgacacgagttgttgctggccctcatgtgggggcatgtgcacgctctgtttacacatccgttcacacctgtgcactctctgtagttctgtggtttgggggctctgtcctcctaccgtggtcctgcatatttacacatacacactgtgataaagtctcatggaatgtatgccgcgtttaacgcaatacaatataactttcagcaacggatctcttggctctc
>OTU_967_9
ttaacgagtaccggtgccttcgggtgcctactcccaccaaatgtgaaccatttaactgttgctttggcggaccggtagctctctaccctccggctccggctggagagcgtccgccggaggatttttaaacccgcttaaccgtggcatctgagtcttgataaataaaaagtcaaaaaactttcaacaacggatctcttggttctg
>OTU_971_11
ttaccgagtttacaactcccaaacccaatgtgaacgttaccaaactgttgcctcggcgggaactcatgccccgggtgcgtcgcagccccggaccaaggcgcccgccggaggaccaaccaaaactctttttgtataccccctcgcgggttttttataatctgagccttctcggcgcctctcgtaggcgtttcgaaaatgaatcaaaactttcaacaacggatctcttggttctg
>OTU_976_89
ttaccgagtgagggccctctgggtccaacctcccacccgtgtctattgtaccttgttgcttcggtgggcccgccttcatggccgccggggggcatccgcccctgggcccgcgcccgccgaagacacccctgaacgctgcctgaagattgcagtctgagcagattagctaaatcagttaaaactttcaacaacggatctcttggttccg
>OTU_977_1
ttatccatctcaaaccaggtgcggtcgcggcccccgggggcttgctcccgggtggtaggggtaacaccctcacgcgccgcctgcctgtaccctctttttacgagcacctttcgttctccttcggcggggcaacctgccgctggaaccaaaacaaaaccttttttgcatctagcattacctgttctgatacaaataatcgttacaactttcaacaatggatctcttggctctg
>OTU_980_9
ttactgaagtgagtcttcatcattgaagcttcttcccattgtgctggctttgaccgtatgttatgttggggattaaacatggttagaatttattttagccgtccccaacaattatctcatcctttattaaattgaactgaactcattttgagattacataaaaaataatcttttaatacaactttcaacaacggatctcttggttctc
>OTU_982_17
ttactgagctgtcgacacgagctgttgctggtcctcaaacaagggggcatgtgcacgctctgttcacacatccactcacacctgtgcaccctctgtagttctatggtctgggggacacacccgtcttcctcctgtagctctacgtctttacacatacaccgtagtgaagttttatggaatgtgtgccgcgtttaacgcaatacaatacaactttcagcaacggatctcttggctctc
>OTU_987_45
ttaccgagtgagcgcctccaggcgcgacctccaacccttttgtgaatcgacctctgttgcctcggggtgacccgggcccccccggcccccggtggacagttccaactctgcatctttgcgtcggatatgaaaagcaaatcgaatgaaaactttcaacaacggatctcttggttctg
>OTU_989_211
ttattgaaataaacctgatgggctgttgctggctctctagggagcactgtgcacgccttgtcatctttatatctccacctgtgcactttttgtaggcctatcaggtctatgttgcttcattttacccccaatgtatgttatcagaatgttgtgccaatataataatatatacaactttcagcaacggatctcttggctctc
>OTU_991_24
ttaaagagttagggtcctctgggcccgacctcccaaccctttgtttactgaacctctgttgcttcggcggacccgtctcacgaccgccggaggatcgcccgtccaggcgtcctctggccagcgtccgccgatagccaacccttcaaactcctgaataaatcatgttatatgtctaagtctatgattaaattaaagcaaaactttcaacaacggatctcttggttctg
>OTU_994_102
ttatcgtacaaaatgtgtgaggcatgcgagggctgtagctgactcaaagtcgtgcacgccggagtgtgtcctctcacataacaatccatctctcaccctttgtgcaccaccgcgtgggcaccctccgatctcggagggggctcgcgttttcacacaaaaccccccctttaaaaagtgtagaatgacctcatttatgcaatcaatacaactttcaacaacggatctcttggctctc
>OTU_999_127
ttacagagttcatgccctccgggtagatctcccacccactgttatcattactctcgttgctttggcgggccgctgggccctgcccggccgccggctccggctggcgcgtgcccgccagaggctccacagactctgaatgttagtgtcgtccgagtaactatataatcgttaaaactttcaacaacggatctcttggttctg
>OTU_1001_4
ttactgaattgtcaacatgagttgttgctggccctcaaacaagggggcatgtgcacgctctgttcacaaatccactcacacctgtgcaccctccgtagttctatggcctggggggctctgtcctcctgctgtggttctatgtctttacacacacacactgtaataaagtcttatggaatgtatgccgcgtttaacgcaatacaatacaactttcagcaacggatctcttggctctc
>OTU_1002_11
ttattgaataaacttggaacaggctgttgctggccctcccttggagggatacgtgcacgccttgtcatctttatttctccaactgtgcacattttgtagaccttgatgtttgatggatttattttcgagttggggactgctgtgctttctcaagtcggctttcccttgtattttcaggtctatgtcattttacacaaaccaccgagtctgtttagaatgttgaatcaggtcattttgtacctataaagttaaatatacaactttcagcaacggatctcttggctctc
>OTU_1003_143
ttacagagttgaaaaactccaaacccactgtgaatctacctctgttccttcggcaggcggcccccggggggggctccgccgtcactggcgcctgccgaggggcaaccaaactctgttaccttagtgtcacctctgagcaactgaaaataagtcacaactttcaacaacggatctcttggttctg
>OTU_1004_2
ttactgaaaaactattttcaagttttttatatcccattgtttacttaccccgttgcttccactggacagatttcatcatgtgtgggatcttttggcttgtgtgtataatacttgccaaagagtttgccagtggcaagatctttttttaccaaaacttgattaaaacacatttgtctgaatatatttcttgaatgaaataaaactttcaacaacggatctctaggctctt
>OTU_1008_38
ttacagaaagtaaacgcggatcgaaccgcgaacttctaaacctttgacgattgactcgcgttgcctcggcgggttctcccgccagaggatacatcaaaactcctgttttaacggtgttgtctgagctacaagcaacgaatcaaaactttcaacaacggatatctaggctctc
>OTU_1009_46
ttcataataagtgttttatggcactttttaaatccatatccaccttgtgtgcaatgtcagttgattttctttatggagatcgaccaaacatcaacctatttttttaactctttgtctgaaaaatattatgaataaaataattcaaaatacaactttcaacaacggatctcttggctctc
>OTU_1011_18
ttaccgagtttacaactcccaaacccctgtgaacataccacttgttgcctcggcggatcagcccgctcccggtaaaacgggacggcccgccagaggacccctaaactctgtttctatatgtaacttctgagtaaccgattaaataaatcaaaactttcaacaacggatctcttggttctg
>OTU_1012_48
ttactgaattgtcaacaagagttgttgctggtccccagatgggggcatgtgcacgctctgttaacacatccactcacaccctgtgcaccctctgtagttctatggttggggggacctgtcctctcctgccgtggttctgcatctttacatacacaccgtaacaaagtcttatggaatgcatgccgcgtttaacgcaataaaatacaactttcagcaacggatatctaggctctc
>OTU_1014_70
ttactgaactgtcgacacgagttgttgctggtcctcgagagggggcatgtgcacactctgtttacacacccactcacacctgtgcaccctctgtagctctgtggtgtgggggccctgtcctcccactgtggttctacgtctttacacacacacagtgataagtctcatggaatgtatgtagcgtttaacgcaatacaatacaactttcagcaacggatatctaggctctc
>OTU_1019_11
ttaacgaatataattcggaggggttgtagctggcttcggaaacgaggcatgtgcacgctcttctgtttttcacaactcacccgtgcacctaatgtaggatgctcctctttcgggaggggggacctatgtcttcatatacctcttcgtgtagaaagtcttagaatgttttactatcggagagtcgcgacttctaggagacgcgaatctctgagataaaagttaattacaactttcagcaatggatctcttggctctc
>OTU_1020_194
ttattgaaataaacctgatgggttgttgctggctctctagggagcatgtgcacaccttgtcatctttatatctccacctgtgcactttttgtagacctttcaggtctatgttgcttcatttaccccaatgtatgttaatagaatgttgtgcctatataatatatacaactttcagcaacggatatctaggctctc
>OTU_1032_1
ttagtaattttgaaaggttgtcttctgtgctggctgcagcaagtgcacgttggtgactttcatccaacaccctgtgaacctttggcctcttgctagcttcggctggcagaggatttacataaactcgcatgtattgaggacccttcatgtgcgcaagcactaatatacaactttcaacaacggatctcttggctctc
>OTU_1033_52
ttagtgattgtgaatcgttaccttcagtgctggctggttccagcaagtgcacgttggtgactttcatccaaatacccttgtgaacctttggcctcttgctggcttcggctgacagaggattttacacaccaactcgaatgtaatgaaaactactgtcgtgcgcaagcactaatgtacaactttcaacaacggatatctaggctctc
>OTU_1034_3
ttaccgagttcatgccctcacgggtagatctcccaccctatgttatcattacctttgttgctttggcgggccgccaggcttcggctaggctaccggctccggctggtaagcgcccgccagaggaccccaaactctgaatgttagtgtcgtctgagtactatctaatagttaaaactttcaacaacggatatctaggctctc
>OTU_1037_2
ttaacgagtttagggtcttctcggcccgacctcccaaccctatgtttactgaacctttgttgcttcggcggacccgtctcacgaccgccggggggccgtaagacgtcctctggcccgtgtccgccggtggcccaactgaacaaattctgattaaaatgtgtcaatgtctgagtagaattcataatcaaaacaaaactttcaacaacggatctcttggttctg
>OTU_1042_7
ttacagagttgtaaaactcccaaacccatgtgaacatacctgttgcctcggcggcctacccggcagctaccctgtagctaccctgtagtccgccgacggatttcaaaactcttgttttcagttgtatctctgagaataaaacaaataaatcaaaactttcaacaacggatctcttggttctg
>OTU_1047_8
ttaccgagttcatgcccttacgggtagatctcccaccctgtgttatcattacctttgttgctttggcgggccgccaggctccggtcaggctatcggcttcggctggtacgcgcccgccagaggaccctaacattctgattatcagtgtcgtctgagtactatataatagttaaaactttcaacaacggatctctaggctctt
>OTU_1051_9
ttacagagttgcaaaactccctaaaccattgtgaacgttacctttaccgttgcttcggcgggcggcccgggtcctgcccggcgcccctcggccctcgcgggcgcccgccggaggaaaaccaaactattgcattgtatggcctctctgagtcttctgtactgaataagtcaaaactttcaacaacggatctcttggttctg
>OTU_1053_60
ttactgagttgttgacacgagctgttgctggtcctcaaagcaaggggggcatgtgcacgctctgttcacacatccactcacacctgtgcaccccccgtagttctatggtctggagggcttgccgtcttcttcccgtagttctacgtctttacacatacaccgtagtaaagtcttatggaatgtgcgccgcgtttaacgcaataaaatacaactttcagcaacggatatctaggctctc
>OTU_1062_1
ttattgaataaacttggttaggttgttgctggtccttaggggcatgtgcacaccgactccatttttaaccacctgtgcaccttgcgtagatctgataatctcgaggaaactcggtttgagaattgctgtgcaaacagctcttcttgtaatcaggtctatgtttctatataccccacatgaatgtattttgaatgtcgtaaatggcctttgtgcctttaaaaacaaatacaactttcaacaacggatctcttggctctc
>OTU_1064_96
ttactgagtgagggccctctgggtccaacctcccacccgtgttttattgtaccttgttgcttcggcaggcccgcctcacggccgccggggggcctctgcccccgggcccgcgcctgccgaagacaccctcgaacgctgtctgaagtttgcagtctgagcgaatagcaaaatttgttaaaactttcaacaacggatctcttggttccg
>OTU_1066_192
ttactgaactgtcgacacgagttgttgctggtcctcaaatgggggcatgtgcacgctctgtttgcatatccactcacacctgtgcaccctatgtagttctatggcttgggggaccctgtcctcctgctgtggttctatatctttacacacactctgcaataaagtcttatggaatgtataccgcgtttaacgcaatacaatacaactttcagcaacggatatctaggctctc
>OTU_1069_6
ttaaagagtaccggagctctcgggttcctactcccaccctatgttgactttgaatgttgctttggcggaccggcagtattgccaccggcctaggctggatagcgtccgccagaggatttttaaattcctttgaatgtcaactctgagtctttgaaaattgaatcaaaactttcaacaacggatctcttggttctg
>OTU_1070_5
ttaccgagttagggtcttctaggcccgacctccaaccctttgttgactataccatgttgctttggtgggcccgcctttaggggccgccggggactttgcccctggtcagtgcctaccagtagccttcttaaattcttataattgtgtcgtctgagtatagatataaattgttaaaactttcaacaacggatctcttggttctg
>OTU_1072_1
ttaacgagttagggtcttccaggcccgacctcccaaccctatgtttattgaacctctgttgcttcggcggacccgtctcacgaccgccggaggaccgccgaaaggcgtcctctggccagcgtccgccgatggccaaccacttaaaactctgaatgaatcgtgtcatatgtctaagtctatgattaaattaaagcaaaactttcaacaacggatctcttggttctg
>OTU_1073_32
ttactgagaacatgccctttagggtatatctcccaccctttgtttacaataccattgttgctttggtgggcccgtcatatgaccaccggctttggctggtttgtgcctgccagaggaccccaaaactctttattatgtcgtctgagtactatgtaatagttaaaactttcaacaacggatctcttggttctg
>OTU_1075_81
ttacagaaagtaaacgcggatcaaaccgcgaacttctaaacctttgacgattgactcatgttgcctcggcgggtcctcccgccagaggatacatcaaaactcctgttttaacggtgttgtctgagctacaagcaacgaatcaaaactttcaacaacggatctcttggctctc
>OTU_1076_6
ttactgagctgtcgacacgagctgttgctggtcctcaaacaaggggggggcatgtgcacgctctgttcacacatccactcacacctgtgcaccctctgtagttctatggtctgggggacacacccgtcttcctcctgtagctctacgtctttacacatacactgtagtgaagttttatggaatgtgtgccgcgtttaacgcaatacaatacaactttcagcaacggatctcttggctctc
>OTU_1077_18
ttatcgtacaatgggggtacccgggttgtcgctgacttttcgtcgtgcacgcccgagtgctctcacatacaaatatccatctcacccctttgtgcatcaccgcgtgcgtccccccccttcctcggagggcggggtgctcacgtttttaacatcaaacaccctagtgtagaatgttctttgcgcgatcacgcgcgaatcaatacaactttcaacaacggatctcttggctctc
>OTU_1081_25
ttaccgagttagggtttcgtctgacgagcccgacctcccaaccctttgtctattataccttgtcgttgcttcggcggaccggtcgcgaccaactggtcgtgatcgccggggcccgtccctggagagcgtccgccgatggcccaaccacaaaactcttgtaccaaaccatgtcgtctgaattacttgattaaaatcaaaaacaaaactttcaacaacggatctcttggttctg
>OTU_1085_8
ttaccgagttcatgcccttacgggtagatctcccaccctgtgttatcattacctttgttgctttggcgggccgccaggctccggtcaggctatcggcttcggctggtacgcgcccgccagaggaccctaacattctgattatcagtgtcgtctgagtactattcaatagttaaaactttcaacaacggatctcttggttctg
>OTU_1093_63
ttaccgagcgagcgtctccgggcgcgacctccaaccctttgtgaacacacctctgttgcctcggggtgacccgggctcgcccggcccccggtggaccatcaaccaactctgcatctttgcgtcggatgtgaaaaaggaaaattgattaaaactttcaacaacggatctcttggttctg
>OTU_1094_2
ttaccgagttcttacagaacccaacccttgtgtgataagctctcacccagcttttacacgggagtctcggcctaatcgccgaggcagtgtaaaaaacaaaacctctgtatcattatcacaaatttctgaagactttttaaaagagtcaaaactttcaacaacggatctcttggttctg
>OTU_1095_7
ttaccgagtgagggccctctgggtccaacctcccacccgtgtttatcgtaccttgttgcttcggcgagcccgccacttgtggccgccggggggcatctgcccccgggcccgcgcccgccgaagacaccattgaacgctgtatgaagattgcagtctgagcttattagctaaattagttaaaactttcaacaacggatctcttggttccg
>OTU_1099_13
ttaccgagttagggtagtattcactgcccgacctcccaaccctgtgtctaccacactttgtcgttgcttcggcggaccggttgaccaactggtcatgaccgccgggggctggctctgtcccccctggagagcgtccgccgatggcccaaccataaaactcttgtaccgaaacgtgtcgtctgaattattgagaaatcaaaaacaaaactttcaacaacggatctcttggttctg
>OTU_1100_19
ttaccgagtgagggccctctgggtccaacctcccacccgtgtttatcgtaccttgttgcttcggcgggcccgccgcaaggccgccggggggcatctgccctctggcccgcgcccgccgaagacaccattgaacgctgtctgaagattgcagtctgagcaattagttaaataacttaaaactttcaacaacggatctcttggttccg
>OTU_1101_3
ttacagagttgcaaaactccctaaaccattgtgaacgttacccaaaccgttgcttcggcgggcggcgcccagcgcccccccggcccctcgcggggcgcccgccggaggtaccccaaaccattgatactttatggcctctctgagtcttctgtactgaataagtcaaaactttcaacaacggatctcttggttctg
>OTU_1102_157
ttaccaattcaacggtgtggtcgcggcctccgggggcttgcccccgggcggtagaggtaacactctcacgcgccacatgtctgaatcctttttttacgagcacccttcgttctccttcggtggggcaacctgccgttggaacttaccaaaaccttttttgcatctagcattacctgttcagatacaaacaatcgttacaactttcaacaatggatctcttggctctg
>OTU_1109_5
ttcataataagtgttttatggcactttttaaatccatatccaccttgtgtgcaatgtcagtcgttctcttatgagaatgaccaaacatcaacctattttttaactctttgtctgaaaaatattatgaataaataattcaaaatacaactttcaacaacggatctcttggctctc
>OTU_1114_19
ttcataatcaagtgtttttatggcactttaaaaatccatatccaccttgtgtgcaatgtcatctcactggaaggtcacggctgtaaaaatccggcggccaacctttgggatttatatctactcagaactttagtgattttgtctgaaacatattatgaataacttaattcaaaatacaactttcaacaacggatctcttggctctc
>OTU_1118_3
ttacaagtgaccccggtctaaccaccgggatgttcataaccctttgttgtccgactctgttgcctccggggcgaccctgccttcgggcgggggctccgggtggacacttcaaactcttgcgtaactttgcagtctgagtaaacttaattaataaattaaaacttttaacaacggatctcttggttctg
>OTU_1120_56
ttaccgagttcatgcccttacgggtagatctcccaccctatgttatcattacctttgttgctttggcgggccgccaggctccggtcaggctatcggcttcggctggtacgcgcccgccagaggaccctaacattctgattatcagtgtcgtctgagtactatataataatagttaaaactttcaacaacggatctcttggctctc
>OTU_1123_126
ttaccgaactgtcgacacgagctgttgctggccctcgaaaggggacatgtgcacgctctgtctacacatccactcacacctgtgcaccctctgtagttccatggccaggggaccccgtccccttgctgcggtcctacgtatttacacacacacagtaacaaagtctcatggaatgcggtcgcgtttaacgcaatacaatacaactttcagcaacggatctcttggctctc
>OTU_1124_21
ttacagagttctcgccctcgcgggtagatctcccacccactgttatcgttactatcgttgctttggcgggccgccgggtcctgcccggcccccggccccggctggggtgcgcccgccagaggccctacaaactctgaatgtcagtgtcgtctgagtactatacaatagttaaaactttcaacaacggatctcttggttctg
>OTU_1129_122
ttacagagttgaaagactccaaacccactgtgaatatacctctgttccttcggcaggcggcccccgggggggctgtgccgtggccggcgcctgccgaggggcaaaccaaaactctgctaccttatagttactctgagcaactgaaaataagtcacaactttcaacaacggatctcttggttctg
>OTU_1130_4
ttacagagttcatgcccttcggggtagatctcccacccttgtgtatcattataagaatgttgctttggcgggccgcgcctcgtgcgcctagattcgcgtctagcgtgcccgccagaggacccctaaactctgaatgttagtgtcgtctgagtactattaaatagttaaaactttcaacaacggatctcttggttctg
>OTU_1132_1
ttaaagaacgccccgttttttgaaatgggtaattcccaaaccgtgtctacatacctttgttgctttggcaggccgctttctaggcgttggctctggctgactgcgcctgccagaggacccaaactcgtttgtttagtgtcgtctgagtactatataatagttaaaactttcaacaacggatctcttggttctg
>OTU_1133_17
ttaccgagtttacaactcccaaacccctgtgaacataccatttgttgcctcggcggtgcctgcttcggcagcccgccagaggacccaaacccttgattttatacagtatcttctgagtaaccgattaaataaatcaaaactttcaacaacggatctcttggttctg
>OTU_1136_39
ttaaagagttagggtcctctgggcccgacctcccaaccctctgtttaccgaacctctgttgcttcggcggacccgtctcacgaccgccggaggaccgcccccccaaggcgtcctctggccagcgtccgccgatagccaaccttcaaaactcctgaataaatcatgttatatgtctaagtcttatgattcaaatcaataaagcaaaactttcaacaacggatctcttggttctg
>OTU_1137_71
ttaacgagttagggtcttccaggcccgacctcccaaccctatgtttattgaacctctgttgcttcggcgcgcccgtctcacgaccgccggaggagtcgccgcgaggcgccctctggccagcgcccgccgacggccaacccctcaaaaactctgaatgaatcgtgtcttgatgtctaagtctatgataaaataaaagcaaaactttcaacaacggatctcttggttctg
>OTU_1143_177
ttcacttgataaaaaggcttctgaagcctttttctctctttcttaaccaagtggggttcttgaattaccactgtgatgctgagatatgggtcttgaagccctttgtgattgctttcaggcatctgatcttatccagcaaaaggttcattgtaaaacccgactgtgagtttgggagtattggaccatatttataatatcactcagttgttggtcaagttgaaaagcaatccactcttggaggggagagaaggatattggttgagggtttgtagcccaaggttgtattctatcttgtctctctcttgagtctagagaactcgagataattgtccatattgtggaattatcataacatgagctcatttacaactttcagcaatggatctcttggttctc
>OTU_1145_147
ttatcatacaaccgaggtgcaagggctgtcgctgaccttcaaaggttgtgcacgcccaagcactctcacacatccatctcaccccctttgtgcatcaccgcgtgggcccccctttgcaggagggcttgcgttttcacataaaactcgatacagtgtagaatgtttttcttttgcggtcacacgcaatcaatacaactttcaacaacggatctcttggctctc
>OTU_1149_21
ttacagagttcatgcccttacagggtagatctcccacccttgaatactatacctttgttgctttggcgggccgcttcggctactggctccggctggtgagtgcccgccaaaggatcccaaaactctgaatattttgtcgtctgagtactatataatagttaaaactttcaacaacggatctcttggttctg
>OTU_1152_30
ttaccgagttagggtcttctaggcccgacctccaaccctttgttaactataccacgttgctttggtgggcccgcctttaggggccgccggggacttctgtacctggtcagtgcccgccagtagccttcttaaattccttcataattgtgtcgtctgagtatacttataaatcgttaaaactttcaacaacggatctcttggttctg
>OTU_1154_20
ttcataatcaaagtgtttttatggcacttttaaaaaaatccatatccaccttgtgtgcaatgtcatctcactggaggtcagcggctgtcaaaagcccgtttggtcacctttgggatttatatctactcagaactttagtgattttgtctgaaaaatattatgaataacttaattcaaaatacaactttcaacaacggatctcttggctctc
>OTU_1155_1
ttacgagagtgtcaccactcccaacccactgtttacctacccgtccaccgtgcttcggcaggcagtcctgtgggacagggcctcgcccccgcgagggggtgcctgccgctggccaaccaaaaattctagctgtttttgtaccatctgagtcttccacaaataaacaaaactttcaacaacggatctcttggttctg
>OTU_1156_17
ttattgaattaaacatgaagcaggttgttgctggctctctgcatgtgcacacttgtttctgctttatttctccaactgtgcacacattgtagatcgtttttggtctatgcttttcacaatctctgaatgtattagaatgcttttgaatctggggaatatacatacaactttcagcaacggatctcttggctctc
>OTU_1161_1
ttaccgaactgtcgacacgagttgttgctggtccccgaatgggggcatgtgcacgctctgtttgtacatccactcacacctgtgcaccctccatagttctgcagcctgggggctctgtcccccctgctgtggccctatgtatttacacatacacactgtaatgaagtctcatggaatgtgtgacgcgtttaacgcaatacaatacaactttcagcaacggatctcttggctctc
>OTU_1168_3
ttaccgaattccaaggagcataggaaggggggatgaccaccagtattccggagtgctgtcgctggccttcgggcaatgtgcacgtctcccgagtccgacgggtatcccctgccgaagctccgatacctctcgaacacccgtgtgcacctgttgtaggtctcgtcagtgggacctatgtattccattataaactcgcatgtatacagaacgttgtctagtcataatataactttcagcaacggatctcttggctctc
>OTU_1172_6
ttacacattcggggcgcttcggcgctccttatacacccaccctctgcctacgtgtacctctatagcttcctcggcgggctcgcccgccgccaggaacccacgaaaccccttgcattatacgcgaaaacttctgataacaaacctaaattatcacaactttcaacaatggatctcttggttctg
>OTU_1175_1
ttaccgagggcccctccccgacgcagcaatgcgtcgccgaaggggctattctcaccacccttttttatacgtacctcttattcttcctcggcggggcaacctgccgatggaacacccatcataacctctttgcatccagcattacttgttctgataccctaaattttacaactttcaacaatggatctcttggctctg
>OTU_1178_34
ttattgactgtgaatcgttgcctccagtgctggctccggcaagtgcacgttggtgactttcatccaacaccctgtgaacctttggcctcttgctcgcttcggctggcagaggatttttacacccactcgaatgtaatgaaattattgtcgtgcgcaagcactaatgtacaactttcaacaacggatctcttggttctg
>OTU_1179_5
ttactgagaacatgccctttagggtatatctcccaccctttgtttacaataccattgttgctttggtgggcccgtcttatgactaccggctttggctggtttgtgcctgccagaggaccccaaaactctttattatgtcgtcagagtactattgtaatagttaaaaactttcaacaacggatctcttggttctg
>OTU_1181_1
ttaacgagttagggtcttctcggcccgacctctcaaccctttgtttactgaacctttgttgcttcggcgggcccgtctcacgaccgccgggggactgtaagacgtcctctggcccgtgtccgccggtggccaaaccgaacaaattctgattaaaatgtgtcaatgtctgagtagaattcataattaaaacaaaactttcaacaacggatctcttggttctg
>OTU_1187_1
ttacagagttgcaaaactcccaaccctttgtgaacgaacctaccagttgcctcggcggcgagcctttcaggcggaccgccggaggaatacaaactctttgcattttagcatctctgattacttattcaataagttaaaactttcaacaacggatctcttggttctg
>OTU_1188_102
ttaacgagttagggtcttctcggcccgacctcccaaccctatgtttactgaacctttgttgcttcggcggacccgttcttacgaccgccgggggaccgtaagacgtcctctggcccgtgtccgccggtggcccaactgaacaaattctgattaaaatgtgtcaatgtctgagtagaattcataattaaaacaaaactttcaacaacggatctcttggctctc
>OTU_1190_42
ttaaagagtaccggagctctcgggctcctactcccaccctatgttgactttaaatgttgctttggcggaccggcaatcttgccaccggcctaggctggacagcgtccgccggaggatttttaaattcctttgactgtgaactccgagtctttgaaaattgaatcaaaactttcaacaacggatctcttggttctg
>OTU_1192_2
ttacagagttgcaaaactcccaaaccattgtgaacgttacctaaaccgttgcttcggcgggcggcccgggtccttcccggcgcccctcggccctcgcgggcgcccgccggaggaaaaccaaactattgcattgtatggcctctctgagtcttctgtactgaataagtcaaaactttcaacaacggatctcttggttctg
>OTU_1194_28
ttaaagagtaagggtcttctaggcccgatctcccaaccctttgtttattgaacctctgttgcttcggcggatccgtctcacggccgccggaggaccgctgaaaggcgtcctctggccagcatccgccgatagccaaccacttaaactctgaataaatcgtgtcatatgtctaagtctatgattaaattaaagcaaaactttcaacaacggatctcttggctctc
>OTU_1196_51
ttattgtataaccgaggtgctagggctgtcgctgaccctttgaagggtcgtgcacgcccaagtgctctctcacatccatctcacccctttgtgcatcaccgcgtgggctacctttttggctttattcaaaaaggttggttcgcgtttttacacacacctttatgtatagaatgtcttaatttttgcggtcatacgcaataaataaataatacaactttcaacaacggatctcttggctctc
>OTU_1197_60
ttaccgagttagggtcttctcaggcccgacctccttacctttgtctaccttacctcacgttgcttcggcgggcccgtcctcttttggaccgccggagggttcaccccctctggcccgttgcccgccgacagccccccaaccaaaaaactcttgcaataactgtgaaattgtctgaattttgcttctaaaataaccaaaaactttcaacaacggatctcttggctctc
>OTU_1198_2
ttactgaattgtcaacatgaagttgttgctggccctcatgtgggggcatgtgcacgctctgtttacacatccgttcacacctgtgcactctctgtagttctgtggtttgggggctctgtcctcctaccgtggtcctgcatatttacacatacacactgtgataaagtctcatggaatgcatgacgcgtttaacgcaatacaatacaactttcagcaacggatctcttggctctc
>OTU_1200_2
ttaacgagttagggtctcctcggcccgacctcccaaccctatgtttactgaacctttgttgcttcggcggacccgttcttacgaccgccgggggaccgtaagcaactgaacaaattctgattaaaatgtgtcaatgtctgagtagaattcataattaaaacaaaactttcaacaacggatctcttggttctg
>OTU_1203_60
ttactgaattgtcaacatgaagttgtcgctggccctcatgtgggggcatgtgcacgctctgtttacacatccattcacacctgtgcactctctgtagttctgtggtttgggggctctgtcctcctaccgtggtcctgcatatttacacatacactgtaataaagtcttatggaatgtatgccgcgtttaacgcaatacaatacaactttcagcaacggatctcttggctctc
>OTU_1205_1
ttactgagttagggtgctctctgcgcccgacctccaaccctttgtctacctgaccagatgttgcttcggcaggcccgtcgccctttcacgggggtggccgccgggggtcctttaccggtcccgggcccgcgcctgtcgatggccctcataaactcttgcttaaaacgtgtcgtctgagtttaacaaacaaaataaaaacaaaactttcaacaacggatctcttggttctg
>OTU_1210_4
ttaccgagtttacaactcccaaacccctgtgaacatacctatttgttgcctcggcggtgcctgttccgacagcccgccagaggaccccaaaccctgattacatttaagaagtcttctgagtaaaccgattaaataaatcaaaactttcaacaacggatctcttggctctc
>OTU_1212_7
ttatagtgttccctgccctcacgggtagaaacgcccacccttgtgtattatatctttgttgctttggcaggccgccttcgggcaccggctccggctggatcgtgtcggccagagaacccccaaattctgaatgttagtgtcgtctgagtactatctaatagttaaaactttcaacaacggatctcttggttctg
>OTU_1215_5
ttaacgagttagggtcttctaggcccgacctcccaaccctatgtttattgaacctctgttgcttcggcgggcccgtctcacggccgccggaggagtcgccgcgaggcaccctctggccagcgcccgccggtagccaaccccctaaactctgaataaatcgtgtcatgatgtctgagtctatgattaaattaaagcaaaactttcaacaacggatctcttggttctg
>OTU_1216_57
ttaccaaaagataatctttcaacattgaaagatcttacctttgtgctggctttgacagttttgtacttttggggctttaaaatggttcagtagttaaaggagggagcaatccctttttttcatgctactgggtcggccccaaataatcatatcatccttaaaaatttttctgattaattaacacatgattttaataatctgtttaaaacaactttcaacaacggatctcttggttctc
>OTU_1217_1
ttactgaagcaatgttagggttgtagctgactctgtcaaaggagtactgtgctcgccctattcttctacttttccccctgtgcaccttttgtaggctatgatatccatcgtgtgtgagttcacgctcattaccggttcaagagattgctgttttattatggctgctcttacccttcatatgcctatgtctttatacactctttaaacaagtctagaatgtcttttgtgggtctattgacctataaacttaatacaactttcaacaacggatctcttggctctc
>OTU_1220_2
ttaaagagtaccggagccctcgggttcctactcccaccctatgttgactttaaatgttgctttggcggaccggcaatcttgccactggcctaggctagacagcgtccgccggaggatttttaaattcctttgactgaatttctgagtctttgaaaattgaatcaaaactttcaacaacggatctcttggttctg
>OTU_1221_1
ttaccgaaccgtcgacacgagttgctgctggccctcaaaaggggcatgtgcgcgctctgtttacacatccactcacacctgtgcaccctctgtagttctatggccttggggaccctgtccccttgccgtggtcctacgtctttacacacacgccgtaacaaagtctcgtggaatgtatgtcgcgtttaacgcaatacaatacaactttcagcaacggatctcttggttctg
>OTU_1232_25
ttactgagaatgctctccggagccctcaaatcttgcgtacccatcttgtacttgcgggtggccctcgggcgcccgcgaggattcaaactgtttgactgtcgtctgattcttacaaaagaaaacaactttcaacaatggatctcttggctccg
>OTU_1236_8
ttacagagtttaaccactcaaaaccattgtgaaccttacctcaaccgttgcttcggcgggcgggccccaaaagcccccccggcccctcgcgggcgcccgccggaggtaaatcgaacccttgaattgtatggcctctctgagtcttctgtactgaataagtcaaaactttcaacaacggatctcttggttctg
>OTU_1239_129
ttactgaagcagttagggttgtagctgactctgtcaaaggagtactgtgctcgccctattcttctacttttccccctgtgcaccttttgtaggctatgatatccatcgtgtgtgagttcacgctcattaccggttcaagagattgctgttttattatggctgctcttacccttcatatgcctatgtctttatacactctttaaacaagtctagaatgtcttttgtgggtctattgacctataaacttaatacaactttcaacaacggatctcttggctctc
>OTU_1240_23
ttaccgaattgtcaacacgagttgttgctggtcctcaaacggggacatgtgcacgctctgtttacacatccactcacacctgtgcaccctccatagttctgcagcctgggggctctgtccccctgatgcggttctatgtatttacacacacactgtagtaaagtctcatggaatgcataccgcgtttaacgcaatataatacaactttcagcaacggatctcttggctctc
>OTU_1241_73
ttaacgagttagggtcttctcggcccgacctcccaaccctttgtttatcgaacctctgttgcttcggcggatccgtccctcgggaccgccggaggatcgtcgtaaggcgtcctctggcccgtatccgtcgatagccaaccacttaaactcagaataaatcgtgttataattgtctaagtttatcataaaaattaaagcgaaaactttcaacaacggatctcttggttctg
>OTU_1242_2
ttattgaatctagagttggttgtcgctggccccagagcaatgtgcacgccttctcttcttcatccacacacacctgtgcacttgtgagacggaggatttgaaaaagtcctccgcctacttaatcacacaaactcattgtacttaaactgaatgtaattgatgtaacgcatcattagaacaaagtttcaacaacggatctcttggctctc
>OTU_1246_4
ttaccgagtgagggccctctgggtccaacctcccacccgtgtttatcgtaccttgttgcttcggcgggcccgccgccaggccgccggggggcagcagcccccgggcccgcgcccgccgaagacacctgtgaacgctgtatgaagattgcagtctgagcgattagctaaatcggttaaaactttcaacaacggatctcttggttccg
>OTU_1247_2
ttactgaattgtcaaacgggttgttgctggcctccaaacgggggcacgtgcacgctctgtttacacatccactcacacctgtgcaccctctgtagtcctatggttcggaagaccccgtcttccttctgtagctctacgtctttacacacacactgtagcgatgtctcatggaatgtttttatgcgtttaacgcgatacaatacaactttcagcaacggatctcttggctctc
>OTU_1255_1
ttactgaattgttaacaagagttgttgctggtccctatacgggggcatgtgcacgctctgtttacacatccactcacacctgtgcaccctctgtagttctgtggcatgggggactctgtcctcctgttatggtcctacgtctttacacacactctgtaacaaagtctcatggaatgtatgtcgcgtttaacgcaatgaaatacaactttcagcaacggatctcttggctctc
>OTU_1257_142
ttaccgatttgttgacacgagctgttgctggtcctcaaacatgggggcatgtgcacgctctgttcacacatccactcacacctgtgcaccctccgtagttctatggtctgggggacaccctgtcctcctcccgtagttctatgtctttacatgtacaccgtagcaaagtcttatggaatgtgcaccgcgtttaacgcaatacaatacaactttcagcaacggatctcttggctctc
>OTU_1259_39
ttatcgtacaatggaggtgctggggttgtcgctgacctttgaaagggtcgtgcacgcctcggtgctttcgctttcacacacaatccatctcaccccttttgtgcatcaccgcgtgggggtcccttttagctagttctgaagggggctttcgcgtttttacaaacacacccttttaatgcaatatgtagaatgtcttactttttgcgatcacacgcaatcaatacaactttcaacaacggatatctaggctctc
>OTU_1267_6
ttactgaattgtcaacaagagttgttgctggtccccagatgggggcatgtgcacgctctgttaacacatccactcacaccctgtgcaccctctgtagttctatggttggggggacctgtcctctcctgccgtggttctgcatctttacatacacaccgtaacaaagtcttatggaatgcatgccgcgtttaacgcaataaaatacaactttcagcaacggatctcttggttctg
>OTU_1268_24
ttatcgatttttaaagatgtgctggcactccttcactggtttgcatgtgcactctgcaaaatatccttctcttaccccactgtgcacacccaagtagacaaacagagttacagagcaatctgtgtaattcctgatttgcctatgtttttatcaaacaccaatgacaatctcagaatgatctatgtagtaagcttttaacaagctgaaatttaatacaactttcaacaatggatctcttggttctc
>OTU_1272_1
ttaatgattacgaacggttgccttcagtgctggctcttaggagcaagtgcacgttggtggctttcatccaacaccctgtgaacctttggcctcttgctagcttcggctggcagaggatttttataacacactcgaatgtaatgagaactattgtcgtgcgcaagcactaatgtacaactttcaacaacggatatctaggctctc
>OTU_1273_7
ttattgaaatgttttgaaggggggactgttgctggccttgtaacaaaggcatgtgcacgtttcctttcaatctgttcatccacctgtgcacctttctgtagaagcttttttcaggtcgttgttaggggctgtacttcagtgcagctctgttgatgatcctgggcttctatgtcttacaaactctaataaaatgtacttgaatgtcttttttattggtacttaactggacctttaaaacttatacaactttcagcaacggatctcttggctctc
>OTU_1278_8
ttactgagttagggtcttataggcccgatctccaaccctttgttaactataccatgttgctttggcgggcccgcctttcagggccaccgggggttcatacccttggtcagtgcccgccagtagccttattaaattctttcataattatgtttgtctgagttataaaattaatcgttaaaactttcaacaacggatctcttggttctg
>OTU_1281_1
ttacagagttcatgcccttcggggtagatctcccacccttgtgtatcattatagaatgttgctttggcgggccgcgtgcctagcacgcctcgattcgcgtcgagcgtgtgcccgccagaggacccctaaactctgaatgttaatgtcgtctgagtactatataatagttaaaactttcaacaacggatctcttggttctg
>OTU_1282_18
ttaccgagtttacaactcccaaacccctgtgaacatacctattgttgcctcggcggtgcctgttccgacagcccgccagaggaccccaaaccctgattacatttaagaagtcttctgagtaaatgattaaataaatcaaaactttcaacaacggatctcttggttctg
>OTU_1283_2
ttaacgagttagggtctcctcggcccgacctcccaaccctatgtttactgaacctttgttgcttcggcggacccgttcttacgaccgccgggggaccgtaagacgtcctctggcccgtgtccgccggtggcccaactgaacaaattctgattaaaactgtgtcaatgtctgagtacaattcataattagaacaaaactttcaacaacggatctcttggttctg
>OTU_1291_125
ttacagagttacctaactccaaacccatgtgaacttacctatgttgcctcggcggggaaagcctacccggtacctaccctgtagctacccgggagcgagctaccctgtagcccgctgcaggcctacccgccggtggacgcttaaactcttgttttttacgtgattatctgagtgttttatacgtaataagttaaaactttcaacaacggatctcttggttctg
>OTU_1292_5
ttaccgaatcgtcaaacacgggttgttgctggcctccaaacgggggcacgtgcacgctctgtttacgcatccactcacacctgtgcaccctctgtagtcctatggttcggaagaccccgtcttccttctgtagctctacgtctttacacacacactgtagcgatgtctcatggaatgtttttatgcgtttaatgcgatacaatacaactttcagcaacggatatctaggctctc
>OTU_1301_9
ttacagagaacatgcccctcggggtatatctcccaccctttgtttacaatacttttgttgctttggcaggcccgtctctgggccgccggctagttgctggtcagtgcctgccagaggatcttaagattcttataatttattgtctgatttatttgcaattattaaaactttcaacaacggatctcttggttctg
>OTU_1304_5
ttactgaactgtcgacacgagttgttgctggtcctcaaatgggggcatgtgcacgctctgtttacacatccactcacacctgtgcaccctctgtagttctgtggctagggggactctgtccttctgctgtggttctatgtctttacacatactctgtattaaagtctcatggaatgtatcctgcgtttaacgcaatacaatacaactttcagcaacggatctcttggctctc
>OTU_1311_8
ttacagagttagggtcttctaggcccgacctcccaaccctatgtttattgaacctctgttgcttcggcggacccgcctcacggccgccggaggaccgctgaaaatcgtcctctggccagcgtctgtcgacagccaaccacttaaactctgaatgaatcgtgtcatatgtctaagtctatgattaaattaaagcaaaactttcaacaacggatctcttggttctg
>OTU_1313_19
ttatcgtacaatggaggtgctggggttgtcgctgacttttgaaatggtcgtgcacacctcggtgctctcatatacaatccatctcacccctcttgtgcatcaccgcgtggaggccccttttggctagttctaaagggggttttcacgtttttatacatacaccattttaatgcaatgcgtagaatgtcatacttttgcgatcacacgcaattaatacaactttcaacaacggatctcttggctctc
>OTU_1315_1
ttaccgaactgtcgacacgagttgttgctggtcctcaaacgggggcatgtgcacactctgtttgcatatccactcacacctgtgcaccctccatagttctatagccctgggggccttaaacccccagctgtggtcctatgtatttacacatacactgtaataaagtcttatggaatgtatgccgcgtttaacgcaatacaatacaactttcagcaacggatctcttggctctc
>OTU_1316_104
ttatcgaaaagctccggctttttattgttgccataccttgacccccatttgttgacttaacctttgttgcttccgtcggccgaaatgcccaaccaacttatggcggcggtggcatcggccggcggagggtttattttgggaaaccagtatgttttagatgcttttagtctgaaaatgttttacagagatgaaattctcaaaaacaaaactttcaacaacggatctcttggctcct
>OTU_1319_85
ttaaaagtaaatgcgtattccttgcgaatatacatatacaaacccattccgagtacatttcctgttgcttccgtggagcagtgattcgtcacctctgaagatggctctggccacctgaggggagtccccacgggaggtatacattaaactcttgcattaccatgtcttctgtctgaatctgtttataacaaatgttaaaactttcaacaacggatctcttggttctc
>OTU_1328_6
ttaccgagttcatgccctcacgggtagatctcccaccctatgttatcattacctttgttgctttggcgggccgccaggcttcggctaggctaccggctccggctggtaagcgcccgccagaggaccccaaactctgaatgttagtgtcgtctgagtactatataataatagttaaaactttcaacaacggatctcttggttctg
>OTU_1338_50
ttagtgaatcttcaaagtcggctcgtcggattgtgctggtgggaaaccacatgtgcacatctacgggtcgcaaacccacacacctgtgcatctatgactctgagcgccgctttgcatggccccttgatttgggcctggtgctcgagtactttcacacactctcgaatgtaatggaatgtcttgttgtgcataacgtacaaacagaaacaactttcaacaacggatctcttggctctc
>OTU_1340_8
ttagtgaacgcccttttgggcttataactatccaaacctctgtgaactgtgcccttcggggctttttaatacaaacactgtgtaatgaacgtaattattttaacaaaacaaaactttcaacaacggatctcttggctctc
>OTU_1346_11
ttacagagttcatgcccttacgggtagatctcccacccttgaatactatacctttgttgctttggcgggccgcctcggctaccggcttcggctggtaagtgcccgccagaggaccctaaactctgaatgttaatgtcgtctgagtactatataatagttaaaactttcaacaacggatctcttggttctg
>OTU_1347_5
ttaccgagttcatgccctcacgggtagatctcccaccctatgttatcattacctttgttgctttggcgggccgccaggcttcggctaggctaccggctccggctggtaagcgcccgccagaggaccctaacattctgattatcagtgtcgtctgagtactatataataatagttaaaactttcaacaacggatctcttggttctg
>OTU_1348_32
ttaccgagtttacaactcccaaacccaatgtgaacgttaccaaactgttgcctcggcgggatctctgccccgggtgcgtcgcagccccggaccaaggcgcccgccggaggaccaaccaaaactcttattgtataccccctcgcgggttttttactatctgagccatctcggcgcccctcgtgggcgtttcgaaaatgaatcaaaactttcaacaacggatctcttggttctg
>OTU_1349_9
ttacagagttcatgccctcacgggtagatctcccacccttgaatattatacttttgttgctttggcgggccgcttcggctgctggctctagctggtgagtgcccgccaaaggatcctaaaactctgaatatttgtgtcgtctgagtactatataatagttaaaactttcaacaacggatctcttggttctg
>OTU_1350_18
ttactgaattgtcgacacgagttgttgctggtcctcaaatgggggcacgtgcacgctctgtttgcacatccactcacacctgtgcaccctccgtagttctgtggcatgggggactctgtcctcctgttgtggttctacgtttttacacacactccgtaataaagtctcatggaatgcatgccgcgtttaacgcaatacaatacaactttcagcaacggatctcttggctctc
>OTU_1352_1
ttactgagttgtcgacatgagctgttgctggtcctcaaaacaagggggcacgtgcacgctctgttcacacatccactcacacctgtgcacccaccgtagttctatggtccagaggacttatcgtcctcttcccgtggttctacgtctttacatatacaccttagtgaagtcttatggaatgtgcgccgcgtttaacgcaatacaatacaactttcaacaacggatctcttggttctg
>OTU_1353_3
ttaccgaattgtcaaacacgggttgttgctggcctccaaacgggggcacgtgcacgctctgtttacacatccactcacacctgtgcaccctctgtagtcctatggttcggaagaccccgtcttccttctgtagctctacgtctttacacacactgtagcgatgtctcatggaatgtttttatgcgtttaacgcgatacaatacaactttcagcaacggatctcttggctctc
>OTU_1362_13
ttacagagttgcaaaactcccaaaccattgtgaacgttacctatcccgttgcttcggcgggcggcccgggccccgtgcccggcgccccccggcccctcgcgggcgcccgccggaggtaaaccaaacccttgaattgtatggcctctctgagtcttctgtactgaataagtcaaaactttcaacaacggatctcttggttctg
>OTU_1368_130
ttattgaaatttaaatttggctgcattgttgctggctcttaggagcatgtgcacatgcaccatttttttttttttttaaaaaaaccacctgtgcacacactgtagatctggattgaactttttcgaggattattaactcggttttgagaactgctttggctgttcttgtgttctccaggtctatgttttaaacaatatactccaacaagtaatagaatgtcaaattatgggccttttgtgcctttaaaatctaatacaactttcaacaacggatctcttggctctc
>OTU_1371_4
ttacagagttgtttaaaactcccaaacccatgtgaacttaccttttgttgcctcggcagggcctattgaggtcatcgcgacctcggtggtggcctgccggtggactaccaaactcttgttattatcgtggaactctgagttgcataaacttaataagttaaaactttcaacaacggatctcttggttctg
>OTU_1375_50
ttaccgagtttacaaactcccaaacccctgtgaacttatacctttacgttgcttcggcgggactcgccccggctgccctcgcggcggaccggacccaggcgcccgccggaggacccaaactcttctgtcttttatagcggcatgtctgagtggatttttatacaaatgaatcaaaactttcaacaacggatctcttggttctg
>OTU_1376_1
ttaccgagttagggtagtattcactgcccgacctcccaaccctgtgtctaccacactttatcgttgcttcggcggaccggttgaccaactggtcgtgaccgccgggggttggctctgtcccccctggagagcgtccgtcgatggcccaaccacaaaatctcttgtaccgaaacgtgtcgtctgaattattgagaaatcaaaaaaacaaaactttcaacaacggatctcttggttctg
>OTU_1377_47
ttaccgagtgagggccctctgggtccaacctcccacccgtgtttatcgtaccttgttgcttcggcgggcccgcctcacggccgccggggggcacccgcccccgggcccgcgcccgccgaagacaccattgaacgctgtctgaagattgcagtctgagcatcttagctaaatcagttaaaactttcaacaacggatctcttggttccg
>OTU_1392_3
ttaccgagttagggtagtcactcactgcccgacctcccaaccctgtgtctaccacactttgtcgttgcttcggcggaccggttgaccaactggtcatgaccgccgggggctggctctgcccccctggagagcgtccgccgatggcccaaccacaaaactcttgtaccgaaacgtgtcgtctgaatcatgtttgagaaatcaaaaacaaaactttcaacaacggatctcttggctctc
>OTU_1393_6
ttacagagttcatgccctcacgggtagacctcccacccttgtgtatctataccatgttgctttggcaggctgctggccccctcgggggacagccccagcgccctcgggcctgagagtcgcctgccggaggaaaaacaaactctgaactgttagtgtcgtctgagtactatctaatagttaaaactttcaacaacggatctcttggttctg
>OTU_1395_2
ttaccgagttcatgccctcacgggtagatctcccaccctatgttatcattacctttgttgctttggcgggccgccaggcttcggctaggctaccggctccggctggtaagcgcccgccagaggaccccaaactctgaatgttagtgtcgtctgagtactattaaatagttaaaactttcaacaacggatctcttggttctg
>OTU_1397_98
ttactgagaacatgccctttagggtatatctcccaccctttacaataccattgttgctttggtgggcccgtcatatgaccaccggctttggctggtttgtgcctgccagaggaccccaaaactctttactatgtcgtctgagtactatgtaatagttaaaaaactttcaacaacggatctcttggttctg
>OTU_1401_3
ttaccgagttagggtagtattcactgcccgacctcccaaccctgtgtctaccacactttatcgttgcttcggcggaccggttgaccaactggtcatgaccgccgggggctggctctgtcccccctggagagcgtccgccgatggcccaaccacaaaactctttgtaccgaaacgtgtcgtctgaattattgagaaatcaaaaacaaaactttcaacaacggatctcttggctctc
>OTU_1403_4
ttagagaaatcttagacggctgtgcgccctcgggccgcacgtcgtttcaatttccacacaactgtgcaccttcgacgggagtggggaaacctgccccccgtcgctttcattaactgaatcagtcctgaatgtaaacccctttataaaaaatacaactttcagcaacggatctcttggctctc
>OTU_1404_3
ttacagagttgcaaaactccctaaacacttgtgaacctaccagtatcgttgcttcggtgggcggccccagggcggggccgtagcctttacaggcgcccgccggaggttacaaactctatatttttaattgtctctctgagtaaacttataaataagtcaaaactttcaacaacggatctcttggttctg
>OTU_1407_2
ttactgaattgtcaaacgggttgttgctggccctcatagggggcatgtgcacgctctgtttacacatccactcacacctgtgcaccctctgtagttctatggtttgggggaccctgtcttccttctgtggttctacgtctttacacacacacactgtaataaagttttatggaatgtacatcgcgtctaacgcaatacaatacaactttcagcaacggatctcttggctctc
>OTU_1408_34
ttattgaaataaacctgatgggttgttgctggttctctagggagcatgtgcacgccttgtcatctttatacctccacctgtgcactttttgtagaccttttaggtctatgttgcttcatttaccccaatgtatgttaatagaatgttgtgcctatgtaataaatacaactttcagcaacggatatctcggctctc
>OTU_1409_12
ttaccgagtttcgtgcccgtacgggtagatctcccaccctatgttattattaccttgttgctttggcgggccgccaggctccggtcaggctatcggcttcggctggtaagcgcccgccagaggacccaatattctgattatcagtgtcgtctgagtactatgataatagttaaaactttcaacaacggatctcttggttctg
>OTU_1410_2
ttaacgagttagggtcctctgggcccgacctcccaaccctttgtttaccgaacctctgttgcttcggcggacccgtctcacgaccgccggaggatccgcctctcggggcgtcctctggccagtgtccgccgagagccaaccctcaaactcctgaataaatcatgttatgtgtctaagtcttatgattcaaaatcaataaagcaaaactttcaacaacggatctcttggttctg
>OTU_1415_29
ttaacgagttagggtctctcacggcccgacctcccaaccctttgtttatcgaacctctgttgcttcggcggatccgtccctcgggaccgccggaggatcgtcgtaaggcgtcctctggcccgtatccgtcgatagccaaccacttaaactcagaataaatcgtgttataattgtctaagtctatgattaaattaaagcaaaactttcaacaacggatctcttggttctg
>OTU_1419_21
ttaccgagtttacaactcccaaacccaatgtgaacgttaccaaactgttgcctcggcggggtcacgccccgggtgcgtcgcagccccggaaccaggcgcccgccggagggaccaaccaaactcttttctgtagtcccctcgcggacgttatttcttacagctctgagcaaaaattcaaaatgaatcaaaactttcaacaacggatctcttggttctg
>OTU_1422_112
ttactgagaacatgccctctagggtatatctcccacccattgtttacaataccaaatgttgctttggcaggcccgtctcatgaccaccggctttggctggtctgtgcctgccagtggaccccaaaactccttttattatgtcgtctgagtactatgataatagttaaaactttcaacaacggatctcttggttctg
>OTU_1426_28
ttaccaaatcataagtcggctgaccgtgctggcggaaacgcaagtgcacgtcggtcgcaaaccaatccacacacctgtgaacgtatggcctttcgagttttctcgggggcaaaccttttttacccactctgattgtaaaggaatgtctatgtgcctaaagcgcagaaagcaaacaactttcaacaacggatctcttggctctc
>OTU_1428_4
ttactgagtgagggcccctcggggtccaacctcccacccgtgtatatcgtacctagttgcttcggcgggcccgcctcacggccgccggggggcatctgcccccgggcccgcgcccgccgaagacccccttgaacgctgtctccaaaatgcagtctgagcgaaaagctaaattagttaaaactttcaacaacggatctcttggttccg
>OTU_1430_1
ttaacgagttagggtctctctaggcccgacctcccaaccctttgtttaccttgctgcatgttgctttggcggacccgtctcacgaccgccctgggaccgccgagaggcgttccctaggcccgtgtccgccgatagccaacctaaactcttggatgaatcgtgttttatgtctgagtcttatgattaaatcaaaagcaaaaactttcaacaacggatctcttggttctg
>OTU_1434_1
ttaatgattacgaacggttgccttcggtgctggctcttgcaagtgcacgttggtggctttcatccaatacccctgtgaacctttggcctcttgctagcttcggctggcagaggatttttacacactcgaatgtaatgaaaactattgtcgtgcgcaagcactaatgtacaactttcaacaacggatctcttggctctc
>OTU_1437_14
ttacagagttcatgccctccgggtagatctcccacccattgctataactactctcgttgctttggcgggccgctgggccctgcccggccgccggccccggctggcgcgtgcccgccagagacctcacagactctgaatgttagtgtcgtccgagtaactatacaatcgttaaaactttcaacaacggatctcttggttctg
>OTU_1441_117
ttcacttgataaaaaaggcttctgaagcctttttctctctttcttaaccaagtggggttcttgaattaccactgtgatgctgagatatgggtcttgaagccctttgtgattgctttcaggcatctgatcttatccagcaaaaggttcatattgtaaaacccgactgtgagtttgggagtattggaccatatttataatatcactcagttgttggtcaagttgaaaagcaatccactcttggaggggagagaaggatatggttgagggtttgtagcccaaggttgtattctatcttgtctctctctctcttgagtctagagaactcgagataattgtccatattgtggaattatcataacatgagctcatttacaactttcagcaatggatctcttggttctc
>OTU_1442_15
ttaaagagtaccggagctctcgggttcctactcccaccctatgttgactttaaatgttgctttggcggaccggcgatctcgccactggcctaggctggacagcgtccgccagaggatttttatattcctttgactgaattctgagtctttgaaaattgaatcaaaactttcaacaacggatctcttggttctg
>OTU_1443_13
ttactgagttgttgacacgagctgttgctggtcctcaaagcaaggggggcatgtgcacgctctgttcacacatccactcacacctgtgcaccccccgtagttctatggtctggagggcttgccgtcttcttcccgtagttctacgtctttacacatacaccgtagtaaagtcttatggaatgtgcgccgcgtttaacgcaataaaatacaactttcagcaacggatctcttggttctg
>OTU_1446_13
ttaccgagtttacaactcccaaacccctgtgaacataccatttgttgcctcggcggtgcctgcttcggcagcccgccagaggaccccaaaccctgattacatttaagaagtcttctgagtaaccgattaaataaatcaaaactttcaacaacggatctcttggttctg
>OTU_1451_95
ttatcgtacaaccgaggtgcaaaggctgtcgctgaccctcaaaggtcgtgcacgcccgagcgctctcgcacaatccatctcacctttgtgcatcaccgcgtgggtcccctttgcgggagggcttgcgttttcacataaaacttgatacagtgtagaatgtttttcttttgcggtcacacgcaatcaatacaactttcaacaacggatctcttggttctg
>OTU_1452_1
ttacagagactctgccctttgggtagacctcccaccctgtgtcgttatacctctgttgctttggcgggccgtggggccccggccctgcccctggctccggctagggcgcgcccgccagaggatctcaaaacctgaacgttagtgtcgtctgagtactatataatagttaaaactttcaacaacggatctcttggttctg
>OTU_1454_84
ttaccgagtaccggagcccctcgggttcctactcccaccctatgttgactttgaatgttgctttggcggaccggtggtctcaccgctggcctaggctggagagcgtccgccagaggattttaaactctgtttaacggtgaattctgagtcttgataaattgaatcaaaactttcaacaacggatctcttggttctg
>OTU_1457_3
ttagtgaatcttcaaagtcggctcgtcggattgtgctggtgggagaccacatgtgcacgtctgcgagtcgcaaacccacacacctgtgcatctatgactctgagtgccgctttgcatggccccttgatttgggcctggcgctcgagtactttcacacactctggaatgtaacggaatgtcttgttgtgcataatgtacaaacagaaacaactttcaacaacggatctcttggctctc
>OTU_1460_47
ttaccgagcgagcgccctcgggcgcggcctccaacccttttgtgaatccaacctcagttgcctcggggtgacccggcctcgcccggcccccgacggacgattcgacccaactctgcatctttgcgtcggatatgaaaagtcaattgatttaaaactttcaacaacggatctcttggttctg
>OTU_1462_5
ttaccgagtttacaactcccaaacccctgtgaacatacctattgttgcctcggcggatcagcccgctcccggtaaaacgggacggcccgccagaggacccctaaactctgtttctatatgtaacttctgagtaaaaccataaataaatcaaaactttcaacaacggatctcttggttctg
>OTU_1465_22
ttaccgagttagggtcttctaagggcccgacctccttacctttgtctaccttacctcacgttgcttcggcgggcccgtcctcttttggaccgccggagggttcacccccctctggcccgttgcccgccgacagccccaaccaaaaaactcttgcaataactgtgaaattgtctgaattttgcttctaaaataaccaaaaactttcaacaacggatctcttggttctg
>OTU_1466_1
ttaccgagttagggttcgtcccacgagcccgacctcccaaccctttgtctattataccttgtcgttgcttcggcggaccggtcacgaccaactggtcgaggccgccgggggctcgtccctggagagcgtccgccgatggcccaaccacaaactcttgtaccaaacgatgtcgtctgaattacttgattaaaatcaaaaacaaaactttcaacaacggatctcttggttctg
>OTU_1467_9
ttaccgagtttacaactcccaaacccctgtgaacataccacttgttgcctcggcggatcagcccgctcccggtaaaacgggacggcccgccagaggacccctaaactctgtttctatatgtaacttctgagtaaatgattaaataaatcaaaactttcaacaacggatctcttggttctg
>OTU_1472_2
ttaccgagtttacaactcccaaacccctgtgaacatacctttatgttgcctcggcggatcagcccgcgccccgtaaaacgggacggcccgccgcaggaaccacaaaactctgattttagtgtaacttctgagtctaaaaaacaaataaatcaaaactttcaacaacggatctcttggttctg
>OTU_1473_1
ttaccgagtgagggccctctgggtccaacctcccacccatgtttatcgtaccttgttgcttcggcgggcccgcctcacggccgccggggggcttctgccctctggcccgcgcccgccgaagacaccattgaacactgtctgaagattgcagtctgagcaattagctaaataagttaaaactttcaacaacggatctcttggttccg
>OTU_1476_10
ttaccgagtttacaactcccaaacccctgtgaacataccatttgttgcctcggcggtgcctgcttcggcagcccgccagaggacccaaacccttgattttatacagtatcttctgagtaaaaccataaataaatcaaaactttcaacaacggatctcttggttctg
>OTU_1479_30
ttaacgagttagggtcttccaggcccgacctcccaaccctatgtttattgaacctctgttgcttcggcgggcccgtctcacggccgccggaggagtcgccgcgaggcaccctctggccagcgtccgccgatagccaaccccaaaactctgaatgaatcgtgtcatgatgtctaagtctatgattaaattaaagcaaaactttcaacaacggatctcttggttctg
>OTU_1482_76
ttaaaagtaaatgcgtattcctcgcgaatatacatatacaaacccattccgagtacattacctgttgcttccgtggagcagtgattcgtcacctctgaagatggctttggccacctgaggggagtctccacgggaggtatacattaaactcttgcattaccatgtcttctgtctgaatctgtttataacaaatgttaaaactttcaacaacggatctcttggttctc
>OTU_1483_8
ttactgagaatgctctccggagccctcaaatcttgcgtacccatctcgtacttgcgggttgcccccgggcgcccgcgaggattcaaactgtttgaccatcgtcagattcttacaaaagaaaacaactttcaacaatggatctcttggctccg
>OTU_1486_10
ttactgaattgttaacaagagttgttgctggtccctatacgggggcatgtgcacgctctgtttacacatccattcacacctgtgcactctctgtagttctgtggtttgggggctctgtcctcctaccgtggtcctgcatatttacacatacacactgtgataaagtctcatggaatgtatgccgcgtttaacgcaatgaaatacaactttcagcaacggatctcttggctctc
>OTU_1487_35
ttacagagttcatgcccttacgggtagatctcccacccttgaatactatacctttgttgctttggcgggccgcttcggctactggctctggctagtgagtgttcgccaaaggatcccaaaactctgaatatttgtgtcgtctgagtactatgtaatagttaaaactttcaacaacggatctcttggttctg
>OTU_1490_4
ttagagaaatcttagacggctgtgcgccctcgggccgcacgtcgtttcaatttccacacaccgtgcaccttcgacgggagtggggaaacctgccccccgtcgctttcattaaccgaatcagtcctgaatgtaaacccctttataaaaaatacaactttcagcaacggatctcttggctctc
>OTU_1492_1
ttaatgattgcgaatcgtcgccttcagtgctggccggcttcggcaagtgcacgtcggtgactttcatccaataccctgtgcacctttggcctcttgctagcttcggcctgcagaggattttacacacactcgaatgtaatgaaatttattgtcgtgcgcaagcactaatgtacaactttcaacaacggatctcttggctctc
>OTU_1497_17
ttaccgagtgagcgcctccgggcgcgacctccaacccattgtgattatacctctgttgcctcggggtgacccggccccgcccggcccccgaaggacgattagacccaactctgcatctttgcgtcggattgaaaagattaattgatgaaaactttcaacaacggatctcttggttctg
>OTU_1498_1
ttacagagttcatgcccgaaagggtagacctcccacccttgtgtattattactttgttgctttggcgagctgccttcgggccttgtatgctcgccagagaaaaccaaaactctttttattaatgtcgtctgagtactatataatagttaaaactttcaacaacggatctcttggttctg
>OTU_1502_37
ttactgagaacatgcccctaggggtatatctcccaccctctgtttacaatacctctgttgctttggcaggcccgtcttatgaccaccggctccggctggtctgtgcctgccagagaacaacccaacactcttttattatgccgtctgagtattatataatagttaaaactttcaacaacggatctcttggttctg
>OTU_1504_14
ttacagagttctcgccctcgcgggtagacctcccacccctgtgattgctgccgtcgttgctttggcgggccgccgtcgtggccccgagtgcccgccagaggccccatcaaccctgatcgtgcgtgtcgtctgagtgctattaaatagtcaaaacttttaacaacggatctcttggttctg
>OTU_1505_2
ttaccgaattccaaggaggggaaagcaaggggggatatttccagtagtatttcggtagtgctgttgctggccttcgggcatgtgcacgtctccggagttgtaatggtgttattattccccttgttccctctaatacctcttgaacacctgtgtgcacctgttgtaggtctcgtcactgggacctatgtattccacattaaactcgcatgtatacagaacgctgtctagtcgtgtcgtccggccccgagggtcggcgaccgaaaaatgataatataatataactttcagcaacggatctcttggctctc
>OTU_1506_3
ttacagagttcatgcccttcggggtagatctcccacccttgtgtatcattatagaatgttgctttggcgggccgcgtgcctagcacgcctcgattcgcgtcgagcgtgcccgccagaggacccctaaactctgaatattaatgtcgtctgagtactatataatagttaaaactttcaacaacggatctcttggttctg
>OTU_1511_1
ttaccgaattgtcaacacgagttgttgctggtcctcaaaacgggggcatgtgcacgctctgtttacacatccactcacacctgtgcaccctctgtagttctgtggcatgggggactctgtcctcctgttatggtcctacgtctttacacacactctgtaacaaagtctcatggaatgcatgacgcgtttaacgcaatacaatacaactttcagcaacggatctcttggttctg
>OTU_1516_2
ttaccgaattgtcaacacgagttgttgctggtcctcaaatggggacatgtgcacgctctgtttacacatccactcacacctgtgcaccctccatagttctgcagcctgggggggctctgttcccctgccgtggttctatgtatttacacacacacacactgtagtaaagtctcatggaatgcataccgcgtttaacgcaatataatacaactttcagcaacggatctcttggctctc
>OTU_1520_8
ttaccgaatggcctctgggccttcgacctcacaaacctgtggaagcgagatgtgcttcggcgtcgtctgtggcgccgttttcatgcttaagtctgtccatgtgtctgagcgtgacataaacaaaaaaaactttcaacaaaggatctcttggctctc
>OTU_1524_1
ttaccgagttcatgccctgacgggtagatctcccatcctctgttatcattacttttgttgctttggcgggccgtcaggccccggtcaggctaccggctccagctggtaagcgcccgccagaggaccccaaaccctgaatattagtgtcgtctgagtcctatgtaatcgttaaaactttcaacaacggatctcttggttctg
>OTU_1525_3
ttactgaattgtcaacacgagttgttgctggccctcatagggggcatgtgcacgctctgtttacacatccactcacacctgtgcaccctctgtagttctatggtttgggggaccctgtcttccttctgtggttctacgtctttacacacacactgtaataaagttttatggaatgtacatcgcgtctaacgcaatacaatacaactttcagcaacggatctcttggctctc
>OTU_1527_16
ttcataataagtgttttatggcactttttaaatccatatccaccttgtgtgcaatgtcagtcgatcttcttcatggagatcgaccaaacatcaaccttatcttttaactctttgtctgaaaaatattatgaataaacaattcaaaatacaactttcaacaacggatctcttggttctg
>OTU_1533_92
ttaccgaattgtcaaacgggttgttgctggtcctcaaacggggacatgtgcacgctctgtttacacatccactcacacctgtgcaccctccgtagttctatggtctgggggaccccgtcttcctgccgtggttctgcgtctttacacacacgctgtaacaaagtcttatggaatgtatgccgcgtctaacgcaatacaatacaactttcagcaacggatctcttggctctc
>OTU_1534_1
ttaccgagttagggtctaaataggcccgacctccaaccctttgtttactataccatgttgctttggcgggcccgcctttcggggctgccgggggttcatgcccctggtcagtgcccgccagtagccttattaaattcttccataattatgttgtctgagtataaataaaatcgttaaaactttcaacaacggatctcttggttctg
>OTU_1535_12
ttaccgagtgcgggccctctgggtccaacctcccacccgtgtttattgtaccttgttgcttcggcgggcccgccattctggccgctaaggggcacctgcccccacgcccgcgcccgccgaagacacctcgaactctgtctgaagattgaagtctgagtgaaaatacaaatagttaaaactttcaacaacggatctcttggttccg
>OTU_1536_5
ttaacgagttagggtcttccaggcccgacctcccaaccctatgtttattgaacctctgttgcttcggcggacccgtctcacgaccgccggaggaccgccgcgaggcgtcctctggccagcgtccgccggtggccaaccacaaaactctgaatgaatcgtgtcattatgtctcagtctatgattaaattagaagcaaaactttcaacaacggatctcttggttctg
>OTU_1537_2
ttattgaaataaacctgagcaggctgttttgctggtccttggacatgtgcacgcttgttcgtctttattcttccaactgtgcacatattgtaggcctggagctttcttgttcgtttgaatagattgaggactgccagtgtggctttgccttgcgtttccaggtctatgttttgtaatacattttcacaatggtttcagaatgtcgagtactggggataaacaaatatacaactttcagcaacggatctcttggctctc
>OTU_1538_34
ttattgaataaactttgagcaggctgtttgctggcccttgtcatgagggtatgtgcacgcttgtcctttgttatttccccaacttgtgcacactttgtagaccctggagatcttatgaattttcattcgagttgggattgctgtgctttctccacttgagaaaagtcagctttgccttgccatcttcaggtctatgtcattttttcacaacctctaaaaacatgttcagaatgttgaatcaggtcttttttgtacctatagaaatgaaatatacaactttcagcaacggatctcttggctctc
>OTU_1540_1
ttcataatgagtgtttatggcacttttaaaaatccatacccaccttgtgtgcatcgttagtcatgttccttgttctctcttgagagagcgaggaggcagacttcaaattaatctatctttaaccagtttgtctgaagcaatgtatcatgagttaacataattctaaaatacaactttcaacaacggatctcttggctctc
>OTU_1542_4
ttattgattgcgaatcgttgtctccagtgctggccaggtcactctggcaagtgcacgtcgacagctttcatccaataccctgtgaacctttggcctcttgctagcttcggcgagcagaggattttacacccactcgaatgtaatgaaattattgttgtgcacaagcactactatacaactttcaacaacggatatctcggctctc
>OTU_1545_53
ttattgaaataaacctgatgggttgttgctggctctctagggagcatgtgcacaccttgtcatctttatatttccacctgtgcactttttgtagacctttcaggtctatgttgctacatttaccccaatgtatgttaatagaatgttgtgactatataatatatacaactttcagcaacggatctcttggctctc
>OTU_1547_5
ttactgaaccgctgacacgagctgttgctggtctccaaatgggggcacgtgcacgctctgtttgtgcatccactcacacctgtgcacccttctgtagttctgtggcctgggggactcgtcctcctgctgtggttctgcatctttacactcacgctgtgataaagtctaatggaatgcatgccgcgtttaacgcaatacaatacaactttcagcaacggatctcttggctctc
>OTU_1552_25
ttacagagttcatgcccttcggggtagatctcccacccttgtgtatcattatagaatgttgctttggcgggccgcgtgcctagcacgcctcgattcgcgtcgagcgtgcccgccagaggacccctaaactctgaatgttagtgtcgtctgagtactatgcaatagttaaaactttcaacaacggatctcttggttctg
>OTU_1556_12
ttaccgagtgagggccctctgggtccaacctcccacccgtgtttatcgtaccttgttgcttcggcgggcccgcctcacggccgccggggggcttctgccctctggcccgcgcccgccgaagacaccattgaacgctgtatgaagattgcagtctgagcttattagctaaattagttaaaactttcaacaacggatctcttggttccg
>OTU_1558_1
ttaaagattcggccctcgggccccaaacctcttcagctgtgcgcttcggctgcacgctgtcgaaactctcacaccctgtgcacatttcggttgcgatcctcacgggtcgcttccgcttttcacatacaactaccagtccagaatgtaacaaaccataaaagtaacaactttcaacaacggatctcttggctctc
>OTU_1560_24
ttaccgagtttacaactcccaaacccatgtgaacatacctactgttgcttcggcgggattgccccgggcgcctcgtgcgccccggatcaggcgcccgcctaggaaacttaactcttgttttattttggaatcttctgagtagtttttacaaataaataaaaactttcaacaacggatctcttggttctg
>OTU_1564_8
ttaaagagtaccggagctctcgggttcctactcccaccctatgttgactttaaatgttgctttggcggaccggcagtactgccactggcctaggctagatagcgtccgccagaggatttttaaattcctttgaaggtgaattctgagtctttgaaaattgaatcaaaactttcaacaacggatctcttggttctg
>OTU_1566_11
ttaccgaattgtcaacaagagttgttgctggtcctcaaatgggggcatgtgcacgctctgttcacacatccactcacaccctgtgcaccctctgtagttctatggtcagggggcctgtcctcctgctgtggttctgcatctttacacacacactgtaacaaagtctcatggaatgcatgccgcgtttaacgcaatataatacaactttcagcaacggatctcttggctctc
>OTU_1574_2
ttaccgaattgtcaaacacgggttgttgctggccctcatagggggcatgtgcacgctctgtttacacatccactcacacctgtgcaccctctgtagttctatggtttgggggaccctgtcttccttctgtggttctacgtctttacacacacactgtaataaagttttatggaatgtacatcgcgtctaacgcaatacaatacaactttcagcaacggatctcttggctctc
>OTU_1577_99
ttaacgaatggtcatccttgatcttcttaccctcaaaccactgtggacacacaagatgtgcttcggcgtcgagaggcgccgccttcatgcttcatcaagtctttcttgtctgtaaaaaacaaatgtcaaaactttcaacaaaggatctcttggttctg
>OTU_1584_8
ttaacgagttagggtcttctaggcccgacctcccaaccctatgtttattgaacctctgttgcttcggcggatccgtctcacggccgccggaggaccgctgaaaggcgtcctctggccagcatccgccgatagccaaccacttaaactctgaataaatcgtgtcatatgtctaagtctatgattaaattaaagcaaaactttcaacaacggatctcttggttctg
>OTU_1587_101
ttactgaactgtcaacacgagttgttgctggtcctcaaatgggggcatgtgcacgctctgtttacatacccactcacacctgtgcaccctctgtagttctgtggtgtgggggactctgtcctcccgctgtggttctacgtctttacacacacacacagtttcatagaatgtatgtcgcgtttaacgcaatacaatacaactttcagcaacggatctcttggctctc
>OTU_1588_5
ttcataataagtgttttatggcactttttaaatccatatccaccttgtgtgcaatgtcagtcgatcttcttcatggagatcgaccaaacatcaacctttattttttaactctttgtctgaaaaatattatgaataaacaattcaaaatacaactttcaacaacggatctcttggttctg
>OTU_1601_16
ttactgaattgttaacaagagttgttgctggccctcatgcgggggcatgtgcacgctctgtttacacatccattcacacctgtgcactctctgtagttctgtggtttgggggctctgtcctcctaccgtggtcctgcatatttacacatacacactgtgataaagtctcatggaatgtatgccgcgtttaacgcaatacaatataactttcagcaacggatctcttggctctc
>OTU_1610_1
ttatcgtacaatggaggtgctggggttgtcgctgacctttgaaagggtcgtgcacgcctcggtgctttcgctttcacacacaatccatctcaccccttttgtgcatcaccgcgtgggggtcccttttagctagttctgaagggggctttcgcgtttttacaaacacacccttttaatgcaatatgtagaatgtcttactttttgcgatcacacgcaatcaatacaactttcaacaacggatctctaggctctt
>OTU_1621_5
ttacagagttcatgcccttcggggtagatctcccacccttgtatatcattatagaatgttgctttggcgggccgcgtgcctagcacgcctcgattcgcgtcgagcgtgcccgccagaggacccctaaactctgaatgttagtgtcgtctgagtactattcaatagttaaaactttcaacaacggatctcttggttctg
>OTU_1623_3
ttaaagagtaccggagctctcgggttcctactcccaccctatgttgactttaaatgttgctttggcggaccggcaatcttgccactggcctagggctagacagcgtccgccagaggatttttaaattcctttaaaaggtgaattctgagtctttgaaaattgaatcaaaactttcaacaacggatctcttggttctg
>OTU_1627_5
ttattgaaataaacctgatgagttgctgctggctctctagagagcatgtgcacgcttgtcgtctttatatctccacctgtgcacctattgtagtcctggatgactctctgaatggctatcattcaggtatgaggattgactttctgcctctccttacatttccaggcctatgttctttcatataacctcaatgtatgttatggaatgtaataattatggccttctgtgccttataaacctatacaactttcagcaacggatatctaggctctc
>OTU_1633_16
ttaccgagttagggtcttctaagggcccgacctccttacctttgtctaccttacctcacgttgcttcggcgggcccgtcctcttttggaccgccggagggttgaacccctctggcccgttgcccgccgacagcccccaaccaaaaaactcttgcaataactgtggaattgtctgaatcttgcttctaaaataaccaaaaactttcaacaacggatctcttggctctc
>OTU_1637_1
ttaccgaaccgtcgacacgagttgttgctggccctcgaaaggggtacgtgcacgctctgtctacacatccactcacacctgtgcaccctctgtagttctatggccttggggaccccgtccccttgccgtggtcctacgtctttacacacacacaccgtaataaagtctcgtggaatgtatgtcgcgtttaacgcaataaaatacaactttcagcaacggatctcttggctctc
>OTU_1638_9
ttaccgagtttacaactcccaaacccctgtgaacatacctattgttgcctcggcggtgcctgttccgacagcccgccagaggaccccaaaccctgattacatttaagaagtcttctgagtaaaaccataaataaatcaaaactttcaacaacggatctcttggttctg
>OTU_1639_65
ttatccatctcaaccaggagtggtggcggccaggcattgcgtcctcggttcagccccgctttcctgcatcctttttttacgagcacctttcgttctccttcggcggggcaacctgccgctggaacttaacaaaacctttttttgcatctagcattacctgttctgatacaaacaatcgttacaactttcaacaatggatctcttggctctg
>OTU_1651_65
ttaacgagcgcggccccttcggggtgccaacctcccacccgtgtctaccgtaccacgttgcttcggcgggcccgcccccaacaggggggccgtcggggggcatgagcccccgggcgtgcgcccgccggagacccccacacgaacgctgtctgaagactgccgtctgagtggattgatcaaatcagttaaaactttcaacaacggatctcttggttccg
>OTU_1655_17
ttattgtataaccgaggtgctagggctgtcgctgaccctttgaagggtcgtgcacgcccaagtgctctctcacatccatctcacccctttgtgcatcaccgcgtgggctacctttttggctttattcaaaaaggttggttcgcgtttttacacacacacctttatgtatagaatgtcttgatttttgcggtcatacgcaatcaatacaactttcaacaacggatctcttggctctc
>OTU_1660_63
ttaaagagtaccggagctctcgggttcctactcccaccctatgatgactttaaatgttgctttggcggaccggcaatattgccaccggcctaggctggatagcgtccgccagaggatttttaaattcctttgaatgtgaattctgagtctttgaaaattgaatcaaaactttcaacaacggatctcttggctctc
>OTU_1661_3
ttactagagcaaaggataggcagcgcgccccacagaagcttgcttcgtggcgggctaccctacttcggtagggtttagagtcgtcgaacctctcggagaagttcggtcctgaactccacccttgaataaattacctttgttgctttggcgggccgcctcgtgccagcggcttcggctgttgagtgcccgccagaggaccacaactcttgttttttgtgatgtctgagtactatataaatagttaaaactttcaacaacggatctcttggttctg
>OTU_1663_5
ttactgagtgagggccctctgggtccaacctcccacccgtgtttattgtaccttgttgcttcggtgagcccgcctcacggccgccggggggcttctgcccccgggtccgcgctcaccgaagacaccattgaactctgtctgaagattgcagtctgagcataaactaaataagttaaaactttcaacaacggatctcttggttccg
>OTU_1664_88
ttacagagtttagggtcccttgtgggcccaaacctccaaccccccttggtgtttactaccctgttgctttggcaggccccaatgggtttacctgccggagccttagtgtaacaatctgtttaatgaattagttagtctgatccttctggaaaacatagaattgttaaaactttcaacaacggatctcttggttccc
>OTU_1665_15
ttaccgagtttacaactcccaaacccctgtgaacatacctattgttgcttcggcggacacgccccggcgcccccgtgcccggaaccaggcgcccgccggaggaccacaaaccatgtcttactatagtggtattatctgagtggcacaaacaaataaatcaaaactttcaacaacggatctcttggttctg
>OTU_1672_14
ttactgaattgttaacaagagttgttgctggtccctatacgggggcatgtgcacgctctgtttacacatccactcacacctgtgcaccctctgtagttctatggcctggggggctctgtcctcctgctgtggctctacgtctttacacacacactgtaacaaagtctcatggaatgtatgtcgcgtttaacgcaatgaaatacaactttcagcaacggatctcttggttctg
>OTU_1674_69
ttaacaaatccaatacttgtgggggactaccaagtctttctgcaaatattaatccgccggctcttagcagctggctgtgataatatatatgcgtgttatgttgacagtgtgtgtgattaatttaatttattgacaacttttaacaatggatctctaggctctt
>OTU_1677_81
ttattgaattttgaaagaggttgtagctgatcaaaaaaagatatgtgcacacctcatttccaaattttacacccatgtgcactctttgtaggccatttggcctatgtcttcataatacactcttgaaaggtttcagaatgtgaaatgtatatcttaaattaatacaactttcaacaacggatctcttggctctc
>OTU_1683_5
ttattgtacaacggaggtgcgagggctgtcgctgaccttcaaaggtcgtgcacgcccgagccctctcacaatccatctcaccctttgtgcatcaccgcgtgggtccccctttagcggggagggctcacgtttttacataaaactcgatgcagtgtagaatgtttatttttgcggtcacacgcaatcaatacaactttcaacaacggatatctcggctctc
>OTU_1687_36
ttattgaaataaacctgatgagttgctgctggctctctagagagcatgtgcacacttgtcgtctttatatctccacctgtgcacctattgtagacctggatgactctctgaatggctatcattcaggtatgaggattgactttctgcctctccttacatttccaggcctatgttctttcatataacctcaatgtatgttatggaatgtaataattatggccttctgtgccttataaacctatacaactttcaacaacggatctcttggttctg
>OTU_1689_24
ttaacgagttagggtcttctcggcccgacctcccaaccctttgtttactgaacctttgttgcttcggcggacccgtctcacgaccgccgggggaccgtaagacgtcctctggcccgtgtccgccggtggccaaaccgaacaaattctgattaaaatgtgtcaatgtctgagtagaattcaattaaaacaaaactttcaacaacggatctcttggttctg
>OTU_1694_2
ttacagaaagtaaacgcggatcaaaccgcgaacttctaaacctttgacgattgacttatgttgcctcggcgggttctcccgccagaggatatatcaaaactcctgttttaacggtgctgtctgagctacaagcaacgaatcaaaactttcaacaacggatatctaggctctc
>OTU_1706_24
ttactgagtgagggcccctggcccgacctccaaccctgtgtcgattccaacctcttgcctcgggggtgacccggtcctccgcggaccggggcccccggtggatcatcaaactctgcgttagagcgtccgagttaataatcaaatggatgaaaactttcaacaacggatctcttggttctg
>OTU_1709_57
ttatcaaaaataattcattactttttgaaatatttttcccaatttcccttgtgtatctgctccatgttgcttccactagacaggcatatatattattaaaaattcaattttcaataatatttttgcccttttaaccctgtcgagggcccaaagaatagtcggtggatagccttgatccaagaaaacacttttagatttagtctgataaatatagtattttttctgaataaaaaaaactttcaacaacggatctctaggctctt
>OTU_1714_10
ttacagagttcatgccctcacgggtagatctcccacccttgaatactatacctttgttgctttggcgggccgccttggctactggctccggctggtaagtgcccgccaaaggaccccaaaactctgaatattagtgtcgtctgagtactatataatagttaaaactttcaacaacggatctcttggttctg
>OTU_1715_1
ttaccgagttgtggaaacacaatctccctttcttgttgtttgaccggttgccttgccttcgggcaggattattctgaatcagtgtcgtctgagtacttttataatactataaaactttcaacaacggatctcttggttctg
>OTU_1722_5
ttaccgagtttacaactcccaaacccaatgtgaaccataccaaactgttgcctcggcggggtcacgccccgggtgcgtaaaagccccggaaccaggcgcccgccggaggaaccaaccaaactctttctgtagtcccctcgcggacgtatttcttacagctctgagcaaaaattcaaaatgaatcaaaactttcaacaacggatctcttggttctg
>OTU_1741_14
ttaccgaatcgtcaaacacgggttgttgctggctccccaaacggggggcatgtgcacgctctgtttacgcatccactcacacctgtgcaccctctgtagttctatggtttgggagacatcgtcttccttctgtggctctacgtctttacacacacaccgtagcgatgtctcatggaatgtatgtagcgtttaacgcaatacaatacaactttcagcaacggatctcttggctctc
>OTU_1747_17
ttaccaaaagataatctttcaacactgaaagatcttacctttgtgctggctttgacagttttgtacttttggggctttaaaatggttcagtagttaaagaagggagcaatccctttttttcatgctactgggtcggccccaaataatcatatcatccttaaaaatttttctgattaattaacacatgattttaataatctgtttaaaacaactttcaacaacggatctcttggttctc
>OTU_1748_13
ttaccaaaagataatctttcaacactgaaagatcttacctttgtgctggctttgacagttttgtacttttggggctttaaaatggttcagtagttaaaggagggagcaatccctttttcattctactgggtcggccccaaataatcatatcatccttaaaaatttttctgattaattaacacatgattttaataatctgtttaaaacaactttcaacaacggatctcttggttctc
>OTU_1750_2
ttattgaatgaacttggtgtggttgttgctggcccttttggggcatgtgcacacccaccatcctcatctctccacctgtgcacctcttgtagacttgggataagactttccgaggcaactcggtctggaggaatgcttgctacccaagctttctttgtatttatttccaggtctatgtttttcatatacccaatagtatgtaacagaatgtatgatttaggcgcttgtgcctataaaccttatacaactttcagcaacggatctcttggctctc
>OTU_1751_2
ttaatgattgcgaatggttaccttcggtgctggctctcgcgagcaagtgcacgttggtggctttcatccactactaccctgtgaacctttggcctcttgctagcttcggctggcagaggatttttacacacacactcgaatggaatgaaaacgtttgtcgtgcgcaagcactaatgtacaactttcaacaacggatctcttggctctc
>OTU_1754_8
ttaccgagtttacaactcccaaacccaatgtgaacgttaccaaactgttgcctcggcgggatctctgccccgggtgcgtcgcagccccggaccaaggcgcccgccggaggaccaaccaaaactcttttgtataccccctcgcgggtttttatatctgagccatctcggcgcctctcgtaggcgtttcgaaaatgaatcaaaactttcaacaacggatctcttggttctg
>OTU_1755_1
ttactgaaaaactattttcaagttttttatatcccattgtttacttaccccgttgcttccactggacagatttcatcatgtgtgggatcttttggcttttgtgtataatacttgccaaagagttgccagtggtgagacttttttaccaaaacttgattaaaacacattgtctgaatatatttcttgaatgaaataaaactttcaacaacggatctctaggctctt
>OTU_1758_4
ttaccgagtttacaactcccaaacccctgtgaacataccacttgttgcctcggcggatcagcccgctcccggtaaaacgggacggcccgccagaggacccctaaactctgtttctatatgtaacttctgagtaaaaccataaataaatcaaaactttcaacaacggatctctaggctctt
>OTU_1763_9
ttactgaattgtcaacatgaagttgttgctggtccctatacgggggcatgtgcacgctctgtttacacatccactcacacctgtgcaccctctgtagttctatggcctggggggctctgtcctcctgctgtggctctacgtctttacacacacactgtaacaaagtctcatggaatgtatgtcgcgtttaacgcaatgaaatacaactttcagcaacggatctcttggctctc
>OTU_1767_3
ttatcgaagtctaagagttgtcgctggcccctaggggtcgtgcacgctcgccgttcttcatttctccatctatttgtgcacctcccgtgtggatccatgtcccttgggggacgattggggactgcttcgcgaatacctctttttgcaggctttccttgtgtcggatcctcattttcttcacataccactagaaacaatagagaagggttggaattataaatatatacaactttcagcaacggatctcttggctctc
>OTU_1772_12
ttaaagagtaccggagctctcgggttcctactcccaccctatgttgactttaaatgttgctttggcggaccggcgatctcgccactggcctaggctggacagcgtccgccagaggatttttaaatccctttgaactgaattctgagtcttgaaaattgaatcaaaactttcaacaacggatctcttggttctg
>OTU_1774_7
ttaaagagtaccggagctctcgggttcctactcccaccctatgttgactttgaatgttgctttggcggaccggcagtattgccaccggcctaggctggatagcgtccgccagaggatttttaaattcctttgaatgtgaattctgagtctttgaaaattgaatcaaaactttcaacaacggatctcttggttctg
>OTU_1787_3
ttaccgagttcatgcccttacgggtagacctcccaccctatgtgatattacctttgttgctttggcaggccgtcaggcttcggtccggctaccggctcaggctggtaagcgcctgccagaggaccccaaactctgaatgttagtgtcgtctgagtactatctaatagttaaaactttcaacaacggatctcttggttctg
>OTU_1792_68
ttaccagagatggcgggcctccgtgcccccgtccccaacccattgttgacgcacctttttgcctcgggggcagagcgtccgccgccgcccccggaggatcaccgaacgctgtctctgtgtgtcggagtattgattaaatgaatgaaaactttcaacaacggatctcttggttctg
>OTU_1795_62
ttactgaatggtcatccttgatcttcataccctcaaaccactgtggacacaagatgtgcttcggcgccaatgcgccgcctttatgcagcgagtcatttatgtctgatttaaaaagaattcaaaactttcaacaaaggatctcttggctctc
>OTU_1805_3
ttattgtaaaaccgaggtgcaaaggctgtcgctgaccctcaaaggtcgtgcacgcctgagcgctctcacacaatccatctcacctttgtgcatcaccgcgtgggtcccctttgcgggagggcttgcgttttcacataaaacttgatacagtgtagaatgtttttcttttgcggtcacacgcaatcaatacaactttcaacaacggatctcttggctctc
>OTU_1812_2
ttaccgagtgcgggccctctgggtccaacctcccacccgtgtttattgtaccttgttgcttcggcgggcccgccattctggccgctaaggggcacctgcccctacgcccgcgcccgccgaagacacctcgaactctgtctgaagattgaagtctgagtgaaaatacaaattagttaaaactttcaacaacggatctcttggttccg
>OTU_1815_25
ttaccgagtgagggccctctgggtccaacctcccacccgtgtttatcgtaccttgttgcttcggcgagcccgccacttgtggccgctggggggcacctgcccccgggcccgcgcccgccgaagacaccattgaacgctgtatgaagattgcagtctgagcttattagctaaattagttaaaactttcaacaacggatctcttggttccg
>OTU_1816_84
ttactgaactgtcgacacgagttgttgctggtcctcaaatgggggcatgtgcacgctctgtttgcatatccactcacacctgtgcaccctatgtagttctatggcttgggggaccctgtcctcctgctgtggttctatatctttacacacactctgcaataaagtcttatggaatgtataccgcgtttaacgcaatacaatacaactttcagcaacggatctcttggttctg
>OTU_1821_79
ttactgaactgtcaacacgagttgttgctggtccctatacgggggcatgtgcacgctctgtttacacatccactcacacctgtgcaccctctgtagttctatggcctggggggctctgtcctcctgctgtggctctacgtctttacacacacactgtaacaaagtctcatggaatgtatgtcgcgtttaacgcaatgaaatacaactttcagcaacggatctcttggctctc
>OTU_1823_4
ttaaagagtaccggagctctcgggttcctactcccaccctatgttgactttgaatgttgctttggcggaccggcagtattgccaccggcctaggctggatagcgtccgccagaggatttttaaattcctttaaaaggtgaattctgagtctttgaaaattgaatcaaaactttcaacaacggatctcttggctctc
>OTU_1824_31
ttactgagtgcgggccctctgggtccaacctcccacccgtgtttatcgtaccttgttgcttcggcgggcccgccacttgtggccgccggggggcacctgcccccgggcccgcgcccgccgaagacaccattgaacgctgtatgaagattgcagtctgagcttattagctaaattagttaaaactttcaacaacggatctcttggttccg
>OTU_1830_1
ttaacgagttagggtcttctcggcccgacctcccaaccctatgtttactgaacctttgttgcttcggcgggcccgtctcacgaccgccgggggaccgtaagacgtcctctggcccgtgtccgccggtggcccaactgaacaaattctgattaaaatgtgtcaatgtctgagtagaattcataattaaaacaaaactttcaacaacggatctcttggttctg
>OTU_1834_1
ttactgaactgtcgacacgagttgttgctggtcctcgaaagggggcatgtgcacgctctgtttacacatccactcacacctgtgcaccctctgtagttctgtggcattggggactctgtcctcttgccgtggtcctacgtctttacacacacaccgtaatacagtctcatggaatgtatgtcgcgtttaacgcaatacaatacaactttcagcaacggatatctcggctctc
>OTU_1838_1
ttaccgaaccgtcgacacgagctgttgctggccctcgaaaggggacatgtgcacgctctgtctacacatccactcacacctgtgcaccctctgtagttccatggcctcggggaccccgtccccttgctgtggtcctacgtatttacacacacagtaacaaagtctcatggaatgcagtcgcgtttaacgcaatacaatacaactttcagcaacggatctcttggctctc
>OTU_1840_64
ttaaagagttagggtcttctaggcccgatctcccaaccctttgtttattgaacctctgttgcttcggcggacccgtctcacggccgctggaggaccgctgaaaggcgtcctctagtcagcgtccgccgatagccaaccacttaaactctgaataaatcgtgtcatatgtctaagtctatgattaaattaaagcaaaactttcaacaacggatctcttggctctc
>OTU_1841_53
ttattgaaataaacctgatgggttgttgctggttctctagggagcatgtgcacaccttgtcatctttatatctccacctgtgcaccttttgtagacctttcaggtctatgttgcttcatttaccccaatgtatgttaatagaatgttgtgcctatataatatatacaactttcagcaacggatatctaggctctc
>OTU_1846_53
ttaccgagtgcgggccctctgggtccaccctcccacccgtgtctaccgtcccttgttgcttcggcgggcctgcccccctcggggcggccggggggccctcgcgcccccgggctccgcgcccgccggagacatccacgcgaacactgtgtgaaggttgctgtctgagtggaatttatccaaatcgttaaaactttcaacaacggatctcttggttccg
>OTU_1849_45
ttaacgagttagggtctctcacggcccgacctcccaaccctttgtttactgaacctttgttgcttcggcggacccgtctcacgaccgccgggggaccgtaagacgtcctctggcccgtgtccgccggtggccaaaccgaacaaattctgattaaaatgtgtcaatgtctgagtagaattcataattaaaacaaaactttcaacaacggatctcttggttctg
>OTU_1851_5
ttaccgagttatacaactcatcaaccctgtgaacatacctaaaacgttgcttcggcgggaacagacggccctgtaacaacgggccgcccccgccagaggacccctaactctgtttttataatgtttttctgagtaaacaagcaaataaattaaaactttcaacaacggatctcttggctctg
>OTU_1859_8
ttaccgagtgagggccctctgggtccaacctcccacccgtgtttatcgtaccttgttgcttcggcgggcccgccacttgtggccgccggggggcacctgcccccgggcccgcgcccgccgaagacaccattgaacgctgtctgaagattgcagtctgagcaattagctaaataagttaaaactttcaacaacggatctcttggttccg
>OTU_1865_12
ttaccgagcgagcgtctccgggcgcgacctccaaccctttgtgaacacacctctgttgcctcggggtgacccgggctcgcccggcccccggtggaccatcaaccaactctgcatctttgcgtcggatgtgaaaaaagaaaatggattaaaactttcaacaacggatctcttggttctg
>OTU_1873_39
ttatcgagttagggtcttctcaggcccgacctccttacctttgtctaccttacctcacgttgcttcggcgggcccgtcctgtttttggaccgccggaggcttcaccccctctggcccgttgcccgccgacagccccccaaccaaaaaactcttgcaataactgtgaaattgtctgaattttgcttctaaaataaccaaaaactttcaacaacggatctcttggttctg
>OTU_1878_75
ttactgaagtgatttggggatgttgctggcctcttcttttcgaggggcaatgtgcactccctgaacaattcacaatttccatcacacccttgtgcacatcttgtaggcgcccactcacctctcttggttggggcgtcctatgtattccttcatctcacactttattgaatggtgctcttgaacgcatctcatggattatttgtaaaatagtaatcacagaaacaactttcaacaatggatctcttggctctc
>OTU_1886_10
ttactgagttagggtcttccaggcccgacctccaaccctctgcctaccttacctcttgttgcttcggccggcccgtcccccctagaaataggggtcgaccgccggagggctcacaccctctggtccgcgcccgccgatggccctcaaaccaaaactcttgttcaatcgtgaattgtctgagtatacaaaacaaaataaaccaaaaactttcaacaacggatctcttggctctc
>OTU_1889_8
ttactgaattactgagaggttgtagctgtctcttcggagaatgtgcacgccgctcaaattcatcttaacctcctgtgcactgttgtagactatgataactctcagctacccagtagttggattgaaggacttgcgcttttagctgtctttcaatatcgtagtctatgttttatctatatacaaaaagtctagaatgtcgtccatgggtcttgtacctataaactttatacaactttcaacaacggatctcttggctctc
>OTU_1899_23
ttattgaaataaacctgatgggttgttgctggctctctagggagcatgtgcacaccttgtcatctttatatctccacctgtgcactttttgtagacctttcaggtctatgttgcttcatttaccccaatgtatgttaatagaatgttgtgcctatgtaataatatacaactttcagcaacggatctcttggctctc
>OTU_1902_3
ttagcgagtatggccctcacgggtctaatccaccataaacacctttgtgaaccagtcagggggactcggccgtgaaagcccaggcgattagcggaagggccctcctttgcaaacccgtttagaagtcttgaaagtacttttgtataatctagtaaaactttcagcaacggatctcttggctctc
>OTU_1913_3
ttaccgagttagggtctaaataggcccgacctccaaccctttgtttactataccatgttgctttggcgggcccgcttttcggggctgccgggggctttcatgcccctggtcagtgcccgccagtagccttattaaattcttccataattatgttgtctgagtataaacataaaaatcgttaaaactttcaacaacggatctcttggttctg
>OTU_1917_44
ttaccaaaagataatctttcaacactgaaagatcttacctttgtgctggctttgacagttttgtacttttggggctttaaaatggttcagtagttaaaggagggtttaatccctttttttcatgctactgggtcggccccaaataatcatatcatccttaaaaatttttctgataaattaacacatgattttaataatctgtttaaaacaactttcaacaacggatctcttggttctc
>OTU_1921_17
ttacagagttcatgccctccgggtagatctcccacccagtgctatcactactctcgttgctttggcgggccgctgggccctgcccggccgccggccccggctggcgcgtgcccgccagagaccttcacagactctgaatgttagtgtcgtccgagtaactatacaatcgttaaaactttcaacaacggatctcttggttctg
>OTU_1929_1
ttacagagttcatgcccttcggggtagatctcccacccttgtgtatcattatagaatgttgctttggcgggccgcgtgcctagcacgcctcgattcgcgtcgagcgtgtgcccgccagaggacccctaaactctgaatattggtgtcgtctgagtactatgaaatagttaaaactttcaacaacggatctcttggttctg
>OTU_1931_3
ttaacgagtgagggtcgtccaggcccgacctcccaaccctttgtttaccgaacctctgttgcttcggcggacccgcctcacggccgccggaggattgccgacaggcgtcctctggcccgcgtccgccgatggccaaccactaaaccctgaattaaccgtgtcgtgtgtctcagtctatgattaaattaaatcaaaactttcaacaacggatctcttggttctg
>OTU_1937_48
ttactggaatcgcaagatcccacaacccctgtgaacctaccgaaacgttgcctcggcgggcgcccggccccgcgccgggcgtcgagcccgccggtggcccactactctgtctctgcagcgtggcatctccgagtacaagaaaaacaagtcaaaactttcaacaacggatctcttggctcta
>OTU_1943_1
ttacagagttcatgccctcacgggtagacctcccacccttgtgtatctataccatgttgctttggcaggctgctggacccctcgggggacagcctcagcgccctcgggcctgagagtcgcctgccggaggaaaaacaaactctgaattgttagtgtcgtctgagtactatataatagttaaaactttcaacaacggatctcttggttctg
>OTU_1947_11
ttactgagaatgctctccggagccctcaaaccttgcgtacccatcttgtacttgcgggttgctctcgggcgcccgcgaggattcaaactgtttgaccatcgtcagattcttacaaaagaaaacaactttcaacaatggatctcttggctctc
>OTU_1950_3
ttgtcgaattgtcaacaagagctgttgctggtccctggatgggggcatgtgcacgctctgttaacacatccactcacaccctgtgcaccctctgtagttctatggttgggggggggacctgtcctctcctgctgtggttctgcgtctttacatacacactgtaacaaagtctcatggaatgcatgctgcgtttaacgcaataaaatacaactttcagcaacggatctcttggctctc
>OTU_1951_69
ttaccgagttcatgcccttacgggtagatctcccaccctgtgttatcattacctttgctttggcgggccgccaggctccggtcaggctatcggcttcggctggtacgcgcccgccagaggaccctaacattctgattatcagtgtcgtctgagtactatataatagttaaaactttcaacaacggatctcttggttctg
>OTU_1967_4
ttaccgagtttacaactcccaaacccaatgtgaacgttaccaaactgttgcctcggcggggtcacgccccgggtgcgtaaaagccccggaaccaggcgcccgccggaggaaccaaccaaactcttttctgtagtcccctcgcggacgttatttcttacagctctgagcaaaaattcaaaatgaatcaaaactttcaacaacggatctcttggttctg
>OTU_1969_3
ttaccgagttagggtctaaaacaggcccgacctccaaccctttgtttactataccatgttgctttggcgggcccgcctttcggggctgccggggactttcatgcccctggtcagtgcccgccagtagccttattaaattcttccataattatgttgtctgagtataactataaaatcgttaaaactttcaacaacggatctcttggttctg
>OTU_1970_13
ttacagagttgaaaaactccctaaaccctttgtttatctacctgttcagttgcttcggcaggcggccccagggcggggcctccggcctgcttaggcaggtgcctgccgctggcgcaaaaaactcttgttatatgttaccactctgagtctcaaaaaaaatgaatcaaaactttcaacaacggatctcttggttctg
>OTU_1980_1
ttaccgagttagggtgctctctgcgcccgacctccaaccctttgtctacctgaccagatgttgcttcggcaggcccgtcgccccttcacgggggtggccgccgggggtcctttaccggtcccgggcccgcgcctgtcgatggcccttataaactcttgcataaaacgtgtcgtctgagtttaacaaacaaaataaaacaaaactttcaacaacggatctcttggttctg
>OTU_1982_2
ttactgagaacatgccctttagggtatatctcccaccctttgtttataataccattgttgctttggtgggcccgtcgtatgaccaccggctttggctggtttgtgcctgccagaggaccccaaaattctttattatgttgtctgagtactatgtaatagttaaaactttcaacaacggatctcttggttctg
>OTU_1984_42
ttaccgagttagggtcttctcaggcccgacctccttacctttgtctaccttacctcacgttgcttcggcgggcccgtcctcttttggaccgccggagggttcaccccctctggcccgttgcccgccgacagcccccaaccaaaaactcttgcaataaccgtggaattgtctgaatcttgcttctaaaataaccaaaaactttcaacaacggatctcttggttctg
>OTU_1985_46
ttatcgtacaaaatgtgagagaggcatgcaagggctgtcgctgactccaagtcgtgcacgccggagtgtgccctctcacataataatccatctcaccctttgtgcaccaccgcgtgggcaccctttgggatcagactgatctcggaggatgctcgcgttttcacacaaaccccccttttaaaagtgtagagtgacctcatttatgcaatcaatacaactttcaacaacggatctcttggctctc
>OTU_1987_74
ttcacttgataaaaaggcttctgaagcctttttctctctttcttaaccaagtggggttcttgaattaccactgtgatgctgagatatgggtcttgaagccctttgtgattgctttcaggcatctgatcttatccagcaaaaggttcattgtaaaacccgactgtgagtttgggagtattggaccatatttataatatcactcagttgttggtcaagttgaaaagcaatccactcttggaggggagagaaggatatggttgagggtttgtagcccaaggttgtattctatcttgtctctctctctcttgagtctagagaactcgagataattgtccatattgtggaattatcataacatgagctcatttacaactttcagcaatggatctcttggttctc
>OTU_2000_2
ttacagagaacctgcccttcagggtagatctcccaccctttgttaacattcctttgttgctttggcaggccagacaaatagtctaccggcttaggctggctagtgtctgccagaggggatttaaactcttaatctaatgtctgagtattataaaatagttaaaactttcaacaacggatctcttggttctg
>OTU_2008_11
ttaccgagttagggtctcctaggcccgatctcccaaccctttgtttaccgaacctctgttgcttcggcggacccgcctcgcggccgccggaggactgcccctaagccgtcctctggccagcgtccgccgacagccaacccttctcaactctgaataaatcgtgttgtaaatgtctaagtctatgattaaattaaagcaaaactttcaacaacggatctcttggttctg
>OTU_2010_2
ttaccgagtttacaactcccaaacccctgtgaacatacctattgttgcctcggcggtgcctgttccgacagcccgccagaggaccccaaaccctgattacatttaagaagtcttctgagtaaccgattaaataaatcaaaactttcaacaacggatctctaggctctt
>OTU_2013_15
ttactgagaatgctctccggagccctcaaaccttgcgtatttatcttgtacttgcgggtggccctcgggtgcccgcgaggattcaaactgtttgactgccgtcagatttttgtctaaaaaacaactttcaacaatggatctcttggctctc
>OTU_2017_6
ttacaggactcgcaagactcctacaaccctgtgaacttaactataagcacgttgcttcggcgggtgtctcggcgcgggttctcccgctccggggcgctaagcccgccggcagcctactatactctgtctctttgcgttggcatctcgagtacaaacaaacaagttaaaactttcaacaacggatctcttggttctg
>OTU_2042_3
ttactgagtactacactctctaccctttgtgaactattatacctgttgcttcggcggcgcccgcaagggtgcccgccggtctcatcagaatctctgttttcgaacccgacgatacttctgagtgttcttagcgaactgtcaaaacttttaacaacggatctcttggctcca
>OTU_2048_20
ttaccgagttagggtcttctcaggcccgacctccttacctttgtctaccttacctcacgttgcttcggcgggcccgtcctcttttggaccgccggagggttgaacccctctggcccgttgcccgccgacagcccccaaccaaaaaactcttgcaataactgtgaaattgtctgaattttgcttctaaaataaccaaaaactttcaacaacggatctcttggttctg
>OTU_2054_13
ttaccgagttagggtcttctaagggcccgacctccttacctttgtctaccttacctcacgttgcttcggcgggcccgtcctcttttggaccgccggagggttccaccccctctggcccgttgcccgccgacagcccccaaccaaaaaactcttgcaataactgtggaattgtctgaatcttgcttctaaaataaccaaaaactttcaacaacggatctcttggttctg
>OTU_2057_2
ttacagaaagtaaacgcgggtcaaaccgtgaacttttaaacctttgacgattgactcatgttgcctcggcgggctcgcccgccagaggatacatcaaaactcctgttttaacggcgttgtctgagctacaagcaacgaatcaaaactttcaacaacggatctctaggctctt
>OTU_2059_3
ttaccgagttttcaactcccaaacccactgtgaacatataccattgtttattcgttgcctcggcgggttctatccctggaaacagggcaagcccccgccgggtgacaccaaaaccctgaatgtgtttagttacacagcagtattactctgagtcacatcattttaaatgaatcaaaactttcaacaacggatctcttggttctg
>OTU_2062_7
ttagtgattatgaaaggttgccctctgtgctggctccggcaggtgcacgatggtgactttcatccaacacccctgtgaacctttggcctcttgctggcttcggctggctgaggatttacacaaactcgcatgtattgaggacccttcatgtgcgcaagcactactatatacaactttcaacaacggatatctaggctctc
>OTU_2073_27
ttacagagttgtttaaaactcccaaacccatgtgaacttaccttttgttgcctcggcagggcctattgaggtcatcgcgacctcggtggtggcctgccggtggtctaccaaactcttgttattattgtggaactctgagttgcataaacttaataagttaaaactttcaacaacggatctcttggttctg
>OTU_2084_15
ttactgagtgaggacctctggcccgacctccaaccccatgttatccgaccactgttgcctcgggggcgacccggccttcgggcgtcggggcctccggtggaccttctaactctgcatctttgcgtctgagtggattattgaatcaatcaaaactttcaacaacggatctcttggttctg
>OTU_2087_7
ttactgagaatgctctccggagccctcaaaccttgcgtacccatcttgtacttgcgggttgctctcgggcgcccgcgaggattcaaactgtttgactgtcgtctgattcttacaaagaaaacaactttcaacaatggatctcttggctccg
>OTU_2100_6
ttactagagcaaaggataggcagcgccccacagaagcttgcttcgtggcgggctaccctacttcagtagggtttagagtcgtcgaacctctcggagaagttcggtcctgaactccacccttgaataaattacctttgttgctttggcgggccgcctcgtgccagcggcttcggctgttgagtgcccgccagaggaccacaactcttgttttttatgatgtctgagtactatataatagttaaaactttcaacaacggatctcttggttctg
>OTU_2101_3
ttcataataagtgttttatggcactttttaaatccatatccaccttgtgtgcaatgtcagtcgatcttcttcatggagatcgaccaaacatcaacctttattttttaactctttgtctgaaaaatattatgaataaacaattcaaaatacaactttcaacaacggatatctcggctctc
>OTU_2115_61
ttaatgagttttgatgtgggtctcttgagctggcattgcaggtgctcggaggctcccattctcatccactcaaccccctgtgcacttctgaacgcaagtgagtcgtcagtccccatgctgggattgacttgatttacttcgtttattacaaactctttttatgtcttgtagaatgcattgcctctttacaggcgaaatgtaatacaactttcaacaacggatctcttggctctc
>OTU_2130_1
ttattgactgtgaatcgttaccttctgtgctggctccggcaagtgcacgttggtgactttcattcccaacacccttgtgaacctttggcctcttgctagcctcggctggcagaggatttacacacagactcgaatgtaatgaaaaacctcttgttgtgcgcaagcacgaatgtacaactttcaacaacggatctcttggttctg
>OTU_2131_1
ttactgaagcagttagggttgtagctgactctgtcaaaggagtactgtgctcgctctattcttctacttttccccctgtgcaccttttgtaggctatgatatccatcgtgtgtgagttcgcgctcattaccggttcaagggattgctggtttactccggctgctcttgcccttcatatgccttatgtctttatacactctttacaagtctagaatgtcttttgtgggtctattgacctataaacttaatacaactttcaacaacggatctcttggttctg
>OTU_2135_9
ttaacgagttagggtcttccaggcccgacctcccaaccctatgtttactgaacctctgttgcttcggcggacccgccttacggccgccggaggaccgccgcgaggcgtcctctggcccgcgtccgccggtggccaactccaaaactcttgaatgaatcgtgtcatattctaagtctatgattaaattaaagcaaaactttcaacaacggatctcttggttctg
>OTU_2136_51
ttaccgagtgcgggccctctgggcccaacctcccacccgtgtttcgttattaccttgttgcttcggcgggcccgccgttccggccgccggggggctttcacgtccccgggcccgcgcccgccgaagacacccccgaacgctgcctgaaggttgcagtctgagtgatcatgataaatcagttaaaactttcaacaacggatctcttggttccg
>OTU_2137_18
ttacagagttcatgccctcacgggtagatctcccacccttgaatactatacctttgttgctttggcgggccgcttcggctactggctctagctggtgagtgcccgccaaaggatcctaaaactctgaatatttgtgtcgtctgagtactatataatagttaaaactttcaacaacggatctcttggttctg
>OTU_2149_10
ttactgagaacatgccctttagggtatatctcccaccctttgtttacaataccattgttgctttggtgggcccgtcatatgaccaccggctttggctggtttgtgcctgccagaggaccccaaaactctttattatgtcgtctgagtactatgtaatagttaaaaaactttcaacaacggatctcttggctctc
>OTU_2159_3
ttacagaaagtaaacgcggatcgaaccgcgaacttctaaacctttgacgattgactcatgttgcctcggcgggctcgcccgccagaggatacatcaaaactcctgttttaacggcgttgtctgagctacaagcaacgaatcaaaactttcaacaacggatctcttggttctg
>OTU_2161_9
ttacagagttcatgcccgaaagggtagacctcccacccttgtgtattattactttgttgctttggcgagccgcccccgggcctcaggctttcgagcctgagcgcgctcgccagaggataccaaaactctttttattaatgtcgtctgagcactatataatagttaaaactttcaacaacggatctcttggttctg
>OTU_2164_1
ttacagagttcatgcccctcggggtagatctcccacccttgtgtatcattatagaatgttgctttggcgggtcgcgcctcgtgcgcctagattcgcgtctagcgtgcccgccagaggacccctaaactctgaatattggtgtcgtctgagtactattcaatagttaaaactttcaacaacggatctcttggttctg
>OTU_2166_4
ttaccaatcatgtctcccagccgaaaggttggtgcaggcgcttaaccctttgtgaaccaaaaaacctttcgcttcggcagcagctcggttggcaacagcctctgcgtcagcctgccggtagcaccaatcatcaaaacttgcagttaataacattgtctgattaccaaattttcgaatgaaaatcaaaactttcaacaacggatctcttggttccc
>OTU_2177_22
ttaccgagttcatgcccttacgggtagatctcccaccctgtgttatcattacctttgttgctttggcgggccgccaggctccggtcaggctatcggcttcggctggtacgcgcccgccagaggaccctaacattctgattatcagtgtcgtctgagtactatataataatagttaaaactttcaacaacggatatctaggctctc
>OTU_2182_41
ttactgaattgtcaacatgaagttgttgctggccctcatgtgggggcatgtgcacgctctgtttacacatccgttcacacctgtgcactctctgtagttctgtggtttgggggctctgtcctcctaccgtggtcctatgtatttacacatacactgtaataaagtcttatggaatgtatgccgcgtttaacgcaatacaatacaactttcagcaacggatctcttggctctc
>OTU_2184_4
ttactgagtgagggccctctgggtccaacctcccacccgtgtttattgtaccttgttgcttcggtgagcccgcctcacggccgccggggggcttctgcccccgggtccgcgctcaccggagacacctttgaactctgtatgaagattgcagtctgagcgaaaactaaataagttaaaactttcaacaacggatctcttggttccg
>OTU_2186_64
ttactgaactgtcaacacgagttgttgctggtcctcaaatgggggcatgtgcacgctctgtttacatacccactcacacctgtgcaccctctgtagttctgtggtgtgggggactctgtcctcccgctgtggttctacgtctttacacacacacagtttcatagaatgtatgtcgcgtttaacgcaatgaaatacaactttcagcaacggatctcttggctctc
>OTU_2190_14
ttcataatcaagtgtttttatggcactttcaaaaatccatatccaccttgtgtgcaatgtcatctcactggagatcaacggctgtaaaaatccgttggtcacctttgggatttatatctactcagaactttagtgattttgtctgaaacatattatgaatacttaattcaaaatacaactttcaacaacggatctcttggctctc
>OTU_2196_4
ttaccgagttatacaactcccaaacccactgtgaaccttaccaaatcgttgcttcggcgggaccgccccgtgccctcgcggcccggacccaggcgcccgccggaggccaaaaactctcctgttttctcctagcggaatttatctgagtggccctcaggggcaagcaaatgaatcaaaactttcaacaacggatctcttggttctg
>OTU_2197_8
ttaccgagtaccggagcctccgggttcctactcccaccctatgttgactttaaatgttgctttggcggaccggtggttctcaccgctggcccaggctggagagcgtccgccagaggattttaaactctgtttaacggtgaattctgagtcttgataaattgaatcaaaactttcaacaacggatctcttggttctg
>OTU_2200_63
ttattgaaattaaaatttggctgcgttgttgctggctcttaggagcatgtgcacatgcaccaatttttttttttttaaaaaccacctgtgcacacactgtagatctggattgaactttttcgacgattattaactcggttttgagaactgctttggctgttcttgtgttctccaggtctatgttttaaacaatatactccaacaagtaatagaatgtcaaattatgggccttttgtgcctttaaaatctaatacaactttcaacaacggatctcttggctctc
>OTU_2207_1
ttaccgaattgtcaacacgagttgttgctggtcctcaaaacgggggcatgtgcacgctctgtttacacatccactcacacctgtgcaccctctgtagttctatggcctggggggctctgtcctcctgctgtggctctacgtctttacacacacactgtaacaaagtctcatggaatgtatgtcgcgtttaacgcaatacaatacaactttcagcaacggatctcttggctctc
>OTU_2213_60
ttattgaataaacttgaacaggctgttgctggtccctcttcagggacacatgtgcacgcttgtcatctttatctctcctcatgtgcacattttgtagaccctggtgtttgaggattgcttattttgctctccttgtttgggttatatcccaggtctatgttatttttcacaatctctttgaaatgtattggaatgtcaataataataaaaagttgatacaactttcagcaacggatatctaggctctc
>OTU_2214_55
ttactgaattgtcaacatgaagttgttgctggccctcatgtgggggcatgtgcacgctctgtttacacatccgttcacacctgtgcactctctgtagttctgtggtttgggggctctgtcctcctaccgtggtcctgcatatttacacatacactgtaataaagtcttatggaatgtatgccgcgtttaacgcaatacaatacaactttcagcaacggatctcttggctctc
>OTU_2219_1
ttacagagttcttgcccttacgggtagatctcccacccttgatatcttattctttgttgctttggcgggacgcactttgctacaggctttagcttgtaagtgcccgccagagaccaatcaaaacctgtttatgtgtcgtctgagtactatataatagttaaaactttcaacaacggatctcttggttctg
>OTU_2221_9
ttagtgattatgaaaggttgccctctgtgctggctccggcaggtgcacgatggtgactttcatccaacacccctgtgaacctttggcctcttgctggcttcggctggctgaggatttacacaaactcgcatgtattgaggacccttcatgtgcgcaagcactaatgtacaactttcaacaacggatctcttggctctc
>OTU_2222_9
ttaacgagttagggcctccgtgcccgacctccaaccctcttgtatcacaacacctcgttgcctcgggggtggccctgcctcacggcgggccccccggaggaccactcaaacactgcattttaatgtcgtctgagttagattttaaatgaaatcaaaactttcaacaacggatctcttggttctg
>OTU_2224_61
ttacaggaagccgaaaggcaacttcaaaccattgcgaacgtatccaagttgcttcggcggcgcgggacccctcgggggaccgcagcccacgccctccggggcgaggggcgcccgccggaggtcacaaactctcgtgtattacggtggcgtctctgagtaaaaaaataaataagtcaaaactttcaacaacggatctcttggctctc
>OTU_2228_16
ttactgagtgtaaaaactcccaaacccctgtgaacataccatctgttgcttcggcgggatcgccccgggcgcctttgcgtgccccggatccaggcgcccgccggaggactccaaactcttgtttttatatgtggcattatctgagtggcttatagcaaaataaatcaaaactttcaacaatggatctcttggctctg
>OTU_2229_1
ttatcgttgggcttcggcccattcgagataacacccttgcctttttgagtaccttttcgtttcctcggcaggctcgcctgccaacggggacccttcaaaacgctttgtaatacctgtaattgtctgatataacaagcaaaaatcaaaactttcaacaacggatctcttggttctg
>OTU_2231_10
ttaaagagttagggtcctctgggcccgacctcccaaccctttgtttactgaacctctgttgcttcggcggacccgtctcacgaccgccggaggatcgcccgtccaggcgtcctctggccagcgtccgccgatagccaacccttcaaactcctgaataaatcgtgtcatatgtctaagtctatgattaaattaaagcaaaactttcaacaacggatctcttggttctg
>OTU_2236_20
ttacagagaaattgccccttggggtagatctcccaccctttgtttacattacctttgttgctttggcaggcccgtctttttgactaccggcttaggctggtccgcgcctgccagaggaccctaaactcttgattttagtgttgtctgagtaatatttaaaataagttaaaactttcaacaacggatctcttggttctg
>OTU_2239_2
ttaccgagttcatgcccttacgggtagatctcccaccctgtgttatcattacctttgttgctttggcgggccgccaggctccggtcaggctatcggcttcggctggtacgcgcccgccagaggaccctaacattctgattatcagtgtcgtctgagtactatattttaatagttaaaactttcaacaacggatctcttggttctg
>OTU_2241_2
ttcataataagtgttttatggcactttttaaatccatatccaccttgtgtgcaatgtcagtcgatcttcttcatggagatcgaccaaacatcaacctttattttttaactctttgtctgaaaaatattatgaataacttaattcaaaatacaactttcaacaacggatctcttggctctc
>OTU_2251_5
ttactgaattactgagaggttgtagctgtctcttcggagaatgtgcacgccgctcaaattcatcttaacctcctgtgcactgttgtagactatggtaactctcaactacctagtagttggattgaaggacgtgcgcttttagctgtctttcaatatcgtagtctatgttttatctaaatacaaaaagtctagaatgtcgttcatgggtcttgtacctataaactttatacaactttcaacaacggatctcttggctctc
>OTU_2256_1
ttaccgagttagggtagtcactcactgcccgacctcccaaccctgtgtctaccacactttgtcgttgcttcggcggaccggttgaccaactggtcgtgaccgccgggggttggctctgtcccccctggagagcgtccgtcgatggcccaaccacaaaatctcttgtaccgaaacgtgtcgtctgaattattgagaaatcaaaaaaacaaaactttcaacaacggatctcttggctctc
>OTU_2257_1
ttactgaaaaacttctaaagttttttatatcccattgtttacttaccccgttgcttccactggacagatttcatcttgtgtggaatcttttggcttgtatgaacaatacttgccaaagagtttgccagtggcaagatcttttttcaccaaaacttgattaaaacacattgtctgaatatatttcttgaatgaaacaaaactttcaacaacggatctctaggctctt
>OTU_2264_10
ttcataataagtgttttatggcactttttaaatccatatccaccttgtgtgcaatgtcagtcgatcttcttcatggagatcgaccaaacatcaaccttatcttttaactctttgtctgaaaaatattatgaataaacaattcaaaatacaactttcaacaacggatatctaggctctc
>OTU_2266_22
ttaccgagttattctactcccaaacccctgtgaacttatacctttactgttgcttcggcgggttaacgccccggaaggcccgcgagggccgccggaaccaggcgcccgccgggggaccaaaactcttgtatcttttttatagcatgtctgagtggaatcataaacaaatgaatcaaaactttcaacaacggatctcttggttctg
>OTU_2267_34
ttacagaaagtaaacgcggatcaaaccgcgaacttctaaacctttgacgattgactcatgttgcctcggcgggtcctcccgccagaggatacatcaaaactcctgttttaacggtgttgtctgagctacaagcaacgaatcaaaactttcaacaacggatatctaggctctc
>OTU_2278_3
ttatcgagtgaagggttctctaagaacctgacctcccaacccttgtgttctttaccactttgctttggcgggcccgtcactgctgtgaccgccgaaaggttatgtttttaactaacctttgggcccgtgcccgccagagatatctgaacgcttgttatgaaataggttgtctgagttataaacaaaaataagtcaaaactttcaacaacggatctcttggttccg
>OTU_2284_21
ttaccgagttagggtcttctaagggcccgacctccttacctttgtctaccttacctcacgttgcttcggcgggcccgtcctcttttggaccgccggagggttgaacccctctggcccgttgcccgccgacagcccccaaccaaaaaactcttgcaataactgtggaattgtctgaatcttctaaaataaccaaaaactttcaacaacggatctcttggttctg
>OTU_2289_1
ttaccgagtaccggaactctcgggttcctactcccaccctatgttgactttaatgttgctttggcggaccggttaagaccaccggcctatgctggatagcgtccgccagaggattcttaaatacttctaatgggaattctgagtctttgaaaattgaattaaaactttcaacaacggatctcttggttctg
>OTU_2293_57
ttactgattgcgaatcgttgccttcagtgctggccggcttcggcaagtgcacgttgctttcatccaataccctgtgaacctttggcctcttgctagcttcggccggcagaggatttttacacacactcgaatgtaatgaaatttattgtcgtgcgcaagcactaatgtacaactttcaacaacggatctcttggctctc
>OTU_2302_1
ttaacgagttagggtcttctcaggcccgacctccccaaccctttgtttaccgaacctctgttgcttcggcgggcccgtctcacgaccgccggaggaccgccgacaggttgtcctctggcccgtgtccgccgacagccaacctctcaaactctgtatgaatcgtgtctttatctctaagtctatgatttaaaacaaaatcaaaaagcaaaactttcaacaacggatctcttggttctg
>OTU_2305_4
ttatcgtaaaacagaggtgcgcgggctgtcgctgactttttaagtcgtgcacgcccgagcgctctcacacaatccacgtcacccctatgtgcaccaccgcgtgggtcccccccttgaaaggggggctcgcgttttcacacaaacttgaagtagtgtagaatgtctttttttgcgatgacacgcaatcaatacaactttcaacaacggatctcttggctctc
>OTU_2313_59
ttatcgattccattctgaagaggtagagagactgtggctggccgtctatatacctatataagaccgcatgtgcacgtcccctcgccttttctatctacacacacctgtgcacctattgtagatccccatccccttttgcctttgaaagagggagggaacctatgtttttcatcacatcacacgcaattgtatgtctagaatgtaatttcattgatcattgatcattgataaaaatataatacaactttcagcaacggatctcttggttctg
>OTU_2316_1
ttactgaattgtcaaacgggttgttgctggccctcatagggggcatgtgcacgctctgtttacacatccactcacacctgtgcaccctctgtagttctatggtttgggggaccctgtcttccttctgtggttctacgtctttacacacacactgtaataaagttttatggaatgtacatcgcgtctaacgcaatacaatacaactttcaacaacggatctctaggctctt
>OTU_2317_1
ttattgactgtgaatcgttgccttcagtgctggctcttaggagcaagtgcacgttggtggctttcatccaacaccctgtgaacctttggcctcttgctagcttcggctggcagaggatttttataacacactcgaatgtaatgagaactattgtcgtgcgcaagcactaatgtacaactttcaacaacggatctcttggctctc
>OTU_2321_1
ttaccgagtttacaactcccaaacccctgtgaacatacctatttgttgcctcggcggtgcctgttccgacagcccgccagaggaccccaaaccctgattacatttaagaagtcttctgagtaaaccgattaaataaatcaaaactttcaacaacggatatctcggctctc
>OTU_2324_56
ttattgattgcgaatcgttgtctccagtgctggccaggtcactctggcaagtgcacgtagacagctttcatccaataccctgtgaacctttggcctcttgctagcttcggcgagcagaggattttacacccactcgcatgtaatgaaattattgttgtgcacacgcactactatacaactttcaacaacggatctcttggttctg
>OTU_2335_2
ttactgagtgcgggccctctgggtccaacctcccacccgtgtttatcgtaccttgttgcttcggcgggcccgcctcacggccgccggggggcttctgccctcgggcccgcgcccgccgaagacaccattgaacgctgtctgaagattgcagtctgagcaattagctaaataagttaaaactttcaacaacggatctcttggttccg
>OTU_2342_2
ttaataaaaggataccttcgggtataccccatccgtgtctacatactcttgttgctttggcaggccgtggtctcccactgtgggctctgcctgcatgtgcctgccagaggaccaaactctgaatgttagtgatgtctgagtactatataatagttaaaactttcaacaacggatctcttggttctg
>OTU_2345_1
ttactagagcaaaggacaggcagcgccccacagaagcttgcttcgtggcgggctaccctacctcggtagggtttagagccgtcgagcctctcggagaagttcggtcctgaactccacccttgaataaattacctttgttgctttggcgggccgcctcgttccagcggcttcggctgttgagtgcccgccagaggaccacaactcttgtttttagtgatgtctgagtactatataatagttaaaactttcaacaacggatctcttggttctg
>OTU_2349_14
ttaccgagttcatgcccttacgggtagatctcccaccctgtgttatcattacctttgttgctttggcgggccgccaggctccggtcaggctatcggcttcggctggtacgcgcccgccagaggaccctaacattctgattatcagtgtcgtctgagtactatataatagttaaaaaactttcaacaacggatctcttggttctg
>OTU_2351_3
ttaaagagttagggtcttctaggcccgacctcccaaccctatgtttattgaacctctgttgcttcggcggacccgccttatgggccgccggaggaccgctgaaaggcggttctctggccagcgtccgccggcagccaaccacttaaaactctgaataaatcgtgtcatatgtctaagtctatgattaaattaaagcaaaactttcaacaacggatctcttggttctg
>OTU_2357_2
ttaaccaaaaggatacctttatggtatacctcatccgtgtctacatactcttgttgctttggcaggccgtggtcttgaccctgtgggctctgcctgcatgtgcctgccagaggaccaaactctgaattttagtgatgtctgagatactatataatagttaaaactttcaacaacggatctcttggttctg
>OTU_2363_1
ttaacgaatatctatggcgttggttgtagctggctcctaggagcattgtgcacgcccgtcattcatatcatctttccacctgtgaaccatgtgtaggcctggatacccctcgctttggcaacaaagcggatgcaaggattgctgcgtcgacaaggccggctctctttgaatttccaggttctatgttttttacacaccccatttgaatgatttagaatgtagtcaatgggctttcatgcctataaaaaaactatacaactttcagcaacggatctcttggctctc
>OTU_2371_3
ttaccgagaaactgccctttgggtagatcttccaccctttgtttacattacctttgttgctttggcaggcccgtctttggaccgccggcttaggctggtctgtgcctgccagaggatcctaaaactcttgatttttgtattgtctgagtaatattataagttaaaactttcaacaacggatctcttggttctg
>OTU_2374_7
ttagtgaatcttcaaagtcggctcgtcggattgtgctggtgggaaaccacatgtgcacatctacgggtcgcaaacccacacacctgtgcatctatgactctgagcaccgctttgcatggcccccttgatttgggcctggtgctcgagtactttcacacactctcgaatgtaatggaatgtcttgttgtgcataacgtacaaacagaaacaactttcaacaacggatctcttggctctc
>OTU_2375_55
ttatcgtacaacggaggcacaagggctgtcgctgactccaagtcgtgcacgccggagtgtgccctctcacataataatccatctcaccctttgtgcaccaccgcgtgggcaccctttgggatcagactgatctcggaggatgctcgcgttttcacacaaaccccccttttaaaagtgtagagtgacctcatttatgcgctaacccgcaatcaatacaactttcaacaacggatctcttggctctc
>OTU_2379_1
ttactgaactgtcgacacgagttgttgctggtcctcgaaagggggcatgtgcacgctctgtttacacatccactcacacctgtgcaccctctgtagttctgtggccttggggactcccgtcctcttgctgcggtcctacgtctttacacacacaccgtaatacagtctcatggaatgtatgtcgcgtttaacgcaatacaattacaactttcagcaacggatctcttggttctg
>OTU_2383_5
ttaccgagtgagggctctcggcccgacctcctaccctttgttgaccaacacctgttgcctcaggggcgacccggcctccgcgccggcccccggcggaccactttaatgctgtttctatacgtcggagtacttgatgaatcaatcaaaactttcaacaacggatctcttggttctg
>OTU_2387_3
ttactgagttagggtcttccaggcccgacctccaaccctttgcctaccttacctcttgttgcttcggccggcccgtcccccctagaaataggggtcgaccgccggagggctcacaccctctggcccgcgcccgccgatggccctcaaccaaaactcttgttcaatcgtgaattgtctgagttaaaaaacaaaataaaccaaaaactttcaacaacggatctcttggttctg
>OTU_2388_1
ttactaaaaaaaactgtttttaaaacgttttttatatcccattgtttacctaccctgttgcttccactggacagatttcatcttgtatgggatcttttggcttttgtatgaataatacttgccaaagagttgccagtggctagactattttaccaaaaacttgattaaaaaaaacacattgtctgaatatatttgctgaatgaaataaaactttcaacaacggatctctaggctctt
>OTU_2390_4
ttaccgagttcatgcccttacgggtagacctcccaccctatgttatattaccttcgttgctttggcaggccgtcaggcttcggtcaggctaccggctcaagctggtaagcgcctgccagaggaccctaaactctgaatattagtgtcgtctgagtactataaaatagttaaaactttcaacaacggatctcttggttctg
>OTU_2391_1
ttacaggactcgcaagactcctatcaccctgtgaacttacctataagcacgttgcttcggcgagtgtcccagagagggtccgccctccccgggacgctacgctcgccggcagcctactaaactctgtttctttgcgttggcatctcgagtaaatcaaacaagttaaaactttcaacaacggatctcttggttctg
>OTU_2396_3
ttagtgaaaaacttttcaaagttttttatatcccattgtttactttccctgttgcttccactggacagatttcatcttgtgtgggatcttttggcttgtatgaataatacttgccaaagagttgccagtggctagacttttttaccaaaacttgatataaaacacattgtctgaatatatttcttgaatgaaaataaaactttcaacaacggatctctaggctctt
>OTU_2398_4
ttaaagagtaccggagctctcgggttcctactcccaccctatgttgactttgaatgttgctttggcggaccggcagtattgccaccggcctaggctggatagcgtccgccagaggatttttaaattcctttgactgaattctgagtcttgaaaattgaatcaaaactttcaacaacggatctcttggttctg
>OTU_2407_2
ttagcgagtatggccctcacgggtctaatccaccataaacacctttgtgaaccagtcagggggactcggccgtgaaagcccaggcgattagcggaagggccctcctttgcaaacccgtttagaagtcttgaaagtacttttgtataatctagtaaaacttatagcaacggatctcttggttctg
>OTU_2415_7
ttactgaactgtcgacacgagttgttgctggtcctcgaaagggggcatgtgcacgctctgtttacacatccactcacacctgtgcaccctctgtagttctgtggcattggggactctgtcctcttgccgtggtcctatgtctttacacacacaccgtaatatagtctcatggaatgtatgtcgcgtttaacgcaatacaatacaactttcagcaacggatctcttggctctc
>OTU_2416_53
ttaatgattgcgaatggctgccttcagtgctggctcttatgagcatgtgcacgttggtggctttcatccaatacccatgtgaacctttggcctcttgctagctttggccggcagaggatttttacacacactcgaatgtaatgaaatttattgtcgtgcgcaagcactaatgtacaactttcaacaacggatctcttggctctc
>OTU_2418_7
ttaaagattcgacctttgggtctatcttctcttcagctgtgcgcttttgctgcacgctgttgaacttcacacacctgtgcacacttcggttgcggctagttaaatctggctgcttccgcttttacatttaacaaccaagtctagaatgtcaaatactataaaataacaacttttaacaacggatctcttggctctc
>OTU_2437_3
ttactgagttagggtcttccaggcccgatctcccaaccctttgtttacttataccatgttgctttggtgggcccgcctttagaggccgccggggacttacactctctggtcagtgctcgccagtagccttcttaaattctattataattatgtcgtctgaataataaaatataaatcgttaaaaactttcaacaacggatctcttggttctg
>OTU_2441_1
ttacagagactctgccctttgggtagacctcccaccctgtgtcgttatacctctgttgctttggcgggccgtggggccccggccctgcccctggctccggctagggcgcgcccgccagaggacctcaaaacctgaatgttagtgtcgtctgagtactatataatagttaaaaaactttcaacaacggatctcttggttctg
>OTU_2447_2
ttattgaatgaaacttctgactggatgttgttgtgctggccctgtggggcaatgtgcacaccctctggtcatctgtttcttctgttccacctgtgcactgtctgtagacacactctgtgtggtctatgatatgttttacaaatacacacatatacacagatctctgggcaatgtacataaataaaattacaactttcaacaatggatctcttggctctc
>OTU_2448_18
ttaccgagttagggtcttctcaggcccgacctccttacctttgtctaccttacctcacgttgcttcggcgggcccgtcctcttttggaccgccggagggttcacccccctctggcccgttgcccgccgacagccccaaccaaaaaactcttgcaataactgtgaaattgtctgaattttgcttctaaaataaccaaaaactttcaacaacggatctcttggctctc
>OTU_2451_13
ttaccgagttagggtcttctcaggcccgacctccttacctttgtctaccttacctcacgttgcttcggcgggcccgtcctcttttggaccgccggagggttcactctcctctggcccgttgcccgccgacagcccccaaccaaaaaactcttgcaataactgtggaattgtctgaatcttgcttctaaaataaccaaaaactttcaacaacggatctcttggttctg
>OTU_2454_2
ttatcaatgggtccaaagactcttaaaacctaccgtgctcccctacgtggataacaggaaagccccccggggcattggaaatctgagaagttgaagaactgcgccccggcgcatgactaagacaaaaattacaacccataacaatggatctcttggctctg
>OTU_2462_3
ttacagagttgcaaaactccaacaaaccatcgcgaatcttacccgtacggttgcctcggcgctggcggtccggaaggccctcgggcccccggatcctcgggtctcccgctcgcgggaggctgcccgccggagtgccgaaaccaaactcttgatattttatgtctctctgagtaaacttttaaataagtcaaaactttcaacaacggatctcttggttctg
>OTU_2465_28
ttaccgagtggagggcctccgggtccgacctccaaccctttgttgaccgacacctgttgcctcgggggcgacccggacgccgacgcgtcgggccccccgacggaccattcaaacactgtgtctgtacgtcggagcacttgataaatcaattaaaactttcaacaacggatctcttggttctg
>OTU_2468_52
ttcacttgataaaaaaggcttctgaagcctttttctctctttcttaaccaagtggggttcttgaattaccactgtgatgctgagatatgggtcttgaagccctttgtgattgctttcaggcatctgatcttatccagcaaaaggttcatattgtaaaacccgactgtgagtttgggagtattggaccatatttataatatcactcagttgttggtcaagttgaaaagcaatccactcttggaggggagagaaggatattggttgagggtttgtagcccaaggttgtattctatcttgtctctctcttgagtctagagaactcgagataattgtccatattgtggaattatcataacatgagctcatttacaactttcagcaatggatctcttggttctc
>OTU_2470_10
ttagtgaacgcccttttgggcttataactatccaaacctctgtgaactgtgcccttcggggctttttaatacaaacactgtgtaatgaacgttatcattttaacaaaacaaaactttcaacaacggatctcttggctctc
>OTU_2472_2
ttatagagtcttgaactccaaaccactgtgaacataccactgttgcttcggcgggttcccggcccaggccgccccgccggaggtcgcaaactctgttttctagcgaatcttctgagtcttaaataagcaatcaaaactttcagcaacggatctcttggctctg
>OTU_2474_3
ttactgaattgtcaacaagagttgttgctggtcctcgagagggggcatgtgcacactctgtttacacacccactcacacctgtgcaccctctgtagctctgtggtgtgggggccctgtcctcccactgtggttctacgtctttacacacacacagtgataagtctcatggaatgtatgtagcgtttaacgcaatacaatacaactttcagcaacggatctcttggctctc
>OTU_2479_2
ttactgagaacatgccctttagggtatatctcccaccctttgtttataataccattgttgctttggtgggcccgtcgtatgaccaccggctttggctggtttgtgcctgccagaggaccccaaaactctttattatgtcgtctgagtactatgtaatagttaaaaaactttcaacaacggatctcttggttctg
>OTU_2489_3
ttaccgagttgcaaaactccaaaccattgtgttcctgaattctctgttgcctcggcaggccgccccagggcggggcgcctacaaggcctgccgaaagcacccctaaaaaactcttctgaaacgcgtcttctgaagcactaagaaataaatcaaaactttcaacaacggatctcttggttctg
>OTU_2493_5
ttacagagactctgccctttgggtagacctcccaccctgtgtcgttatacctctgttgctttggcgggccgtggggccccggccctgcccctggctccggctagggcgcgcccgccagaggacctcaaaacctgaatgttagtgtcgtctgagtactatataataatagttaaaactttcaacaacggatctcttggttctg
>OTU_2499_3
ttaccgaattgtcaacacgagttgttgctggtcctcaaacggggacatgtgcacgctctgtttacacatccactcacacctgtgcaccctccatagttctgcagcctgggggctctgtccccctgatgcggttctatgtatttacacacacacactgtagtaaagtctcatggaatgcataccgcgtttaacgcaatataatacaactttcagcaacggatctcttggttctg
>OTU_2502_43
ttaatgattgcgaatgggtcaccttcagtgctggctctcaagagcaagtgcacgttggtggctttcatccaattccacaccctgtgaacctttggcctcttgctagcttcggctggcagaggattttacatactcgaatgtaatgaaatttattgtcgtgcgcaagcactaatgtacaactttcaacaacggatctcttggctctc
>OTU_2505_10
ttacagagactctgccctttgggtagacctcccaccctgtgtcgttatacctttgttgctttggcgggccgcggggcttaggccctgcccctggctccggctagggcgcgcccgccagaggacctcaaaacctgaatgttagtgtcgtctgagtactatataataatagttaaaactttcaacaacggatctcttggttctg
>OTU_2506_7
ttagtgaacgccctcacgggcttataactattccaaacctctgtgaaccgtgcccttcggggctattttacaaacatggtgtaatgaacgtcatatatcataacaaaaacaaaactttcaacaacggatctcttggcttctc
>OTU_2510_1
ttaccgagttagggtcttccaggcccgacctccaaccctttgttaactataccatgttgctttggtgggcccgcctttaggggccgccggggactttcacgcccctggtcagtacccgccagtagccttcttaaattccttcataattgtgtcgtctgagtatacttataaatcgttaaaactttcaacaacggatctcttggttctg
>OTU_2513_41
ttaccgagattacatctcccaaacccctgtgaacatacctttatagttgcttcggcgggtctttgccctggaaacaggtgcccgccggggaccccaaacccttgattttttactagattgtctgagtggaatcatatcaaatgaatcaaaactttcaacaacggatctcttggttctg
>OTU_2519_1
ttaatgattgcgaatcgtcgccgtcagtgctggccggcttcggcaagtgcacgtcggtgactttcatccaataccctgtgcacctttggcctcttgctagcttcggccggcagaggatttttacacactcgaatgtaatgaaaactattgtcgtgcgcaagcactaatgtacaactttcaacaacggatctcttggctctc
>OTU_2520_12
ttatcgtacaaaatgtgtgaggcatgcgagggctgtagctgactcaaagttgtgcacgccggagtgtgtcctctcacataacaatccatctcaccctttgtgcaccaccgcgtgggcaccctccgatctcggagggggctcgcgttttcacacaaaaccccccccctttaaaaagtgtagaatgacctcatttatgcgctaacccgcaatcaatacaactttcaacaacggatctcttggctctc
>OTU_2532_46
ttattgaataaatctggcgtggttgtagctgactctcaggagtatgtgcacacccgtcacctttatctttccacctgtgcacacactgtagtcctggatacctctcgccgaaaggcggatgcggaggctgctgtgcccgcaagggccagcttcctctgaatttccaggtctatgatttttatacaccccaaacgaatgtcttagaatgtctttacaaggccttgtgcctataaacctatacaactttcagcaacggatctcttggctctc
>OTU_2534_10
ttcataataagtgttttatggcactttttaaatccatatccaccttgtgtgcaatgtcagttgattttctttatggagatcgaccaaacatcaacctatttttttaactctttgtctgaaaaatattatgaataaacaattcaaaatacaactttcaacaacggatctcttggctctc
>OTU_2535_8
ttactgagttagggtcttccaggcccgacctccaaccctttgcctaccttacctcttgttgcttcggccggcccgtcccccctagaaataggggtcgaccgccggagggctcacaccctctggcccgcgcccgccgatggccctcaaccaaaactcttgttcaatcgtgaattgtctgagtatacaaaacaaaataaaccaaaaactttcaacaacggatctcttggctctc
>OTU_2536_46
ttgtcgaacgtcgacacgagttgttgctggtcctcgaatgggggcatgtgcacgctctgtttacacatccactcacacctgtgcaccctccgtagttctatggccttgggggcctctgtcccctttgcccacggttctacgtctttacacatacaccgtaacaaagtctcatggaatgtatgcagcgtttaacgcaatacaatacaactttcagcaacggatctcttggctctc
>OTU_2537_8
ttaacgagttagggtcttgtaggcccgacctcccaaccctttgtttatcgaacctctgttgcttcggcggacccgtctcacgaccgccggaggaccgctgaaaggcgtcctctggccagcgtccgccgatagccaaacacttaacattctgaatgaatcgtgtcttctgtctaagtctatgattaaataaaaagcaaaactttcaacaacggatctcttggttctg
>OTU_2540_1
ttaccgagttagggtctacctctaggcccgacctccttacctctgtttacctgacctcacgttgcttcggcgggcccgtccccttaaccaggcgaccgccggaagggctcaccccttctggcccgttgcccgccgatggccctcaaccaaaacacttttaaactgtggaattttagtctgaatcttgtttctaaaataaccaaaaactttcaacaacggatctcttggttctg
>OTU_2550_1
ttaccgagtttacaactcccaaacccctgtgaacatacctatgttgcttcggcggaccaccccaaccccctcggggcgaggggccgccagaggacccaaaacccaactgttttttccgtaacgaaacctattctgagtggaattattaaataaatcaaaactttcaacaacggatctcttggctctg
>OTU_2558_21
ttaaagagtaccggagctctcgggttcctactcccaccctatgttgactttaaatgttgctttggcggaccggcaatcttgccactggcctaggctggacagcgtccgccagaggatttttaaattcctttgactgaattctgagtctttaaaaattgaatcaaaactttcaacaacggatctcttggctctc
>OTU_2559_9
ttattgtggggacctcggtccttccaagatgcaacccttgcctttttcaagcgtctctctgtttcctcggcagctgcgcctgccagcgaggacccttaaaaaccttttgcaatccccgtacaaacttctgaaaataccaaaaaaacgttaaaactttcaacaatggatctcttggctctg
>OTU_2563_2
ttactgaattgtcaacatgaagttgttgctggccctcatgtgggggcatgtgcacgctctgtttacacatccgttcacacctgtgcactctctgtagttctgtggtttgggggctctgtcctcctaccgtggtcctgcatatttacacacactctgtaacaaagtctcatggaatgcatgacgcgtttaacgcaatacaatacaactttcagcaacggatctcttggctctc
>OTU_2574_19
ttatcgtacaaaatgtgtgaggcatgcgagggctgtagctgactcaaagtcgtgcacgccggagtgtgtcctctcacataacaatccatctcaccctttgtgcaccaccgcgtgggcaccctttgggatcagactgatctcggaggatgctcgcgttttcacacaaaccccccttttaaaagtgtagagtgacctcatttatgcgctaacccgcaatcaatacaactttcaacaacggatctcttggctctc
>OTU_2576_1
ttaaatggcgtgcgacccgcatgcggacgctcccgggctccggcctcgcggatggtcgtgtgcggttcctcgctctatacccttgcctatttgtaccctcttgttgtttcctcggcgggtcttgactcgccggcgggaattttataaaccttttgcagtagcatccaatcattctgatcaaaagtcttaaatcgttacaactttcaacaatggatctcttggttctg
>OTU_2578_23
ttgtcgaactgtcgacacgagttgttgctggtcctcaaacgggggggcatgtgcacactctgtttgcatatccactcacacctgtgcaccctccatagttctatagccctgggggccttaaacccccagctgtggtcctatgtatttacacatacactgtaataaagtcttatggaatgtatgccgcgtttaacgcaatacaatacaactttcagcaacggatctcttggctctc
>OTU_2582_35
ttattgaataaacttgaacgggctgctgctggcgtcccctggggcgcatgtgcacgcctgtcatctttatttctccaactgtgcactcattgtcgacctgaagggttcttcgaatcgattgttttcgagttgggggactgctgtgcttaaaaagagtcggctttcccttgtatttttcaggtcgatgtcattttcacaaacctatgaaacctgtttagaatgttgaatcaggtcctttttgtacctataaagttaaatatacaactttcagcaacggatctcttggctctc
>OTU_2584_2
ttaccgagttagggtcttccaggcccgacctccaaccctttgtttactataccatgttgctttggcgggcccgcctttcggggctgccgggggctttcatgcccatggtcagtgcccgccagtagccttattaaattcttccataattatgttgtctgagtataaccataaaatcgttaaaactttcaacaacggatctcttggttctg
>OTU_2586_15
ttaccgagtgagggccctctgggtccaacctcccacccgtgtttatcgtaccttgttgcttcggcgggcccgccacttgtggccgccggggggcacctgcccccgggcccgcgcccgccgaagacaccattgaacgctgtatgaagattgcagtctgagcttattagctaaattagttaaaactttcaacaacggatctcttggctctc
>OTU_2593_1
ttaccgagcgagggcctccgggtccgacctccaaccccatgttattcgaccctgttgcctcgggggcgacccggccttcgggcgtcggggcccccggtggaccactcaactctgcatcattgcgtccgagtcacatattgaatcaatcaaaactttcaacaacggatctcttggttctg
>OTU_2606_16
ttaatgagaacttgccctttggggtagatctcccaccctttgtgaaccattcctttgttgctttggcaggcccgtctccggaccgccggcttcggctggccagcgcctgccagaggattttcaaaaccttctgttagtgttgtctgagtataaatcaaatcgttaaaactttcaacaacggatctcttggttctg
>OTU_2609_6
ttaacgagttagggtcttctcggcccgacctcccaaccctttgtttactgaacctttgttgcttcggcggacccgtctcacgaccgccgggggaccgtaagacgtcctctggcccgtgtccgccggtggcccaactgaacaaattctgattaaaatgtgtcaatgtctgagtagaattcaattaaaacaaaactttcaacaacggatctcttggttctg
>OTU_2612_48
ttaccgaacgtcgacacgagttgttgctggtcctcgaatgggggcatgtgcacgctctgtttacacatccactcacacctgtgcaccccccgtagttctatggtctggagggcttgccgtcttcttcccgtagttctacgtctttacacatacaccgtaacaaagtctcatggaatgtatgcagcgtttaacgcaatacaatacaactttcagcaacggatctcttggctctc
>OTU_2613_1
ttaccgagttcatgccctcacgggtagatctcccaccctatgttatcattacctttgttgctttggcaggccgtcaggcttcggtccggctaccggctcaggctggtaagcgcctgccagaggaccccaaactctgaatattagtgttgtctgagtactataaaatagttaaaactttcaacaacggatctcttggttctg
>OTU_2616_5
ttaccgagttagggtcttctaagggcccgacctccttacctttgtctaccttacctcacgttgcttcggcgggcccgtcctcttttggaccgccggagggttcaccccctctggcccgttgcccgccgacagccccccaaccaaaaaactcttgcaataactgtggaattgtctgaatcttgcttctaaaataaccaaaaactttcaacaacggatctcttggttctg
>OTU_2618_11
ttaccgagtgagggccctctgggtccaacctcccacccgtgtttatcgtaccttgttgcttcggcgggcccgcctcacggccgccggggggcatccgcccccgggcccgcgcccgccgaagacacctgtgaacactgtctgaagttgcagtctgagaaactagctaaattagttaaaactttcaacaacggatctcttggttccg
>OTU_2630_1
ttatcgtagggcttctgccctgtcgagatagcgtccttgcctattttttggagtaccttttcgtttcctcagcaggcttgcctgctactggggaccctttaaactctttgtaaatacagcatctgtctgaataaacaaacaaaaaatcaaaactttcaacaatggatctcttggttctg
>OTU_2634_17
ttaacgagttagggtcttctcggcccgacctcccaaccctttgtttactgaacctttgttgcttcggcggacccgtctcacgaccgccgggggaccgtaagacgtcctctggcccgtgtccgccggtggccaaaccgaacaaattctgattaaaatgtgtcaatgtctgagtagaattcataattaaagcgaaaactttcaacaacggatctcttggttctg
>OTU_2645_1
ttacagagttttataactcccaaacccttgtgaacctacctgtacgtcgttgcttcggcgggtagccccccacggggcccagcctcctagcgaggtgcccgccggaggaccaccttaaaactcttgcaatttgtggcctctctgagtattatataaataagtcaaaactttcaacaacggatctcttggttctg
>OTU_2647_1
ttaacgagctagggtcttcttggcccgacctcccaaccctatgtttactgaacctttgttgcttcggcggacccgttcttacgaccgccgggggaccgtaagacgtcctctggcccgtgtccgccggtggcccaactgaacaaattctgattaaaatgtgtcaatgtctgagtagaattcataattaaaacaaaactttcaacaacggatctcttggttctg
>OTU_2651_4
ttatcgtaaaacagaggtgcgagggctgtcgctgactttctaagtcgtgcacgcccgagcgctctcacacaatccacgtcacccctatgtgcaccaccgcgtgggtcccccttgaaaggggggctcgcgttttcacacaaacttgaagtagtgtagaatgtctttttttgcgatgacacgcaatcaatacaactttcaacaacggatctcttggctctc
>OTU_2659_30
ttacagagttttcacaactcccaacccttgcgaaccgtacccattctgttctcgttgcttccggcgggggggacgggggggcccgaaacggcctccccgccccgcccgccgggggcggcgggccctacgaacctttatatctcaaccactagaaaccgtctgagaaacaaacaaaataatcaaaactttcaacaacggatctcttggctctg
>OTU_2664_41
ttattgaataaatctggcgtggttgtagctgactctcaggagtatgtgcacacccgtcacctttatctttccacctgtgcacacactgtagtcctggatacctctcgccgaaaggcggacgcggaggctgctgtgcccgcaagggccagcttcctctgaatttccaggtctatgattttttatacaccccaaacgaatgtcttagaatgtctttacaaggccttgtgcctataaaaacctatacaactttcagcaacggatctcttggctctc
>OTU_2667_17
ttattgactgtgaatcgttgcctccagtgctggctccggcaagtgcacgttggtgactttcatccaacaccctgtgaacctttggcctcttgctcgcttcggctggcagaggatttttacacccactcgaatgtaatgaaattattgtcgtgcgcaagcactaatgtacaactttcaacaacggatatctaggctctc
>OTU_2674_4
ttacagagttgcaaaactcccaaaccattgtgaacgttacccatcccgttgcttcggcgggcggcccgggccccgtgcccggcgccccccggcccctcgcgggcgcccgccggaggtaaaccaaactcttgaattgtatggcctctctgagtcttctgtactgaataagtcaaaactttcaacaacggatctcttggttctg
>OTU_2675_2
ttaccgagttcatgccctttgggtagacctcccaccctgtgtcgttatacctctgttgctttggcgggccgtggggccccggccctgcccctggctccggctagggcgcgcccgccagaggacctcaaaacctgaatgttagtgtcgtctgagtactatataatagttaaaactttcaacaacggatctcttggttctg
>OTU_2679_25
ttaccgagtttacaactcccaaacccaatgtgaaccataccaaactgttgcctcggcggggtcacgccccgggtgcgtcgcagccccggaaccaggcgcccgccggagggaccaaccaaactcttttctgtagtcccctcgcggacgttatttcttacagctctgagcaaaaattcaaaatgaatcaaaactttcaacaacggatatctaggctctc
>OTU_2683_4
ttaccgagttagggtagtcactcactgcccgacctcccaaccctgtgtctaccacactttgtcgttgcttcggcggaccggttgaccaactggtcatgaccgccgggggctggctctgcccccctggagagcgtccgccgatggcccaaccacaaaactcttgtaccgaaacgtgtcgtctgaatcatgtttgagaaatcaaaaacaaaactttcaacaacggatatctcggctctc
>OTU_2684_7
ttacagagttgcaaaactccctaaaccattgtgaacgttacctaaaccgttgcttcggcgggcggcgccctcgcgcgcccctgggccccaccgcgggcgcccgccggaggtcaccaaactcttgatatttattggcctctctgagtcttctgtactgaataagtcaaaactttcaacaacggatctcttggttctg
>OTU_2687_43
ttatcgtacaacgaggggggcccgagggctgtcgctgatcccttccgaggggtcgtgcacgcccttgacgtcgtcctcacgcaatcaatccatctcaccttttgtgcatcaccgcgtgggcctcgaaaggggcctgtgcctttttcacacacactcgtacgattcagtctagaatgtctttgcctttgtatgcaatcaatacaactttcaacaacggatctcttggctctc
>OTU_2689_20
ttattgtataaccgaggtgctagggctgtcgctgaccctttgaagggtcgtgcacgcccaagtgctctctcacatccatctcacccctttgtgcatcaccgcgtgggctacctttttggctttattcaaaaaggttggttcgcgtttttacacacacacacctttatgtatagaatgtcttaatttttgcggtcatacgcaataaataaataatacaactttcaacaacggatctcttggctctc
>OTU_2693_4
ttaaagagttagggtcttctaggcccgatctcccaaccctttgtttattgaacctctgttgcttcggcggacccgtctcacgaccgccggaggaccgctgaaaggcgtcctctggccagcgtccaccgatagccaaccacttaaactctgaataaatcgtgtcatatgtctaagtttatgattaaattaaagcaaaactttcaacaacggatctcttggttctg
>OTU_2696_1
ttaccgagtgcgggccctcgcggcccaacctcccacccttgtctctaatacacctgttgctttggcgggcccaccggggccacccggtcgccgggggacgcacgtccccgggcccgcgcccgccgaagcgctctgtgaaccctgatgaagatgggctgtctgagcacgatgaaaattgtcaaaactttcaacaatggatctcttggttccg
>OTU_2699_12
ttaatgattgcgaatcgtcgccttcagtgctggccggcttcggcaagtgcacgtcggtggctttcatccaataccctgtgcacctttggcctcttgctagcttcggccggcagaggatttttacacacactcgaatgtaatgaaaactattgtcgtgcgcaagcactaatgtacaactttcaacaacggatctcttggctctc
>OTU_2711_1
ttacagagttcatgcccttcggggtagatctcccacccttgtgtatcattatagaatgttgctttggcgggccgcgtgcctagcacgcctcgattcgcgtcgagcgtgcccgccagaggacccctaaactctgaatattaatgtcgtctgagtactattcaatagttaaaactttcaacaacggatctcttggctctc
>OTU_2716_23
ttacagaaagtaaacgcggatcaatccgcgaacttctaaacctttgacgattgactcgcgttgcctcggcgggttctcccgccagaggatacatcaaaactcctgttttaacggtgttgtctgagctacaagcaacgaatcaaaactttcaacaacggatctcttggctctc
>OTU_2720_13
ttacagagactctgccctttgggtagacctcccaccctgtgtcgttatacctttgttgctttggcgggccgcggggcttaggccctgcccctggctccggctagggcgcgcccgccagaggacctcaaaacctgaatgttagtgtcgtctgagtactatataatagttaaaaaactttcaacaacggatctcttggttctg
>OTU_2722_1
ttaccgaattgtcgacacgagttgttgctggtcctcgaaagggggcatgtgcacgctctgtttacacatccactcacacctgtgcaccctctgtagttctgtggcattggggactctgtcctcttgccgtggtcctacgtctttacacacacaccgtaatacagtctcatggaatgtatgtcgcgtttaacgcaatacaatacaactttcagcaacggatctcttggctctc
>OTU_2733_2
ttactagggggcttcggccccatcaagataccaccccttgcattttgagtaccttctgtttcctcggcgggcctgcccgccaacggggaccacaccaaacccttctgtagtcgcagtaaacgtctaaaaacataatttaaaactttcaacaacggatctcttggttctg
>OTU_2742_10
ttatagagttttctaaactcccaacccatgtgaacttaccattgttgcctcggcagaagctgctcggtgcaccctaccttggaacggcctaccctgtagcgccttaccctggaacggcttaccctgtagcggctgccggtggactaccaaactcttgttattttattgtaatctgagcgtcttattttaataagtcaaaactttcaacaacggatctcttggttctg
>OTU_2744_3
ttacagagttcatgccctcacgggtagatctcccacccttgcgtactataccatcgttgctttggcgggccgcctcggctaccggctcccgctggtaagtgcccgccagaggacccctaaactcttacgtatcagtgttgtccgagtacgatatcaatagttaaaactttcaacaacggatctcttggttctg
>OTU_2748_9
ttatcgtacaaaatgtgtgaggcatgcgagggctgtagctgactcaaagtcgtgcacgccggagtgtgtcctctcacataacaatccatctcaccctttgtgcaccaccgcgtgggcaccctccgatctcggagggggctcgcgttttcacacaaaaccccccccctttaaaaagtgtagaatgacctcatttatgcaatcaatacaactttcaacaacggatctcttggttctg
>OTU_2754_3
ttattgatagtttatattatcaaaaacccctttgtttatcatttaccatgttgcttccattggacagaccggcattgtgtatacaagttggtcctctagttttgtgtttatttggcactagctagggagctgccagtggatagaatccaaaaaaaaaaaattcgtgaagaaacatatattgtctgatttattagcacaaaaaaaataaaactttcaacaacggatctctaggctctt
>OTU_2757_4
ttattgactgtgaatcgttgcctccagtgctggctctggcaagtgcacattggtgactttcatccaacaccctgtgaacctttggcctcttgctcgctttggctggcagaggatttttacactcactcgaatgtaatgaaattattgtcatgcgcaagcactaatgtacaactttcaacaatggatctcttggctctt
>OTU_2766_2
ttaccgagttttcaactcccaaacccactgtgaacatatacctttgttttcgttgcctcggcgggtcgtgctccctggaaacaggggttcgcgccgccgggtgacacctaaaccctgatttaattacagaagtctttctgagtaaaacattctaaatgaatcaaaactttcaacaacggatctcttggttctg
>OTU_2770_1
ttattgattgcgaatcgttgccttctgtgctggccagctctggcaagtgcacgtcggtgactttcatccaataccctgtgaacctttggcctctgctagctttggccggcaggggaatttacacacactcgaatgtaatgaaaactattgtcgtgcgcaagcactaatgtacaactttcaacaacggatctcttggctctc
>OTU_2780_6
ttactgagtgcgggccctctgggtccaacctcccacccgtgtttatcgtaccttgttgcttcggcgggcctccgtgcccgccggagacacctttgaacgctgtctgaaggttgcagtctgagtcgattagctaaataagttaaaactttcaacaacggatctcttggttccg
>OTU_2787_1
ttacagagttgcaaaactcccaaaccattgtgaacgatacccgtaccgttgcttcggcgggccggctcctggagccgcagccccccctcggggggcggcccgccggacgacaccaaaactcttgtctcacacaggcctctctgagaacttatacaaatgagtcaaaactttcaacaacggatctcttggttctg
>OTU_2789_38
ttaaagagtaccggagctctcgggttcctactcccaccctatgttgactttaaatgttgctttggcggaccggcgatctcgccactggcctaggctggacagcgtccgccagaggatttttaaattcctttgactgaattctgagtctttaaaaattgaatcaaaactttcaacaacggatctcttggttctg
>OTU_2800_6
ttcataataagtgttttatggcactttttaaatccatatccaccttgtgtgcaatgtcagtcgatcttcttcatggagatcgaccaaacatcaaccttatcttttaactctttgtctgaaaaatattatgaataacttaattcaaaatacaactttcaacaacggatctcttggctctc
>OTU_2801_31
ttaccgagtgagggccctctgggtccaacctcccacccgtgtttatttaccttgttgcttcggcgggcccgcctcacggccgccggggggcacctgcccctgggcccgcgcccgccgaagacaccattgaactctgtctgaagtttgcagtctgagcgattagctaaatcagttaaaactttcaacaacggatctcttggttccg
>OTU_2802_2
ttaccgaattgtcaacaagagttgttgctggtcctcaaacaggggacatgtgcacgctctgttcacacatccacccacaccatgtgcaccctctgtagttctatggtcaagggggcctgttcctcttgctgtggttctgcttctttacacacacaccgtaacaaagtctcatggaatgcatgccgcgtttaacgcaatacaatacaactttcagcaacggatctcttggttctg
>OTU_2803_1
ttatggtacaacggaggcgcctgggctgtcgctgacctttaaaggacgtgcacgcccagagtcgctctctcacacatccatctcacccctttgtgcatcaccgcgtggggccctctcttttggcttgttccgggaggggggttcacgtttttacacgaacaacccattaatgcatgtgtagaatgtcttacttatttaaaatacaactttcaacaacggatctcttggctctc
>OTU_2810_5
ttaacgagctagggtcttccaggcccgacctcccaaccctatgtttattgaacctctgttgcttcggcgggcccgcctcacggccgccggaggagtcgccgcgaggcaccctctggccagcgtccgccgatagccaacctcaaaaaactctgaatgaatcgtgtcatgatgtctaagtctatgattaaattaaagcaaaactttcaacaacggatctcttggttctg
>OTU_2815_3
ttactgaactgtcaacacgagttgttgctggtcctcaatcgggggggcacgtgcacactctgtttacatatccactcacacctgtgcactcactgtagttccatggcatgggggatcctgtcctcctgctgtggttctatgtctttacacacacacactgtaacaaaagtctcatggaatgtataccgcgtttaacgcaatacaatataactttcagcaacggatctcttggctctc
>OTU_2816_2
ttaccgagttaacacctcccaaacccaatgtgaaccataccaaactgttgcctcggcggggtcacgccccgggtgcgtcgcagccccggaaccaggcgcccgccggagggaccaaccaaactctttctgtagtcccctcgcggacgttatttcttacagctctgagcaaaaattcaaaatgaatcaaaactttcaacaacggatctcttggttctg
>OTU_2818_12
ttactgagaatgctctccggagccctcaaaccttgcgtatttatcttgtacttgcgggtgcggtgtcgcaagacatcccccgcgaggattcaaactgtttgactgtcgtcagattcttacaaaagaaaacaactttcaacaatggatctcttggctccg
>OTU_2821_3
ttaccgagtttacaactcccaaacccaatgtgaacgttaccaaactgttgcctcggcgggatctctgccccgggtgcgtcgcagccccggaccaaggcgcccgccggaggaccaacctaaaactcttattgtataccccctcgcgggtttttttataatctgagccttctcggcgcctctcgtaggcgtttcgaaaatgaatcaaaactttcaacaacggatctcttggttctg
>OTU_2826_10
ttaaagagtaccggagctctcgggttcctactcccaccctatgatgactttaaatgttgctttggcggaccggcaatattgccaccggcctaggctggatagcgtccgccagaggatttttaaattcctttgactgaattctgagtctttaaaaattgaatcaaaactttcaacaacggatctcttggttctg
>OTU_2827_14
ttatcgagtgaagggttctctaagaacctgacctcccaacccttgtgttctttaccactttgctttggcgggcccgtcactgctgtgaccgccgaagggttatgtttttaactaacctttgggcccgtgcccgccagagatatctgaacgcttgttatgaaataggttgtctgagttataaagaaaaataagtcaaaactttcaacaacggatctcttggttccg
>OTU_2830_4
ttaccgagtgcgggccctcgcggcccaacctcccacccttgtctctatacacctgttgctttggcgggcccaccggggccacctggtcgccgggggacgctcgtccccgggcccgcgcccgccgaagcgctctgtgaaccctgatgaagatgggctgtctgagtagtatgaaaattgtcaaaactttcaacaatggatctcttggttccg
>OTU_2842_42
ttaacgaataaacttgatcaggctgttgctggcccccttgcaggggtatgtgcacgcttgtcatatttatcatttctccaactgtgcacatattgtagacctggatcttttttgaagaaattcaagttgggggactgctgtgctctttgtttttttttcaagagtcggctttccctcgtatttttcaggtctatgtcattttcacaacctctagaatgtgtttagaatgttgaatcatatataaagttaatatacaactttcagcaacggatatctaggctctc
>OTU_2844_23
ttatcagagctttgctcatcctattcttgtgaaaattccagtctatacattaaaactttcaacaacggatctcttggctccg
>OTU_2846_3
ttatcgtacaaaatgtgaggggcatgcaagggctgtcgctgactgaatgtcgtgcacgcccgggtgtgtttcctcacataataatccatctcaccctttgtgcatcaccgcgtgggcaccctttgggatcatctcggagggggctcgcgttttcacacaaacaccccttttaaaaagtgtagaatgacctcatttatgcaatcaatacaactttcaacaacggatctcttggctctc
>OTU_2849_1
ttaccgagctcatgcccttccgggtagatctcccaccctatgttattattacctttgttgctttggcgggccgccaggctccggtcaggctatcggcttcggctggtaagcgcccgccagaggacccaatatcctgattattagtgtcgtctgagtactatataatagttaaaactttcaacaacggatctcttggttctg
>OTU_2854_1
ttaacgagttagggttccttttgggcccgacctcccaaccctttgtctacttgaccatcgttgcttcggcgagcccgtcctcacggaccgccggagggatctttactggccctctggtccgcgctcgtcggtagcccaacctttaaaatctttaactaaacgtgccttaatctaagtacaattattaaataaaagcaaaactttcaacaacggatctcttggttctg
>OTU_2864_1
ttatcgagtgtaaaaactcaccaaaccctgtgaacataccttctgttgcttcggcgggatcgccccgggcgccctcgtgtgccccggatccaggcgcccgccggaggacctaaactcttgtctttatgagatttatctgagtggctttatagcaaaatgaatcaaaactttcaacaacggatctcttggttctg
>OTU_2866_19
ttacatggcggaggaggtgggcacctggtaaccctaggtgtgttgccttcacgcttactctacccttgtctatttgcaccttttgtcgttccctcggcgggtcttgtactcgccggcggcaactttcaaaacctcttgcagttagtatctaattcttctgattcaataacaaatttattacaactttcaacaatggatctcttggttctg
>OTU_2867_1
ttactgatttgcttaattgcaccacatgtgttttttactggacagctgctttggcggtggggactcgtttccgccgccagaggtcacaactaaaccaaactttttattaccagtcaaccatacgttttaatagtcaaaactttcaacaacggatctcttggttctc
>OTU_2879_2
ttatcgagtttacaactcccaaaccctatgtgaacatacctacagttgcttcggcggactcgccccggcgtccggacggcctagcgccgcccgcggcccggatccaggcggccgccggagaccaccaaactctttgtattatcagtgtattctgaatccgccgcaaggcaaaacaaatgaatcaaaactttcaacaacggatctcttggttctg
>OTU_2884_5
ttactgagtgagggcccctcggggtccaacctcccacccgtgtttaacgtaccttgttgcttcggcgggcccgcctcacggccgccggggggcatccgcccccgggcccgcgcccgccgaagccacctgtgaacgctgtctgaagtatgcagtctgagacaattattaaattaattaaaactttcaacaacggatctcttggttccg
>OTU_2897_20
ttaatgattgcgaatcgtcgccgtcagtgctggccggcttcggcaagtgcacgtcggtgactttcatccaataccctgtgcacctttggcctcttgctagcttcggccggcagaggatttttacacacactcgaatgtaatgaaaactattgtcgtgcgcaagcactaatgtacaactttcaacaacggatctcttggctctc
>OTU_2910_1
ttacagagaacttgccctttggggtagatctcccaccctctgtctaccatactttgttgctttggcaggcccgcccttgggccgccggcttcggctggccagcgcctgccagaggaccacaaactcttgtttttagtgtcgtctgagtacttgataatagttaaaactttcaacaacggatctcttggttctg
>OTU_2911_1
ttacagagttcatgcccttacagggtagatctcccacccttgaatactatacctttgttgctttggcgggccgcttcggctactggctccggctggtgagtgcccgccaaaggatctcaaaactctgaatatttgtgtcgtctgagtactatataatagttaaaactttcaacaacggatctcttggttctg
>OTU_2912_22
ttaccagagatggcgggcccccgcgcccccgtccccaaccctttgttgacaaacctttttgcctcgggggcagagcgtccgccgccgcccccggaggaccaccaaacgctgtctctgtacgtcggagtctttgatgaaatgaatgaaaactttcaacaacggatctcttggttctg
>OTU_2919_2
ttaccaaaacttagctgtctggccgcaaggctctgacgcttcaaccctttgtgaaccaaaaaccctttcgcttcggcagccgcgccggttgggaacagcctgcgcttcagcctgccgttagcaccaaacatcaaaacttgcagtcaaaaacattgtctgataccaaattttcgaatgaaaatcaaaactttcaacaacggatctcttggttccc
>OTU_2924_3
ttaccgagtttacaactcccaaacccctgtgaacataccttattgttgcctcggcggatcagcccgctccccgtaacacgggacggcccgccagaggacccaaactctattgtatttagtgtatcttctgagtaacacaaacaaataaatcaaaactttcaacaacggatctcttggttctg
>OTU_2929_1
ttactgagtttttaactctccaaaccatgtgaacttaccactgttgcctcggtggtcggtgctggaaacagtgctgccaccggtggactactaaactcttgttaatttttgtcaaatctgaatcaaaactaagaaataagttaaaactttcaacaacggatctcttggttctg
>OTU_2931_1
ttaccgagttagggtctacctctaggcccgacctccttacctctgtttacctgacctcatgttgcttcggcgggcccgtccccttaaccaggcgaccgccggaagggctcaccccttctggcccgttgcccgccgatggccctcaaccaaaacactcttaaactgtggaatttaagtctgaatcttgtttctaaaataaccaaaaactttcaacaacggatctcttggttctg
>OTU_2934_38
ttaccgaattgtcaacaagagttgttgctggtcctcgaatgggggcatgtgcacgctctgttcacacatccactcacaccctgtgcaccctctgtagttctatggtcagggggcctgtcctcctgctgtggttctgcatctttacacacacacacacagtaacaaagtctcatggaatgcatgccgcgtttaacgcaatataatacaactttcagcaacggatctcttggctctc
>OTU_2935_5
ttaccgagaaactgccctttgggtagatctcccaccctttgtttacattacctttgttgctttggcaggcccgtctttggaccgccggcttaggctggtctgtgcctgccagaggatcctaaaactcttgatttttgtattgtctgagtactatataatagttaaaactttcaacaacggatctcttggttctg
>OTU_2940_2
ttaccacatatcatcgggggggaaacttcggtttcccctgtctgaacccttgtcttttgcgtactatttgtttcctcggtgggcttgcctgccgataggacaaccattaaaccttttgtaattgcaatcagcgtcagaaaaacataatagttacaactttcaacaacggatctcttggttctg
>OTU_2963_2
ttaccgagttagggtcttccaggcccgacctccaaccctttgttaactataccatgttgctttggtgggcccgcctttaggggccgccggggactttcatgcccctggtcagtgcctgccagtagccttctaaaattcttataattatgtcgtctgagtatagatataaatcgttaaaactttcaacaacggatctcttggttctg
>OTU_2967_7
ttactgagaatgctctccggagccctcaaatcttgcgtacccatcttgtacttgcgggtggccccccgggcgcccgcgaggattcaaactgtatgactttgtctgattcttacaaagaaaacaactttcaacaatggatctcttggctccg
>OTU_2973_40
ttattgaataaacttggttgggttgctgctggctcttaggagcatgtgcacgctcactccattttaaccacctgtgcacatactgtagacctgaatgaaacttttcgaggtaactcggtttgaggactgttgtgaaaatcagctgttcttgcattccaggtctatgtttttaaaaatatactccataacaagttatagaatgttaataatgggccttctgtgcctttaaattataatacaactttcaacaacggatctcttggttctg
>OTU_2983_4
ttatcgtacaatggaggtgcgagggctgtcgctgacctttttttggtcgtgcacgcccgagcgctctcacacaatccatctcaccccttgtgcaccaccgcgtgggttccctttctggcttgtccgaaggggggctcgcgttttcacacaaacttgaattagtgtagaatgtccttttttgcgataacacgcaattaatacaactttcaacaacggatctcttggctctc
>OTU_2990_13
ttaccgagttagggtcttctcaggcccgacctccttacctttgtctaccttacctcacgttgcttcggcgggcccgtcctcttttggaccgccggagggttcaccccctctggcccgttgcccgccgacagccccccaaccaaaaaactcttgcaataactgtgaaattgtctgaattttgcttctaaaataaccaaaaactttcaacaacggatatctaggctctc
>OTU_2994_16
ttattgagtgaagggttctctaggaacctgacctcccaacccttgtgttctttaccactttgctttggcgcgcccgtcactgtgaccgccgaagggttatgtttttaactaacctttgggcccgtgcccgccagagatatctgaactcttgatatgaaataggttgtctgagttataaagaaaaataaatcaaaactttcaacaacggatctcttggttccg
>OTU_2996_4
ttaccgaattgtcaacaagagttgttgctggtcctcaaatgggggcatgtgcacgctctgttcacacatccactcacaccctgtgcaccctctgtagttctatggtcagggggcctgtcctcctgctgtggttctgcatctttacacacacacactgtaacaaagtctcatggaatgcatgccgcgtttaacgcaatataatacaactttcagcaacggatctcttggttctg
>OTU_2997_1
ttaccgagtcacgtgctcaaacaacccttctgtggacctaaattaaaccgttgcctcggcgggcggtccttctcaaccccccatgtgggggttgagaagggccagacagtccggcacgcctaagggcgtcccccggcgccagccgctggccccaaaaaattaaactctagtaaccttaccttctgagttataaatacaaaaaataagtcaaaactttcaacaacggatctcttggctctg
>OTU_3003_2
ttaacgagttagggtctcctcggcccgacctcccaaccctatgtttactgaacctttgttgcttcggcggacccgttcttacgaccgccgggggaccgtaagacgtcctctggtccgtgtccgccggtggccaaactgaacaaattctgattaaaactgtgtcaatgtctgagtacaattcataattagaacaaaactttcaacaacggatctcttggttctg
>OTU_3007_3
ttattgaatgaatatagagttggttgtcgctggctcctccgggagcatgtgcacgctttctctttcatccacacacacctgtgcacttgtgagacggaggaccgtaaaaaagtcttccgtctattaaaccacacaaaccccattgtatttaaattgaatgtaattgatgtaacgcatcattagaactaagtttcaacaacggatctcttggctctc
>OTU_3010_35
ttaccgaattgtcaacacgagttgttgctggtcctcaaacggggacatgtgcacgctctgtttacacatccactcacacctgtgcaccctccatagttctgcagcctgggggctctgtccccctgatgcggttctatgtatttacacacacacactgtagtaaagtctcatggaatgcataccgcgtttaacgcaatgaaatacaactttcagcaacggatctcttggctctc
>OTU_3015_1
ttaccgagttcatgcccttacgggtagacctcccaccctatgtgatattacctttgttgctttggcaggccgtcaggcttcggtccggctaccggctcaggctggtaagcgcctgccagaggaccccaaactctgaatattagtgttgtctgagtactatctaatagttaaaactttcaacaacggatctcttggttctg
>OTU_3020_4
ttactgaattgtcaacatgaagttgttgctggccctcatagggggcatgtgcacgctctgtttacacatccactcacacctgtgcaccctctgtagttctatggtttgggggaccctgtcttccttctgtggttctacgtctttacacacacactgtaataaagttttatggaatgtacatcgcgtctaacgcaatacaatacaactttcagcaacggatctcttggctctc
>OTU_3022_37
ttgtcgaattgtcaacacgagctgttgctggtcctcaaacgggggcatgtgcacgctctgtttacacatccacttacacctgtgcacccttcatagttctgtggcctgggggcaccgtcccccctgctgtggtcctatggatttaacacacacacactgtaaccaagtctcatggaatgtacaccgcgtttaacgcaatacaatacaactttcagcaacggatctcttggctctc
>OTU_3027_1
ttagcgagttaattacaaactccaaaacccctgtgaacttacctatgttgccttggcaggtcgtggtgtgtagcgtgatcgctataagggatcaccgcctcgcaccacgctgaaagacctgtcaaaggacccctaaactctgtcttacaactgtatctctgagtctattatataaacaagttaaaactttcaacaacggatctcttggctctg
>OTU_3030_9
ttaccgagttgtaacactcaccaacccactgcaaacgtacccattttggcgactccggcaggcggccccagggcggggcctcagcctttttaggcgcctgccgaaggtgccgcaactttactctgtaattatgtattctgagtcctaagacaaaatatcaaaattttcaattgtggatctcttggttctg
>OTU_3033_20
ttatcgtacaaaatgtgtgaggcatgcgagggctgtagctgactcaaagtcgtgcacgccggagtgtgtcctctcacataacaatccatctctcaccctttgtgcaccaccgcgtgggcaccctccgatctcggagggggctcgcgttttcacacaaaaccccccctttaaaaagtgtagaatgacctcatttatgcgctaacccgcaatcaatacaactttcaacaacggatctcttggctctc
>OTU_3035_37
ttaatgagttgtgatggggtttgatgctggcagccaaatttttttggatgcatgtgcttgccctgacaatcatatcccaaacacctgtgcacatatttgagggaattaagttgatttgccgccttttggtggtcgtggtcgtcttgcattcccttaaattactatacgctgtcaataatgttgaacgtgcttgtgccgcaaggccattaatataatataacttttaacaacggatctcttggctctc
>OTU_3052_33
ttactgaactgtcgacacgagttgttgctggtcctcgaaaggggtcacgtgcacgctctgtttacacatccactcacacctgtgcacccttgtagttctatggcctggggacttggtcctcttgctgtggtcctacgtctttacacacacacaccgtaacaaagtatcatggaatgtatgtcgcgtctaacgcaatacaatacaactttcagcaacggatctcttggctctc
>OTU_3063_4
ttacaaatatctgggttgtcctatcgggctcccatgcaaaacacattcatgtgtattcttccatattttaaacttttgaatcaatttagttgtctgagaaggccatgtgccataaaatttaaatacttcaaaactttcaacaacggatatctaggctctc
>OTU_3066_3
ttacagagaaaattgccctttggggtagatctcccaccctttgtttacattacctttgttgctttggcaggcccgtctttggaccaccggcttaggctggtctgcgcctgccaaaggaccctaaactcttgatttttgtgttgtctgagtactatataatagttaaaactttcaacaacggatctcttggttctg
>OTU_3068_34
ttaatgattgcgaatcgtcgccttcagtgctggccggcttcggcaagtgcacgtcggtggctttcatccaataccctgtgcacctttggcctcttgctagcttcggccggcagaggatttttatacacacactcgaatgtaatgaaaactattgtcgtgcgcaagcactaatgtacaactttcaacaacggatctcttggctctc
>OTU_3074_12
ttattgtataaccgaggtgctagggctgtcgctgaccctttgaagggtcgtgcacgcccaagtgctctctcacatccatctcacccctttgtgcatcaccgcgtgggctacctttttggctttattcaaaaaggttggttcgcgtttttacacacacacctttatgtatagaatgtcttaatttttgcggtcatacgcaataaataatacaactttcaacaacggatctcttggctctc
>OTU_3077_1
ttagcgaaactcgaaaggtgcggaggggggacctttgccggtccttcgaagccctttttactttgtccacacctctgtgcaccccgttcgcgcgaggttcttcggaaccctgtgtgatgcttatctcgaactcgtatgtctacagaatgtgacctagtgtgttggaaatggaaatacaactttcagcaacggatctcttggctctc
>OTU_3085_6
ttaaagagttagggtcttctaggcccgatctcccaaccctttgtttattgaacctctgttgcttcggcggacccgtctcacggccgctggaggaccgctgaaaggcgtcctctggccagcatccgccgatagccaaccacttaaactctgaataaatcgtgtcatatgtctaagtctatgattaaattaaagcaaaactttcaacaacggatctcttggttctg
>OTU_3093_17
ttacagaaagtaaacgcggatcaatccgcgaacttctaaacctttgacgattgactcatgttgcctcggcgggctcgcccgccagaggatacatcaaaactcctgttttaacggcgttgtctgagctacaagcaacgaatcaaaactttcaacaacggatctcttggctctc
>OTU_3103_5
ttattgtaacaatggaggtgcaagggctgtcgctgaccttccacggtcgtgcacgcccgagcgctctcaccacaatccatctcacccctttgtgcatcaccgcgtgggtccccctttgcaggagggctcgcgttttcacataaaacttgacacagtctagaatgatcttttttgcggtaacacgcaatcaatacaactttcaacaacggatctcttggctctc
>OTU_3115_22
ttaacgagttagggtcttctcggcccgacctcccaaccctttgtttactgaacctttgttgcttcggcggacccgtctcacgaccgccgggggaccgtaagacgtcctctggcccgtgtccgccggtggccaaaccgaacaaattctgattaaaatgtgtcaatgtctgagtagaattcataattaaaacaaaactttcaacaacggatatctaggctctc
>OTU_3118_2
ttactgagtgcgggccctctgggtccaacctcccacccgtgtttatcgtaccttgttgcttcggcgggcccgccgccaggccgccggggggcagcagcccccgggcccgcgcccgccgaagacacctgtgaacgctgtatgaagattgcagtctgagcgattagctaaatcggttaaaactttcaacaacggatctcttggttccg
>OTU_3121_2
ttacagagttcgtgccctcacgggtagatctcccacccttgtgtattctctacatcttgttgctttggcaggccgttggttcgccaaccaccggctcgcgggctggtgcgtgcctgccagaggacctctcaaactcggtttgtcagtgtcgtctgagtaccataacaatcgttaaaactttcaacaacggatctcttggttctg
>OTU_3123_15
ttaacgagttagggtctctcacggcccgacctcccaaccctttgtttatcgaacctctgttgcttcggcggatccgtccctcgggaccgccggaggatcgtcgtaaggcgtcctctggcccgtatccgtcgatagccaaccacttaaactcagaataaatcgtgttataattgtctaagtttatcataattaaaacaaaactttcaacaacggatctcttggttctg
>OTU_3127_6
ttactgagtgagggcctccggcccgacctccaaccctgtgtcgattccaacctctgttgcctcgggggtcacctggtcctttgctgtccagggcccccggtggaccactccaactctgcgtatgagcgtccgagtccatcgttaaatgaatgaaaactttcaacaacggatctcttggttctg
>OTU_3128_3
ttaccgaatcgtcaaacacgggttgttgctggcctccaaacgggggcacgtgcacgctctgtttacgcatccactcacacctgtgcaccctctgtagtcctatggttcggaagaccccgtcttccttctgtagctctacgtctttacacacacactgtagcgatgtctcatggaatgtttttatgcgtttaacgcgatacaatacaactttcagcaacggatctcttggttctg
>OTU_3132_2
ttaaagagttagggtcttctaggcccgacctcccaaccctatgtttattgaacctctgttgcttcggcggacccgcctcacggccgccggaggaccgctgaaaagcgtcctctggccagcgtctgtcgacagccaaccacttaaactctgaatgaatcgtgtcatatgtctaagtctatgattcaaatcaaataaaagcaaaactttcaacaacggatctcttggttctg
>OTU_3133_7
ttaccgagttagggtctcctaggcccgatctcccaaccctttgtttaccgaacctctgttgcttcggcggacccgcctcgcggccgccggaggactgcccctaagccgtcctctggccagcgtccgccgacagccaacccttctcaactctgaataaatcgtgttgtaaatgtctaagtctatgattcaaatcaataaagcaaaactttcaacaacggatctcttggttctg
>OTU_3139_29
ttactgaactgtcgacacgagttgttgctggcccccgaaagggggcatgtgcacgctctgtttacacatccactcacacctgtgcaccctctgtagttctgtggccttggggactcccgccctctcgctgcggtcctacgtctttacacacacacaccgtaatacagtctcatggaatgtatgccgcgtttaacgcaatacaattacaactttcagcaacggatctcttggctctc
>OTU_3140_36
ttactgaatcgtcaacatgaagttgttgctggccctcatgtgggggcatgtgcacgctctgtttacacatccattcacacctgtgcactctctgtagttctgtggtttgggggctctgtcctcctaccgtggtcctgcatatttacacatacacactgtgataaagtctcatggaatgtatgtcgcgtttaacgcaatgaaatacaactttcagcaacggatctcttggctctc
>OTU_3141_26
ttaaagagtaccggagctctcgggttcctactcccaccctatgttgactttaaatgttgctttggcggaccggctgtactgccaccggcctaggctggatagcgtccgccagaggatctttaaattcctttgaatgtgaattctgagtctttgaaaattgaatcaaaactttcaacaacggatctcttggttctg
>OTU_3144_6
ttatcgtacaaaatgtgaggggcatgcaagggctgtcgctgactgaatgtcgtgcacgcccgggtgtgtttcctcacataataatccatctcaccctttgtgcatcaccgcgtgggcaccctttgggatcatctcggagggggctcgcgttttcacacaaacaccccttttaaaaagtgtagaatgacctcatttatgcgctaacccgcaatcaatacaactttcaacaacggatctcttggttctg
>OTU_3149_9
ttaccgagttagggtttcgtccaacgagcccgacctcccaaccctttgtctactacaccttgtcgttgcttcggcggaccggtcgcgaccaactggtcgtgaccgccggggacccgtccctggagagcgtccgccgacggcccaaccaccaaactcttgtatcaaaccatgtcgtctgaattacttgattaaaatcaaaaacaaaactttcaacaacggatctcttggttctg
>OTU_3153_37
ttaatgagttgtgatggggtttgatgctggcagccaaatttttttttggatgcatgtgcttgccctgacaatcatatcccaaacacctgtgcacatatttgagggaattaagttgatttgccgccttttggtggtcgtcttgcattcccttaaattactatacgctgtcaataatgttgaacgtgcttgtgccgcaaggccattaatataatataacttttaacaacggatctcttggctctc
>OTU_3155_15
ttaacgagttagggtctctcacggcccgacctcccaaccctatgtttactgaacctttgttgcttcggcggacccgttcttacgaccgccgggggaccgtaagacgtcctctggcccgtgtccgccggtggcccaactgaacaaattctgattaaaatgtgtcaatgtctgagtagaattcataattaaaacaaaactttcaacaacggatctcttggttctg
>OTU_3159_16
ttacagagaaattgcccttcagggtaatcttccatcctatgtttacattacctttgttgctttggcaggcccgtcttttgaccactggcttaggctggtcagtgtctgccagaggcccccaaactcttgatattttgttgtctgagtacactataataagttaaaactttcaacaacggatctcttggttctg
>OTU_3162_34
ttaccgagtaccggagccctcgggttcctactcccaccctatgttgactttgaatgttgctttagcggaccggtggtccccaccgctggcctaggctggagagcgtccgctagaggatttttaaactctgtttaattggtgaattctgagtcttgataaattgaatcaaaactttcaacaacggatctcttggttctg
>OTU_3174_36
ttaccgagttcatgcccttacgggtagatctcccaccctgtgttatcattacctttgctttggcgggccgccaggctccggtcaggctatcggcttcggctggtacgcgcccgccagaggaccctaacattctgattatcagtgtcgtctgagtactatataataatagttaaaactttcaacaacggatctcttggttctg
>OTU_3181_6
ttacagagaaattgcccttttggggtagatctcccacccgtgtttacagtacctttgttgctttggcaggcccgtcttatgaccactggcttaggctagtccgcgcctgccagaggatcctaaactctttgatttttgtgttgtctgagttataaacaaaattaagtcaaaactttcaacaacggatctcttggttctg
>OTU_3186_34
ttactgaattgtaaacaagggttgtcgctggccctcatgtgggggcatgtgcacgctctgtttacacatccattcacacctgtgcactctctgtagttctgtggtttgggggctctgtcctcctaccgtggtcctgcatatttacacatacacactgtgataaagtctcatggaatgtatgccgcgtttaacgcaatacaatataactttcagcaacggatctcttggctctc
>OTU_3191_12
ttacagagttcatgccctccgggtagatctcccacccattgctatcactactctcgttgctttggcgggccgctgggccctgcccggccgccggccccggctggcgcgcgcccgccagagacctcacagactctgaatgttagtgtcgtccgagtaactatataataatagttaaaactttcaacaacggatctcttggttctg
>OTU_3193_10
ttaccgagttagggtcttctaagggcccgacctccttacctttgtctaccttacctcacgttgcttcggcgggcccgtcctcttttggaccgccggagggttcactctcctctggcccgttgcccgccgacagcccccaaccaaaaaactcttgcaataactgtgaaattgtctgaattttgcttctaaaaataaccaaaaactttcaacaacggatctcttggttctg
>OTU_3201_30
ttatcgtacaatggaggtgctggggctgtcgctgacctttaaaaagggtcgtgcacgcccggagcactctctcatatccatctcacccctttgtgcattgccgcgtgggtcccctgaaacaaagggggacctgcgtttttacatagacaccctttgaatgcatgtgtagaacgtcttacgatatcaatacaactttcaacaacggatctcttggctctc
>OTU_3211_23
ttaacgagttagggtcttccaggcccgacctcccaaccctatgtttattgaacctctgttgcttcggcggacccgtctcacggccgctggaggaccgctgaaaggcgtcctctagtcagcgtccgccgatagccaaccacttaaactctgaataaatcgtgtcatatgtctaagtctatgattaaattaaagcaaaactttcaacaacggatctcttggttctg
>OTU_3213_15
ttacagagattgacatacctagtgtgtcacctccaaccccttgtacaatcaaccataaagttgctttggtgtttcatcgccagaggctccatataatctttttaatcaatgctgtctgagtaaaatataaaatcgttaaaactttcaacaacggatctcttggctctc
>OTU_3223_35
ttattgaaattaaaatttggctgcgttgttgctggctcttaggagcatgtgcacatgcaccatttttttttttaaaaaaaaaccacctgtgcacacactgtagatctggattgaactttttcgaggattattaactcggttttgagaactgctttggctgttcttgtgttctccaggtctatgttttaaacaatatactccaacaagtaatagaatgtcaaattatgggccttttgtgcctttaaaatctaatacaactttcaacaacggatctcttggctctc
>OTU_3227_1
ttaacgagttagggtcttctaggcccgacctcccaaccctatgtttattgaacctctgttgcttcggcgggcccgtctcacgaccgccggaggagtcgccgcgaggcaccctctggccagcgtccgccgatagccaacctcaaaactctgaatgaatcgtgtcatgatgtctgagtctatgattaaattaaagcaaaactttcaacaacggatctcttggttctg
>OTU_3228_2
ttcataatcaaagtgtttttatggcacttttaaaaaaatccatatccaccttgtgtgcaatgtcatctcactggaggccagctggctgtcaaaagcccgtttggtcacctttgggatttatatctactcagaactttagtgattttgtctgaaaaatattatgaataaacaattcaaaatacaactttcaacaacggatctcttggctctc
>OTU_3237_3
ttaccaaagatggcgggcctccgcgcccccgtctccaacccattgttgaccaaaacccttttgcctcgggggcagagcgtccgccgccgcccccggaggaccattcaacgctgtctctttgcgtcggagtattgattaaatgaatgaaaactttcaacaacggatctcttggttctg
>OTU_3247_1
ttaccgagttcttacagaacccaacccttgtgtgataagctctcacccagcttttacacgggagtctcggcctaatagccgaggcagtgtaaaaaaaacaaaacctctatatcattatcacaaatttctgaagactttttaaaagagtcaaaactttcaacaacggatctcttggttctg
>OTU_3248_1
ttacagagttctcgccctcgcgggtagatctcccacccactgttattgctaccgtgttgctttggcgggctgctgggcctagcccggccgccggctccggctggcgcgcccgccagaggcccgacaaattctgattgtcagtgtcgtctgagtactatataatagttaaaactttcaacaacggatctcttggttctg
>OTU_3258_33
ttactgaattgtaaacaagggttgttgctggtcctcaaacggggacatgtgcacgctctgttcacacatccaatcacacctgtgcaccctctgcggttctgtggtcaggggggctttgccttcctgtgctgtggttctgcttctttacacacacactgtaacaaagtcttgtggaatgtatgctgcgtttaacgcaatacaatacaactttcagcaacggatctcttggctctc
>OTU_3264_7
ttatcgtacaacggaggtgcgagggctgtcgctgaccttcaaaggtcgtgcacgcccgagccctctcacaatccatctcaccctttgtgcatcaccgcgtgggtccccctttagcggggagggctcacgtttttacacaaaactcgatgcagtgtagaatgtttatttttgcggtcacacgcaatcaatacaactttcaacaacggatctcttggctctc
>OTU_3265_1
ttacacaatatgaaagcgggctggcatccttcggggttacagccttgctgaattattcacccgtgtcttttgcgtacttcttgtttccttggtgggttcgcccaccataggacaaaccataaaccttttgtaattgcaatcagcgtcagtaaaaaaattaataattacaacttttaacaacggatctcttggttctg
>OTU_3266_19
ttacagaaagtaaacgcggatcgaaccgcgaacttctaaacctttgacgattgactcatgttgcctcggcgggttctctcgccagaggatacatcaaaactcctgttttaacggtgttgtctgagctacaagcaacgaatcaaaactttcaacaacggatctcttggctctc
>OTU_3268_6
ttacagagaatatcgccctcgcgggtgactctccaacccaatgttattctacctttgttgctttggcgagccgccttggccaccggctcacgctggtgagcgcttgccagaggacccaaaactctgaattaaagtattgtctgagtactatataatagttaaaactttcaacaacggatctcttggttctg
>OTU_3278_4
ttaccgagtttacaactcccaaacccctgtgaacatacctatacgttgcctcggcggatcagcccgcgccccgtaaaaagggacggcccgcccgaggacccctaaactctgtttttagtggaacttctgagtaaaacaaacaaataaatcaaaactttcaacaacggatctcttggttctg
>OTU_3281_1
ttatcgagttagggtcttctaggcccgacctcccaaccctttgtttactgaaccactgttgcttcggcgggcccgtctcacgaccgccggaggagcgctgaaaagcgtgtcctctggcccgcgtccgccgatggccaaccacaaaactctgtgtaaatcgtgtctttatgtctaagtctatgattaaattaaagcaaaactttcaacaacggatctcttggttctg
>OTU_3284_1
ttacctagagtttgtggacttcggtctgctacctcttacccatgtcttttgagtaccttcgtttcctcggcgggtccgcccgccggttggacaacattcaaaccctttgcagttgcaatcagcgtctgaaaaaacttaatagttacaactttcaacaacggatctcttggttctg
>OTU_3287_24
ttattgaaataaacctgatgggttgttgctggttctctagggagcatgtgcacaccttgtcatctttatatctccacctgtgcaccttttgtagacctttcaggtctatgttgcttcatttaccccaatgtatgttaatagaatgttgtgcctatataatatatacaactttcagcaacggatctcttggttctg
>OTU_3296_5
ttaaagagttagggtcctctgggcccgacctcccaaccctttgtttactgaacctctgttgcttcggcggacccgtctcacgaccgccggaggatcgcccgtccaggcgtcctctggccagcgtccgccgatagccaacccttcaaactcctgaataaatcatgttatatgtctaagtcttatgattcaaatcaaataaaagcaaaactttcaacaacggatctcttggttctg
>OTU_3299_11
ttaccgagtgagggccctctgggtccaacctcccacccgtgtttaacgaaccttgttgcttcggcgggcccgcctcacggccgccggggggcatccgcccccgggcccgcgcccgccgaagacacctgtgaacactgtctgaagttgcagtctgagaaactagctaaattagttaaaactttcaacaacggatctcttggctctc
>OTU_3301_8
ttaacgagttagggtcttctcggcccgacctcccaaccctttgtttactgaacctttgttgcttcggcggacccgtctcacgaccgccgggggaccgtaagacgtcctctggcccgtgtccgccggtggcccaactgaacaaattctgattaaaattgtcaatgtctgagtagtattcataattaaaacaaaactttcaacaacggatctcttggttctg
>OTU_3319_32
ttattgaaataaacctgatgggttgttgctggctctctagggagcatgtgcacaccttgtcatctttatatctccacctgtgcactttttgtagacctttcaggtctatgttgcttcatttaccccaatgtatgttaatagaatgttgtgccttataaacctatacaactttcagcaacggatctcttggctctc
>OTU_3322_3
ttactgattgcgaatcgttgccttcagtgctggccggcttcggcaagtgcacgttggtgactttcatccaataccctgtgaacctttggcctcttgctagcttcggccggcagaggatttttacacacactcgaatgtaatgaaaactactgtcgtgcgcaagcactaatgtacaactttcaacaacggatctcttggctctc
>OTU_3324_8
ttacagaaagtaaacgcgggtcaaaccgtgaacttttaaacctttgacgattgactcatgttgcctcggcgggttctctcgccagaggatacatcaaaactcctgttttaacggtgttgtctgagctacaagcaacgaatcaaaactttcaacaacggatctcttggctctc
>OTU_3328_1
ttaacgttggggactaacaatccctcagcgagatagaacccttgctttttcgagtaccacacgtttcctcggcaggtacgcctgccaatggggaccattaaaaaccttttgtaatagcagtaaacgtctaaaacaacaaaaatttaaatcaaaactttcaacaacggatctcttggttctg
>OTU_3333_4
ttattgagtgaagggttctctaagaacctgacctcccaacccttgtgttctttaccactttgctttggcgggcccgtcactgctgtgaccgccgaaaggttatgcttttaactaacctttgggcccgtgcccgccagagatatctgaactcttgatatgaaataggttgtctgagtcatacagaaaaataaatcaaaactttcaacaacggatctcttggttccg
>OTU_3334_8
ttaaagagtaccggagctctcgggttcctactcccaccctatgttgactttaaatgttgctttggcggaccggcaatcttgccactggcctagggctagacagcgtccgccagaggatttttaaattcctttgaatgtgaattctgagtctttgaaaattgaatcaaaactttcaacaacggatctcttggttctg
>OTU_3351_33
ttaatgattgcgaatggctgccttcagtgctggctcttatgagcatgtgcacgttggtggctttcatccaataccctgtgcacctttggcctcttgctagcttcggccggcagaggatttttacacacactcgaatgtaatgaaatttattgtcgtgcgcaagcactaatgtacaactttcaacaacggatctcttggctctc
>OTU_3352_1
ttattataacaatggaggtgctggggttgttgctgacttttgaaatggttgtgcacaccttggtgctctcatatacaatccatctcacccctcttgtgcatcaccacatggaggccccttttagctagttctaaagggggttttcacgtttttatacatacaccattttaatgcaatgcgtagaatgtcatacttttgcgatcacacgcaattaatacaactttcaacaacggatctcttggctctc
>OTU_3356_1
ttaccgagttagggtcttctaggcccgacctccaaccctttgtttactataccatgttgctttggcgggcccgcctttcggggctgccgggggcttacatgcccctggtcagtgcccgccagtagccttattaaattcttccataattatgttgtctgagtataactataaaatcgttaaaactttcaacaacggatctcttggttctg
>OTU_3357_2
ttatcgaaacagaacgcccgtgggggaagggggagcgccgacggccggagactgtcgcacggcctcgtgcacgtcactcgccgtcgctccttccgaccctttgaggcttcgaccttctcaacacccgtgcacccactgtaggtccttcgggatctacgtccttcttcgaactcgcatgtctacagaacgtattcgtcgtgtcccggcctcgaccctcagggtcccgcgtcggcgaccgcaaaacctataatacaactttcagcaacggatatctaggctctc
>OTU_3363_10
ttaacgagttagggtcttctaggcccgacctcccaaccctatgtttattgaacctctgttgcttcggcgggcccgcctcacggccgccggaggagtcgccgcgaggcaccctctggccagcgtccgccgatagccaaccccccaaactccgaataaatcgtgtcatgatgtctgagtctatgattaaattaaagcaaaactttcaacaacggatctcttggttctg
>OTU_3366_6
ttaacgttggggacccccggtccctcagcgagatagaacccttgctttgagtaccacacgtttcctcggcaggtgcgcctgccaatggggaccacaaccaaaccctttgtatagcagtaaccgtatccaaaaccaaaaaaattaaaactttcaacaacggatctcttggttctg
>OTU_3371_1
ttatagagtttacaaaactcccaacccatgtggacatacctcaagttgcctcggcaggtcgtgcctacccggtaccctacctacccggtagggtctaccctgtagactcggtgaagcctgccagtggataaccaaactctgattattattgaattctgaaaacataactaaataagttaaaactttcaacaacggatctcttggttctg
>OTU_3372_2
ttaccgagttagggtagtattcactgcccgacctcccaaccctgtgtctaccacactttatcgttgcttcggcggaccggttgaccaactggtcatgaccgccgggggctggctctgtcccccctggagagcgtccgccgatggcccaaccacaaaactcttgtaccgaaacgtgtcgtctgaattattgagaaatcaaaaacaaaactttcaacaacggatatctcggctctc
>OTU_3374_34
ttaccgaattgtcaacacgagctgttgctggtcctcaaacgggggcatgtgcacgctctgtttacacatccacttacacctgtgcacccttcatagttctgtggcctgggggcaccgtcccccctgctgtggtcctatggatttaacacacacacacactgtaaccaagtctcatggaatgtacaccgcgtttaacgcaatacaatacaactttcagcaacggatctcttggctctc
>OTU_3376_16
ttatcgtacaaaatgtgtgaggcatgcgagggctgtagctgactcaaagtcgtgcacgccggagtgtgccctctcacataataatccatctcaccctttgtgcaccaccgcgtgggcaccctttgggatcagactgatctcggaggatgctcgcgttttcacacaaaccccccttttaaaagtgtagagtgacctcatttatgcgctaacccgcaatcaatacaactttcaacaacggatctcttggctctc
>OTU_3385_14
ttcataataagtgttttatggcactttttaaatccatatccaccttgtgtgcaatgtcagttgatcttctttatggagatcgaccaaacatcaacctattttttaactctttgtctgaaaaatattatgaataaataattcaaaatacaactttcaacaacggatctcttggctctc
>OTU_3387_21
ttaccgaattgtcaacacgagttgttgctggtcctcaaatgggggcatgtgcacgctctgtttacatacccactcacacctgtgcaccctctgtagttctgtggtgtgggggactctgtcctcccgctgtggttctacgtctttacacacacacagtttcatagaatgtatgtcgcgtttaacgcaatacaatacaactttcagcaacggatctcttggctctc
>OTU_3390_1
ttaatcgaacaaacatgcttctcggcatgacgtttcaaacaccacctgtgtatcttacctgttgcttccgtgctgcacatgctgtcaaggtgcttcagggtacggtctaccgagtactcgggacccccggtaccctggggagtcggcacgggagggataaccacaaactctttcctttgaatgccttccgtctgaactgtaatacatgaaaagttaaaactttcaacaacggatctcttggttctc
>OTU_3403_3
ttacaagtgaccccggctacggccgggatgttcataaccctttgttgtccgactctgttgcctccggggcgaccctgccttcgggcgggggctccgggtggacacttcaaactcttgcgtaactttgcagtctgagtaaacttaattaataaattaaaacttttaacaacggatctcttggttctg
>OTU_3411_1
ttaccgagtgagggcctccgggtccgacctccaaccccatgttatccgaccctgttgcctcgggggtgacccggccctttcggcgccggggccctcggtggaccactcaacactgcatctttgcgtctgagtcacatattgaatcaatcaaaactttcaacaacggatctcttggttctg
>OTU_3413_6
ttaccgagtttacaactcccaaacccaatgtgaacgttaccaaactgttgcctcggcggggtcacgccccgggtgcgtaaaagccccggaaccaggcgcccgccggaggaaccaaccaaactctttctgtagtcccctcgcggacgtatttcttacagctctgagcaaaaattcaaaatgaatcaaaactttcaacaacggatctcttggctctc
>OTU_3414_1
ttactgagtgcgggccctctgggtccaacctcccacccgtgtttatcgtaccttgttgcttcggcgggcctccgtgcccgccgaagacacctgtgaacgctgtatgaagattgcagtctgagcgattagctaaatcggttaaaactttcaacaacggatctcttggttccg
>OTU_3418_21
ttactgaactgtcaacacgagttgttgctggtcctcaaatgggggcatgtgcacgctctgtttacatacccactcacacctgtgcaccctctgtagttctgtggtgtgggggactctgtcctcccgctgtggttctacgtctttacacacacacagtttcatagaatgtatgtcgcgtttaacgcaatacaatacaactttcagcaacggatatctaggctctc
>OTU_3426_3
ttaccgagttcatgcccttcacggggtagacctcccaccctgtattattacgattgttgctttggcaggccgccttcgggcactcggctccggctgaggcgcgcctgccagaagactcaaaccctgtatttagtgtcgtccgagtactattttaatacgttaaaactttcaacaacggatctcttggttctg
>OTU_3427_9
ttaccgagtttacaactcccaaacccctgtgaaccataccatatcgttgcctcggcggcgccctctcgggggaccgccagaggacccaaactcttgcttttatacaagtttgttctgagtgttgctgcaagcaaaaataaatcaaaactttcaacaacggatctcttggctctg
>OTU_3432_2
ttatcgtacaaccgaggtgcaaaggctgtcgctgaccctcaaaggtcgtgcacgcccgagcgctctcgcacaatccatctcacctttgtgcatcaccgcgtgggtccccctttgcaggagggctcgcgttttcacataaaacttgacacagtctagaatgatctttttttgcggtaacacgcaatcaatacaactttcaacaacggatctcttggctctc
>OTU_3438_1
ttacagtagaggcctttggccttcacgagatgcaacccttgtatttttgtgcacctttgtttcctcggcagcttcgcctgccaacgaggaccccctaaaaaccttttgcaatcccagtataaccttctgaaaacaaaacaatattaaaactttcaacaatggatctcttggctctg
>OTU_3445_10
ttaccgagttagggtcttctaagggcccgacctccttacctttgtctaccttacctcacgttgcttcggcgggcccgtcctgtttttggaccgccggaggcttcaccccctctggcccgttgcccgccgacagcccccaaccaaaaactcttgcaataaccgtggaattgtctgaatcttgcttctaaaataaccaaaaactttcaacaacggatctcttggttctg
>OTU_3450_20
ttaacgagttagggtcttctcggcccgacctcccaaccctttgtttactgaacctttgttgcttcggcggacccgtctcacgaccgccgggggaccgtaagacgtcctctggcccgtgtccgccggtggcccaactgaacaaattctgattaaaatgtgtcaatgtctgagtagaattcataattaaaacaaaactttcaacaacggatctcttggctctc
>OTU_3471_1
ttactgaactgtcaacacgagttgttgctggtcctcaaatgggggcatgtgcacgctctgtttacatacccactcacacctgtgcaccctctgtagttctgtggtgtgggggactctgtcctcccactgtggttctacgtctttacacacacacagtctcatagaatgtatgtcgcgtttaacgcaatacaatacaactttcagcaacggatatctcggctctc
>OTU_3475_7
ttattgattggtcgaaagaccttatcagattctaccacctctgtgaaccgttgacctccgggttaataatcaaacatcagtgtaacgaacgtaagagtatcttaacgaaacaaaactttcaacaacggatctcttggctctc
>OTU_3494_7
ttaccgagcgagcgcctccgggtgcgacctccaacccttctgtgaaccaacctctgttgcctcggggtgacccgggcccgcccggcccccgaaggacaatatgacaactctgcatctttgcgtcggatatgaaaagtaattcgaaatgaaaactttcaacaacggatctcttggttctg
>OTU_3496_4
ttaccgagtgcgggccctctgggtccaacctcccacccgtgtttatcgtaccttgttgcttcggcgggcccgccgctctggcggccggggggctctcgcccccgggttagcgcccgccgaagacaccaacgaacgctgtctgaagattgcagtctgagcttattagctaaatcggttaaaactttcaacaacggatctcttggttccg
>OTU_3497_23
ttactgagtgagggcctccggcccgacctccaaccctgtgtttccaacctctgttgcctctgggggtcacctggtcctttgctgtccagggcccccggtggaccactccaactctgcgtatgagcgtccgagtccatcgttaaatgaatgaaaactttcaacaacggatctcttggttctg
>OTU_3506_6
ttaccgagtgagggccctctgggtccaacctcccacccgtgtttatcgtaccttgttgcttcggcgggcccgcctcacggccgccggggggcacctgcccccgggcccgcgcccgccgaagacaccattgaacgctgtatgaagattgcagtctgagcttattagctaaattagttaaaactttcaacaacggatctcttggttccg
>OTU_3511_15
ttaccgagtaccggagctctcgggttcctactcccaccctatgatgactttaaatgttgctttggcggaccggcaatattgccaccggcctaggctggatagcgtccgccagaggatttttaaattcctttgaatgtgaattctgagtctttgaaaattgaatcaaaactttcaacaacggatctcttggttctg
>OTU_3512_1
ttaaagagtaccggagctctcgggttcctactcccaccctatgttgactttgaatgttgctttggcggaccggcaatattgccaccggcctaggctgggtagcgtccgccagaggatttttaaattcctttgaatgtcaactctgagtctttgaaaattgaatcaaaactttcaacaacggatctcttggttctg
>OTU_3518_3
ttaccgagttcatgcccttacgggtagatctcccaccctgtgttatcattacctttgttgctttggcgggccgccaggctccggtcaggctatcggcttcggctggtacgcgcccgccagaggaccctaacattctgattatcagtgtcgtctgagtaatattataataagttaaaactttcaacaacggatctcttggttctg
>OTU_3520_13
ttatcgtacaaaatgtgtgaggcatgcgagggctgtagctgactcaaagtcgtgcacgccggagtgtgtcctctcacataacaatccatctcaccctttgtgcaccaccgcgtgggcaccctccgatctcggagggggctcgcgttttcacacaaaccccccttttaaaagtgtagagtgacctcatttatgcgctaacccgcaatcaatacaactttcaacaacggatctcttggctctc
>OTU_3540_20
ttatcgtagggcttcggccctgtcgagatagaacccttgcctttttgagtaccttttcgtttcctcggcaggctcgcctgccaatggggaccccaacaaacactttgcagtacctgtaaacagtctgaacaaacttttaaaaattaaaactttcaacaacggatctcttggttctg
>OTU_3541_1
ttaccgagttcatgccctcacgggtagatctcccaccctatgttatcattacctttgttgctttggcgggccgccaggcttcggctaggctaccggctccggctggtaagcgcccgccagaggaccccaaactctgattatcagtgtcgtctgagtactatataatagttaaaactttcaacaacggatctcttggttctg
>OTU_3550_5
ttagtgattgtgaatcgttgcctttggtgctggctcttgtacaagtgcaagggcaagtgcacattggtgactttcatccaataacccatgtgaacctttggcctcttgttggcttcggctgacagaggattattttacacattaactcgaatgtaatgaaaactactgttgtgcgcaagcacgaatgtacaactttcaacaacggatctcttggttctg
>OTU_3556_13
ttatcgagttagggtcttctcaggcccgacctccttacctttgtctaccttacctcacgttgcttcggcgggcccgtcctgtttttggaccgccggaggcttcaccccctctggcccgttgcccgccgacagcccccaaccaaaaaactcttgcaataactgtggaattgtctgaatcttgcttctaaaataaccaaaaactttcaacaacggatctcttggttctg
>OTU_3568_18
ttatcgtacaaaatgtgagagaggcatgcaagggctgtcgctgactccaagtcgtgcacgccggagtgtgccctctcacataataatccatctcaccctttgtgcaccaccgcgtgggcaccctttgggatcagactgatctcggaggatgctcgcgttttcacacaaaccccccttttaaaaagtgtagaatgacctcatttatgcaatcaatacaactttcaacaacggatctcttggctctc
>OTU_3576_3
ttaccgagttagggtcttccaggcccgacctccaaccctttgttaactatactatgttgctttggtgggcccgcctttaggggccgctggggactttgcccctggtcagtgcccgccagtagccttctcaaattcttttataattgtgtcgtctgagtaaagatataaatcgttaaaactttcaacaacggatctcttggttctg
>OTU_3592_2
ttaccgagtgcgggccctctgggtccaacctcccacccgtgtctatcgtacctcgttgcttcggcgggcccgcgcccgccgaagacacctttgaacgctgtctgaaggttgcagtctgagcttgattagcaaatcaatcaaaactttcaacaacggatctcttggttccg
>OTU_3595_1
ttatcgtggggctccggccccgtcgagatagaacccttgcctttttgagtaccattttcgtttcctcggcgggctcgcccgccaacggggaccaaccacaaacgctttgtagtacctgtaattgtctgaaaaacaagcaaaaaattaaaactttcaacaacggatctcttggttctg
>OTU_3596_1
ttatctattccatgaggtgcggtcgcggccctcggcgggagcaacagctaccgtcgggcggtagaggtaacactttcacgcgccgcatgtctgaatcctttttttacgagcacctttcgttctccttcggcggggcaacctgccgttggaacctatcaaaaccttttttttgcatctagcattacctgttctgatacaaacaatcgttacaactttcaacaatggatctcttggctctg
>OTU_3597_1
ttaatgaacggcctctgggccttcaaccttgcaaacctgtggaagcaacatgtgcttcggcgctttggcgtcgcttttatgctacgaatcagtctttgtgtctgaatcaaatacaagaaataaaactttcaacaaaggatctcttggctctc
>OTU_3599_4
ttattgattgcgaatcgttgccttcagtgctggctcttaggagcaagtgcacgttggtggctttcatccaacaccctgtgaacctttggcctcttgctagcttcggctggcagaggatttttataacacactcgaatgtaatgagaactattgtcgtgcgcaagcactaatgtacaactttcaacaacggatctcttggctctc
>OTU_3601_2
ttaccgagttcctgcccctggggtagatctcccacccattgtgttcgttaccgtgttgctttggcgggccgccggggccagccccgccgccggctccggccgtcgcgcgcccgccagaggccctacaaaccctgagtgtcagtgtcgtccgagtactacgtaatagttaaaactttcaacaacggatctcttggttctg
>OTU_3611_2
ttaacaaatccaataattgcgggagaacgtttgttctttttgcaaatattaatccgctggcttgctcttagtagcagctggctgtgatataaatgcgtgatatgttgacagtgtgtgtataattaaatattgacaacttttaacaatggatctctaggctctt
>OTU_3616_2
ttacctagagtttgtgggctttgcccactgtctcttacccatgtcttttgagtacttacgtttcctcggtgggctcgcccgccggttggacaatttaaaccatttgcagttgcaatcagcgtctgaaaaacataatagttacaactttcaacaacggatctcttggttctg
>OTU_3617_30
ttactgaactgtcaacacgagttgttgctggtcctcaaatgggggcatgtgcacgctctgtttacatacccactcacacctgtgcaccctctgtagttctgtggtgtgggggactctgtcctcccgctgtggttctacgtctttacacacacacacacagtttcatagaatgtatgtcgcgtttaacgcaatacaatacaactttcagcaacggatatctaggctctc
>OTU_3621_17
ttattgaaataaacctgatgggttgttgctggctctctagggagcatgtgcacaccttgtcatctttatatctccacctgtgcactttttgtagacctttcaggtctatgttgcttcatttaccccaatgtatgttaatagaatgttgtgcctatataatatatacaactttcagcaacggatctcttggttctg
>OTU_3623_1
ttactgagtgagggccctctgggtccaacctcccacccgtgtttattgtaccttgttgcttcggtgagcccgcctcacggccgccggggggcttctgcccccgggcccgcgcccaccgaagacaccattgaactctgtctgaagattgcagtctgagcataaactaaataagttaaaactttcaacaacggatctcttggttccg
>OTU_3629_9
ttaccgagttagggtcttctaagggcccgacctccttacctttgtctaccttacctcacgttgcttcggcgggcccgtcctcttttggaccgccggagggttgaacccctctggcccgttgcccgccgacagcccccaaccaaaaaactcttgcaataactgtggaattgtctgaatcttgcttctaaaataaccaaaaactttcaacaacggatatctaggctctc
>OTU_3640_14
ttaacgagttagggtcttccaggcccgacctcccaaccctatgtttattgaacctctgttgcttcggcgggcccgtctcacgaccgccggaggagtcgccgcgaggcaccctctggccagcgtccgccgatagccaacctcaaaactctgaataaatcgtgtcatgatgtctaagtctatgattaaattaaagcaaaactttcaacaacggatctcttggttctg
>OTU_3649_4
ttaaagagtaccggagctctcgggttcctactcccaccctatgttgactttaaatgttgctttggcggaccggcaatcttgccactggcctaggctggacagcgtccgccagaggatttttaaattcctttaaaaggtgaattctgagtctttgaaaattgaatcaaaactttcaacaacggatctcttggttctg
>OTU_3652_17
ttaccgagtgagggccctttgggtccgacctcccacccgtgtttatcgtaccttgttgcttcggcgagcccgccacttgtggccgccggggggcacctgcccccgggcccgcgcccgccgaagacaccattgaacgctgtatgaagattgcagtctgagcttattagctaaattagttaaaactttcaacaacggatctcttggttccg
>OTU_3657_1
ttaccaaagatggcgggcctccgggcccccgtccccaacccattgttgactcaaacccctttttgcctcgggggcagagcgtccgccgccgcccccggaggaccatttaacgctgtctttttgcgtcggagtattgattaaatgaatgaaaactttcaacaacggatctcttggttctg
>OTU_3670_2
ttaaagattcgaccttcgggtctatcttctcttcagctgtgcgcttttgctgcacgctgttgaacttcacacacctgtgcacacttcggttgtggctagttaactctagtcactgccgcttttacatttaacaaccaagtctagaatgtcatttacaataaaaataacaactttcaacaacggatctcttggctctc
>OTU_3671_28
ttactgattgcgaatcgttgccttcagtgctggccggcttcggcaagtgcacgttggtgactttcatccaataccctgtgaacctttggcctcttgctagcttcggccggcagaggatttttacacacactcgaatgtaatgaaatttattgtcgtgcgcaagcactaatgtacaactttcaacaacggatatctaggctctc
>OTU_3680_16
ttatcgtacaaaatgtgtgaggcatgcaagggctgtcgctgactccaagtcgtgcacgccggagtgtgccctctcacataataatccatctcaccctttgtgcaccaccgcgtgggcaccctttgggatcagactgatctcggaggatgctcgcgttttcacacaaaccccccttttaaaagtgtagagtgacctcatttatgcgctaacccgcaatcaatacaactttcaacaacggatctcttggctctc
>OTU_3689_4
ttaaagagtaccggagctctcgggttcctactcccaccctatgttgactttgaatgttgctttggcggaccggcagtattgccaccggcctaggctggatagcgtccgccagaggatttttaaattcctttgactgaattctgagtctttaaaaattgaatcaaaactttcaacaacggatctcttggttctg
>OTU_3691_2
ttactgaattgttaacaagagttgttgctggtccctatacgggggcatgtgcacgctctgtttacacatccactcacacctgtgcaccctctgtagttctatggcctggggggctctgtcctcctgctgtggctctacgtctttacacacacacactgtaacaaagtctcatggaatgtatgtcgcgtttaacgcaatgaaatacaactttcagcaacggatctcttggctctc
>OTU_3692_2
ttaccgagtttacaactcccaaacccctgtgaacataccaaactgttgcctcggcggggtcacgccccgggtgcgtcgcagccccggaaccaggcgcccgccggagggaccaaccaaactctttctgtagtcccctcgcggacgttatttcttacagctctgagcaaaaattcaaaatgaatcaaaactttcaacaacggatctcttggttctg
>OTU_3694_26
ttaacgagttagggtcttctcggcccgacctccccaccctatgtttgctgaacctttgttgcttcggcggacccgtctcacgaccgccgggggaccgtaagacgtcctctggcccgtgtccgccggtggcccaactgaacaaattctgattaaaatgtgtcaatgtctgagtagaattcataattaaaacaaaactttcaacaacggatctcttggttctg
>OTU_3706_1
ttaacgagttagggtcttctaggcccgacctcccaaccctatgtttattgaacctctgttgcttcggcggacccgcctcacggccgccggaggaccgctgaaaggcgtcctctggccagcatccgccgatagccaaccacttaaactctgaataaatcgtgtcatatgtctaagtctatgattaaattaaagcaaaactttcaacaacggatctcttggttctg
>OTU_3714_7
ttaccgagttcatgccctttgggtagacctcccaccctgtgtcgttatacctttgttgctttggcgggccgcggggcttaggccctgcccctggctccggctagggcgcgcccgccagaggacctcaaaacctgaatgttagtgtcgtctgagtactatataatagttaaaactttcaacaacggatctcttggttctg
>OTU_3718_4
ttaccgagttagggtcttctaggcccgacctccaaccctttgttaactataccatgttgctttggtgggcccgcctttcagggccgccgggggcttttatacccttggtcagtgcccgccagtagccttattaaattctttcataattatgtttgtctgagttataaaattaatcgttaaaactttcaacaacggatctcttggttctg
>OTU_3722_12
ttacagagttctcgccctcgcgggtagatctcccacccactgtgattgctaccgtgttgctttggcgggccgccgggcccagcccagccgccggccccggccggcgcgtgcccgccagaagccccgcaaactctgaatgtcagcgtcgtctgagtactatataatagttaaaactttcaacaacggatctcttggttctg
>OTU_3723_2
ttaccgagtactataactcataaccctttgtgaacctttatacctgttgcttcggcggcgcgcctcccggggcgtgcccgccggcattatcagaatctctgttcgaacccgacgatacatctgagtgttctaagcgaactgttaaaactttcaacaacggatctcttggctcca
>OTU_3726_8
ttgtcgaaagtaaacgcggatcaatccgcgaacttctaaacctttggcgattgactcatgttgcctcggcgggttctctcgccagaggatacatcaaaactcctgttttaacggtgttgtctgagctacaagcaacgaatcaaaactttcaacaacggatctcttggttctg
>OTU_3729_29
ttactgaattgtcaacatgaagttgttgctggtcctcaaacggggacatgtgcacgctctgttcacacatccaatcacacctgtgcaccctctgcggttctgtggtcaggggggctttgccttcctgtgctgtggttctgcttctttacacacacacactgtaacaaagtcttgtggaatgtatgctgcgtttaacgcaatacaatacaactttcagcaacggatctcttggctctc
>OTU_3739_29
ttaccgaacgtcgacacgagttgctgctggctctctagagagcatgtgcacacttgtcgtctttatatctccacctgtgcacctattgtagacctggatgactctctgaatggctatcattcaggtatgaggattgactttctgcctctccttacatttccaggcctatgttctttcatataacctcaatgtatgttatggaatgtaataattatggccttctgtgccttataaacctatacaactttcagcaacggatctcttggctctc
>OTU_3745_1
ttaacgaataaacttgatcaggctgttgctggcccccttgcaggggtatgtgcacgcttgtcatatttatcatttctccaactgtgcacatattgtagacctggatcttttttgaagaaattcaagttgggggactgctgtgctctttgttttttcaagagtcggctttccctcgtatttttcaggtctatgtcattttcacaacctctagaatgtgtttagaatgttgaatcatatataaagttaatatacaactttcagcaacggatctcttggctctc
>OTU_3746_1
ttactgaactgtcgacacgagttgttgctggtcctcgagagggggcatgtgcacactctgtttacacacccactcacacctgtgcaccctctgtagctctgtggtgtgggggccctgtcctcccactgtggttctacgtctttacacacacacacacagtgataagtctcatggaatgtatgtagcgtttaacgcaatacaatacaactttcagcaacggatctcttggctctc
>OTU_3751_9
ttactgaattactgagaggttgtagctgtctcttcggagaatgtgcacgccgctcaaattcatcttaacctcctgtgcactgttgtagactatgataactctcaactacctagtagttggattgaaggacgtgcgcttttagctgtctttcaatatcgtagtctatgttttatctatatacaaaaagtctagaatgtcgttcatgggtcttgtacctataaactttatacaactttcaacaacggatctcttggttctg
>OTU_3763_13
ttaccgagtgcgggccctctgggcccaacctcccacccgtgtttcattattaccttgttgcttcggcgggcccgccgttccggccgccggggggctttcacgtccccgggcccgcgcccgccgaagacacctccgaacgctgcctgaaggttgcagtctgagtgattatgataaatcagttaaaactttcaacaacggatctcttggttccg
>OTU_3766_1
ttaacgagttagggtcttctaggcccgacctcccaaccctatgtttactgtaccattgttgcttcggtgggtccgtctaacgaccgccgggggaccgccgcgaggcccccctggccagcacccgccgatagccaaatttaaacccttatgaatcgtgtcttatgtctaaatcatgattaaaaatcaaaacaaaactttcaacaacggatctcttggttctg
>OTU_3775_1
ttaccgagttagggtcttctaggcccgacctccaaccctttgttaactataccatgttgctttggtgagcccgcctttaggggccgccggggactttgcccctggtcagtgcttaccagtagccttcttaaattcttataattatgtcgtctgagtatagatataaattgttaaaactttcaacaacggatctcttggttctg
>OTU_3779_5
ttatcgagtgaagggctctctgagaacccgacctcccaacccttgtgtttcttaccactttgcttcggcgggcccgtcactgctgtgaccgccgaagggttatgttttttaactagcctttgggcccgtgcccgccggagatacctgaacgcttgttatgaaataggttgtctgagttataaagaaaataagtcaaaactttcaacaacggatctcttggttccg
>OTU_3797_5
ttattgtaaaaccgaggtgcaagggctgtcgctgaccttccacggtcgtgcacgcccgagcgctctcaccacaatccatctcacccctttgtgcatcaccgcgtgggtccccctttgcaggagggctcgcgttttcacataaaacttgacacagtctagaatgtttttcttttgcggtcacacgcaatcaatacaactttcaacaacggatctcttggctctc
>OTU_3815_2
ttaccgagttcatgcccttacgggtagacctcccaccctatgtgatattacctttgttgctttggcgggccgccaggcttcggctaggctaccggctccggctggtaagcgcccgccagaggaccccaaactctgaatgttagtgtcgtctgagtactatctaatagttaaaactttcaacaacggatctcttggttctg
>OTU_3841_1
ttacagagttgcaaaactccctaaaccattgtgaacgttacctaaaccgttgcttcggcgggcggcccgggtctctcccggcgcccctgggcccctcgcgggcgcccgccggaggtaaaccaaactattgcattgtatggcctctctgagtcttctgtactgaataagtcaaaactttcaacaacggatctcttggttctg
>OTU_3847_2
ttaaagagtaccggagctctcgggttcctactcccaccctatgatgactttaaatgttgctttggcggaccggcaatattgccaccggcctaggctggatagcgtccgccagaggatttttaaattcttttgactgaatttctgagtctttgaaaattgaatcaaaactttcaacaacggatctcttggttctg
>OTU_3848_9
ttactgagaatgctctccggagccctcaaaccttgcgtatttatcttgtacttgcgggtggccctcgggtgcccgcgaggattcaaactgtttgactgccgtcagatttttgtctaaaaaacaactttcaacaacggatctcttggttctg
>OTU_3852_5
ttagtgattgtgaatcgttgcctttggtgctggctcttgtacaagtgcaagggcaagtgcacattggtgactttcatccaataacccatgtgaacctttggcctcttgttggcttcggctgacagaggattattttacacattaactcgaatgtaatgaaaactactgttgtgcgcaagcacgaatgtacaactttcaacaacggatatctaggctctc
>OTU_3875_1
ttaccgagtgcgggccctctgggtccaacctcccacccgtgtttatcgtaccttgttgcttcggcgggcccgccactctggcggccggggggctcttgcccccgggctagtgcccgccgaagaccccaacgaacgctgtctgaagattgcagtctgagcttattagctaaatcggttaaaactttcaacaacggatctcttggttccg
>OTU_3880_3
ttaaaagtaaatgtgttcttaggaatatgcattttgaaacccatcagtgtattctacctgttgcttccgtactgcgtggactgcggtcccctcttgccgtaccttgggtacggttcagagtgtcggtatggggggggtatacatcaaactcttgcatttcgtgtcatcagtctgaactgtttataaaaacaagttaaaactttcaacaacggatctcttggttctc
>OTU_3881_2
ttaccgagtttacaactcccaaacccctgtgaacatacctatcgttgcctcggcgggatcgccccggtgcctccgggcccggacccaggcgcccgccgcaggaccctaaactcttgttttctatacgaatcttctgagtgacacaagcaaataaattaaaactttcaacaacggatctcttggttctg
>OTU_3888_2
ttagtgaattaaacatgcttggtgtcttcgcttcggcaaggcccttgcgtaactcacatcccaacacctgtgaactgtaaggcgtacgatgaggcttcggctcaatcgtcgtctgccctttttaacaaacgatcaatgtaacaaacgtagtcttattataacctaataaaactttcaacaacggatctcttggctctc
>OTU_3891_1
ttactagggggcttcggccccatcaagataccaccccttgcattttgagtaccttctgtttcctcggcgggcctgcccgccaacggggaccacaccaaacccttctgtagtcgcagtaaacgtctaaaaacaaaataatcaaaactttcaacaacggatctcttggttctg
>OTU_3894_3
ttaaagagtaccggagctctcgggttcctactcccaccctatgttgactttaaatgttgctttggcggaccggcgatctcgccactggcctaggctggacagcgtccgccagaggatttttaaattcctttaaaaggtgaattctgagtctttgaaaattgaatcaaaactttcaacaacggatctcttggttctg
>OTU_3908_3
ttcataatgagtgtttatggcacttttaaaaatccatacccaccttgtgtgcatcgttagtcatgttccttgttctctcttgagaggacgaggaggcaggcttcaaattaatctatctttaaccagtttgtctgaagcaatgtatcatgagttaacataattctaaaatacaactttcaacaacggatctcttggctctc
>OTU_3910_4
ttaacgagttagggtctctcacggcccgacctcccaaccctttgtttatcgaacctctgttgcttcggcggatccgtccctcgggaccgccggaggatcgtcgtaaggcgtcctctggcccgtatccgtcgatagccaaccacttaaactcagaataaatcgtgttataattgtctaagtttatcataaaaatcaataaagcaaaactttcaacaacggatctcttggttctg
>OTU_3911_8
ttaccgagttagggtcttctaagggcccgacctccttacctttgtctaccttacctcacgttgcttcggcgggcccgtcctcttttggaccgccggagggttgaacccctctggcccgttgcccgccgacagcccccaaccaaaaactcttgcaataaccgtggaattgtctgaatcttgcttctaaaataaccaaaaactttcaacaacggatctcttggttctg
>OTU_3912_1
ttaaagagtaccggagctctcgggttcctactcccaccctatgttgactttaaatgttgctttggcggaccggcaatcttgccactggcctagggctagacagcgtccgccagaggatttttaaattcctttgaatgtcaactctgagtctttgaaaattgaatcaaaactttcaacaacggatctcttggttctg
>OTU_3920_6
ttaaagagtaccggagctctcgggttcctactcccaccctatgttgactttaaatgttgctttggcggaccggcaatcttgccactggcctaggctggacagcgtccgccagaggatttttaaattcctttgaatgtgaattctgagtctttgaaaattgaatcaaaactttcaacaacggatctcttggttctg
>OTU_3923_22
ttaacgtatctctgacggcgagggtccgcccagctccgtaccgaaagggcgtgcagcgggtgggcctgagtcgctcaacctttgcaaacccactgtgcacttttgtcggcggagggcccctgtggccccgagccggatcacaaactccaagtttgatttgaatgtgaagcggacatgtgccgcctaaactttaacaactttcaacaacggatctcttggctctc
>OTU_3926_5
ttacaagaagccgaaaggctacttcaaaccatcgcgaactcgtccaagttgcttcggcggtgcggctccccccggggggccgcggcgccgcgcctccccggaggtgcggggcgcccgccggaggtcagaaactctcatgtattatagtggcatctctgagtaaaacacaaataagttaaaactttcaacaacggatctcttggttctg
>OTU_3931_4
ttaccgagtttacaactcccaaacccctgtgaacataccatttgttgcctcggcggtgcctgcttcggcagcccgccagaggacccaaacccttgattttatatgtaacttctgagtaaaaccataaataaatcaaaactttcaacaacggatctcttggttctg
>OTU_3958_7
ttcataataaagtgttttatggcactttttaaaaaaatccatatccaccttgtgtgcaatgttaagatctcattagtcaagtaatattttctgatgggattttttatacatcaactcattcttaaactaatttgtctgaaaaatattatgaatacttaattcaaaatacaactttcaacaacggatctcttggctctc
>OTU_3965_1
ttcataatgagtgtttatggcacttttaaaaatccatacccaccttgtgtgcatcgttagtcatgttccttgttctctcttgagagagcgagcgaggaggcagacttcaaattaatctatctttaaccagtttgtctgaagcaatgtatcatgagttaacataattctaaaatacaactttcaacaacggatctcttggctctc
>OTU_3967_1
ttactgagaaattgccctttgggtagatctcccaccctttgtttacattacctttgttgctttggcaggcccgtctttggaccaccggcttaggctggtctgcgcctgccaaaggaccctaaactcttgatttttgtattgtctgagtaatattataataagttaaaactttcaacaacggatctcttggttctg
>OTU_3977_1
ttatcgaattttgaaagggctgttgctgatcttttagatacgtgcacgccttgttccaaatttctacaccttgtgcacattttgtagaccggtttcattagaaatcggtttatgttttactatatatacttgttttagaatgtcatataataagcttgttagcaagcttaacaaatttaatacaactttcaacaacggatatctcggctctc
>OTU_3978_3
ttactgagtgcaaactctccaaccattgtttatctacctattttgcttctccggcaggcggttccagggagggaccaagcctatggcctagcctaagcacctgccgaaggggtacctactgctctatattatactgtctgagaaaacaaaaaaaaaattaaaattttcaattgtggatctcttggttctg
>OTU_3992_9
ttatcatacaaccgaggtgcgagggctgtcgctgaccccgtagaaaggtcgtgcacgcccaagtgctctcacacaatccatcacacccccttgtgcatcaccgcgtggggtccccctttgccgggagaacctgcgtttccacattaaactcgataaagtgtagaatgtttatttttgcggtcacacgcaatcaatacaactttcaacaacggatctcttggctctc
>OTU_4006_5
ttaccgagtgagggcctccggcccgacctccaaccctttgttgaccaacacttgttgcctcgggggcgacccggacgccgacgcgtcggccgtcgggcaccccggtggaccactccaactctgtatctgtacgtcggagtacttgatgaatcaatcaaaactttcaacaacggatctcttggttctg
>OTU_4019_24
ttattgaataaacttgatcaggttgttctgctggccccctctccagggggtatgtgcacgcctgtcatctttatattctcccaactgtgcaccttttgtagacctgattgaaattgattgattcatctttcgagttggggactgctgcgcttttctcaagtcggctttcccttgtattttttcaggtctatgtcattatcacaaacctatgaatctgttttagaatgttgaattgggtctttttgtacctataaagttaaatatacaactttcagcaacggatctcttggctctc
>OTU_4031_19
ttaaagagtaccggagctctcgggttcctactcccaccctatgttgactttaaatgttgctttggcggaccggctgtactgccaccggcctaggctggatagcgtccgccagaggatctttaaattccttcgaatgtgaattccgagtctttgaaaattgaatcaaaactttcaacaacggatctcttggctctc
>OTU_4038_1
ttaccgagttagggtagtattcactgcccgacctcccaaccctgtgtctaccacactttgtcgttgcttcggcggaccggttgaccaactggtcatgaccgccgggggctggctctgtcccccctggagagcgtccgccgatggcccaaccataaaactcttgtaccgaaacgtgtcgtctgaatcatgtttgagaaatcaaaaacaaaactttcaacaacggatctcttggttctg
>OTU_4041_1
ttaccgaattgtcaacacgggttgttgctggtcctcatatgggggcatgtgcacactctgttcacacatccactcacacctgtgcaccctccgtagttctgtggcatgggggactctgtcctcttgccgtggttctacatctttacacacactgtaataaagtcttatggaatgcatatcgcgtttaacgcaatacaatacaactttcagcaacggatctcttggctctc
>OTU_4042_4
ttaccgagttagggtagtcactcactacccgacctcccaaccctatgtctaccacactttgtcgttgcttcggcggaccggttgaccaactggtcatgaccgccgggggctggctttacccccctggagagcgtccgccgatagcccaaccacaaaactcttgtaccgaaacgtgtcgtctaaatcatgtttaagaaatcaaaaacaaaacttttaacaacggatctcttggttctg
>OTU_4048_1
ttacagagttgcaaaactcccaaacccctgtgaacatacctattgttgcctcggcggtgcctgttccgacagcccgccagaggaccccaaaccctgattacatttaagaagtcttctgagtaaccgattaaataaatcaaaactttcaacaacggatctcttggttctg
>OTU_4058_1
ttacagagactctgccctttgggtagacctcccaccctgtgtcgttatacctttgttgctttggcgggccgcggggctccggccctgcccctggctccggctagggcgcgcccgccagaggatctcaaaacctgaacgttagtgtcgtctgagtactatataatagttaaaaaactttcaacaacggatctcttggttctg
>OTU_4072_1
ttacagagttcatgcccttcggggtagatctcccacccttgtgtatcattatagaatgttgctttggcgggccgcgtgcctagcacgcctcgattcgcgtcgagcgtgtgcccgccagaggacccctaaactctgaatgttaatgtcgtctgaagtactattcaatagttaaaactttcaacaacggatatctcggctctc
>OTU_4075_3
ttaaagagtaagggtcttctaggcccgatctcccaaccctttgtttattgaacctctgttgcttcggcggatccgtctcacggccgccggaggaccgctgaaaggcgtcctctggccagcatccgccgatagccaaccacttaaactctgaataaatcgtgtcatatgtctaagtttatcataaaaattaaagcgaaaactttcaacaacggatctcttggttctg
>OTU_4080_13
ttatcgtacaaaatgtgagagaggcatgcaagggctgtcgctgactccaagtcgtgcacgccggagtgtgtcctctcacataacaatccatctcaccctttgtgcaccaccgcgtgggcaccctccgatctcggagggggctcgcgttttcacacaaaaccccccccctttaaaaagtgtagaatgacctcatttatgcaatcaatacaactttcaacaacggatctcttggctctc
>OTU_4084_4
ttaaagagtaagggtcttctaggcccgatctcccaaccctttgtttattgaacctctgttgcttcggcggatccgtctcacggccgccggaggaccgctgaaaggcgtcctctggccagcatccgccgatagccaaccacttaaactctgaataaatcgtgtcatatgtctaagtctatgattcaaatcaaataaaagcaaaactttcaacaacggatctcttggttctg
>OTU_4095_1
ttaccgagttttcaactccctaacccttctgtgaacctacctatcgttgcttcggcggactcgccccagcccggacgcggactggaccagcggcccgccggggacctcaaactcttgtattccagcatcttctgaatacgccgcaaggcaaaacaaatgaatcaaaactttcaacaacggatctcttggctctg
>OTU_4099_16
ttaacgagttagggtcttctcggcccgacctcccaaccctatgtttactgaacctttgttgcttcggcggacccgttcttacgaccgccgggggaccgtaagacgtcctctggcccgtgtccgccggtggcccaactgaacaaattctgattaaaattgtcaatgtctgagtagtattcataattaaaacaaaactttcaacaacggatctcttggttctg
>OTU_4115_2
ttaccgagtttacaactcccaaacccctgtgaacataccacttgttgcctcggcggatcagcccgctcccggtaaaacgggacggcccgccagaggacccctaaacccttgattttatacagtatcttctgagtaaatgattaaataaatcaaaactttcaacaacggatctcttggttctg
>OTU_4117_25
ttactgagttgttgacacgagctgttgctggtcctcaaagcaaggggggcatgtgcacgctctgttcacacatccactcacacctgtgcaccctccgtagttctatggccttgggggcctctgtcccctttgcccacggttctacgtctttacacatacaccgtagtaaagtcttatggaatgtgcgccgcgtttaacgcaataaaatacaactttcagcaacggatctcttggctctc
>OTU_4134_1
ttaaagagtaccggagctctcgggttcctactcccaccctatgttgactttgaatgttgctttggcggaccggcagtattgccaccggcctaggctggatagcgtccgccagaggatttttaaattcctttaaaaggtgaattctgagtctttgaaaattgaatcaaaactttcaacaacggatatctcggctctt
>OTU_4142_7
ttaaagagtaagggtcttctaggcccgatctcccaaccctttgtttattgaacctctgttgcttcggcggatccgtctcacggccgccggaggaccgctgaaaggcgtcctctggccagcatccgccgatagccaaccacttaaactctgaataaatcgtgtcatatgtctaagtctatgattaaattaaagcaaaactttcaacaacggatatctaggctctc
>OTU_4147_1
ttacagagttcatgccctcacgggtagatctcccaccctttgagtactatacttttgttgctttggcaggccgcttcggctaccggcttcggctggtgagtgcctgccagaggaccccaaactctgaattatagtgtcgtctgagtactatctaatagttaaaactttcaacaacggatctcttggttctg
>OTU_4152_3
ttactgaatggccttttgggccttcaaccttgtaaacctgtggaagcaagatgtgctttggcgccttggcgccacttttatgcttgaacaagtatttgtgtctgaataaaatacaagaaataaaactttcaacaaaggatctcttggctctc
>OTU_4162_11
ttaaagagtaccggagctctcgggttcctactcccaccctatgatgactttaaatgttgctttggcggaccggcaatattgccaccggcctaggctggatagcgtccgccagaggatttttaaattcctttgaatgtgaattctgagtctttgaaaattgaatcaaaactttcaacaacggatatctaggctctc
>OTU_4165_2
ttagtgaacgcccttttgggcttataactatccaaacctctgtgaactgtgcccttcggggctttttaatacaaacactgtgtaatgaacgtcatatatcataacaaaacaaaactttcaacaacggatctcttggctctc
>OTU_4166_7
ttaacaaatccaatacttgtggggggactaccaagtctttctgcaaatattaatccgccggctcttagcagctggctgtgataatatatatgcgtgtaatgttgacagtgtgtgtgattaatttaatttattgacaacttttaacaatggatctctaggctctt
>OTU_4171_6
ttactgagtgagggcctctggcccgacctccaaccccatgttatccgaccactgttgcctcgggggcgacccggccttcgggcgccggggcctccggtggaccttctaactctgcatctttgcgtctgagtggattattgaatcaatcaaaactttcaacaacggatctcttggttctg
>OTU_4175_5
ttaccgagaaactgccctttgggtagatctcccaccctttgtttacattacctttgttgctttggcaggcccgtctttggaccgccggcttaggctggtctgtgcctgccagaggatcctaaaactcttgatttttgtattgtctgagtactatgtaatagttaaaaaactttcaacaacggatctcttggttctg
>OTU_4195_1
ttacctagagttgtaggctttgcctgctatctcttacccatgtcttttgagtaccttcgtttcctcggcgggtccgcccgccgattggacaatttaaaccatttgcagttgcaatcagcgtctgaaaaaacttaatagttacaactttcaacaacggatctcttggttctg
>OTU_4201_1
ttaccgagttagggtcttccaggcccgatctccaaccctttgttgactataccatgttgctttggtgggcccgcctttaggggccgccggggactttcatgcccctggtcagtgcctgccagtagccttctaaaattcttttataattatgtcgtctgagtatagatataaatcgtttaaaactttcaacaacggatctcttggttctg
>OTU_4217_4
ttatcgtacaatggaggtgctagggctgtcgctgaccctttgaagggtcgtgcacgcccaagtgctctctcacatccatctcacccctttgtgcatcaccgcgtgggctacctttttggctttattcaaaaaggttggttcgcgtttttacacacacacctttatgtatagaatgtcttaatttttgcggtcatacgcaataaataaataatacaactttcaacaacggatctcttggctctc
>OTU_4218_1
ttcataataagtgttttatggcactttttaaatccatatccaccttgtgtgcaatgtcggtcgatcttcttcttggggatcgaccaaacatcaacctttatcttttaactctttgtctgaaaaatattatgaataaacaattcaaaatacaactttcaacaacggatctcttggctctc
>OTU_4222_1
ttacagagttcatgcccctcggggtagatctcccacccttgtgtatcattatagaatgttgctttggcgggtcgcgcctcgtgcgcctagattcgcgtctagcgtgcccgccagaggacccctaaactctgaatattggtgtcgtctgagtactatataatagttaaaactttcaacaacggatctcttggttctg
>OTU_4242_21
ttattgattgcgaatcgttgtcttcagtgctggccagctctggcaagtgcacgttggcagcttttcatccaatacccctgtgaacctttggcctcttgctagcctcggccggcagaggattttttacacccactcgcatgtaatgaaattattgttgtgcgtaagcactaatgtacaactttcaacaacggatctcttggctctc
>OTU_4250_1
ttagtgaagatttgggcaggccatacggacgccaaaaagtgtccctggccgcctacacccactatacatccacaaacccgtgtgcactgtcttggagaaaggcttcagagaagttttttgtggcctctcttggggtctttcttcgctacaaactcgaatggttagtatgaacgtggaacttggttggaccgtcactggccaacaaactatacacaactttcgacaacggatctcttggttctc
>OTU_4257_5
ttatcgtacaatggaggtgctggggttgtcgctgacctttgaaagggtcgtgcacgcctcggtgctttcacacacaatccatctcaccccttttgtgcatcaccgcgtgggggtcccttttagctagttctgaagggggctttcgcgtttttacaaacacacccttttaatgcaatatgtagaatgtcttactttttgcgatcacacgcaatcaaaactttcaacaacggatctcttggttctg
>OTU_4259_1
ttaccgagttcctgccctcgcgggtagatctcccaccctgtgttatcattacctttgttgctttggcgggccgtcaggcttcggtcaggctaccggctccggctggtacgcgcccgccagaggaccccaaactctgaatattagtgtcgtctgagtactatctaatagttaaaactttcaacaacggatctcttggctctc
>OTU_4275_24
ttatcgtacaaaatgtgtgaggcatgcgagggctgtagctgactcaaagtcgtgcacgccggagtgtgtcctctcacataacaatccatctctcaccctttgtgcaccaccgcgtgggcaccctccgatctcggagggggctcgcgttttcacacaaaccccccttttaaaagtgtagagtgacctcatttatgcgctaacccgcaatcaatacaactttcaacaacggatctcttggctctc
>OTU_4278_1
ttattgaataaacctgatggactgttagctggcttttcgaagcatgtgctcgtctgtcatctttatctctccacctgtgcacattttgtagtcttggatacctctcgaggcaactcggattttaggatcgccgtgctgtacaagttggcttttctttcatttccaagactatgtttttatatatacaccaaagtatgtttatagaatgtcatcaatgggaacttgtttcctataaaattatacaactttcagcaacggatctcttggctctc
>OTU_4281_11
ttacagaaagtaaacgcggatcaatccgcgaacttctaaacctttggcgattgactcatgttgcctcggcgggttctctcgccagaggatacatcaaaactcctgttttaacggtgttgtctgagctacacgcaatcaatacaactttcaacaacggatctcttggctctc
>OTU_4297_6
ttaacgagttagggtcttccaggcccgacctcccaaccctatgtttattgaacctctgttgcttcggcgcgcccgtctcacgaccgccggaggagtcgccgcgaggcgccctctggccagcgcccgccgacggccaacccctcaaaaactctgaatgaatcgtgtcttgatgtctaagtctatgattaaattaaagcaaaactttcaacaacggatctcttggttctg
>OTU_4304_24
ttattgaataaacttggttgggttgctgctggctcttaggagcatgtgcacgctcactccattttaaccacctgtgcacatactgtagacctgaatgaaacttttcgaggtaactcggtttgaggactgttgtgaaaatcagctgttcttgcattccaggtctatgtttttaaaaatatactccataacaagtcatagaatgttaataatgggccttctgtgcctttaaattataatacaactttcaacaacggatatctaggctctc
>OTU_4309_11
ttatcgtacaaaatgtgagagaggcatgcaagggctgtcgctgactccaagtcgtgcacgccggagtgtgccctctcacataataatccatctcaccctttgtgcaccaccgcgtgggcaccctccgatctcggagggggctcgcgttttcacacaaaaccccccccctttaaaaagtgtagaatgacctcatttatgcaatcaatacaactttcaacaacggatctcttggctctc
>OTU_4318_23
ttatcgtaaaaccgaggtgcgagggctgtcgctgaccttttttggtcgtgcacgcccgagcgctctcacacaatccatctcaccccttgtgcaccaccgcgtgggttccctttttggcttgtccgaaggggggctcgcgttttcacacaaacttgaattggtgtagaatgtcctcatttatgcaataaatacaactttcaacaacggatctcttggctctc
>OTU_4325_8
ttcataataagtgttttatggcactttttaaatccatatccaccttgtgtgcaatgtcagtcgatcttcttcatggagatcgaccaaacatcaaccttatcttttaactctttgtctgaaaaatattatgaataaaataattcaaaatacaactttcaacaacggatctcttggctctc
>OTU_4342_1
ttacagagttgcaagactccctaaaccatcgtgaacgttacccaaaccgttgcttcggcgggcggcgcccctgcgcgcccccgggccccaccgcgggcgcccgccggaggtcaccgaactcctgataatttatggcctctctgagtattctttactgaataagtcaaaactttcaacaacggatctcttggctctc
>OTU_4350_23
ttaccgaattgtcaacacgagctgttgctggtcctcaaacgggggcatgtgcacgctctgttcacacatccactcacaccctgtgcaccctctgtagttctatggtcagggggcctgtcctcctgctgtggttctgcatctttacacacacacactgtaaccaagtctcatggaatgtacaccgcgtttaacgcaatacaatacaactttcagcaacggatctcttggctctc
>OTU_4399_3
ttacaagaagccgaaaggctacttaaaaccatcgcgaactcatccaagttgcttcggcggcgcggggcccttcaccgggcgccgcggccccgcctctccggaggtatggggcgcccgccggaggtacgaaactctcctgtattatagtggcatctctgagtactaagcaaataagtcaaaactttcaacaacggatctcttggctctg
>OTU_4400_3
ttatcgagttagggtcttctaggcccgacctcccaaccctttgtttactgaaccactgttgcttcggcgggcccgtctcacgaccgccggaggagcgctgaaaagcgtgtcctctggcccgcgtccgccgatggccaaccacaaaactctgtgtaaatcgtgtcatatgtctaagtctatgattaaattaaagcaaaactttcaacaacggatctcttggttctg
>OTU_4401_4
ttacagagaaaatgccctttggggtatatctcccaccctttgtttataataccaattgttgctttggcaggcccgtccaaggaccactggcttctgctggtcagtgcctgccagaggaccccaacactcttttataatttatcgtctgagtactatataatagttaaaactttcaacaacggatctcttggttctg
>OTU_4402_2
ttaccgagttagggtctaaaaaggcccgacctccaaccctttgtttactataccatgttgctttggcgggcccgcctttcggggctgccgggggctttcatgcccatggtcagtgcccgccagtagccttattaaattcttccataattatgttgtctgagtataaacataaaaatcgttaaaactttcaacaacggatctcttggttctg
>OTU_4404_2
ttattataacaatggaggtgctggggttgttgctgacttttgaaatggttgtgcacaccttggtgctctcatatacaatccatctcacccctcttgtgcatcaccacatggaggccccttttagctagttctaaagggggttttcatgtttttatacatacaccattttaatgcaatttgtagaatgtcatacttttgcgaccacatgcaattaatacaactttcaacaatagatctcttggctctc
>OTU_4405_2
ttattgtaacaatggaggtgctggggttgtcgctgacttttgaaatggtcgtgcacacctcggtgctctcatatacaatccatctcacccctcttgtgcatcaccgcgtggaggccccttttggctagttctaaagggggttttcacgtttttatacatacaccattttaatgcaatttgtagaatgtcatacttttgcgaccacatgcaattaatacaactttcaacaatagatctcttggctctc
>OTU_4409_6
ttatcgtacaatggaggtgctggggttgtcgctgacctttgaaagggtcgtgcacgcctcggtgctttcacacaatccatctcaccccttttgtgcatcaccgcgtgggggtcccttttagctagttctgaagggggctttcgcgtttttacaaacacacccttttaatgcaatatgtagaatgtcttactttttgcgatcacacgcaatcaatacaactttcaacaacggatctcttggctctc
>OTU_4423_2
ttaccgagttcatgcccttacgggtagatctcccaccctgtgttatcattacctttgttgctttggcgggccgccaggctccggtcaggctatcggcttcggctggtacgcgcccgccagaggaccccaaactctgaatgttagtgtcgtctgagtactatctaatagttaaaactttcaacaacggatctcttggctctc
>OTU_4436_3
ttaccgagtgagggccctctgggtccaacctcccacccgtgtttatcgtaccttgttgcttcggcgggcccgccacttgtggccgccggggggcttctgccctctggcccgcgcccgccgaagacaccattgaacgctgtctgaagattgcagtctgagcaattagctaaataagttaaaactttcaacaacggatctcttggttccg
>OTU_4440_4
ttattgaataaacctgatggactgttagctggcttttcgaagcatgtgctcgtctgtcatctttatctctccacctgtgcacattttgtagtcttggatacctctcgaggcaactcggattttaggatcgccgtgctgtacaagttggcttttctttcatttccaagactatgtttttatatacaccaaagtatgtttaaagaatgtcatcaataggaacttgtttcctataaaattatacaactttcagcaacggatctcttggttctg
>OTU_4445_9
ttaacgtatctccgacggggtgcggcgagccctgctccggccaaacggccgtgcagcgggttcccgtccccgttcgcaccttacaaacccactgtgaaccttcacggcggacgggaggcgactccccgaggccgcataccgaactccagtcgcaaagaatgtcgtgcggactttgtgccgcctaaaacacgaaacaactttcaacaacggatctcttggctctc
>OTU_4470_7
ttactgagtgcgggccctctgggtccaacctcccacccgtgtttatcgtaccttgttgcttcggcgggcctccgtgcccgccggagacacctttgaacgctgtctgaaggttgcagtctgagtcgattttttccaaatcaatcaaaactttcaacaacggatctcttggttccg
>OTU_4473_1
ttaacgagctagggtcttcttggcccgacctcccaaccctatgtttactgaacctttgttgcttcggcggacccgtctcacgaccgccgggggaccgtaagacgtcctctggcccgtgtccgccggtggcccaactgaacaaattctgattaaaacgtgtcaatgtctgagtagaattcataattaaaacaaaactttcaacaacggatctcttggctctc
>OTU_4474_9
ttaacgagttagggtctctcacggcccgacctccttacctttgtctaccttacctcacgttgcttcggcgggcccgtcctcttttggaccgccggagggttcaccccctctggcccgttgcccgccgacagccccccaaccaaaaaactcttgcaataactgtgaaattgtctgaattttgcttctaaaataaccaaaaactttcaacaacggatctcttggttctg
>OTU_4475_2
ttaccgagtttacaactcccaaacccaatgtgaacgttaccaaactgttgcctcggcgggatctctgccccgggtgcgtcgcagccccggaccaaggcgcccgccggaggaccaaccaaaaactcttttgtataccccctcgcgggttttttatatctgagccatctcggcgcctctcgtaggcgtttcgaaaatgaatcaaaactttcaacaacggatctcttggctctc
>OTU_4498_2
ttattgattgcgaatcgttgccttctgtgctggccagctctggcaagtgcacgtcggtgactttcatccaataccctgtgaacctttggcctctgctagctttggccggcaggggaatttacacacactcgaatgtaatgaaatttattgtcgtgcgcaagcactaatgtacaactttcaacaacggatatctcggctctc
>OTU_4507_1
ttactgagaatgctctccggagccctcaaatcttgcgtacccatctcgtacttgcgggttgctctcgggcgcccgcgaggattcaaactgtttgaccatcgtcagattcttacaaaagaaaacaactttcaacaatggatctcttggctccg
>OTU_4523_3
ttaacgagttagggtctttctcggcccgacctcccaaccctatgtttactgaacctttgttgcttcggcggacccgttcttacgaccgccgggggaccgtaagacgtcctctggcccgtgtccgccggtggcccaactgaacaaattctgattaaaatgtgtcaatgtctgagtagaattcataaaaattaaagcaaaaactttcaacaacggatctcttggttctg
>OTU_4534_8
ttactgaactgtcaacacgagttgttgctggtcctcaaatgggggcatgtgcacgctctgtttacatacccactcacacctgtgcaccctctgtagttctgtggtgtgggggactctgtcctcccgctgtggttctacgtctttacacacacagtttcatagaatgtatgtcgcgtttaacgcaatacaatacaactttcagcaacggatctcttggctctc
>OTU_4563_3
ttatcgtacaagatgtgaggggcatgcaagggctgtcgctgactgaatgtcgtgcacgcccgggtgtgtttcctcacataataatccatctcaccctttgtgcatcaccgcgtgggcaccctttgggatcatctcggagggggctcgcgttttcacacaaacaccccttttaaaaagtgtagaatgacctcatttatgcgctaacccgcaatcaatacaactttcaacaacggatatctaggctctc
>OTU_4565_1
ttaccgagtttacaactcccaaacccaatgtgaaccataccaaactgttgcctcggcggggtcacgccccgggtgcgtcgcagccccggaaccaggcgcccgccggagggaccaaccaaactcttttctgtagtcccctcgcggacgttatttcttacagctctgagcaaaaccataaataaatcaaaactttcaacaacggatctcttggttctg
>OTU_4569_1
ttaacgagttagggtcttctaggcccgacctcccaaccctatgtttattgaacctctgttgcttcggcggacccgcctcacggccgccggaggaccgctgcaaggcgtcctctggccagcgtccgccgatggccaaccacttaaactctgaataaatcgtgtcatgtgtctaagtctatgattaaattaaagcaaaactttcaacaacggatctcttggttctg
>OTU_4573_8
ttaccgagtgagcgccctcgggcgcgacctccaacccattgtgaaacaacctctgttgcctcggggtgacccggcctcgcccgggccccgatggacgatctgccccaactctgcatctttgcgtcggatgtgaaaagtaaattgatttaaaactttcaacaacggatctcttggttctg
>OTU_4590_1
ttaccgagttagggtcttctcaggcccgacctccttacctttgtctaccttacctcacgttgcttcggcgggcccgtcctcttttggaccgccggagggttccaccccctctggcccgttgcccgccgacagcccccaaccaaaaaactcttgcaataactgtggaattgtctgaatcttgcttctaaaataaccaaaaactttcaacaacggatctcttggctctc
>OTU_4591_1
ttaccgagtgagggccctctgggtccaacctcccacccgtgtttatcgtaccttgttgcttcggcgggcccgcctcacggccgccggagacacctttgaacgctgtctgaaggttgcagtctgagtcgattttttaaatcattaaaactttcaacaacggatctcttggttccg
>OTU_4595_2
ttaccgaattgtcaacaagagttgttgctggcctccaaacgggggcacgtgcacgctctgtttacacatccactcacacctgtgcaccctctgtagtcctatggttcggaagaccccgtcttccttctgtagctctacgtctttacacacacactgtagcgatgtctcatggaatgtttttatgcgtttaacgcgatacaatacaactttcagcaacggatctcttggctctc
>OTU_4601_1
ttacagagttgcaaaactccctaaaccattgtgaacgttacctaaaccgttgcttcggcgggcggcccgggtccttcccggcgccccccggccctcgcgggcgcccgccggaggtaaaccaaactattgcattaatggcctctctgagtcttctgtactgaataagtcaaaactttcaacaacggatctcttggttctg
>OTU_4610_1
ttacagagttgtaaaactcccaaacccatgtgaacatacctgttgcctcggtggcctacccggtagctaccctgtagctaccctgtagtccgccgacggaacttaaaactcttgttttcaattgtcatttctgagaataaaacaaataagttaaaactttcaacaacggatctcttggttctg
>OTU_4612_1
ttacagagttgcaaaactcccaaccattgtgaacctacctcaccgttgcttcggcgggtggcccggggagacccgagccgcgccggcccccctcggggagccggcaacccgtcagaggccccgaaaccctagcatattggcctctctgagtaacttatacaataagtcaaaactttcaacaacggatctcttggttctg
>OTU_4613_7
ttaaagagtaccggagctctcgggttcctactcccaccctatgatgactttaaatgttgctttggcggaccggcaatattgccaccggcctaggctggatagcgtccgccagaggatttttaaattcctttgactgaattctgagtcttgaaaattgaatcaaaactttcaacaacggatctcttggttctg
>OTU_4614_2
ttaccgagtgagggcccccggcccgacctccaaccctttgttgaccacacccgttgcctcgggggcgacccggacgccgacgcgtcggccgtcgggcaccccggtggaccattccaactctgttgtccgtacgtcggagtactttgcgaatcaatcaaaactttcaacaacggatctcttggttctg
>OTU_4630_1
ttcataataagtgttctactgcactttttaaacaagtactcaccttgtgtgcaatgtttgctgtcgagtcgtttgttcgtctcgacagtataataccaaccttttattgtaaattttttgtctgaagctatttcgaatatataattctaaatacaactttcaacaacggatctcttggctctc
>OTU_4639_22
ttaatgattgcgaatcgtcgccgtcagtgctggccggcttcggcaagtgcacgtcggtgactttcatccaataccctgtgcacctttggcctcttgctagcttcggccggcagaggatttttatacacacactcgaatgtaatgaaaactattgtcgtgcgcaagcactaatgtacaactttcaacaacggatctcttggctctc
>OTU_4641_19
ttaacgagttagggtcttctcggcccgacctccccaccctatgtttgctgaacctttgttgcttcggcggacccgtctcacgaccgccgggggaccgtaagacgtcctctggcccgtgtccgccggtggccaaaccgaacaaattctgattaaaatgtgtcaatgtctgagtagaattcataattaaaacaaaactttcaacaacggatctcttggttctg
>OTU_4643_12
ttacagagttcatgcccagtagggtagatctcccaccctgtgttgacatatacgtttgttgctttggcaagcccgctgggctttggttcggccaccggcttcggctggtgagcgcttgtcagaggaccctaaaactcttgaatgttagtatcgtctaagcaccatatgaatagttaaaactttcaacaacggatctcttggttctg
>OTU_4655_10
ttaccgagtgagggccctctgggtccaacctcccacccgtgtttatcgtaccttgttgcttcggcgggcccgccacttgtggccgctggggggcacctgcccccgggcccgcgcccgccgaagacaccattgaacgctgtctgaagaatgcagtctgagcttcttagctaaattagttaaaactttcaacaacggatctcttggttccg
>OTU_4664_1
ttaccgagtttacaactcccaaacccttatgtgaaccgtacctatcgttgcttcggcgggaacgccccggcgccctgcgcccggatccaggcgcccgccggagaccccaaactcttgtgtttttttcagtattctctgagtggcaaacgcaaaaataaatcaaaacttttaacaacggatctcttggctctg
>OTU_4672_3
ttaccatggcgcagaaggtggtggacatactggtaaccccaggagttaatcccttcacgctcttctacccttgtctatttgcacctttgtcgttccctcggcgggtcttgaacctgccggcggcaactttcaaaacctcttgcagttagtatctaattcttctgattcaatatcaaattattacaactttcaacaatggatctcttggttctg
>OTU_4712_17
ttgatgaaacgcgaggacgctctctttagaggtccgacctaattcatttccttcaaccctgtgcacatactacttttttttacaccttttttgacgcattagttttaagaatgtaatcaagtctcttaattgagcacaaatataaacaaaactttcagcaacggatctcttggctctc
>OTU_4717_1
ttacagagttgcaagactcccaaaccactgtgaacatacccgtaccgttgcctcggcgggcggccccagggcggggctgcagcctcctcgcggaggcgcccgccgcaggtcgcacaactatactattcattggcccctctgagtacgctccaaacaatcaaaactttcaacaacggatctcttggttctg
>OTU_4728_1
ttacagagttgcaaaactcccaaaccactgtgaacatacccgtaccgttgcctcggcgggcggccccagggcggggccgcagcctcctagcggaggcgcccgccgcaggctacagcaactatacttatccactggcccctctgagtacaccacaaacaattaaaactttcaacaacggatctcttggttctg
>OTU_4729_8
ttaccgagagagggcgccccgcgcgcccgacctcccaaccctttgtcgaccgtaccgcgttgcctcggggtggcctttcacgggcgccccagaggacgtcaaccaaaactctgcgtctttgcgtcgagtcttttatattgaatcaatcaaaactttcaacaacggatctcttggttctg
>OTU_4738_2
ttaccgagtgagggccctctgggtccaacctcccacccgtgtttatcgtaccttgttgcttcggcgggcccgccgcaaggccgccggggggcttccgtccccgggtccgcgcccgccgaagacacctgtgaacgctgtatgaagattgcagtctgagcgaaaagctaaataagttaaaactttcaacaacggatctcttggttccg
>OTU_4744_2
ttaccgagttagggtctttcaggcccgacctccaaccctttgttgactataccatgttgctttggtgggcccgcctttagatgccgctggggactttcacatccctggtcagtgcccgccagtagccttcttaaattctattataattatgttgtctgagtataaacataaaaatcgttaaaactttcaacaacggatctcttggttctg
>OTU_4751_1
ttactagggggcttcggccccatcaagataccaccccttgcattttgagtaccttctgtttcctcggcgggcctgcccgccaacggggaccacaccaaacccttctgtagtcgcagtaaacgtctaaaaacaataaatatcaaaactttcaacaacggatctcttggttctg
>OTU_4758_3
ttattgtaacaatggaggtgctggggttgtcactgacttttgaaatggtcgtgcataccttggtgctctcatatacaatccatctcacccctcttgtgcatcactgcatggaggccccttttggctagttctaaagggggttttcacatttttatacatacaccattttaatgcaatgcgtagaatgtcatacttttgcgatcacacgcaattaatacaactttcaacaacggatctcttggctctc
>OTU_4759_1
ttattgattgcgaatcgttgtctccagtgctggccaggtcactctggcaagtgcacggagacagctttcatccaataccctgtgaacctttggcctcttgctagcttcggcgagcagaggattttacacgcactcgaatgtaatgaaattattgttgtgcacaagcactaatgtacaactttcaacaacggatctcttggctctc
>OTU_4770_4
ttacagagttctcgccctcgcgggtagatctcccacccactgtgattgctaccgtgttgctttggcgggctgctgggcctagcctggccgccggctccggccggcgcgcgcccgccagaggcccgacaaattctgattgtcagtgtcgtctgagtactatataatagttaaaaaactttcaacaacggatctcttggttctg
>OTU_4773_5
ttaccgagtttacaactcccaaacccctgtgaacataccacttgttgcctcggcggatcagcccgctcccggtaaaacgggacggcccgccagaggacccctaaactctgtttctatatgtaacttctgagtaaaaattcaaaatgaatcaaaactttcaacaacggatctcttggttctg
>OTU_4782_3
ttactgaattgttaacaagagttgttgctggtcctcaaatgggggcatgtgcacgctctgtttacacatccactcacacctgtgcaccctctgtagttctatggcctggggggctctgtcctcctgctgtggctctacgtctttacacacacactgtaacaaagtctcatggaatgtatgtcgcgtttaacgcaatgaaatacaactttcagcaacggatctcttggctctc
>OTU_4790_5
ttactgagagtggtaggctctgcctgcgcatctcttacccgtgtcttttgcgtactctttttgtttccttggtgggcatgcccgccaacaggactctttaaaacccctttgcaattgcagtcagcgtctgataaacatactagttacaactttcaacaacggatctcttggttctg
>OTU_4792_1
ttactgaattgtcaacaagagttgttgctggtccccagatgggggcatgtgcacgctctgttaacacatccactcacaccctgtgcaccctctgtagttctatggtcagggggcctgtcctcctgctgtggttctgcatctttacacacacacactgtaacaaagtctcatggaatgcatgccgcgtttaacgcaatataatacaactttcagcaacggatctcttggctctc
>OTU_4826_3
ttaaagagttagggtcctctgggcccgacctcccaaccctctgtttaccgaacctctgttgcttcggcggacccgtctcacgaccgccggaggaccgccccccaaggcgtcctctggccagcgtccgccgatagccaacctccaaaactcctgaataaatcatgttatatgtctaagtcttatgattcaaatcaataaagcaaaactttcaacaacggatctcttggttctg
>OTU_4829_3
ttaaagagtaccggagctctcgggttcctactcccaccctatgttgactttaaatgttgctttggcggaccggcgatctcgccactggcctaggctggacagcgtccgccagaggatttttaaattcctttgactgtgaactccgagtctttgaaaattgaatcaaaactttcaacaacggatctcttggttctg
>OTU_4843_1
ttacagagttgcaaaactcctaaaccattgtgaatattaccgtaactattgcttcggcaggtagttcccactagggttctgcatctccccctcggggcggataacctgccgaagttattttaactctcgattttaattggtatctctgagtaacttataaaataagtcaaaactttcaacaacggatctcttggttctg
>OTU_4849_12
ttaatgattgcgaatcgtcgccttcagtgctggccggcttcggcaagtgcacgtcggtggctttcatccaataccctgtgcacctttggcctcttgctagcttcggccggcagaggatttttacacacactcgaatgtaatgaaatttattgtcgtgcgcaagcactaatgtacaactttcaacaacggatctcttggttctg
>OTU_4854_2
ttaccgagtaacggggcttccagcctcgactcccacccaatgttgactttacgatggttgctttggcggaccggtggcttcccgccgccgccggccctcgagctggagagcgtccgccagaggattttccaaaactcctgttaaaccgtggaattctgagtttgcttgcaaatgaatcaaaactttcaacaacggatctcttggttctg
>OTU_4859_7
ttaacgagttagggtcttctcggcccgacctcccaaccctttgtttactgaacctttgttgcttcggcggacccgtctcacgaccgccgggggaccgtaagacgtcctctggcccgtgtccgccggtggccaaaccgaacaaattctgattaaaattgtcaatgtctgagtagtattcataattaaaacaaaactttcaacaacggatctcttggttctg
>OTU_4868_20
ttactgaactgtcgacacgagttgttgctggtcctcaaatgggggcatgtgcacgctctgtttgcatatccactcacacctgtgcaccctatgtagttctatggcttgggggaccctgtcctcctgctgtggttctatatctttacacacactctgcaataaagtcttatggaatgtataccgcgtttaacgcaatacaatacaactctcggcaacggatatctaggctctc
>OTU_4869_1
ttaccgagtttacaactcccaaacccctgtgaacataccacttgttgcctcggcggatcagcccgctcccggtaaaacgggacggcccgccagaggacccctaaactctgtttctatacagtatcttctgagtaaatgattaaataaatcaaaactttcaacaacggatctcttggttctg
>OTU_4875_3
ttaccgaggcccccgcgcctctgctcccaccctgtgttgacctgcaatgttgctttggcgggccggcgggtacacccgccgccgggccgcgcctggagagcgcccgccagagaaccacgcgaaactctttgcaactttgagcgtctgagcactttgagaattgaccaaaactttcaacaacggatctcttggttctg
>OTU_4881_4
ttaaagagttagggtcttctaggcccgacctcccaaccctttgtttattgaacctctgttgcttcggcggacccgcctcacggccgccggaggaccgctgaaaagcgtcctctggccagcgtctgtcgacagccaaccacttaaactctgaatgaatcgtgttataattgtctaagtttatcataaaaattaaagcgaaaactttcaacaacggatctcttggttctg
>OTU_4886_3
ttaccgagttcatgcccttacgggtagatctcccaccctgtgttatcattacctttgttgctttggcgggccgtcaggcttcggtcaggctaccggctccggctggtacgcgcccgccagaggaccccaaactctgaatattagtgtcgtctgagtactatctaatagttaaaactttcaacaacggatctcttggttctg
>OTU_4902_15
ttatcgtacaatgggggtacgagggttgtcgctgcctttatgtcgtgcacgcccgagtgctctcacatacaaatatccatctcacccctttgtgcatcaccgcgtgcgtccccccttcctcggaggggggtgctcacgtttttaaacatcaaacgccctagtgtagaatgttctttgcgcaatcacgcgctattaatacaactttcaacaacggatctcttggctctc
>OTU_4915_6
ttaccgagtgagggccctctgggtccaacctcccacccgtgtttatcgtaccttgttgcttcggcgggcccgccacttgtggccgccggggggcacctgcccccgggcccgcgcccgccgaagacacctgtgaacactgtctgaagttgcagtctgagaaactagctaaattagttaaaactttcaacaacggatctcttggttccg
>OTU_4916_4
ttatcgtacaaccgaggtgctggggttgtcgctgacttttgaaatggtcgtgcacacctcggtgctctcatatacaatccatctcacccctcttgtgcatcaccgcgtggaggccccttttggctagttctaaagggggttttcacgtttttatacatacaccattttaatgcaatgcgtagaatgtcatacttttgcgatcacacgcaattaatacaactttcaacaacggatctcttggctctc
>OTU_4926_10
ttaatgattgcgaatggctgccttcagtgctggctcttatgagcatgtgcacgttggtggctttcatccaatacccatgtgaacctttggcctcttgctagctttggccggcagaggatttttatacacacactcgaatgtaatgaaaactattgtcgtgcgcaagcactaatgtacaactttcaacaacggatctcttggttctg
>OTU_4929_19
ttaaagagttagggtcttctaggcccgatctcccaaccctttgtttattgaacctctgttgcttcggcggacccgcctcacggccgctggaggaccgctgaaaggcgtcctctggccagcgtccgccgatagccaaccacttaaactctgaataaatcgtgtcatatgtctaagtttatgattaaattaaagcaaaactttcaacaacggatctcttggctctc
>OTU_4934_17
ttatcgtacaaaatgtgagagaggcatgcaagggctgtcgctgactccaagtcgtgcacgccggagtgtgccctctcacataataatccatctcaccctttgtgcaccaccgcgtgggcaccctttgggatcagactgatctcggaggatgctcgcgttttcacacaaaaccccccctttaaaaagtgtagaatgacctcatttatgcaatcaatacaactttcaacaacggatctcttggctctc
>OTU_4935_12
ttaaagagtaccggagctctcgggttcctactcccaccctatgatgactttaaatgttgctttggcggaccggcaatattgccaccggcctaggctggatagcgtccgccagaggatctttaaattccttcgaatgtgaattccgagtctttgaaaattgaatcaaaactttcaacaacggatctcttggttctg
>OTU_4961_13
ttgctggaacgcgccccaggcgcacccagaaaccctttgtgaacttataccttactgttgcctcggcgcaggccggcctcactgaggcccctcggaaacgaggagcagcccgccggcggccaaccaaactcttgtttcttagtgaatctctgagtaaaaaacataaatgaatcaaaactttcaacaacggatctcttggttctg
>OTU_4970_17
ttacagagttgcaagactccctaaacccactgtaaacctacctatattgttgcttcggcgggcggccccagggcggggtccaggcctgttacaggtgcccgccgacggccgtcgcaaaactcggttgttatatttataggtgctctgagtcaaatgacaaaatcagtcaaaactttcaacaacggatctcttggttctg
>OTU_4972_2
ttactgagtgcgggccctctgggtccaacctcccacccgtgtttaacgaaccttgttgcttcggcgggcccgcctcacggccgccggggggcatccgcccccgggcccgcgcccgccgaagacacctgtgaacactgtctgaagttgcagtctgagaaactagctaaattagttaaaactttcaacaacggatctcttggttccg
>OTU_4996_18
ttaccgaacgtcgacacgagttgttgctggtcctcaaagcaaggggggcatgtgcacgctctgttcacacatccactcacacctgtgcaccctccgtagttctatggccttgggggcctctgtcccctttgcccacggttctacgtctttacacatacaccgtaacaaagtctcatggaatgtatgcagcgtttaacgcaatacaatacaactttcagcaacggatctcttggctctc
>OTU_4998_2
ttaccgagtttacaactcccaaacccctgtgaacataccatttgttgcctcggcggtgcctgcttcggcagcccgccagaggacccaaacccttgattttatacagtatcttctgagtggaattttaaataaatcaaaactttcaacaacggatctcttggctctg
>OTU_5023_1
ttaccgagttcatgcccttacgggtagatctcccaccctgtgttatcattacctttgttgctttggcgggccgccaggctccggtcaggctatcggcttcggctggtaagcgcccgccagaggacccaatattctgattatcagtgtcgtctgagtactatgataatagttaaaactttcaacaacggatctcttggttctg
>OTU_5028_1
ttaccttataccatcggagggccggccatggactgcggatccttggggaacgcttccgtgcccgccctgtctgattctacccatgatttttgcgtactcattgtttcctcggtaggcttgcctgccgataggacattatacaaccctttgtaattgcagtcagcgtcagaaaaaaacttaatagttacaactttcaacaacggatctcttggttctg
>OTU_5036_1
ttattgaataaacctgatggactgttagctggcttttcgaagcatgtgctcgtctgtcatctttatctctccacctgtgcacattttgtagtcttggatacctctcgaggaaactcggattttaggatcgctgtgctgtacaagtcggcttttctttcatttccaagactatgtttttatatatacaccaaagtatgtttatagaatgtcatcaatgggaacttgtttcctataaaattatacaactttcagcaacggatctcttggctctc
>OTU_5046_1
ttactgaattgtcaacaagagttgttgctggtccccagatgggggcatgtgcacgctctgttaacacatccactcacaccctgtgcaccctctgtagttctatggttggggggggacctgtcccctcctgctgtggctctgcatctttacatacacactgtaacaaagtctaatggaatgcatgccgcgtctaacgcaataaaatacaactttcagcaacggatctcttggttctg
>OTU_5068_3
ttaccgagtgagggccctctgggtccaacctcccacccgtgtttaacgaaccttgttgcttcggcgggcccgcctcacggccgccggggggcatccgcccccgggcccgcgcccgccgaagacacctgtgaacactgtctgaagttgcagtctgagcaattagctaaataagttaaaactttcaacaacggatctcttggttccg
>OTU_5070_1
ttactgagttagggttctttcagagcccgatctccaaccctttgttaaattactttgttgctttggcaggcccgtctttcgggaccgccgggggggttaggtcacctctggtcagtgtttgccagtagccaatttaaattatttttaaccatgtcgtctgaatcttaattaaaatttaattaaaactttcaacaacggatctcttggttctg
>OTU_5077_2
ttatcgtacaaaatgtgtgaggcatgcgagggctgtagctgactcaaagttgtgcacgccggagtgtgtcctctcacataacaatccatctcaccctttgtgcaccaccgcgtgggcaccctccgatctcggagggggctcgcgttttcacacaaacaccccttttaaaaagtgtagaatgacctcatttatgcgctaacccgcaatcaatacaactttcaacaacggatctcttggctctc
>OTU_5078_11
ttacagagttcatgcccttcggggtagatctcccacccttgtgtatcattatagaatgttgctttggcgggccgcgtgcccgccagaggacccctaaactctgaatattaatgtcgtctgagtactattcaatagttaaaactttcaacaacggatctcttggttctg
>OTU_5079_2
ttaaagagtaccggagctctcgggttcctacttccaccctatgttgactttaaatgttgctttggcggaccggcgatctcgccaccggcctaggctggacagcgtctgccggaggatttttatattcctttgactgaattctgagtctttgaaaattgaatcaaaactttcaacaacggatctcttggttctg
>OTU_5090_3
ttaaagagtaccggagctctcgggttcctactcccaccctatgttgactttaaatgttgctttggcggaccggcaatcttgccactggcctagggctagacagcgtccgccagaggatttttaaattcttttgactgaatttctgagtctttgaaaattgaatcaaaactttcaacaacggatctcttggctctc
>OTU_5096_2
ttaccgagttcatgccctttagggtatatctcccaccctttgtttacaataccattgttgctttggtgggcccgtcatatgaccaccggctttggctggtttgtgcctgccagaggaccccaaaactctttattatgtcgtctgagtactatgtaatagttaaaaaactttcaacaacggatctcttggttctg
>OTU_5100_5
ttaccgaattgtcaacacgagttgttgctggtcctcaaacggggacatgtgcacgctctgtttacacatccactcacacctgtgcaccctccatagttctgcagcctgggggctctgtccccctgatgcggttctatgtatttacacacacacacactgtagtaaagtctcatggaatgcataccgcgtttaacgcaatataatacaactttcagcaacggatctcttggctctc
>OTU_5111_5
ttatagagttcttgcccagtagggtagatctcccaccctgtgttgatatatacgtctgttgctttggcaagcccgctgggctttggttcggccaccggcttcggctggtgagcgcttgtcagaggaccctaaaacccttgaatgttagtatcgtctaagcaccatatgaatagttaaaactttcaacaacggatctcttggttctg
>OTU_5114_7
ttaccgagcgagcgcctccgggcgcgacctccaaccctttgcgaaccaacctctgttgcctcggggtgacccgggcccgcccggcccccgaaggacaatatgacaactctgcatctttgcgtcggatatgaaaaggaattcgaatgaaaactttcaacaacggatctcttggttctg
>OTU_5119_2
ttaccgagtgcgggccctcgcggggcccaacctcccacccgtgtctaccgtcacctgttgcttcggcgggcccgccttcgtgccgccggggggccctctgcgcccccgggcccgcgcccgccgaagaccctaggaacactggatgaaggatgccgtctgagtcaacgacacaatcgttaaaactttcaacaacggatctcttggttccg
>OTU_5130_2
ttagtgaacgcccttttgggcttataactatccaaacctctgtgaaccgtgcccttcggggctattttacaaacatggtgtaatgaacgtcatatatcataacaaaacaaaactttcaacaacggatctcttggctctc
>OTU_5131_1
ttaccgagttttcaactcccaaacccctgtgaacataccatgttgcttcggcggatcaccccaagcccctcggggcgcggggcccgccagaggacccaaaactcaactgtattttttgtatcaaaacgtattctgagtggaattttaaataaatcaaaactttcaacaacggatatctaggctctc
>OTU_5132_1
ttactgaattgttaacaagagttgttgctggtccctatacgggggcatgtgcacgctctgtttacacatccattcacacctgtgcactctctgtagttctgtggtttgggggctctgtcctcctgctgtggctctacgtctttacacacacactgtaacaaagtctcatggaatgtatgtcgcgtttaacgcaatgaaatacaactttcagcaacggatctcttggctctc
>OTU_5135_14
ttaacgagttagggtctttctcggcccgacctcccaaccctatgtttactgaacctttgttgcttcggcggacccgttcttacgaccgccgggggaccgtaagacgtcctctggcccgtgtccgccggtggccaaaccgaacaaattctgattaaaatgtgtcaatgtctgagtagaattcataattaaaacaaaactttcaacaacggatctcttggctctc
>OTU_5147_1
ttaccgagtttacaactcccaaaccctatgtgaaccttaccttttgttgcttcggcgggatcgccctggcgcttcacggcgccggatctcaggcgcccgccggaggcccaaaactcttgtttatttatgtttcctctccgagtggcacaagcaaaataaatcaaaactttcaacaacggatctcttggttctg
>OTU_5184_1
ttaccgagtttacaactcccaaacccctgtgaacataccttactgttgcctcggcggatcagcccgctcccggtaaaacgggacggcccgccagaggacccctaaactctgtttctatatgtaacttctgagtaaaaccataaataaatcaaaactttcaacaacggatatctcggctctc
>OTU_5187_1
ttaccgagtgcgggccctcgcggcccaacctcccacccttgtctctatacacctgttgctttggcgggcccaccggggccacctggtcgccgggggacgcacgtccccgggcccgcgcccgccgaagcgctctgtgaaccctgatgaagatgggctgtctgagtactatgaaaattgtcaaaactttcaacaatggatctcttggttccg
>OTU_5218_1
ttgtcgattgcgaatcgttgtctccagtgctggccaggtcactctggcaagtgcacgtcgacagctttcatccaataccctgtgaacctttggcctcttgctagcttcggcgagcagaggattttacacccactcgaatgtaatgaaattattgttgtgcacaagcactactatacaactttcaacaacggatctcttggctctc
>OTU_5219_4
ttactgagttagggtcttccaggcccgacctccaaccctttgcctaccttacctcttgttgcttcggccggcccgtcccccctagaaataggggtcgaccgccggagggctcacaccctctggtccgcgcccgccgatggccctcaaaccaaaactcttgttcaatcgtgaattgtctgagtatacaaaacaaaataaaccaaaaactttcaacaacggatatctaggctctc
>OTU_5224_17
ttactgattgcgaatcgttgccttcagtgctggccggcttcggcaagtgcacgttggtgactttcatccaataccctgtgaacctttggcctcttgctagcttcggctggcagaggattttacatactcgaatgtaatgaaatttattgtcgtgcgcaagcactaatgtacaactttcaacaacggatctcttggctctc
>OTU_5226_15
ttactgaactgtcaacacgagttgttgctggtcctcaaatgggggcatgtgcacgctctgtttacatacccactcacacctgtgcaccctctgtagttctgtggtgtgggggactctgtcctcccgctgtggttctacgtctttacacacacactgtaacaaagtctcatggaatgtatgtcgcgtttaacgcaatacaatacaactttcagcaacggatctcttggctctc
>OTU_5228_9
ttaacgttggggactaaccatccctcagcgagatagaacccttgcctttcaagtaccacacgtttcctcggcaggtccgcctgccaatggggaccacaacaaaaccttttgtaatagcagtaaacgtctaaaaaacaaaaatttaaaactttcaacaacggatctcttggttctg
>OTU_5240_1
ttacaaaatgtatgggatgccctatcggactcccaagcaaaacacattcctgtgtactctcccccattaaacatttgaaccaattagtagtctgagaaggccatgtgccgtaaaatttaaacatgttaaaactttcaacaacggatatctaggctctc
>OTU_5256_2
ttaccgagtactacactcataaccctttgtgaacctttatacctgttgcttcggcggcgcgcctcccggggcgtgcccgccggcattatcagaatctctgttcgaacccgacgatacttctgagtgttctaagcgaactgttaaaactttcaacaacggatctcttggctcca
>OTU_5265_16
ttatcgtacaaaatgtgagagaggcatgcaagggctgtcgctgactccaagtcgtgcacgccggagtgtgccctctcacataataatccatctcaccctttgtgcaccaccgcgtgggcaccctccgatctcggagggggctcgcgttttcacacaaaaccccccctttaaaaagtgtagaatgacctcatttatgcaatcaatacaactttcaacaacggatctcttggctctc
>OTU_5272_4
ttaccgagtgagggcccccggcccgacctccaacccattgttgaccaacacctgttgcctcgggggcgacccggacgccgacgcgtcgggcaccccgacggaccattccaactctgtgtccctacgtcggagtactttgatgaatcaatcaaaactttcaacaacggatctcttggttctg
>OTU_5284_1
[truncated: 264,646 more chars]
