# Supplementary material for: Habitat- and soil-related drivers of the root-associated fungal community of Quercus suber in the Northern Moroccan forest
Source: PLoS One. 2017 Nov 20;12(11):e0187758. doi: 10.1371/journal.pone.0187758 (PMC5695781; doi:10.1371/journal.pone.0187758)
Supplement: S2 Table — (DOCX) [file pone.0187758.s003.docx]

**Table S2.** Variation in *Quercus suber* root-associated fungal community structures among Moroccan cork oak habitats

| Model/Factors | | Df | SS | MS | | F. Model | | R^2^ | | P value ^1^ | |  |
| --- | --- | --- | --- | --- | --- | --- | --- | --- | --- | --- | --- | --- |
| Pairwise PERMANOVA | |  |  |  | |  | |  | |  | |  |
| Maâmora-Benslimane | | 1 | 0.9597 | 0.95969 | | 2.6651 | | 0.14278 | | 0.001 | |  |
| Maâmora-Chefchaoun | | 1 | 0.8114 | 0.81136 | | 2.4208 | | 0.13142 | | 0.001 | |  |
| Benslimane-Chefchaoun | | 1 | 0.7896 | 0.78962 | | 0.11316 | | 0.11316 | | 0.002 | |  |
|  |  | |  | |  | |  | |  | |  | |

Df, degrees of freedom ; SS, sum of squares ; MS, mean squares; F.Model, F value by permutation

^1^ The significance of multivariate analysis of variance and dispersion was assessed with permutational test (Iterations = 999)
