# Supplementary material for: Habitat- and soil-related drivers of the root-associated fungal community of Quercus suber in the Northern Moroccan forest
Source: PLoS One. 2017 Nov 20;12(11):e0187758. doi: 10.1371/journal.pone.0187758 (PMC5695781; doi:10.1371/journal.pone.0187758)
Supplement: S3 Table — (DOCX) [file pone.0187758.s004.docx]

**Table S3.** Fungal indicators of Moroccan cork oak habitats (Maâmora, Benslimane and Chefchaoun)

| Fungal OTU (Taxonomic assignment) | Maâmora ^1^ (40) | Benslimane (21) | | Chefchaoun (49) |
| --- | --- | --- | --- | --- |
| **72**-58 **(Russula sp)**, **246 (Cladophialophora sp)**, **366-407**-141 **(Sordariomycetes sp)**,  **75 (Mycosphaerellaceae sp)**, **105 (Oidiodendron maius)**, 16-138 (Russulaceae sp),  46-97 (Herpotrichiellaceae sp), 243 (Capnodiales sp) | ***^2^ | |  |  |
| **33-1241 (Cladophialophora sp)**, **1937 (Chaetosphaeriales sp)**,  **82 (Aspergillus amstelodami)**, **236 (Archaeorhizomyces sp)**, **999 (Oidiodendron sp)**,  593 (Herpotrichiellaceae sp), 1674 (Fungi sp), 403 (Valsaceae sp), 550 (Rasamsonia sp), 359 (Cryptosporiopsis sp) | ** | |  |  |
| 2013 (Sordariomycetes sp), 2073 (Hansfordia sp), 1064 (Penicillium adametzii),  277-242-92 (Russulaceae sp), 198 (Chaetothyriales sp), 1577 (Archaeorhizomyces sp),  106 (Penicillium nodositatum), 656 (Dothideomycetes sp), 453 (Oidiodendron griseum), 351 (Cladophialophora sp), 405-1188 (Herpotrichiellaceae sp), 784 (Lactarius sp),  318 (Trechispora sp) | * | |  |  |
| Fungal OTU (Taxonomic assignment) | Maâmora ^1^ (40) | | Benslimane (21) | Chefchaoun (49) |
| **759-69 (Ilyonectria mors-panacis)**, **1190 (Dothideomycetes sp),** 17 (Tomentella atramentaria), 66 (Tomentella sp) |  | | ** |  |
| 67-358 (Tomentella sp), 928 (Sordariales sp), 187-582 (Ascomycota sp),  949 (Ilyonectria mors-panacis), 36 (Ilyonectria estremocensis),  1114 (Mortierella amoeboidea), 184-98-989 (Cortinarius sp),  536-390 (Tomentella atramentaria), 28 (Saccharicola sp), 38 (Thelephoraceae sp),  315 (Inocybe sp) |  | | * |  |
| **32-362 (Cenococcum geophilum)**, **292 (Capnodiales sp)**, **112 (Cladophialophora sp)**, **492 (Chaetothyriales sp)**, **78 (Dothideomycetes sp)**, **572 (Sebacinales Group B sp)**, **95 (Cryptosporiopsis brunnea)** |  | |  | *** |
| **1194 (Cladophialophora sp)**, **56 (Cenococcum geophilum)**, **610 (Sarcoleotia globosa)**, **254**-59-413 **(Cryptosporiopsis brunnea)**, **1137 (Cladophialophora chaetospira)**, 51 (Hygrophorus cossus), 298 (Tuber sp), 369 (Helotiales sp) |  | |  | ** |
| Fungal OTU (Taxonomic assignment) | Maâmora ^1^ (40) | | Benslimane (21) | Chefchaoun (49) |
| 2349-271 (Helotiales sp), 3714-2720 (Cryptosporiopsis brunnea), 127 (Sebacinaceae sp), 551 (Pezizales sp), 1197-3556-526-646 (Chaetothyriales sp), 704 (Degelia plumbea), 3266-1008-467-471 (Cenococcum geophilum), 177 (Penicillium restrictum), 2844 (Saccharomycetales sp), 1677 (Hygrophorus cossus), 1633 (Penicillium malmesburiense), 321-445 (Fungi sp), 100 (Russula odorata), 596 (Humicola nigrescens), 2866 (Pleosporales sp), 741 (Sebacinales Group B sp), 372 ( Sarcoleotia globosa), 493 (Archaeorhizomyces sp), 1487 ( Leotiomycetes sp), 444 (Cladophialophora sp), 171-37 (Sebacinaceae sp) |  | |  | * |

^2^ The corrected Pearson’s phi coefficient of association (“r.g”) was used as model to determine indicator OTUs. The total number of indicator OTUs is indicated between brackets. For each line, OTU are sorted by decreasing r.g values.

^3^ ‘***’ corresponds to *P* < 0.001; ‘**’ *P* < 0.01; ‘*’ *P* < 0.05; ‘NS’ *P* > 0.05. OTUs with a r.g > 0.5 and *P* < 0.01 are indicated in bold.
