# Supplementary material for: Habitat- and soil-related drivers of the root-associated fungal community of Quercus suber in the Northern Moroccan forest
Source: PLoS One. 2017 Nov 20;12(11):e0187758. doi: 10.1371/journal.pone.0187758 (PMC5695781; doi:10.1371/journal.pone.0187758)
Supplement: S6 Table — (DOCX) [file pone.0187758.s007.docx]

**Table S6.** Fungal OTU indicators with respect to soil properties (pH, C:N ratio, available P)

| Fungal OTUs (taxonomic assignment) | A | B | | IndVal ^2^ | |
| --- | --- | --- | --- | --- | --- |
| **Soil acidity**^1^ |  |  | |  | |
| Very strongly acid (pH < 5.0) |  |  | |  | |
| 97 (Herpotrichiellaceae sp.) | 0.8902 | | 1.0000 | | 0.943* |
| **1188 (Herpotrichiellaceae sp.)** | 1.0000 | | 0.6667 | | 0.816** |
| 1674 (unidentified fungi) | 0.8421 | | 0.6667 | | 0.749* |
| Strongly acid (pH: 5.0 – 5.5) |  | |  | |  |
| **3363 (Cladophialophora sp.)** | 1.0000 | | 0.6667 | | 0.816** |
| 409 (Cenococcum sp.) | 0.9333 | | 0.6667 | | 0.789* |
| Moderately acid (pH: 5.6 – 6.0) |  | |  | |  |
| **51 (Hygrophorus cossus)** | 0.9938 | | 1.0000 | | 0.997** |
| 490 (Oidiodendron chlamydosporicum) | 0.9506 | | 0.8333 | | 0.890* |
| 364 (Hydnotrya cerebriformis) | 0.9375 | | 0.8333 | | 0.884* |
| **Table S7.** Continued |  | |  | |  |
| Fungal OTUs (taxonomic assignment) | A | | B | | IndVal ^2^ |
| 572 (Sebacinales Group B) | 0.8772 | | 0.8333 | | 0.855* |
| 493 (Archaeorhizomyces sp) | 0.9889 | | 0.6667 | | 0.812* |
| 650 (Chaetothyriales sp) | 0.8000 | | 0.6667 | | 0.730* |
| Slightly acid (pH: 6.1 – 6.5) |  | |  | |  |
| **928 (Sordariales sp)** | 1.0000 | | 0.6667 | | 0.816** |
| Neutral (pH > 6.5) |  | |  | |  |
| **315 (Inocybe sp)** | 0.9944 | | 1.0000 | | 0.997** |
| **85 (Tomentella sp)** | 0.9799 | | 1.0000 | | 0.990** |
| **45 (Lophiostoma cf cynaroidis)** | 0.9655 | | 1.0000 | | 0.983*** |
| **705 (Sebacina sp)** | 0.9632 | | 1.0000 | | 0.981** |
| 306 (Tuber sp) | 0.9529 | | 1.0000 | | 0.976* |
| 66 (Tomentella sp) | 0.9437 | | 1.0000 | | 0.971* |
| **19070 (Paecilomyces sp)** | 1.0000 | | 0.6667 | | 0.816** |
| **Table S7.** Continued |  | |  | |  |
| Fungal OTUs (taxonomic assignment) | A | | B | | IndVal ^2^ |
| **4242 (Sebacina sp)** | 0.9714 | | 0.6667 | | 0.805** |
| **376 (Agaricales sp)** | 0.9474 | | 0.6667 | | 0.795** |
| 250 (Sebacina sp) | 0.9412 | | 0.6667 | | 0.792* |
| 3852 (Sebacinaceae sp) | 0.8889 | | 0.6667 | | 0.770* |
| 1899 (Cortinarius sp) | 0.8571 | | 0.6667 | | 0.756* |
| 205 (Tomentella sp) | 0.8235 | | 0.6667 | | 0.741* |
| 738 (Preussia flanaganii); 1114 (Mortierella amoeboidea) | 0.8000 | | 0.6667 | | 0.730* |
| **Soil organic matter decomposition rate** |  | |  | |  |
| Strong (C:N ratio < 15) |  | |  | |  |
| **17 (Tomentella_atramentaria)** | 0.9968 | | 1.0000 | | 0.998*** |
| **69 (Ilyonectria mors-panacis)** | 0.9201 | | 1.0000 | | 0.959** |
| **38 (Thelephoraceae sp**) | 0.9936 | | 0.8889 | | 0.940** |
| **358 (Tomentella sp)** | 0.9949 | | 0.7778 | | 0.880** |
| **Table S7.** Continued |  | |  | |  |
| Fungal OTUs (taxonomic assignment) | A | | B | | IndVal ^2^ |
| **66 (Tomentella sp)** | 0.9779 | | 0.7778 | | 0.872** |
| **28 (Saccharicola sp)** | 0.9537 | | 0.7778 | | 0.861** |
| 49 (Cortinarius sp) | 0.9982 | | 0.6667 | | 0.816* |
| 315 (Inocybe sp) | 0.9970 | | 0.6667 | | 0.815* |
| 67 (Tomentella sp) | 0.8889 | | 0.6667 | | 0.770* |
| Moderate (C:N ratio: 15 – 20) |  | |  | |  |
| **33 (Cladophialophora sp)** | 0.9901 | | 1.0000 | | 0.995*** |
| **16 (Russulaceae sp)** | 0.9865 | | 1.0000 | | 0.993** |
| **112 (Cladophialophora sp)** | 0.8970 | | 1.0000 | | 0.947** |
| 75 (Mycosphaerellaceae sp) | 0.9844 | | 0.8889 | | 0.935* |
| **95 (Cryptosporiopsis brunnea)** | 0.9748 | | 0.8889 | | 0.931** |
| 32 (Cenococcum geophilum) | 0.9596 | | 0.8889 | | 0.924* |
| **246 (Cladophialophora sp)** | 1.0000 | | 0.8333 | | 0.913*** |
| **Table S7.** Continued |  | |  | |  |
| Fungal OTUs (taxonomic assignment) | A | | B | | IndVal ^2^ |
| **82 (Aspergillus amstelodami)** | 0.8580 | | 0.9444 | | 0.900** |
| 97 (Herpotrichiellaceae sp) | 0.9551 | | 0.8333 | | 0.892* |
| **60 (Lactarius sp)** | 0.9952 | | 0.7778 | | 0.880** |
| **453 (Oidiodendron griseum)** | 0.9286 | | 0.8333 | | 0.880** |
| 46 (Herpotrichiellaceae sp) | 0.9863 | | 0.7778 | | 0.876* |
| 78 (Dothideomycetes sp) | 0.9101 | | 0.8333 | | 0.871* |
| 237 (Chaetothyriales sp) | 0.8909 | | 0.8333 | | 0.862* |
| 359 (Cryptosporiopsis sp) | 1.0000 | | 0.6667 | | 0.816* |
| 51 (Hygrophorus cossus) | 0.9977 | | 0.6667 | | 0.816* |
| 103 (Atractiellales sp) | 0.8351 | | 0.7778 | | 0.806* |
| 407 (Sordariomycetes sp) | 0.9322 | | 0.6111 | | 0.755* |
| 72 (Russula sp) | 0.8500 | | 0.6667 | | 0.753* |
|  |  | |  | |  |
| **Table S7.** Continued |  | |  | |  |
| Fungal OTUs (taxonomic assignment) | A | | B | | IndVal ^2^ |
| **Soil available P content** |  | |  | |  |
| Low (mg P_available_ / kg soil < 10) |  | |  | |  |
| 903 (Thelephoraceae_sp) | 1.0000 | | 0.6667 | | 0.816* |
| **780 (Tomentella atramentaria)** | 0.9971 | | 0.6667 | | 0.815** |
| 148 (Tomentella sp) | 0.9893 | | 0.6667 | | 0.812* |
| 1133 (Ilyonectria mors-panacis) | 0.9259 | | 0.6667 | | 0.786* |
| 928 (Sordariales sp) | 0.8824 | | 0.6667 | | 0.767* |
| 536 (Tomentella atramentaria) | 0.8712 | | 0.6667 | | 0.762* |
| 759 (Ilyonectria mors-panacis) | 0.8434 | | 0.6667 | | 0.750* |
| Moderate (mg P_available_ / kg soil: 11 – 25) |  | |  | |  |
| 93 (Russulaceae sp) | 0.9757 | | 0.9333 | | 0.954* |
| High (mg P_available_ / kg soil > 25 – 49) |  | |  | |  |
| **362 (Cenococcum geophilum)** | 0.9398 | | 1.0000 | | 0.969*** |
| **Table S7.** Continued |  | |  | |  |
| Fungal OTUs (taxonomic assignment) | A | | B | | IndVal ^2^ |
| **471 (Cenococcum geophilum)** | 0.9911 | | 0.8333 | | 0.909** |
| 171 (Sebacinaceae sp) | 0.9598 | | 0.8333 | | 0.894* |
| **1008 (Cenococcum geophilum)** | 0.9770 | | 0.6667 | | 0.807** |
| 599 (Sordariomycetes sp) | 0.9459 | | 0.6667 | | 0.794* |
| 321 (Unidentified fungi) | 0.8553 | | 0.6667 | | 0.755* |
| Very high (mg P_available_ / kg soil > 50) |  | |  | |  |
| **493 (Archaeorhizomyces sp)** | 0.9907 | | 1.0000 | | 0.995*** |
| **445 (Unidentified fungi)** | 0.9677 | | 1.0000 | | 0.984*** |
| **703 (Degelia plumbea)** | 0.9653 | | 1.0000 | | 0.982*** |
| **230 (Tomentella sp)** | 0.9135 | | 1.0000 | | 0.956** |
| **551 (Pezizales sp)** | 0.8889 | | 1.0000 | | 0.943** |
| **1137 (Cladophialophora chaetospira)** | 0.8451 | | 1.0000 | | 0.919** |
| 572 (Sebacinales Group B) | 0.8061 | | 1.0000 | | 0.898* |
| **Table S7.** Continued |  | |  | |  |
| Fungal OTUs (taxonomic assignment) | A | | B | | IndVal ^2^ |
| 234 (Dothideomycetes sp); 330 (Mycosphaerellaceae sp); 513 (Helotiales sp); 857 (Agaricales sp); 1149 (Leotiomycetes sp); 1239 (Agaricales sp); 1397 (Helotiales sp); 1441 (Craterellus sp); 1987 (Craterellus sp); 2398 (Hysteriales sp); 2437 (Unidentified fungi); 2468 (Craterellus sp); 2606 (Leotiomycetes sp); 3213 (Sarcoleotia globosa); 4402 (Unidentified fungi); 4770 (Aspergillus sp); 5738 (Degelia plumbea); 5928 (Helotiales sp); 7290 (Unidentified fungi); 7887 (Sebacinales Group B); 10216 (Terfezia pini) | 1.0000 | | 0.6667 | | 0.816* |
| **1664 (Trichoglossum hirsutum)** | **0.9975** | | **0.6667** | | **0.815**** |
| 1143 (Craterellus sp); 734 (Pleosporales sp) | 0.9967 | | 0.6667 | | 0.815* |
| 832 (Russula foetens) | 0.9898 | | 0.6667 | | 0.812* |
| 6933 (Ascomycota sp) | 0.9783 | | 0.6667 | | 0.808* |
| 967 (Mycosphaerellaceae sp) | 0.9722 | | 0.6667 | | 0.805* |
| 490 (Oidiodendron chlamydosporicum) | 0.9699 | | 0.6667 | | 0.804* |
| 1487 (Leotiomycetes sp) | 0.9667 | | 0.6667 | | 0.803* |
| **Table S7.** Continued |  | |  | |  |
| Fungal OTUs (taxonomic assignment) | A | | B | | IndVal ^2^ |
| 495 (Dothideomycetes_sp) | 0.9632 | | 0.6667 | | 0.801 * |
| 1124 (Oidiodendron sp) | 0.9589 | | 0.6667 | | 0.800* |
| 374 (Dothideomycetes_sp) | 0.9524 | | 0.6667 | | 0.797* |
| 2236 (Helotiales sp) | 0.9474 | | 0.6667 | | 0.795* |
| 1073 (Helotiales sp) | 0.9130 | | 0.6667 | | 0.780* |
| 1621 (Lachnum sp) | 0.9091 | | 0.6667 | | 0.778* |
| 482 (Leotiomycetes sp); 2720 (Cryptosporiopsis brunnea) | 0.9000 | | 0.6667 | | 0.775* |
| 2684 (Ascomycota sp) | 0.8824 | | 0.6667 | | 0.767* |
| 2844 (Saccharomycetales sp) | 0.8571 | | 0.6667 | | 0.756* |

^1^ Only pH, C:N ratio and available P content were considered because of their significance as drivers of EcM-related fungal community structure. Soil categories were defined according the Natural Ressources conservation service (<https://www.nrcs.usda.gov/>) and Oregon State University (<http://extension.oregonstate.edu/>).

^2^ For each soil category, OTU are sorted by decreasing IndVal value index. Only fungal OTUs with A (specificity) and B (sensitivity) superior to 0.8 and 0.6, respectively, are considered. ‘***’ corresponds to *P* < 0.001; ‘**’ *P* < 0.01; ‘*’ *P* < 0.05; ‘NS’ *P* > 0.05. OTUs with a *P* < 0.01 are indicated in bold.
