# Supplementary figures and images for: Habitat- and soil-related drivers of the root-associated fungal community of Quercus suber in the Northern Moroccan forest
Source: PLoS One. 2017 Nov 20;12(11):e0187758. doi: 10.1371/journal.pone.0187758 (PMC5695781; doi:10.1371/journal.pone.0187758)

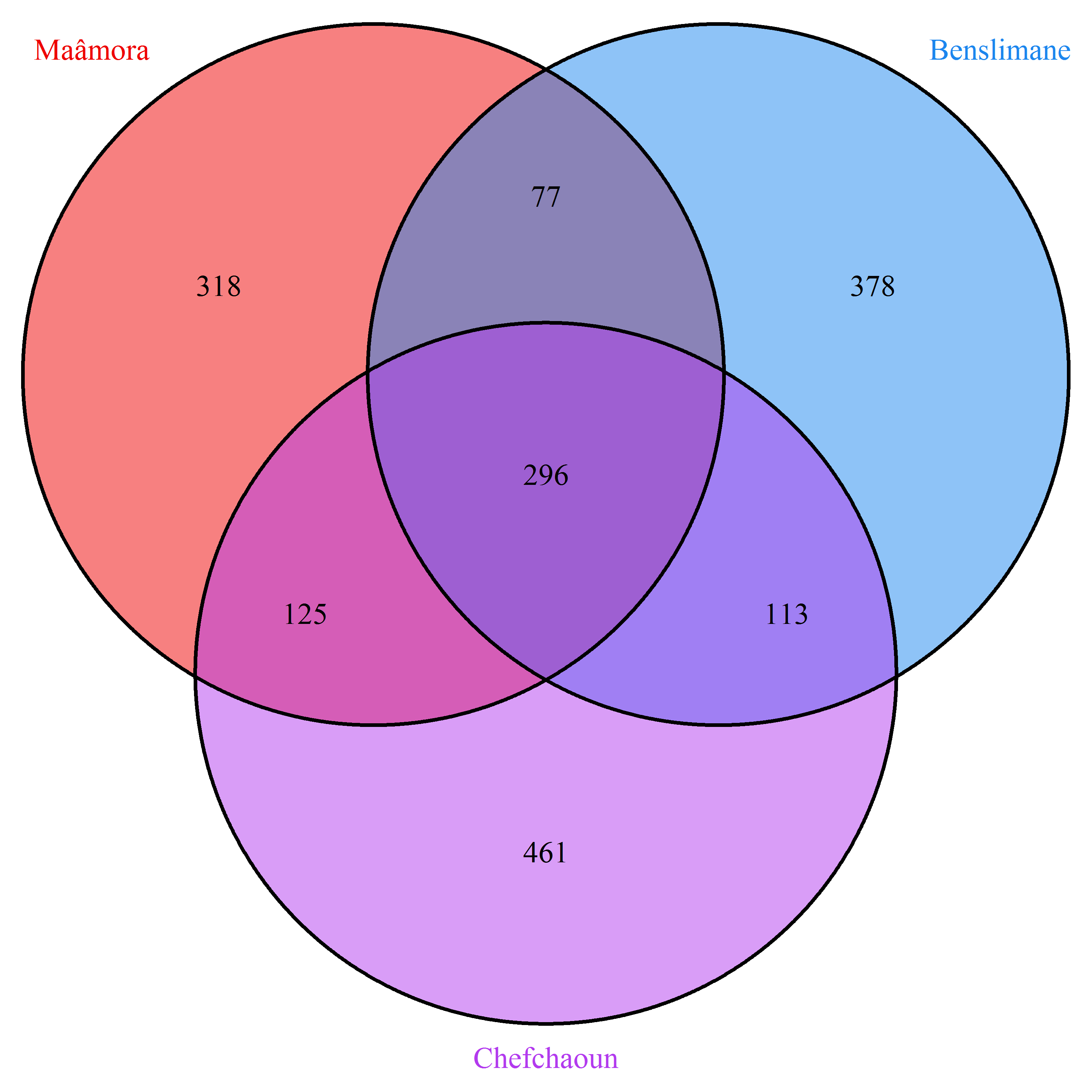

Supplement: S1 Fig — (TIFF) [file pone.0187758.s008.tiff]
